# Proceedings of the 23^rd^ Paediatric Rheumatology European Society Congress: part three: Genoa, Italy. 28 September – 01 October 2016

**DOI:** 10.1186/s12969-017-0143-7

**Published:** 2017-05-30

**Authors:** Riccardo Papa, Alessandro Consolaro, Francesca Minoia, Roberta Caorsi, Gianmichele Magnano, Marco Gattorno, Angelo Ravelli, Paolo Picco, Roberto Pillon, Denise Pires Marafon, Lidia Meli, Claudia Bracaglia, Andrea Taddio, Fabrizio De Benedetti, Enes Turan, Sara Sebnem Kilic, Yasuhiko Itoh, Tomoko Shigemori, Shingo Yamanishi, Hidehiko Nagasaki, Ela Tarakci, Nilay Arman, Devrim Tarakci, Yusuf S. Akgul, Ozgur Kasapcopur, Emily Wilson, Hanna Lythgoe, Eve Smith, Jenny Preston, Michael W. Beresford, Lynn R. Spiegel, Jennifer Stinson, Mark Connelly, Adam Huber, Nadia Luca, Argerie Tsimicalis, Stephanie Luca, Naweed Tajuddin, Roberta Berard, Julie Barsalou, Sarah Campillo, Brian Feldman, Shirley Tse, Paul Dancey, Ciaran Duffy, Nicole Johnson, Patrick McGrath, Natalie Shiff, Lori Tucker, Charles Victor, Lynn R. Spiegel, Chitra Lalloo, Lauren Harris, Joseph Cafazzo, Lori Tucker, Kristin Houghton, Brian Feldman, Nadia Luca, Ronald Laxer, Jennifer Stinson, Nilay Arman, Ela Tarakci, Ozgur Kasapcopur, Madeleine Rooney, Roisin Campbell, Catherine Wright, Wineke Armbrust, Otto Lelieveld, Jolanda Tuinstra, Nico Wulffraat, Joyce Bos, Jeanette Cappon, Marion van Rossum, Mariët Hagedoorn, Anna Vermé, Ylva Lampela, Ayse Huri Ozdogan, S. Ugurlu, K. Barut, A. Androvic, O. Kasapçopu, Emily Wilson, Jody Etheridge, Eve Smith, Katie Dobson, Sue Kemp, Michael W. Beresford, AnnaCarin Horne, Karin Palmblad, Malin Höglund, Natalia Stepanenko, Svetlana Salugina, Evgeny Fedorov, Irina Nikishina, Maria Kaleda, Nilay Arman, Ela Tarakci, Kenan Barut, Amra Adrovic, Sezgin Sahin, Ozgur Kasapcopur, Nilay Arman, Ela Tarakci, Ozgur Kasapcopur, Laurence Toumoulin, Johnny Frossard, Stephanie Archimbaut, Anne Paitier, Rolande Guastalli, Severine Guillaume Czitrom, Sirirat Charuvanij, Chollada Chaiyadech, Takako Miyamae, Hisashi Yamanaka, Cecile Picard, Guillaume Thouvenin, Caroline Kannengiesser, Jean-Christophe Dubus, Nadia Jeremiah, Frédéric Rieux-Laucat, Bruno Crestani, Véronique Secq, Christelle Ménard, Martine Reynaud-Gaubert, Françoise Thivolet-Bejui, Philippe Reix, Alexandre Belot, Ezgi Deniz Batu, Hafize Emine Sonmez, Abdulsamet Erden, Ekim Z. Taskiran, Omer Karadag, Umut Kalyoncu, İbrahim Oncel, Berkan Kaplan, Zehra Serap Arici, Cagri Mesut Temucin, Haluk Topaloglu, Yelda Bilginer, Mehmet Alikasifoglu, Seza Ozen, Lien Van Eyck, Ellen De Langhe, Isabelle Jéru, Erika Van Nieuwenhove, Vasiliki Lagou, Paul J. Baker, Jocelyn Garcia-Perez, James Dooley, Lien De Somer, Raf Sciot, Pierre-Yves Jeandel, Julia Ruuth-Praz, Bruno Copin, Myrna Medley-Hashim, Andre Megarbane, Sinisa Savic, An Goris, Serge Amselem, Adrian Liston, Seth Masters, Carine Wouters, Nami Okamoto, Yuko Sugita, Kousuke Shabana, Takuji Murata, Hiroshi Tamai, Juliana Ferenczová, Erika Banóova, Pavol Mrážik, Veronika Vargova, Dubravko Bajramovic, Ksenija Stekic Novacki, Kristina Potocki, Marijan Frkovic, Marija Jelusic, Irina Nikishina, Olga Kostareva, Svetlana Arsenyeva, Maria Kaleda, Anna Shapovalenko, Lennart Jans, Nele Herregods, Jacob Jaremko, Rik Joos, Joke Dehoorne, Nele Herregods, Jacob Jaremko, Xenofon Baraliakos, Joke Dehoorne, Rik Joos, Lennart Jans, Sofia Ramiro, Julio C. Casasola-Vargas, Désirée van der Heijde, Robert Landewé, Ruben Burgos-Vargas, Ruben Burgos-Vargas, Shirley M. Tse, Gerd Horneff, Kristina Unnebrink, Jaclyn K. Anderson, Aysenur Paç Kisaarslan, Betül Sözeri, Zübeyde Gündüz, Gökmen Zararsız, Hakan Poyrazoğlu, Ruhan Düşünsel, Kazutaka Ouchi, Shinji Akioka, Hiroshi Kubo, Norio Nakagawa, Hajime Hosoi, Lovro Lamot, Fran Borovecki, Sanja Kapitanovic, Kristina Gotovac, Mandica Vidovic, Mirta Lamot, Edi Paleka Bosak, Miroslav Harjacek, Ricardo A. Russo, María M. Katsicas, Ruben Burgos Vargas, Ana L. Ortiz-Peyegahud, Zhang Pingping, Mou Yikun, Qi Jun, Jiang Yutong, Gu Jieruo, Mikhail M. Kostik, Shilova Ekaterina, Ilia Avrusin, Yuriy Korin, Olga Kopchak, Eugenia Isupova, Irina Chikova, Panova Tatyana, Margarita Dubko, Vera Masalova, Ludmila Snegireva, Tatyana Kornishina, Olga Kalashnikova, Vyacheslav Chasnyk, Mikhail M. Kostik, Irina Chikova, Eugenia Isupova, Margarita Dubko, Vera Masalova, Ludmila Snegireva, Tatyana Kornishina, Tatyana Likhacheva, Olga Kalashnikova, Vyacheslav Chasnyk, N. Ruperto, H. I. Brunner, P. Quartier, T. Constantin, E. Alexeeva, R. Schneider, I. Kone-Paut, K. Schikler, K. Marzan, N. Wulffraat, S. Padeh, V. Chasnyk, C. Wouters, J. B. Kuemmerle-Deschner, T. Kallinich, B. Lauwerys, E. Haddad, E. Nasonov, M. Trachana, O. Vougiouka, K. Leon, A. Speziale, K. Lheritier, E. Vritzali, A. Martini, D. Lovell, PRINTO/PRCSG, Nienke Ter Haar, Rianne Scholman, Wilco de Jager, Tamar Tak, Pieter Leliefeld, Bas Vastert, Sytze de Roock, Nienke Ter Haar, Rianne Scholman, Wilco de Jager, Ariane de Ganck, Nadia Ryter, Miha Lavric, Dirk Foell, Sytze de Roock, Bas Vastert, Renee F. Modica, Kathleen G. Lomax, Pamela Batzel, Armelle Cassanas, Melissa E. Elder, Rina Denisova, Ekaterina Alexeeva, Saniya Valieva, Tatyana Bzarova, Kseniya Isayeva, Tatyana Sleptsova, Olga Lomakina, Alexandra Chomahidze, Margarita Soloshenko, Meyry Shingarova, Elena Kachshenko, Wilco De Jager, Sebastiaan J. Vastert, Gerdien Mijnheer, Berent J. Prakken, Nico M. Wulffraat, Hafize E. Sönmez, Asuman N. Karhan, Ezgi D. Batu, Yelda Bilginer, Zehra S. Arıcı, Ersin Gümüş, Hülya Demir, Aysel Yüce, Seza Özen, Jasmina Ahluwalia, Bhavneet Bharti, Sweta Rajpal, Varun Uppal, Alaknanda Walia, Surjit S. Samlok, Narender Kumar, Clarissa C. Valões, Beatriz C. Molinari, Ana Claudia G. Pitta, Natali W. Gormezano, Sylvia C. Farhat, Kátia Kozu, Adriana M. Sallum, Simone Appenzeller, Ana Paula Sakamoto, Maria T. Terreri, Rosa M. Pereira, Claudia S. Magalhães, Cássia Maria Barbosa, Francisco Hugo Gomes, Eloisa Bonfá, Clovis A. Silva, Kubra Ozturk, Zelal Ekinci, Maie Helal, Natalia Cabrera, Alexandre Belot, Jean Christophe Lega, Jocelyne Drai, Rene Ecochard, O. V. Shpitonkova, N. S. Podchernyaeva, Y. O. Kostina, N. G. Dashkova, M. K. Osminina, Gozde Yucel, Sezgin Sahin, Amra Adrovic, Kenan Barut, Ela Tarakci, Ahmet Arvas, Nandini Moorthy, Ozgur Kasapcopur, Paraskevi Dimou, Angela Midgley, Matthew Peak, Simon C. Satchell, Rachael D. Wright, Michael W. Beresford, Rachel Corkhill, Eve M. Smith, Michael W. Beresford, Sagar Bhattad, Amit Rawat, Surjit Singh, Anju Gupta, Deepti Suri, Martin de Boer, Taco Kuijpers, Sagar Bhattad, Amit Rawat, Anju Gupta, Deepti Suri, Vignesh Pandiarajan, Surjit Singh, Sapna Sandal, Amit Rawat, Anju Gupta, Surjit Singh, Sebastian Giraldo, Roy Sanguino, Adriana S. Diaz, Selcuk Uzuner, Sezgin Sahin, Gizem Durcan, Amra Adrovic, Kenan Barut, Ali Guven Kilicoglu, Ayhan Bilgic, Kayhan Bahali, Ozgur Kasapcopur, Sezgin Sahin, Amra Adrovic, Kenan Barut, Sinem Durmus, Hafize Uzun, Ozgur Kasapcopur, Sezgin Sahin, Amra Adrovic, Kenan Barut, Nur Canpolat, Salim Caliskan, Lale Sever, Ozgur Kasapcopur, Tomomi Sato, Fuminori Kimura, Wafaa Suwairi, Reem Abdwani, Abdulaziz Al Rowais, Jubran Al qanatish, Abdulrahman Al Asiri, Kubra Ozturk, Zelal Ekinci, Ekaterina Gaidar, Mikhail Kostik, Margarita Dubko, Vera Masalova, Elena Serogodskaya, Ludmila Snegireva, Tatyana Nikitina, Vyacheslav Chasnyk, Olga Kalashnikova, Evgenia Isupova, Elham Sardar, Perrine Dusser, Antoine Rousseau, Marc Labetoulle, Emanuel Barreau, Bahram Bodaghi, Isabelle Kone-Paut, Ivan Foeldvari, Jordi Anton, Rosa Bou, Sheila Angeles-Han, Regitze Bangsgaard, Gabriele Brumm, Tamas Constantin, Clive Edelsten, Jens Klotsche, Kirsten Minden, Elisabetta Miserocchi, Susan Nielsen, Gabriele Simonini, Arnd Heiligenhaus, Ivan Foeldvari, Jordi Anton, Rosa Bou, Sheila Angeles-Han, Regitze Bangsgaard, Gabriele Brumm, Tamas Constantin, Clive Edelsten, Jens Klotsche, Kirsten Minden, Elisabetta Miserocchi, Susan Nielsen, Gabriele Simonini, Arnd Heiligenhaus, Ivan Foeldvari, Jordi Anton, Rosa Bou, Sheila Angeles-Han, Regitze Bangsgaard, Gabriele Brumm, Tamas Constantin, Clive Edelsten, Jens Klotsche, Kirsten Minden, Elisabetta Miserocchi, Susan Nielsen, Gabriele Simonini, Arnd Heiligenhaus, Ivan Foeldvari, Jordi Anton, Rosa Bou, Sheila Angeles-Han, Regitze Bangsgaard, Gabriele Brumm, Tamas Constantin, Clive Edelsten, Jens Klotsche, Kirsten Minden, Elisabetta Miserocchi, Susan Nielsen, Gabriele Simonini, Arnd Heiligenhaus, Juan Manuel Mosquera Angarita, Rosa Bou, Carmen Garcia de Vicuña, Maria Victoria Hernandez, Alfredo Adan, Victor Llorens, Rosa Alcobendas, Susana Noval, Juan Carlos Lopez Robledillo, Isabel Valls, Mari Carmen Pinedo, Alejandro Fonollosa, Jaime de Inocencio, Pilar Tejada, Beatriz Bravo, Manuel Torribio, María Jesús García de Yebenes, Jordi Antón, Uveitis Working Group of the Spanish Pediatric Rheumatology Society, Lorenza Maria Argolini, Irene Pontikaki, Maria Orietta Borghi, Laura Cesana, Elisabetta Miserocchi, Barbara Castiglioni, Maurizio Gattinara, Pierluigi Meroni, Pierre Quartier, Veronique Despert, Sylvaine Poignant, Amandine Baptiste, Caroline Elie, Isabelle Kone-Paut, Alexandre Belot, Laurent Kodjikian, Dominique Monnet, Michel Weber, Bahram Bodaghi, Laura Moal, Antoine Rousseau, LuuLy Pham, Emmanuel Barreau, Cherif Titah, Pascal Dureau, Marc Labetoulle, Bahram Bodaghi, Severine Guillaume Czitrom, Vanessa Cecchin, Maria Elisabetta Zannin, Daniele Ferrari, Francesco Comacchio, Irene Pontikaki, Claudia Bracaglia, Rolando Cimaz, Fernanda Falcini, Antonella Petaccia, Stefania Viola, Luciana Breda, Francesco La Torre, Fabio Vittadello, Giorgia Martini, Francesco Zulian, Caroline Galeotti, Guillaume Sarrabay, Olivier Fogel, Isabelle Touitou, Bahram Bodaghi, Corinne Miceli-Richard, Isabelle Koné-Paut, Hala Etayari, Hashad Soad, Ihab El Kadry, Habibullah Eatamadi, Kais AlAlgawi, Mustafa Al Maini, Khulood Khawaja, Sophie Van den Berghe, Ilse de Schryver, Ann Raes, Rik Joos, Joke Dehoorne, Lídia L. C. Teixeira, Ana Duarte, Sandra Sousa, Filipe Vinagre, Maria J. Santos, Nataly S. Shevchenko, Ludmila F. Bogmat, Marina V. Demyanenko, Navdha R. Ramchurn, Mark Friswell, Rebecca A. James, Lucy R. Wedderburn, Clive Edelsten, Reshma Pattani, Clarissa A. Pilkington, Sandrine Compeyrot-Lacassagne, Rebecca A. James, Sandrine Compeyrot-Lacassagne, Clive Edelsten, Reshma Pattani, Clarissa A. Pilkington, Lucy R. Wedderburn, Ana V. Villarreal, Nydia Acevedo, Enrique Faugier, Rocio Maldonado, Dilek Yılmaz, Hilal Bektaş Uysal, Evgeny Fedorov, Svetlana Salugina, Elena Kamenets, Ekaterina Zaharova, Stefka Radenska-Lopovok, Joao Nascimento, Helena Sofia, Carla Zilhão, Rui Almeida, Margarida Guedes, Kubra Ozturk, Murat Deveci, Zelal Ekinci, Svetlana Rodionovskaya, Vera Vinnikova, Svetlana Salugina, Evgeny Fedorov, Irina Tsymbal, Edyta Olesińska, Jacek Postępski, Agnieszka Mroczkowska-Juchkiewicz, Agnieszka Pawłowska-Kamieniak, Beata Chrapko, Damjana Ključevšek, Nina Emeršič, Nataša Toplak, Tadej Avčin, Faina Rokhlina, Galina Glazyrina, Natalia Kolyadina, Kwangnam Kim, Sinae Eom, Daeyoung Kim, Jungwoo Rhim, Francesca Ricci, Paola Montesano, Barbara Bonafini, Veronica Medeghini, Ilaria Parissenti, Antonella Meini, Marco Cattalini, Paolo Airò, Nataliya Panko, Nataliya Shevchenko, Iryna Lebec, Yevgeniya Zajceva, Sara Rostlund, Marie André, Takuma Hara, Takayuki Kishi, Yumi Tani, Aki Hanaya, Takako Miyamae, Satoru Nagata, Hisashi Yamanaka, Velma Selmanovic, Aida Omercahic-Dizdarevic, Adisa Cengic, Almira Cosickic, Aida Omerčahić Dizdarević, Gemma Lepri, Paolo Picco, Clara Malattia, Eleonora Bellucci, Marco Matucci-Cerinic, Fernanda Falcini, Margarita Dubko, Anton Solovyev, Elena Fedotova, Rocio Maldonado, Enrique Faugier, Ana Victoria Villarreal, Nydia Acevedo, Talia Diaz, Yuridiana Ramirez, Teresa Giani, Achille Marino, Gabriele Simonini, Rolando Cimaz, Daniel Hunt, Muthana Al Obaidi, Veli Veli, Charalampia Papadopoulou, Jochen Kammermeier, Edyta Olesińska, Anna Poluha, Jacek Postępski, Gangadhara C. Bharmappanavara, Alison Kelly, Lindsay Shaw, Teresa Giani, Giovanna Ferrara, Michele Luzzati, Achille Marino, Mattia Giovannini, Gabriele Simonini, Rolando Cimaz, Liliana Jurado, Sebastian Giraldo, Juliana Chamorro, Lorena Sarmiento, Adriana S. Diaz, Veronica Medeghini, Francesca Ricci, Paola Montesano, Barbara Bonafini, Ilaria Parissenti, Antonella Meini, Ester Conversano, Marco Cattalini, Maria Francesca Gicchino, Giulia Macchini, Carmela Granato, Assunta Tirelli, Alma N. Olivieri, Marija Perica, Lana Tambić Bukovac, Ludmila F. Bogmat, Nataly S. Shevchenko, Marina V. Demyanenko, Reza Sinaei, Vadood Javadi Parvaneh, Reza Shiari, Khosro Rahmani, Fatemeh F. Mehregan, Mehrnoush Hassas Yeganeh, Inmaculada Calvo Penadés, Berta López Montesinos, Ma Isabel González Fernández, Adriana Rodríguez Vidal, Anand Prahalad Rao, Ayesha Romana, Jyothi Raghuram, Ankur Kumar, Deepti Suri, Vishali Gupta, Amit Rawat, Surjit Singh, Elif Comak, Gülşah Kaya Aksoy, Aygen Yılmaz, Atike Atalay, Mustafa Koyun, Reha Artan, Sema Akman, Maria Francesca Gicchino, Giulia Macchini, Carmela Granato, Alma N. Olivieri, Maria I. Kaleda, Irina P. Nikishina, Sergei K. Soloviev, Victor A. Malievsky, Ekaterina V. Nikolaeva, Teresa Giani, Achille Marino, Gabriele Simonini, Rolando Cimaz, Agnieszka Gazda, Beata Kołodziejczyk, Lidia Rutkowska-Sak, Angela Mauro, Teresa Giani, Gabriele Simonini, Rolando Cimaz, Maria Francesca Gicchino, Pierluigi Marzuillo, Stefano Guarino, Alma N. Olivieri, Angela La Manna

**Affiliations:** 1 Pediatria II, Reumatologia, Istituto Giannina Gaslini, Genoa, Italy; 2 Radiologia, Istituto Giannina Gaslini, Genoa, Italy; 3 University of Trieste, Trieste, Italy; 4 Division of Rheumatology, Ospedale Pediatrico Bambino Gesù IRCCS, Roma, Italy; 5 Institute for Maternal and Child Health - IRCCS “Burlo Garofolo”, Trieste, Italy; 6 Pediatric Rheumatology, Uludag University Medical Faculty, Bursa, Turkey; 7 Department of Pediatrics, Nippon Medical School, Tokyo, Japan; 8 Division of Physiotherapy and Rehabilitaiton, Faculty of Health Sciences, Istanbul University, Istanbul, Turkey; 9 Department of Computer Engineering, Gebze Institute of Technology, Kocaeli, Turkey; 10 Department of Pediatric Rheumatology, Medical Faculty of Cerrahpasa, Istanbul University, Istanbul, Turkey; 11 Department of Paediatric Rheumatology and Psychology, Alder Hey Children’s NHS Foundation Trust, Liverpool, UK; 12 Department of Women’s and Children’s Health, Institute of Translational Medicine, Liverpool, UK; 13 UK Experimental Arthritis Treatment Centre for Children, University of Liverpool, Liverpool, UK; 14 Rheumatology, Hospital for Sick Children, Toronto, Canada; 15 Pediatrics, University of Toronto, Toronto, Canada; 16 Child Evaluative Health Sciences, The Hospital for Sick Children, Toronto, Canada; 17 Lawrence S. Bloomberg Faculty of Medicine, University of Toronto, Toronto, Canada; 18 Psychology, University of Kansas Medical Center, Lawrence, KS USA; 19 Rheumatology, IWK Health Center, Halifax, Canada; 20 Rheumatology, Alberta children’s Hospital, Calgary, Canada; 21 Ingram School of Nursing, Mcgill University, Montreal, Canada; 22 Child Health Evaluative Sciences, Hospital for Sick Children, Toronto, Canada; 23 Rheumatology, Children’s Hospital of Western Ontario, London, Canada; 24 Rheumatology, CHU Ste-Justine, Montreal, Canada; 25 Rheumatology, Montreal Children’s Hospital, Montreal, Canada; 26 Pediatrics, Memorial University of Newfoundland, St John’s, Canada; 27 Pediatrics, Children’s Hospital of Eastern Ontario, Ottawa, Canada; 28 Research, Innovation and Knowledge Translation, IWK Health Center, Halifax, Canada; 29 Pediatrics, University of Florida, Gainesville, FL USA; 30 Rheumatology, BC Children’s Hospital, Vancouver, Canada; 31 Institute of Health Policy, Management and Evaluation, University of Toronto, Toronto, Canada; 32 Pediatrics, University of Toronto, Toronto, Canada; 33 Rheumatology, The Hospital for Sick Children, Toronto, Canada; 34 Lawrence S. Bloomberg Faculty of Nursing, University of Toronto, Toronto, Canada; 35 Child Health Evaluative Sciences, The Hospital for Sick Children, Toronto, Canada; 36 Centre for Global eHealth Innovation, University Health Network, Toronto, Canada; 37 Pediatrics, University of British Columbia, Vancouver, Canada; 38 Rheumatology, Hospital for Sick Children, Toronto, Canada; 39 Rheumatology, Alberta Children’s Hospital, Calgary, Canada; 40 Rheumatology, The Hospital for Sick Children, Toronto, Canada; 41 Faculty of Health Sciences, Division of Physiotherapy And Rehabilitation, Istanbul University, Istanbul, Turkey; 42 Department of Pediatric Rheumatology, Medical Faculty of Cerrahpasa, Istanbul University, Istanbul, Turkey; 43 Center Experimental Medicine, Queens University Belfast, Belfast, UK; 44 Pharmacy, Belfast Hospital Trust, Belfast, UK; 45 Arthritis Care, Belfast, UK; 46 Beatrix Children’s Hospital, Department of Pediatric Rheumatology and immunology, Groningen, Netherlands; 47 Center for Rehabilitation, Groningen, Netherlands; 48 Department of Health Sciences, University of Groningen, University Medical Center Groningen, Groningen, Netherlands; 49 Department of Pediatric Immunology, University Medical Center Utrecht, Wilhelmina Children’s Hospital, Utrecht, Netherlands; 50 Location: Dr. Jan van Breemenstraat, Reade, Center for Rehabilitation and Rheumatology, Amsterdam, Netherlands; 51 Department of Health Sciences, University of Groningen, University Medical Center Groningen, Groningen, Netherlands; 52 Pediatric Rheumathology, Karolinska, Sweden; 53 Youth clinic, Södersjukhuset, Stockholm, Sweden; 54 Department of Adult Rheumatology, Cerrahpasa Medical Faculty, İstanbul, Turkey; 55 Department of Pediatric Rheumatology, Cerrahpasa Medical Faculty, İstanbul, Turkey; 56 Department of Pediatric Rheumatology, Cerrahpasa Medical Faculty, İstanbul, Turkey; 57 Department of Paediatric Rheumatology and Psychology, Alder Hey Children’s NHS Foundation Trust, Liverpool, UK; 58 Department of Women’s and Children’s Health, Institute of Translational Medicine, University of Liverpool, Liverpool, UK; 59 Pediatric rheumathology, Karolinska Hospital, Stockholm, Sweden; 60 Nasonova Research Institute of Rheumatology, Moscow, Russian Federation; 61 Faculty of Health Sciences, Division of Physiotherapy and Rehabilitaiton, Medical Faculty of Cerrahpasa, Istanbul University, Istanbul, Turkey; 62 Department of Pediatric Rheumatology, Medical Faculty of Cerrahpasa, Istanbul University, Istanbul, Turkey; 63 Faculty of Health Sciences, Division of Physiotherapy and Rehabilitaiton, Istanbul University, Istanbul, Turkey; 64 Department of Pediatric Rheumatology, Medical Faculty of Cerrahpasa, Istanbul University, Istanbul, Turkey; 65 AJIADOS, Le Kremlin Bicetre Cedex, France; 66 Service de Medecine des Adolescents, CHU DE BICETRE, Le Kremlin Bicetre Cedex, France; 67 Department of Pediatrics, Siriraj Hospital, Mahidol University, Bangkok, Thailand; 68 Institute of Rheumatology, Tokyo Women’s Medical University, Tokyo, Japan; 69 Pathology, Hospices civils de Lyon, Hôpital Femme-Mère-Enfant, Lyon, France; 70 Inserm U1111, Lyon, France; 71 Pediatric Pulmonology, Assistance publique-Hôpitaux de Paris, Paris, France; 72 Genetic, Assistance publique-Hôpitaux de Paris, Paris, France; 73 Assistance publique-Hôpitaux de Marseille, Marseille, France; 74 Institut Imagine, hôpital Necker, Paris, France; 75 INSERM, Unité 1152, Université de Paris Diderot, Paris, France; 76 Pathology, Assistance publique-Hôpitaux de Marseille, Hôpital de la Timone, Marseille, France; 77 Pediatric Pulmonology, Assistance publique-Hôpitaux de Paris, hôpital Armand Trousseau, Paris, France; 78 Pulmonology Department, CHU Nord, Assistance publique-Hôpitaux de Marseille, Marseille, France; 79 Pediatric Pulmonology, Nephrology and Dermatology, Hospices civils de Lyon, Hôpital Femme-Mère-Enfant, Lyon, France; 80 Pediatric Rheumatology, Nephrology and Dermatology, Hospices civils de Lyon, Hôpital Femme-Mère-Enfant, Lyon, France; 81 Department of Pediatrics, Division of Rheumatology, Ankara, Turkey; 82 Department of Internal Medicine, Division of Rheumatology, Ankara, Turkey; 83 Department of Medical Genetics, Ankara, Turkey; 84 Department of Pediatrics, Division of Neurology, Ankara, Turkey; 85 Department of Neurology, Hacettepe University Faculty of Medicine, Ankara, Turkey; 86 Department of Immunology and Microbiology, University of Leuven, Leuven, Belgium; 87 Translational Immunology Laboratory, VIB Leuven, Belgium; 88 Department of Rheumatology, University Hospitals Leuven, Leuven, Belgium; 89 Université Pierre et Marie Curie-Paris, UMR_S933, Paris, France; 90 Service de Génétique et d’Embryologie médicales, Assistance Publique Hôpitaux de Paris, Hôpital Trousseau, Paris, France; 91 Department of Neurosciences, University of Leuven, Leuven, Belgium; 92 Inflammation division, The Walter and Eliza Hall Institute of Medical Research, Parkville, Australia; 93 Department of Medical Biology, University of Melbourne, Parkville, Australia; 94 Department of pediatrics, University Hospitals Leuven, Leuven, Belgium; 95 Department of pathology, University Hospitals Leuven, Leuven, Belgium; 96 Département de Médecine Interne, Hôpital Archet 1, Nice, France; 97 Department of Life and Earth Sciences, Lebanese University, Beirut, Lebanon; 98 Al-Jawhara Center, Arabian Gulf University, Manama, Bahrain; 99 Department of Clinical Immunology and Allergy, St James’s University Hospital, Leeds, UK; 100 Department of Pediatrics, Graduate School of Medicine, Takatsuki-city, Japan; 101 Pediatrics, Osaka Medical and Pharmaceutical University, Takatsuki-city, Japan; 102 Department of Paediatrics and Adolescent Medicine, Šafárik University and children faculty hospital in košice, Košice, Slovakia; 103 Department of radiology, University Hospital Center Zagreb, Zagreb, Croatia; 104 Department of pediatrics, University Hospital Center Zagreb, Zagreb, Croatia; 105 V.A.Nasonova research Institute of Rheumatology, Moscow, Russian Federation; 106 Ghent University Hospital, Gent, Belgium; 107 University of Alberta, Edmonton, Canada; 108 Department of Radiology and Medical Imaging, Ghent University Hospital, Ghent, Belgium; 109 Department of Radiology & Diagnostic Imaging, University of Alberta Hospital, Edmonton, Canada; 110 Rheumazentrum Ruhrgebiet, Ruhr-University Bochum, Herne, Germany; 111 Department of Pediatric Rheumatology, Ghent University Hospital, Ghent, Belgium; 112 LUMC, Leiden, Netherlands; 113 Hospital General de Mexico, Mexico, Mexico; 114 ARC, Amsterdam, Netherlands; 115 Hospital General de Mexico, Universidad Nacional Autonoma de Mexico, Mexico City, Mexico; 116 University of Toronto, The Hospital for Sick Children, Ontario, Canada; 117 Asklepios Klinik Sankt Augustin, Sankt Augustin, Germany; 118 AbbVie Deutschland GmbH & Co. KG, Ludwigshafen, Germany; 119 AbbVie Inc, North Chicago, IL USA; 120 Pediatric Rheumatology, Erciyes University, Kayseri, Turkey; 121 Bioistatistic, Erciyes University, Kayseri, Turkey; 122 Pediatrics, Kyoto Prefectural University of Medicine, Kyoto, Japan; 123 Clinical Hospital Center Sestre Milosrdnice, Zagreb, Croatia; 124 University of Zagreb School of Medicine, Zagreb, Croatia; 125 Ruđer Bošković Institute, Zagreb, Croatia; 126 Immunology & Rheumatology, Hospital de Pediatría Garrahan, Buenos Aires, Argentina; 127 Rheumatology, Mexico, Mexico; 128 Hospital General de Mexico, Mexico, Mexico; 129 Pediatrics, The Third Affiliated Hospital of Sun Yat-Sen University, Guangzhou, China; 130 Rheumatology, The Third Affiliated Hospital of Sun Yat-Sen University, Guangzhou, China; 131 Hospital Pediatry, Saint-Petersburg State Pediatric Medical University, Saint-Petersburg, Russian Federation; 132 Pediatry, Kirov’s regional children’s hospital, Kirov, Russian Federation; 133 Pediatry, Leningrad regional children’s clinical hospital, Saint-Petersburg, Russian Federation; 134 Hospital Pediatry, Saint-Petersburg State Pediatric Medical University, Saint-Petersburg, Russian Federation; 135 PRINTO-Istituto Gaslini, Genoa, Italy; 136 PRCSG, Cincinnati, OH USA; 137 Necker-Enfants Malades Hospital, Paris, France; 138 Cliniques Universitaires Saint-Luc and Université Catholique de Louvain, Brussels, Belgium; 139 Novartis Pharmaceuticals Corporation, East Hanover, NJ USA; 140 Novartis Pharma AG, Basel, Switzerland; 141 Laboratory for Translational Immunology, University Medical Center Utrecht, Utrecht, Netherlands; 142 Pediatric Rheumatology, University Medical Center Utrecht, Utrecht, Netherlands; 143 Laboratory for Translational Immunology, Utrecht, Netherlands; 144 Pediatric Rheumatology, UMCU, Utrecht, Netherlands; 145 Biogazelle NV, Zwijnaarde, Belgium; 146 BÜHLMANN Laboratories AG, Basel, Switzerland; 147 Pediatric Rheumatology and Immunology, University of Muenster, Muenster, Germany; 148 Department of Pediatrics, University of Florida, Gainesville, FL USA; 149 Immunology and Dermatology Medical Affairs, Novartis Pharmaceuticals Corp, East Hanover, NJ USA; 150 Treato, Princeton, NJ USA; 151 Rheumatology, Scientific Center of Children’s Health, Moscow, Russian Federation; 152 I.M. Sechenov First Moscow State Medical University, Moscow, Russian Federation; 153 Pediatric Immunology, University Medical Center Utrecht, Utrecht, Netherlands; 154 Department of Pediatrics, Division of Rheumatology, Hacettepe University Faculty Of Medicine, Ankara, Turkey; 155 Department of Pediatrics, Division of Gastroenterology and Hepatology, Hacettepe University Faculty Of Medicine, Ankara, Turkey; 156 Hematology, Chandigarh, India; 157 Pediatrics, Postgraduate Institute of Medical Education and Research, Chandigarh, India; 158 Pediatric Rheumatology Unit, Faculdade de Medicina d Universidade de São Paulo, São Paulo, SP Brazil; 159 Pediatric Rheumatology Division, Faculdade de Medicina d Universidade de São Paulo, São Paulo, SP Brazil; 160 Pediatric Rheumatology Unit, UNICAMP, Campinas, SP Brazil; 161 Pediatric Rheumatology Unit, UNIFESP, São Paulo, SP Brazil; 162 Pediatric Rheumatology Unit, UNESP, Botucatu, SP Brazil; 163 Pediatric Rheumatology Unit, Hospital Darcy Vargas, São Paulo, SP Brazil; 164 Pediatric Rheumatology Unit, USP-Ribeirão Preto, Ribeirão Preto, SP Brazil; 165 Department of Pediatrics Rheumatology, Kocaeli University Faculty of Medicine, Kocaeli, Turkey; 166 Alexandria University, Alexandria, Egypt; 167 Hôpital Femme Mère Enfant, Bron, Lyon, France; 168 Internal Medicine and Vascular Department, CHU Lyon Sud, Lyon, France; 169 Laboratory of Biochemistry and Molecular Biology, Lyon Sud, Lyon, France; 170 Biostatistics Health Laboratory. “Claude Bernard” University of Lyon 1, UCBL1, Lyon, France; 171 Pediatric Department, I.M. Sechenov First Moscow State Medical University, Moscow, Russian Federation; 172 Pediatric Rheumatology, Istanbul University, Cerrahpasa Medical Faculty, Istanbul, Turkey; 173 Pediatrics, Istanbul University, Cerrahpasa Medical Faculty, Istanbul, Turkey; 174 Pediatric Rheumatology, Rutgers University, Robert Wood Johnson Medical School, Liverpool, UK; 175 Women’s and Children’s Health, Institute of Translational Medicine, University of Liverpool, Liverpool, UK; 176 Alder Hey Children’s Hospital, Liverpool, UK; 177 Academic Renal Unit, University of Bristol, Bristol, UK; 178 Woman and Childrens Health, Liverpool University, Liverpool, UK; 179 Pediatrics, PGIMER, Chandigarh, India; 180 Blood lab, Sanquin Blood Supply Foundation, Amsterdam, Netherlands; 181 Pediatrics, PGIMER, Chandigarh, India; 182 Pediatrics, PGIMER, Chandigarh, India; 183 Rheumatology and Inmunology, Hospital Militar Central, Bogota, Colombia; 184 School of Medicine, Universidad Militar Nueva Granada, Bogota, Colombia; 185 Cardiology Department, Hospital de La Misericordia, Bogota, Colombia; 186 Rheumatology, Hospital de La Misericordia, Bogota, Colombia; 187 Rheumatology, Care for Kids, Bogota, Colombia; 188 Pediatrics, Bezmialem University, Istanbul, Turkey; 189 Pediatric Rheumatology, Istanbul University, Cerrahpasa Medical School, Istanbul, Turkey; 190 Child and Adolescent Psychiatry, Istanbul University, Cerrahpasa Medical School, Istanbul, Turkey; 191 Child and Adolescent Psychiatry, Bakirkoy Research and Training Hospital for Psychiatry, Neurology and Neurosurgery, Istanbul, Turkey; 192 Child and Adolescent Psychiatry, Necmettin Erbakan University, Meram Medical Faculty, Konya, Turkey; 193 Department of Pediatric Rheumatology, Istanbul University, Cerrahpasa Medical School, Istanbul, Turkey; 194 Department of Biochemistry, Istanbul University, Cerrahpasa Medical School, Istanbul, Turkey; 195 Department of Pediatric Rheumatology, Istanbul University, Cerrahpasa Medical School, Istanbul, Turkey; 196 Department of Pediatric Nephrology, Istanbul University, Cerrahpasa Medical School, Istanbul, Turkey; 197 Department of Pediatrics, Shiga University of Medical Science, Otsu, Japan; 198 Department of Obstetrics and Gynecology, Shiga University of Medical Science, Otsu, Japan; 199 Pediatrics, King Abdullah specialized children hospital, Riyadh, Saudi Arabia; 200 Pediatrics, Sultan Qaboos University Hospital, Maskat, Oman; 201 Pediatrics, Prince Sultan Military Medical City, Riyadh, Saudi Arabia; 202 Department of Pediatrics Rheumatology, Kocaeli University Faculty of Medicine, Kocaeli, Turkey; 203 Saint-Petersburg State Pediatric Medical University, Saint-Petersburg, Russian Federation; 204 Paediatric Rheumatology, Paris-Sud University Hospital, APHP, Paris, France; 205 Ophtalmology, Paris-Sud University Hospital, APHP, Paris, France; 206 Ophtalmology, Pitie-Salpetriere Hospital,University of Paris VI, Paris, France; 207 AM Schön Klinik Eilbek, Hamburg, Germany; 208 Universitat de Barcelona, Hospital Sant Joan de Deu, Barcelona, Spain; 209 Emory University, Atlanta, GA USA; 210 Juliane Marie Centret, Rigshospitalet, Copenhaven, Denmark; 211 Universitätsklinikum Hamburg-Eppendorf, Hamburg, Germany; 212 Semmelweis University, Budapest, Hungary; 213 Great Ormond Street Hospital for Children NHS Foundation Trust, London, UK; 214 German Rheumatism Research Center, Berlin, Germany; 215 Università Vita-Salute, Scientific Institute San Raffaele, Milano, Italy; 216 Universita degli studi Firenze, Firenze, Italy; 217 St. Franziskus Hospital, Muenster, Germany; 218 AM Schön Klinik Eilbek, Hamburg, Germany; 219 Universitat de Barcelona, Hospital Sant Joan de Deu, Barcelona, Spain; 220 Emory University, Atlanta, GA USA; 221 Juliane Marie Centret, Rigshospitalet, Copenhaven, Denmark; 222 Universitätsklinikum Hamburg-Eppendorf, Hamburg, Germany; 223 Semmelweis University, Budapest, Hungary; 224 Great Ormond Street Hospital for Children NHS Foundation Trust, London, UK; 225 German Rheumatism Research Center, Berlin, Germany; 226 Università Vita-Salute, Scientific Institute San Raffaele, Milano, Italy; 227 Universita degli studi Firenze, Firenze, Italy; 228 St. Franziskus Hospital, Muenster, Germany; 229 AM Schön Klinik Eilbek, Hamburg, Germany; 230 Universitat de Barcelona, Hospital Sant Joan de Deu, Barcelona, Spain; 231 Emory University, Atlanta, GA USA; 232 Juliane Marie Centret, Rigshospitalet, Copenhaven, Denmark; 233 Universitätsklinikum Hamburg-Eppendorf, Hamburg, Germany; 234 Semmelweis University, Budapest, Hungary; 235 Great Ormond Street Hospital for Children NHS Foundation Trust, London, UK; 236 German Rheumatism Research Center, Berlin, Germany; 237 Università Vita-Salute, Scientific Institute San Raffaele, Milano, Italy; 238 Universita degli studi Firenze, Firenze, Italy; 239 St. Franziskus Hospital, Muenster, Germany; 240 AM Schön Klinik Eilbek, Hamburg, Germany; 241 Universitat de Barcelona, Hospital Sant Joan de Deu, Barcelona, Spain; 242 Emory University, Atlanta, GA USA; 243 Juliane Marie Centret, Rigshospitalet, Copenhaven, Denmark; 244 Universitätsklinikum Hamburg-Eppendorf, Hamburg, Germany; 245 Semmelweis University, Budapest, Hungary; 246 Great Ormond Street Hospital for Children NHS Foundation Trust, London, UK; 247 German Rheumatism Research Center, Berlin, Germany; 248 Università Vita-Salute, Scientific Institute San Raffaele, Milano, Italy; 249 Universita degli studi Firenze, Firenze, Italy; 250 St. Franziskus Hospital, Muenster, Germany; 251 Hospital Sant Joan de Déu, Barcelona, Spain; 252 Hospital Clínic, Barcelona, Spain; 253 Hospital La Paz, Madrid, Spain; 254 Hospital Niño Jesus, Madrid, Spain; 255 Hospital Cruces, Bilbao, Spain; 256 Hospital 12 de Octubre, Madrid, Spain; 257 Hospital Virgen de las Nieves, Granada, Spain; 258 Instituto de Salud Musculoesqueletica, Madrid, Spain; 259 Rheumatology, Gaetano Pini Institute, Center of Pediatric Rheumatology, Chair of Rheumatology, University of Milan, Milan, Italy; 260 Experimental Laboratory of ImmunoRheumatologic Researches, Department of Clinical Sciences and Community Health, University of Milan, IRCCS Istituto Auxologico Italiano, Milan, Italy; 261 Ophthalmology and Visual Sciences, University Vita-Salute, Scientific Institute San Raffaele, Milan, Italy; 262 Ophtometrist, Department of Rheumatology, JIA Uveitis Center, Gaetano Pini Institute, Milan, Italy; 263 Unité d’Immuno-Hématologie et Rhumatologie Pédiatrique, Hôpital Necker-Enfants Malades, Paris, France; 264 Pediatrie, CHU, Rennes, France; 265 Pédiatrie, CHU, Nantes, France; 266 Biostatistiques, Hôpital Necker-Enfants Malades, Paris, France; 267 Pédiatrie, CHU, Kremlin-Bicetre, France; 268 Pédiatrie, Hopital femme-mere-enfant, Lyon, France; 269 Ophtalmologie, CHU, Lyon, France; 270 Ophtalmologie, CHU Cochin, Paris, France; 271 Ophtalmologie, CHU, Nantes, France; 272 Ophtalmologie, CHU Pitié Salpétrière, Paris, France; 273 Paediatrics, CHU de Bicetre, Le Kremlin Bicetre Cedex, France; 274 Ophthalmology, CHU de Bicetre, Le Kremlin Bicetre Cedex, France; 275 Ophthalmology, Fondation Rothschild, Paris, France; 276 Ophthalmology, CHU Pitie Salpetriere, Paris, France; 277 Service de Medecine des Adolescents, CHU de Bicetre, Le Kremlin Bicetre Cedex, France; 278 Department of Pediatrics, University of Padua, Padua, Italy; 279 ORCHIDEA Network (OculaR involvement in CHIldhood rheumatic DisEAses, Padua, Italy; 280 Pediatric Rheumatology, Bicêtre Hospital, Le Kremlin Bicêtre, France; 281 Genetics, CHU Montpellier, Montpellier, France; 282 Rheumatology, Cochin Hospital, Paris, France; 283 Ophtalmology, La Pitié Salpêtrière, Paris, France; 284 Pediatric rheumatology, Tripoli Children Hospital, Tripoli, Libya; 285 Academic Affair, Al Mafraq Hospital, Abu Dhabi, United Arab Emirates; 286 Ophthalmology Department, Sheikh Khalifa Medical City, Abu Dhabi, United Arab Emirates; 287 Ophthalmology Department, Al Mafraq Hospital, Abu Dhabi, United Arab Emirates; 288 Rheumatology Department, Al Mafraq Hospital, Abu Dhabi, United Arab Emirates; 289 Pediatric rheumatology, Pediatric Universitary Hospital, Gent, Belgium; 290 Pediatric ohthalmology, Universitary Hospital, Gent, Belgium; 291 Rheumatology, Hospital Garcia de Orta, Almada, Portugal; 292 Department of cardiorheumatology, Institute of Children and Adolescents Health Care, Kharkiv, Ukraine; 293 Paediatric Medicine, Great North Children’s Hospital, Newcastle upon Tyne, UK; 294 Paediatric Rheumatology, Great North Children’s Hospital, Newcastle upon Tyne, UK; 295 Department of Rheumatology, Great Ormond Street Hospital for Children NHS Foundation Trust, London, UK; 296 UCL Institute of Child Health, London, UK; 297 ARUK Centre for Adolescent Rheumatology, University College London, London, UK; 298 Department of Rheumatology, Great Ormond Street Hospital for Children NHS Foundation Trust, London, UK; 299 UCL Institute of Child Health, London, UK; 300 ARUK Centre for Adolescent Rheumatology, University College London, London, UK; 301 Pediatrics Rheumatology, Hospital Infantil de Mexico Federico Gómez, Ciudad de Mexico, Mexico; 302 Paediatric Rheumatology, Hospital Infantil de Mexico Federico Gomez, Ciudad de Mexico, Mexico; 303 Pediatric Nephrology, Adnan Menderes University Faculty of Medicine, Aydın, Turkey; 304 Internal Medicine, Adnan Menderes University Faculty of Medicine, Aydın, Turkey; 305 Nasonova Research Institute of Rheumatology, Moscow, Russian Federation; 306 FSBI “Research Centre for Medical Genetics”, Moscow, Russian Federation; 307 Pathological Anatomy, I.M.Sechenov First T Moscow State. Medical University, Moscow, Russian Federation; 308 Pediatrics, Centro Hospitalar Porto, Porto, Portugal; 309 Pediatrics, Hospital Vila Franca Xira, Vila Franca de Xira, Portugal; 310 Pediatric Rheumatology Unit, Centro Hospitalar Porto, Porto, Portugal; 311 Pediatrics, Hospital Pedro Hispano, Matosinhos, Portugal; 312 Department of Pediatrics Rheumatology, Kocaeli University Faculty of Medicine, Kocaeli, Turkey; 313 Department of Pediatrics Cardiology, Kocaeli University Faculty of Medicine, Kocaeli, Turkey; 314 Central Children’s Clinical Hospital of the Federal Medical-Biological Agency of Russia, Moscow, Russian Federation; 315 V.A.Nasonova Research Institute of Rheumatology, Moscow, Russian Federation; 316 Department of Paediatric Pulmonology and Rheumatology, Children’s University Hospital, Lublin, Poland; 317 Department of Paediatric Pulmonology and Rheumatology, Medical University of Lublin, Lublin, Poland; 318 Department of Paediatrics, Medical University of Lublin, Lublin, Poland; 319 Departament of Nuclear Medicine, Medical University of Lublin, Lublin, Poland; 320 Radiology Unit, University Children’s Hospital Ljubljana, Ljubljana, Slovenia; 321 Department of Allergology, Rheumatology and Clinical Immunology, University Children’s Hospital Ljubljana, Ljubljana, Slovenia; 322 Department of Allergology, Rheumatology and Clincal Immunology, University Children’s Hospital, Ljubljana, Slovenia; 323 Pediatric, SPbGPMU, St.Petersburg, Russian Federation; 324 South Ural Governmental Medical University, Chelyabinsk, Russian Federation; 325 Chelyabinsk Regional Clinical Paediatric Hospital, Chelyabinsk, Russian Federation; 326 Pediatrics, Hallym University Medical Center, AnYang City, Korea, Republic Of; 327 Pediatrics, Inje University, Goyang City, Korea, Republic Of; 328 Pediatrics, The Catholic University, Daejeon City, Korea, Republic Of; 329 Clinical and Experimental Sciences, Pediatric Clinic, University of Brescia, Brescia, Italy; 330 Clinical and Experimental Sciences, Immunology and Rheumatology Unit, University of Brrescia, Brescia, Italy; 331 Department of Pediatrics, V.N. Karazin Kharkov National University, Kharkov, Ukraine; 332 Department of Cardiorheumatology, SE “Institute of Adolescence and children health care of National Academy of Medical Sciences”, Kharkov, Ukraine; 333 Department of Cardiorheumatology, Kharkiv children’s city hospital N 24, Kharkov, Ukraine; 334 Master program in Clinical Medical Science, Departmet of Neurobiology, Care Sciences and Society, Karolinska Institute, Stockholm, Sweden; 335 Physiotherapy unit, Karolinska University Hospital, Stockholm, Sweden; 336 Institute of Rheumatology, Tokyo Women’s Medical University, Tokyo, Japan; 337 Department of Pediatrics, Tokyo Women’s Medical University, Tokyo, Japan; 338 Department for Allergology, Rheumatology and Clinical Immunology, Children’s Hospital University Clinical Center Sarajevo, Sarajevo, Bosnia and Herzegovina; 339 Children’s Hospital University Clinical Center Tuzla, Tuzla, Australia; 340 Allergology,rheumatology and Clinical immunology, Childrens hospital, Sarajevo, Bosnia and Herzegovina; 341 Department of Rheumatology, Transition Care, University of Florence, Florence, Italy; 342 Istituto Giannina Gaslini, Genova, Italy; 343 Istituto Giannina Gaslini, University of Genoa, Genova, Italy; 344 Saint-Petersburg Pediatric Medical University, Saint-Petersburg, Russian Federation; 345 Paediatric Rheumatology, Hospital Infantil de México Federico Gómez, Mexico City, Mexico; 346 Pediatric, A. Meyer Children Hospital, Florence, Italy; 347 Imperial College London, London, UK; 348 Paediatric Rheumatology, Great Ormond Street Hospital and UCL Institute of Child Health, London, UK; 349 Paediatric Gastroenterolog, Great Ormond Street Hospital and UCL Institute of Child Health, London, UK; 350 Department of Paediatric Pulmonology and Rheumatology, Children’s University Hospital, Lublin, Poland; 351 Department of Medical Genetics, Medical University of Lublin, Lublin, Poland; 352 Department of Paediatric Pulmonology and Rheumatology, Medical University of Lublin, Lublin, Poland; 353 Paediatric Rheumatology Department, Bristol Royal Hospital for Children, Bristol, UK; 354 Department of Dermatology, Bristol Royal Hospital for Children, Bristol, UK; 355 Pediatric, A. Meyer Children Hospital, Florence, Italy; 356 Pediatric, Burlo Garofalo, Trieste, Italy; 357 School of Medicine, Universidad Nacional de Colombia, Bogota, Colombia; 358 Rheumatology and Inmunology, Hospital Militar Central, Bogota, Colombia; 359 School of Medicine, Universidad Militar Nueva Granada, Bogota, Colombia; 360 Pediatric Nephrology, Hospital de la Misericoria, Bogota, Colombia; 361 School of Medicine, Fundación Universitaria Sanitas, Bogota, Colombia; 362 Rheumatology, Hospital de La Misericordia, Bogota, Colombia; 363 Rheumatology, Care for Kids, Bogota, Colombia; 364 Clinical and Experimental Sciences, Pediatric Clinic, University of Brescia, Brescia, Italy; 365 Department of Women, Chid and General and Specialistic Surgery, Second University of Naples, Naples, Italy; 366 Department of Pediatric and Adolescent Rheumatology, Children’s Hospital Srebrnjak, Zagreb, Croatia; 367 Department of Cardiorheumatology, Institute of Children and Adolescents Health Care, Kharkiv, Ukraine; 368 Pediatric Rheumatology, Mofid Children’s Hospital, Tehran, Islamic Republic of Iran; 369 Hospital Universitario Policlínico La Fe, Valencia, Spain; 370 Pediatric Rheumatology Clinic, Bangalore, India; 371 Indira Gandhi Institute of Child health, Bangalore, India; 372 Pediatrics, PGIMER, Chandigarh, India; 373 Ophthalmology, PGIMER, Chandigarh, India; 374 Pediatric Nephrology - Rheumatology, Akdeniz University, Antalya, Turkey; 375 Pediatric Gastroenterology, Hepatology & Nutrition, Akdeniz University, Antalya, Turkey; 376 Department of Women, Chid and General and Specialistic Surgery, Second University of Naples, Naples, Italy; 377 Pediatrics, V.A. Nasonova Research Institute of Rheumatology, Moscow, Russian Federation; 378 Pediatrics, Bashkortostan State Medical University, Ufa, Russian Federation; 379 Pediatric, A. Meyer Children Hospital, Florence, Italy; 380 Paediatric Rheumatology Clinic, National Institute of Geriatrics, Rheumatology and Rehabilitation, Warsaw, Poland; 381 Rheumatology Unit, Second University of Naples, Naples, Italy; 382 Rheumatology Unit, Anna Meyer Children’s Hospital, Florence, Italy; 383 Department of Women, Chid and General and Specialistic Surgery, Second University of Naples, Naples, Italy

## Poster Session: Miscellaneous rheumatic diseases

### P381 Transient periosteal hyperostosis with dysproteinemia (Goldbloom syndrome): two cases report

#### Riccardo Papa^1^, Alessandro Consolaro^1^, Francesca Minoia^1^, Roberta Caorsi^1^, Gianmichele Magnano^2^, Marco Gattorno^1^, Angelo Ravelli^1^, Paolo Picco^1^

##### ^1^Pediatria II, Reumatologia, Istituto Giannina Gaslini, Genoa, Italy; ^2^Radiologia, Istituto Giannina Gaslini, Genoa, Italy

###### **Presenting author:** Riccardo Papa

**Introduction:** Transient periosteal hyperostosis with dysproteinemia, also named Goldbloom syndrome (GS), is a rare pediatric disease characterized by recurrent crisis of bone pain, fever, increased inflammatory markers and dysproteinemia. From 1966 only few cases have been reported in the English literature.

**Objectives:** To better define the clinical and epidemiological features of GS.

**Methods:** We report clinical, laboratory and radiological features of our patients in Table 1, comparing with the English literature.

**Results:** Case 1 was a 9-year-old girl who presented daily crisis of bone pain at the lower limbs, associated with fever spikes, limping and nocturnal awakenings. Physical examination was normal. Laboratory tests showed mild anemia, thrombocytosis, increased inflammatory markers and high antibody levels against streptolysine O and DNase-B (ASO 4280 IU/ml and ADN-B 6310 UI/ml, respectively). Throat swab was positive for group A β-hemolytic streptococcus (GAS). Unusual dysproteinemia, characterized by hypoalbuminemia with increased a1, a2 and g globulinemia, was noted. X-ray evaluation of the lower limbs showed increased bone density at femurs and tibias with signs of periostitis: on STIR sequence MRI these bones presented areas of hyperintense signal. Bone biopsy revealed a thickened periosteum that was strongly adherent to the underlying tissue. Histopathologic study showed signs of chronic inflammation. Steroid treatment was started, leading to a prompt resolution of the clinical picture within few days.

Case 2 was a 6-years-old girl who developed, two weeks after an untreated febrile pharyngitis, daily attacks of severe pain at ankles with fever. Joint examination was normal. Throat swab was positive for GAS. In the following weeks, recurrent crisis of bone pain persisted with a severe weight loss. She was hospitalized and laboratory tests showed mild anemia, thrombocytosis and unusual dysproteinemia with hypoalbuminemia and high a1, a2 and g globulinemia. Inflammatory markers and antibodies against GAS were elevated (ASO 775 IU/ml, AND-B 1660 U/ml). STIR sequence MRI showed hyperintense areas at the femurs, tibias, humerus and ulnas, associated with a thickened pretibial soft tissue. Bone marrow biopsy showed signs of chronic inflammation. A short cycle of steroids was administered with rapid resolution of symptoms, turning off inflammatory markers. Immaging became normal after three months.

**Conclusion:** Our patients fulfill the GS features with evidence of previous GAS infection. Our patients lived in the same area of Northern Italy and presented the onset of GS a week apart. Our experience suggests that a timely diagnosis and a short cycle of steroid may rapidly change the history of GS.

**Disclosure of Interest**: None Declared

**Table 1 (abstract P381).** Main features of our patients with English literature review

	Case 1	Case 2	English literature (7 patients)
Demographic data: gender; age at onset (years)	F; 9	F; 6	M:F = 3:4; 8 (0.3-14)
Previous intercurrent episode	pharyngitis	pharyngitis	pharyngitis (4), scarlet fever (1), otitis (1)
Fever and bone pain at the limbs	+	+	all
Increased inflammatory markers	+	+	6 (86%)
Characteristic dysproteinemia (hypoalbuminemia with increased a1, a2 and g globulinemia)	+	+	all
Throat swab culture positive for GAS	+	+	ND
Presence of antibodies against GAS	+	+	1 (14%)
Radiographic periosteal reaction	+	+	all
Complete resolution (months)	1	3	24 (10-66)

*ND* not done

### P382 Diagnosis of acute rheumatic fever with the 2015 revision of Jones criteria

#### Roberto Pillon^1^, Denise Pires Marafon^2^, Lidia Meli^2^, Claudia Bracaglia^2^, Andrea Taddio^1,3^, Fabrizio De Benedetti^2^

##### ^1^University of Trieste, Trieste, Italy; ^2^Division of Rheumatology, Ospedale Pediatrico Bambino Gesù IRCCS, Roma, Italy; ^3^Institute for Maternal and Child Health - IRCCS “Burlo Garofolo”, Trieste, Italy

###### **Presenting author:** Roberto Pillon

**Introduction:** In 2015 the historic Jones criteria for the diagnosis of Acute Rheumatic Fever (ARF) were revised introducing two different sets of criteria for low-risk and for moderate/high-risk populations (according to ARF incidence). In Italy the exact ARF incidence is unknown but small regional or local reports suggest an incidence of 2-5/100.000 per year, suggesting that our population might be considered at moderate risk for ARF.

**Objectives:** To evaluate the performance of the revised Jones criteria in a retrospective population and to compare it with the performance of the previous version of Jones criteria.

**Methods:** We conducted a retrospective study on 288 patients with ARF (108 female; median age 8.5 years, IQR 7.1-10.3) diagnosed from 2001 to 2015 in a Pediatric Rheumatology Division by pediatric rheumatologists, discharged with an ICD 9 code consistent with ARF. We retrospectively applied the two sets (for low-risk and for moderate/high-risk) of the 2015 revised Jones criteria and the 1992 version of the Jones criteria.

**Results:** Of 288 patients, 253 (87.8%) met the 1992 version of the Jones criteria, 237 (82.3%) met the revised criteria for low-risk populations and 259 (89.9%) for moderate/high-risk populations. None of these differences was significant. Prevalence of major and minor criteria is shown in Table. With the exception of difference in arthritis, the 1992 version and the 2015 revised version did not show major differences. Of the 288 patients with a clinical diagnosis of ARF 29 did not meet any version of the Jones criteria. Patients in this group presented with isolated chorea or silent carditis without other manifestations.

Prevalence of the clinical characteristics and comparison among the 1992 version of Jones criteria and the 2015 revised Jones criteria (low risk and moderate-high risk populations):

**Table:** 

		**1992 version**	**2015 low risk**	**2015 mod-high risk**	1992 version vs 2015 low risk*	1992 version vs 2015 mod-high risk*
Total of patients		253	237	259	0.08	0.51
**MAJOR CRITERIA**	Arthritis	94 (37.2)	88 (37.1)	209 (75.3)	1.0	<0.0001
	Erythema marginatum	3 (1.2)	3 (1.3)	3 (1.2)	1.0	1.0
	Chorea	48 (19)	49 (17)	49 (17)	0.73	0.91
	Carditis	230 (90.9)	247 (85.8)	247 (85.8)	0.51	0.39
**MINOR CRITERIA**	ESR/CRP	218/236 (92.4)	208/259 (80.3)	226/259 (87.3)	0.0026	0.13
	Fever	140 (55.3)	157 (54.5)	196 (68.1)	0.65	0.0004
	Arthralgia	186/249 (74.7)	166/235 (70.6)	8/257 (3.1)	0.36	<0.0001
	Prolonged PR	38 (15)	34 (14.3)	35 (13.5)	0.90	0.70

Values are expressed in Number (percentage). *p value (Fisher Exact test)

**Conclusion:** The revised Jones criteria for low-risk populations are slightly more sensitive than the 1992 version of Jones criteria, while the revised Jones criteria for moderate/high populations are slightly less sensitive than the 1992 version. In this population, the revised criteria did not substantially modify the diagnosis of ARF. Approximately 10% of patients presented with isolated chorea or silent carditis.

**Bibliography:**

1. Gewitz M, et al. Revision of the Jones Criteria for the Diagnosis of Acute Rheumatic Fever in the Era of Doppler Echocardiography: A Scientific Statement From the American Heart Association. Circulation. 2015.

2. Breda L, et al. Population-Based Study of Incidence and Clinical Characteristics of Rheumatic Fever in Abruzzo, Central Italy, 2000-2009. The Journal of Pediatrics 2012

**Disclosure of Interest**: None Declared

### P383 Restrospective view of primary raynaud’s phenomenon in childhood

#### Enes Turan, Sara Sebnem Kilic

##### Pediatric Rheumatology, Uludag University Medical Faculty, Bursa, Turkey

###### **Presenting author:** Sara Sebnem Kilic

**Introduction:** Raynaud’s phenomenon (RP) refers to transient vasospasm of peripheral arteries and arterioles, usually involving peripheral small vessels of the fingers or toes and resulting in a triple-colour change starting with pallor and followed by cyanosis and erythema. Attacks are typically triggered by cold or emotional stress. The diagnosis of RP can be made on the basis of the patient’s clinical symptoms. Primary RP occurs without underlying disease and is considered a benign. Connective tissue diseases are the most common cause of secondary RP. **Objectives:** Detailed history, physical, and laboratory findings of the patients with primary RP were evaluated restrospectively.

**Methods:** Data collection was performed via the patient files. The study was approved by the Ethical Committee of the Uludag University Faculty of Medicine.

**Results:** Out of the 58 patients, 38 were girls whereas 20 were boys. The patients’ boy/girl ratio was 1.9 (38/20) and their ages ranged from 6 to 21 years (median:16 years). The median age at onset of complaints due to RP was 13 (minimum:2, maximum:17), median age at diagnosis:15 (minimum:3, maximum:17). In 37.9% cases (n = 22) There were similar complaints in first-degree relatives in 37.9% of patients. The most common symptom was feeling cold in the extremities ends (100%), followed by pain (87%) and tingling sensation (70.7%). While 38 patients(65.5%) had biphasic colour changes, 20 (34.5%) had triphasic colour changes. Triphasic colour change was statistically significant with high incidence (p = 0.026) in cases with ANA positivity and family history. ***Low titer positive*** ANA was present in 14 patients(24.1%). Presence of migraine in 29,3% of the cases was the most common disease associated with RP. Migraine was significantly more frequent in the girls compared to the boys (p = 0.02). Furthermore, cases with migraine had a significant lower hemoglobin levels and significant higher mean platelet volume. Apart from preventive measures, calcium channel blockers (CCB) were the most used drugs (n = 32, 55.2%) and were beneficial in 78.1% (25 out of 32). Nitroglycerin patch was applied to the rest of patients (n = 26, 44.8%), which was useful to relieve the symptoms in 50% of them. Sildenafil was used in one patient who was resistant to both of the drugs and the result was positive.

**Conclusion:** There is very limited data concerning childhood RP. In previous studies conducted in children there is no data on association between RP and migraine. Anemia in patients with RP was thought to be a predisposition to migraine development. Calcium channel blockers were found to be more effective than nitroglycerin patch treatment. Further studies with a greater number of patients are required in order to confirm our results.

**Disclosure of Interest**: None Declared

### P384 Prevention of Sjögren’s syndrome by immunosuppressants in children with positive anti-ro antibodies and chronic nonspecific complaints

#### Yasuhiko Itoh, Tomoko Shigemori, Shingo Yamanishi, Hidehiko Nagasaki

##### Department of Pediatrics, NIPPON MEDICAL SCHOOL, Tokyo, Japan

###### **Presenting author:** Yasuhiko Itoh

**Introduction:** Anti-Ro antibodies are found in children with either Sjögren’s syndrome (SS), systemic lupus erythematosus (SLE) or, occasionally, chronic nonspecific complaints such as fatigue and low-grade fever. We have reported that about 10% of children with chronic nonspecific complaints and with positive antinuclear antibodies were positive for anti-Ro antibodies.

**Objectives:** Although a few of them showed positive lip biopsy findings equivalent to that of SS (subclinical SS), the majority of such children exhibited no evidence of SS. To clarify the pathogenic role of anti-Ro antibodies in various conditions and the future development of dryness, those children have been followed for more than 10 years, both clinically and immunologically.

**Methods:** The patients included in this study were as follows: 1) 27 children with chronic nonspecific complaints, with anti-Ro antibodies, and with negative lip biopsies; 2) 10 children with chronic nonspecific complaints, with anti-Ro antibodies, and with positive biopsies; 3) nine children with SLE and with positive anti-Ro antibodies; 4) three children with MCTD and with positive anti-Ro antibodies. They have been followed for more than 10 years. Anti-Ro antibodies were measured by using either the Ouchterlony, ELISA, western immunoblot or, in some cases, RNA-immunoprecipitation methods.

**Results:** Twenty-three out of 27 patients in group 1 showed no evidence of SS even after more than 10 years. Most of the patients in group 2 who had not been treated by immunosuppressants have gradually developed dryness. On the other hand, patients who had been treated have developed no or little dryness. Patients in the groups 3 and 4 have developed no dryness.

All patients in groups 3 and 4 had been treated by immunosuppressants against their basic diseases and developed no dryness. In the same way, most patients with only nonspecific complaints treated with immunosuppressants did not develop dryness.

**Conclusion:** These results suggest that there may be a chance to prevent SS with immunosuppressants in children with positive anti-Ro antibodies as long as the treatment is initiated before they develop dryness.

**Disclosure of Interest**: None Declared

## Poster Session: Psycho-social aspects and rehabilitation

### P385 Invention of rehabilitative games using the leap motion controller for hand rehabilitation in children with juvenile idiopathic arthritis and investigation of its’ effectiveness

#### Ela Tarakci^1^, Nilay Arman^1^, Devrim Tarakci^1^, Yusuf S. Akgul^2^, Ozgur Kasapcopur^3^

##### ^1^Faculty of Health Sciences, Division of Physiotherapy and Rehabilitaiton, Istanbul University, Istanbul, Turkey; ^2^Department of Computer Engineering, Gebze Institute of Technology, Kocaeli, Turkey; ^3^Department of Pediatric Rheumatology, Medical Faculty of Cerrahpasa, Istanbul University, Istanbul, Turkey

###### **Presenting author:** Ela Tarakci

**Introduction:** Juvenile idiopathic arthritis (JIA) is the most common chronic pediatric rheumatic disease. Depending on the underlying pathologies, the common symptoms of this disorder are the limitations of the upper extremity joint movement angles, muscle imbalance and the functional limitations caused by the contracture due to these patients. By utilizing exciting new sensor technologies, such as Microsoft Kinect, Nintendo Wii and Leap Motion, practical game based rehabilitation applications have been becoming popular in the current.

**Objectives:** The aim of this study was to investigation of effectiveness of invented rehabilitative games using The Leap Motion Controller (LMC) for hand rehabilitation in patients with JIA.

**Methods:** 18 patients with JIA (14 girls, 4 boys), age range 8-18 participated in this study. Range of motion (ROM) of hand, grip strength, functional ability, fatique and quality of life were assessed with a goniometer, hand dynamometer, Childhood Health Assessment Questionnaire (CHAQ), Numeric Rating Scale (NRS), and the Pediatric Quality of Life Inventory (PedsQL), respectively. In order to improve hand functions, two games were invented with hand free LMC by our team. One of the games, “Leapball” was invented as picking the ball and throw to the basket. The game is to ensure the activities of repeated hold and release by simulating the hand grip function. The other game, “CatchAPet” is to intend touching the rabbits going out its burrow for simulating wrist flexion and extension. The patients completed a 8 week individually planned leap motion based exercise programme 3 times a week at the department of physical therapy and rehabilitation. Duration of the each treatment session was 30-45 minutes. Parameters of the games (Time, size of ball, speed of rabbits) were adjusted according to patient’s functional ability and progressed during the treatment.

**Results:** The mean age and duration of disease was 12.22 ± 3.30 (age range 8-18), 7.28 ± 4.22 years, respectively. The means of the pre/post treatment scores of NRS fatigue were 6.11 ± 1.53 / 2.22 ± 1.21, PedsQL-patients 63.71 ± 18.13 / 85.93 ± 12.08, PedsQL-parents 57.63 ± 18.88 / 80.80 ± 13.21, CHAQ-total 1.36 ± 0.67 / 0.30 ± 0.28, CHAQ-pain 31.94 ± 30.49 / 6.94 ± 11.77, and CHAQ-well being scores were 55.28 ± 19.28 / 21.94 ± 15.44, respectively. Table 2 shows ROM of wrist flexion and extension, fingertip to palm distance, hand grip and pinch grips scores for pre and post treatment. Significant statistical differences were found between pre and post-treatment all outcomes. (p < 0.001).

**Conclusion:** The study demonstrated that participating in a 8-week individually planned leap motion based exercise programme improves the range of motion of hand, grip strength, fatigue, physical function and the quality of life in patients with JIA. We think that improvements of the results may base on realistic animations of hand, virtual environments, patient motivation, consistent visual feedback and feasible and easy progression options of the invented games.

**Disclosure of Interest**: None Declared

**Table 2 (abstract P385).** ROM of wrist flexion and extension, fingertip to palm distance, hand grip and pinch grips scores for pre and post treatment

	Pre-treatment		Post-treatment		*p*	
	Mean ± SD		Mean ± SD			
	R	L	R	L	R	L
ROM-wrist flexion	58.72 ± 20.55	53.61 ± 23.56	80.28 ± 12.42	78.61 ± 14.32	0.000	0.000
ROM-Wrist extension	36.94 ± 20.51	34.17 ± 17.25	62.78 ± 10.74	62.50 ± 15.74	0.000	0.000
Fingertip to palm distance (cm)	1.05 ± 1.93	1.08 ± 1.78	0.58 ± 1.43	0.38 ± 0.84	0.001	0.009
Hand Grip (kg)	14.15 ± 9.99	9.04 ± 7.49	21.44 ± 11.10	18.08 ± 10.18	0.000	0.000
Lateral Pinch Grip (kg)	6.16 ± 3.08	5.14 ± 3.31	8.16 ± 3.72	6.87 ± 3.44	0.000	0.001
Tip Pinch Grip (kg)	2.14 ± 2.59	1.14 ± 1.19	3.58 ± 2.21	2.69 ± 1.77	0.002	0.001
Palmar Pinch Grip (kg)	3.18 ± 2.44	2.61 ± 2.97	5.83 ± 3.10	5.30 ± 2.82	0.000	0.000

### P386 Lupus and you: developing a workshop for young people with lupus and their families

#### Emily Wilson^1^, Hanna Lythgoe^1^, Eve Smith^1,2^, Jenny Preston^1,3^, Michael W. Beresford^1,2,3^

##### ^1^Department of Paediatric Rheumatology and Psychology, Alder Hey Children’s NHS Foundation Trust, Liverpool, UK; ^2^Department of Women’s and Children’s Health, Institute of Translational Medicine, Liverpool, UK; ^3^UK Experimental Arthritis Treatment Centre for Children, University of Liverpool, Liverpool, United Kingdom

###### **Presenting author:** Emily Wilson

**Introduction:** Lupus is a severe, autoimmune disease, potentially affecting any organ in the body. It is very rare in children and young people. As a regional service and the UK’s only ‘Centre of Excellence for Childhood Lupus’, our patients live across a large geographical catchment area. Many of the patients accessing our service report never having had the chance to meet another young person with the condition and how isolating this could feel. The mental health needs of young people with chronic health conditions are well known and potentially debilitating.

**Objectives:** We aimed to develop a series of day-long multidisciplinary “Lupus and You” workshops to be attended by young people with lupus and their families, within the Lupus Centre at Alder Hey Children’s Hospital. We wanted to continue to be responsive to the ongoing needs of our patients whilst also looking to intervene proactively to support them in developing resilience and coping strategies to minimise the impact of their condition on their lives.

**Methods:** 7 families participated in the first workshop which focused on two main themes; managing fatigue and research participation.

Fatigue was one of the most commonly reported concerns identified by young people and their parents on a routinely completed pre-clinic screening tool. A recent survey of young people with lupus suggested patients wanted to know more about research participation.

All participants completed a range of standardized measures to capture psychological well-being (including fatigue, emotional well-being, resilience and quality of life) at the time of attendance. Evaluations forms were given out to all attendees in order to review the day and identify areas for consideration within future workshops.

**Results:** Participants participated in exercises designed to facilitate sharing of difficulties as well as examples of strategies utilized to manage fatigue. The team’s psychologist, physiotherapist and occupational therapist also shared ideas and strategies for families to consider. Information about local and national research in paediatric lupus was shared in an interactive session.

High levels of fatigue were identified amongst lupus patients (mean score on the fatigue severity scale 5.1 (range 4 – 5.8) where people scoring above 3 points are considered to suffer with fatigue). Young people and their families reported increased confidence in managing fatigue when pre- and post-workshop data were compared.

Otherwise there was considerable variation in the extent to which young people were coping with having lupus. The results will be reviewed with the individuals and their parents in order to inform ongoing intervention and support.

Feedback from workshop evaluations indicated that attendees found the activities to be helpful and convenient to attend. Support for siblings, medication, and knowledge of common symptoms were identified as potential topics for future workshops.

**Conclusion:** The “Lupus and You” workshop allowed young people with lupus and their families to share experiences of managing this chronic illness, to develop supportive relationships whilst also developing resilience and practical strategies for living their lives fully despite their ongoing health needs.

Workshops such as this could be used as a forum to facilitate collection of important data from patients with rare diseases, whilst simultaneously benefitting patients and their families.

The workshops are being considered as part of a stepped-care model of psychological support offered to lupus patients.

**Disclosure of Interest**: None Declared

### P387 Development and usability testing of an ipad and desktop psycho-educational game for children with juvenile idiopathic arthritis and their parents

#### Lynn R. Spiegel^1,2^, Jennifer Stinson^3,4^, Mark Connelly^5^, Adam Huber^6^, Nadia Luca^7^, Argerie Tsimicalis^8^, Stephanie Luca^9^, Naweed Tajuddin^3^, Roberta Berard^10^, Julie Barsalou^11^, Sarah Campillo^12^, Brian Feldman^1^, Shirley Tse^1^, Paul Dancey^13^, Ciaran Duffy^14^, Nicole Johnson^7^, Patrick McGrath^15^, Natalie Shiff^16^, Lori Tucker^17^, Charles Victor^18^

##### ^1^Rheumatology, Hospital for Sick Children, Toronto, Canada; ^2^Pediatrics, University of Toronto, Toronto, Canada; ^3^Child Evaluative Health Sciences, The Hospital for Sick Children, Toronto, Canada; ^4^Lawrence S. Bloomberg Faculty of Medicine, University of Toronto, Toronto, Canada; ^5^Psychology, University of Kansas Medical Center, Lawrence, KS, United States; ^6^Rheumatology, IWK Health Center, Halifax, Canada; ^7^Rheumatology, Alberta children’s Hospital, Calgary, Canada; ^8^Ingram School of Nursing, Mcgill University, Montreal, Canada; ^9^Child Health Evaluative Sciences, Hospital for Sick Children, Toronto, Canada; ^10^Rheumatology, Children’s Hospital of Western Ontario, London, Canada; ^11^Rheumatology, CHU Ste-Justine, Montreal, Canada; ^12^Rheumatology, Montreal Children’s Hospital, Montreal, Canada; ^13^Pediatrics, Memorial University of Newfoundland, St John’s, Canada; ^14^Pediatrics, Children’s Hospital of Eastern Ontario, Ottawa, Canada; ^15^Research, Innovation and Knowledge Translation, IWK Health Center, Halifax, Canada; ^16^Pediatrics, University of Florida, Gainesville, FL, United States; ^17^Rheumatology, BC Children’s Hospital, Vancouver, Canada; ^18^Institute of Health Policy, Management and Evaluation, University of Toronto, Toronto, Canada

###### **Presenting author:** Lynn R. Spiegel

This abstract is not included here as it has already been published.

### P388 Icancope: user-centred design and development of a smartphone app to support self-management for youth with arthritis pain

#### Lynn R. Spiegel^1,2^, Chitra Lalloo^3,4^, Lauren Harris^4^, Joseph Cafazzo^5^, Lori Tucker^6^, Kristin Houghton^6^, Brian Feldman^1,7^, Nadia Luca^8^, Ronald Laxer^1,9^, Jennifer Stinson^3,4^

##### ^1^Pediatrics, University of Toronto, Toronto, Canada; ^2^Rheumatology, The Hospital for Sick Children, Toronto, Canada; ^3^Lawrence S. Bloomberg Faculty of Nursing, University of Toronto, Toronto, Canada; ^4^Child Health Evaluative Sciences, The Hospital for Sick Children, Toronto, Canada; ^5^Centre for Global eHealth Innovation, University Health Network, Toronto, Canada; ^6^Pediatrics, University of British Columbia, Vancouver, Canada; ^7^Rheumatology, Hospital for Sick Children, Toronto, Canada; ^8^Rheumatology, Alberta Children’s Hospital, Calgary, Canada; ^9^Rheumatology, The Hospital for Sick Children, Toronto, Canada

###### **Presenting author:** Lynn R. Spiegel

This abstract is not included here as it has already been published.

### P389 Use of the Xbox Kinect virtual gaming system to improve upper extremity functions and activity performance in patients with with juvenile idiopathic arthritis

#### Nilay Arman^1^, Ela Tarakci^1^, Ozgur Kasapcopur^2^

##### ^1^Faculty of Health Sciences, Division of Physiotherapy And Rehabilitation, Istanbul University, Istanbul, Turkey; ^2^Department of Pediatric Rheumatology, Medical Faculty of Cerrahpasa, Istanbul University, Istanbul, Turkey

###### **Presenting author:** Nilay Arman

**Introduction:** Juvenile idiopathic arthritis (JIA) is most common chronic rheumatic disease in childhood. The upper extremity involvement in JIA causes muscle imbalance, joint destruction, pain, stiffness and limitations on activities of daily living (ADL) in varying degrees. It has been reported that improvements of upper extremity functions were achieved by video-based games (VBG) in various disease groups.

**Objectives:** The aim of this preliminary study was to investigate effects of client-centered task-oriented activity training (TOAT) with VBG on upper extremity functions and activity performance in children with JIA.

**Methods:** 23 patients (19 girls, 4 boys) with JIA (7 oligoarticular, 16 polyarticular) have upper extremity involvements, participated in this study. Muscular strength was measured by using a portable digital handheld dynamometer. Also, hand grip and pinch strength evaluations was done by a dynamometer and pinchmeter. ADL was evaluated by Duruoz Hand Index (DHI) and Childhood Health Assessment Questionnaire (CHAQ). Activity performance was performed by Jebsen Hand Function Test (JHFT). Also, activity performance and satisfaction were measured by The Canadian Occupational Performance Measure (COPM). Five most important problems of activity performance were determined with the patient and his/her parents. Fatigue severity was measured by Numeric Rating Scale (NRS). We have created training protocol with Xbox 360 games. We prefered ‘Dance Central2’, one of the Xbox 360 games, for warming (macerana dancing, 10 minutes). Other games, ‘Fruit Ninja’, ‘Table Tennis’, ‘Boxing’, ‘Volleyball’, ‘Darts’ and ‘Bowling’ were selected appropriately for TOAT according gto 5 most important limited ADL of each patient that determined with COPM. The games were set as client-centered for 45-60 minutes by the physiotherapist. All the participants completed 8 weeks (3 times in a week) of client-centered TOAT with Xbox 360 Kinect™ games.

**Results:** The mean age and duration of disease was 12,26 ± 3,09 (age range 8-18), 6,78 ± 4,16 years, respectively. 20 of patients had bilateral involvement of upper extremity. Wilcoxon test showed significant statistically differences pre and post-treatment, in almost all the values, except some scores of subtasks of JHFT (p < 0.001). All muscles strength of upper extremities were statistically significant increased (p < 0.001). Table 3 shows the scores of functional ability, fatigue, grip and pinch strength and activity performance outcomes pre and post-treatment.

**Conclusion:** Our Kinect Xbox 360 protocol that inclued client-centered TOAT has showed improvements on upper extremity functions and activity performance in patients with JIA. We think that TOAT with VBG improves the activity performance and physical functions via being stimulative and interactive in order to provide feedback and to increase interest and motivation. Xbox Kinect virtual gaming system is more fun and provides motivation, and may be a preferable method of treatment for patients with JIA but further studies are needed to compare with the potential benefits of VBG and conventional therapy in patients with JIA.

**Disclosure of Interest**: None Declared

**Table 3 (abstract P389).** Scores of outcome measures pre and post-treatment

	Pre-treatment	Post-treatment	*p*		Pre-treatment		Post-treatment		*p*
					Mean ± SD		Mean ± SD		
	Mean ± SD	Mean ± SD			R	L	R	L	R/L
CHAQ-total	1.30 ± 0.66	0.30 ± 0.30	0.000	Hand Grip (kg)	15.68 ± 10.50	11.13 ± 9.50	23.19 ± 10.78	20.49 ± 10.40	0.000 / 0.000
DHI	20.35 ± 11.90	0 ± 0	0.000	Lateral Pinch Grip (kg)	6.19 ± 3.11	5.39 ± 3.16	8.37 ± 3.64	7.35 ± 3.30	0.000 / 0.000
COPM-performance	3.56 ± 1.63	8.64 ± 1.06	0.000	Tip Pinch Grip (kg)	2.14 ± 2.35	1.29 ± 1.29	3.72 ± 2.0	3.07 ± 1.81	0.003 / 0.000
COPM-satisfaction	1.50 ± 0.63	9.42 ± 0.77	0.000	Palmar Pinch Grip (kg)	3.35 ± 2.34	2.90 ± 2.94	6.43 ± 3.25	5.99 ± 3.16	0.000 / 0.000
NRS-fatigue	6.30 ± 1.57	2.35 ± 1.46	0.000						

### P390 The truth, the whole truth and nothing but the truth: methotrexate and compliance; the JIA patient perspective

#### Madeleine Rooney^1^, Roisin Campbell^2^, Catherine Wright^3^

##### ^1^Center Experimental Medicine, QUEENS UNIVERSITY BELFAST, Belfast, United Kingdom; ^2^Pharmacy, Belfast Hospital Trust, Belfast, United Kingdom; ^3^Arthritis Care, Belfast, United Kingdom

###### **Presenting author:** Roisin Campbell

**Introduction:** Methotrexate is the first-choice disease-modifying drug (DMARD) for the treatment of JIA. It is accepted as an effective drug that induces remission in more than 70% of patients. Serious adverse effects are infrequent and usually transient due to rigid monitoring guidelines. However the impact on the quality of a child’s and their family lives has yet to be adequately researched.

**Objectives:** The aim of this research was to investigate the JIA patient’s perspective and tolerance and compliance of oral or parental Methotrexate in accordance with BSPAR guidelines.

**Methods:** Children between 10-17 years of age with a diagnosis of JIA (all sub-types included) attending the Paediatric Rheumatology Day Ward were asked to complete an adapted MISS (Methotrexate Intolerance Severity Score) questionnaire during their routine medicines reconciliation and compliance interview, carried out by the paediatric rheumatology pharmacist. A total of 15 questionnaires were completed.

Arthritis Care, host activity weekends for children between 10 – 17 years of age with a diagnosis of JIA, during one such weekend they devoted a group session to discussing and completing the adapted MISS questionnaire. To date a total of 15 questionnaires have been completed in the two different settings and with two interviewees of differing provenance.

**Results:** Results

**Table:** 

Venue	MISS score range	MISS average score
Rheumatology Day Ward	0 – 8	3.6
Activity Weekend	4 - 32	19

The children were asked “choose one word to describe how you feel about taking Methotrexate”.

Children attending Rheumatology Day Ward examples – Fine, Ok, Necessary, Annoying.

Children at the Activity Weekend examples – Evil, Depressed, Stressed, Sore, Terrible, Hate.

**Conclusion:** The MISS questionnaire is a well -designed validated tool widely used by Paediatric Rheumatology teams to assess Methotrexate tolerance in JIA patients. Anticipatory nausea and behavioural effects of Methotrexate are not clinically evident during physical assessment but may be detected by the MISS questionnaire. However this research poses the question how much value can we place upon the results we receive if they are so profoundly affected by the environment in which they are completed and the person who is requesting the information.

The narrow score range and low average MISS score obtained when completed with the rheumatology pharmacist creates the impression that Methotrexate is a well-tolerated drug which young patients find at worst “annoying”. Is it safe to dismiss this as the Hawthorne effect? (or should we say young patients simply trying to please us?).

Yet the dramatically higher average score and broad score range obtained at the Activity weekend indicate that Methotrexate has a significant detrimental effect on young patients creating a greatly anticipated stressful event every week.

Arthritis Care, activity weekends create an environment where young patients are free to express themselves in their own language and allow their anger and emotions to be expressed away from reproachful and anxious parents.

These findings challenge the validity of this questionnaire if it is so dependent on the environment and the questioner. Our preliminary finding demonstrate the need for further research in this field and highlight the already underestimated degree of morbidity and non-compliance associated with MTX in children with rheumatic diseases.

**Disclosure of Interest**: None Declared

### P391 Fatigue in patients with juvenile idiopathic arthritis: relationship to perceived health, physical health, self-efficacy, and participation

#### Wineke Armbrust^1^, Otto Lelieveld^2^, Jolanda Tuinstra^3^, Nico Wulffraat^4^, Joyce Bos^2^, Jeanette Cappon^5^, Marion van Rossum^5^, Mariët Hagedoorn^6^

##### ^1^Beatrix Children’s Hospital, Department of Pediatric Rheumatology and immunology, Groningen, Netherlands; ^2^Center for Rehabilitation, Groningen, Netherlands; ^3^Department of Health Sciences, University of Groningen, University Medical Center Groningen, Groningen, Netherlands; ^4^Department of Pediatric Immunology, University Medical Center Utrecht, Wilhelmina Children’s Hospital, Utrecht, Netherlands; ^5^location: Dr. Jan van Breemenstraat, Reade, Center for Rehabilitation and Rheumatology, Amsterdam, Netherlands; ^6^Department of Health Sciences, University of Groningen, University Medical Center Groningen, Groningen, Netherlands

###### **Presenting author:** Wineke Armbrust

**Introduction:** Fatigue is present in 60-75% of patients with JIA. Fatigue is multidimensional, meaning that it can be physical or mental in its manifestation and cause. The causes and consequences of fatigue are not well known.

**Objectives:** To assess the presence and severity of fatigue in patients with JIA, including factors presumed associated with fatigue (e.g., disease activity, disability, pain, physical activity, exercise capacity, and self-efficacy), and whether fatigue is related to participation in physical education classes, school attendance, and sports frequency.

**Methods:** Eighty patients with JIA (age 8-13) were included. Primary outcome measurements were fatigue (multi dimensional Pediatric-Quality-of-Life-Inventory (PedsQl)-Fatigue-scale) and energy level (uni dimensional VAS scale (0-10; 0 meaning respectively lowest an highest energy)). Predictors of fatigue were disease activity (PGAS (0-10 cm)), disability(CHAQ), physical activity (accelerometer), exercise capacity (Bruce test) and self-efficacy (Childhood Arthritis Self-Efficacy Scale). Consequences of fatigue were school attendance and participation to physical education class and sports using a questionnaire.

**Results:** Median age was 9.8 years [8.7; 11.0], disease duration was 2.95 years [1.22; 6.19], disease activity was .03 [.00;.90] and 60 patients were on medication. Sixty percent of patients with JIA suffer from daily low-energy levels; 27% suffer from very low-energy levels more than half the week. Fatigue measured with the PEDsQL was higher compared to the norm-population (77.5 compared to 80.5). High fatigue was correlated with low energy levels (P < .01). Patients who reported higher levels of fatigue showed higher disability (P < .01), higher pain (p < .01), lower exercise capacity p = .05), lower physical activity (<.01), and lower self-efficacy scores (P < .01). Disability (p = .01) and low self-efficacy (p = .04) were main predictors of fatigue in a multivariate model. Self-efficacy was a predictor of fatigue but did not act as moderator. Low energy levels were correlated with high disability (p < .01), pain (p = .01), a lower PAL (p < .01), and being on medication (p = .03). Low energy levels were best predicted by disability (p < .01)and low physical activity (p < .01) in a multidimensional model. Fatigue was a predictor for sports frequency (p = .01) but not for school attendance (p = .08).

**Conclusion:** Fatigue is present in more than half of the patients with JIA. Uni- and multidimensional measurements can be used to determine the level of fatigue. However, related factors were different for both measurements as single correlations as well as in a prediction model, indicating that they are not interchangeable. Interventions aimed at reducing perceived disability, stimulating physical activity, and enhancing self-efficacy might reduce fatigue and thereby enhance participation.

**Trial registration identifying number:** R@W Trial number ISRCTN92733069

**Disclosure of Interest**: None Declared

**Table 4 (abstract P391).** Patient characteristics and outcome measurements of 80 (52 female) patients

Characteristic	M *N*	[IQR] / (%)
Fatigue	77.8	[68.1; 86.1]
Daily energy level (cm)	6.00	[4.92; 7.49]
Exercise capacity (seconds)	592	[489; 638]
Physical activity level	1.53	[1.46; 1.60]
Full participation physical education class	*53*	(66)
Full school attendance	*56*	(70)
Sports frequency/week	1	[.25; 3.00]
Self-efficacy symptom	3.75	[2.50; 6.72]
Self-efficacy Activity	6.88	[4.38; 9.38]

*M* Median, *IQR* Inter Quartile Range, *N* Number, *yr* years, *cm* centimeters

### P392 Nurses’ experiences of meeting children afraid of needlestick

#### Anna Vermé^1^, Ylva Lampela^2^

##### ^1^Pediatric Rheumathology, Karolinska, Sweden; ^2^Youth clinic, Södersjukhuset, Stockholm, Sweden

###### **Presenting author:** Anna Vermé

**Introduction:** Regardless where nurses work they meet both adults and children who are scared of needle stick. Both the empirical and the literature reveals that the meeting with the children who are afraid of needle stick can be challenging and difficult to manage. According to competence description to pediatric nurses work to ensure that blood tests, examinations and treatments are tailored to the individual child. When meeting with children who are afraid of needle stick and meeting their families, it is important that the nurse takes into account several factors: the child’s age, developmental stage, previous experience and the situation the child is in. The parents’ past experiences and feelings about needle stick can affect how the child reacts to different procedures.

**Objectives:** To describe nurses’ experiences of meeting children scared of needle stick and meeting their families.

**Methods:** The study was qualitative and the collected material was analyzed using qualitative content analysis. Five nurses who worked in child care 3-42 years participated in the study.

**Results:** The results were reported as three categories and eleven subcategories. The categories were: Challenging encounters with children who are afraid of needle stick, Cooperation and Child different reactions. The meeting was affected by various things such as the nurse’s own experience of meeting children who are afraid of needle stick, the circumstances and the nurse’s ability to build trust and confidence. A working relationship with colleagues, children and parents was crucial so the situation would be as good as possible.

**Conclusion:** Fear of needle stick is a common phenomenon, especially in pediatrics. Sharing experiences and gaining knowledge on this subject make it easier for nurses who feel insecure about meeting children who are afraid of needle stick and their families. We also hope that this work will lead to discussions on this topic in the working groups who in their daily work meet children who are afraid of needle stick.

**Disclosure of Interest**: None Declared

### P393 The first transition clinic from pediatric to adult rheumatology in Turkey

#### Ayse Huri Ozdogan^1^, S Ugurlu^1^, K Barut^2^, A Androvic^2^, O Kasapçopu^3^

##### ^1^Department of Adult Rheumatology, Cerrahpasa Medical Faculty, İstanbul, Turkey; ^2^Department of Pediatric Rheumatology, Cerrahpasa Medical Faculty, İstanbul, Turkey; ^3^Department of Pediatric Rheumatology, Cerrahpasa Medical Faculty, İstanbul, Turkey

###### **Presenting author:** Ayse Huri Ozdogan

**Introduction:** -

**Objectives:** To assess the outcome of the first transition clinic from pediatric to adult rheumatology in İstanbul, Turkey

**Methods:** A transition outpatient clinic has been started in 2014 in Cerrahpaşa Medical Faculty with the contribution of 2 adult and 4 pediatric rheumatologists who get together once every 2 to 3 months. Patients who have been followed in the pediatric rheumatology clinic were asked to attend the transition OPC after the age of 18. These patients are seen by all the attending physicians initially in the pediatric clinic. A standardized form is filled for all which includes information on diseases history, last physical and laboratory examinations, and treatment. The next appointment is given for the adult rheumatology clinic where they are seen by one of the adult rheumatologist who have attended the first visit. All patients seen in the transition clinic during this 2 year period were contacted to assess the follow-up and outcome.

**Results:** Since 2014, 82 patients (52 F, 30 M) were seen in the transition OPC. The mean age was 21 years (18-24). The diagnoses of the patients were FMF in 44, JIA in 28, BD in 3, SLE in 2, vasculitis 2 and others 3. Fifty six patients were regularly attending the adult rheumatology clinic (68%), whereas 3 were being followed in another center, 9 never attended to a visit in the adult clinic and 14 were lost to follow up. The attendance rate was somewhat higher in girls compared to boys (71% vs 60%). Their main complaints were the crowdness of the adult clinics (8 patients) and insufficient attention (7 patients).

**Conclusion:** These are the preliminary results of the first transition clinic from pediaitric to adult rheumatology from Turkey. Compared to well established clinics from other countries like Canada, the attendance rate and the age at transition are comparable. In order to increase the attendance and the overall satisfaction of the patients the programme needs to be improved.

**Disclosure of Interest**: None Declared

### P394 The use of a screening tool to support children and young people with lupus and their families in prioritising issues for discussion in clinic appointments

#### Emily Wilson^1^, Jody Etheridge^1^, Eve Smith^1,2^, Katie Dobson^1^, Sue Kemp^1^, Michael W. Beresford^1,2^

##### ^1^Department of Paediatric Rheumatology and Psychology, Alder Hey Children’s NHS Foundation Trust, Liverpool, UK; ^2^Department of Women’s and Children’s Health, Institute of Translational Medicine, University of Liverpool, Liverpool, United Kingdom

###### **Presenting author:** Emily Wilson

**Introduction:** Patients attending the UK’s only ‘Centre of Excellence for Childhood Lupus’ at Alder Hey Children’s Hospital, Liverpool, routinely attend clinic appointments every three months. During these appointments there is pressure for clinicians to review complex medical presentations and treatment in a timely manner. As a service we recognise the importance of holistic, multi-disciplinary care and the need to consider the priorities of patients and their families in relation to the illness and its management.

The challenges faced by patients in asking questions and asserting influence over a medial consultation are widely acknowledged and so a ‘screening tool’ was developed to facilitate this process.

**Objectives:** To develop a screening tool to facilitate conversations with young people with lupus and their families in order to identify areas of concern that they would like to discuss within or outside of a given clinic appointment.

**Methods:** Immediately prior to each clinic appointment, patients with juvenile-onset systemic lupus erythematosus (‘lupus’) and the parent or guardian accompanying them were asked to complete a bespoke screening questionnaire.

The tool was developed through detailed consultation with patients, parents and multi-disciplinary team members (nursing, occupational therapy, physiotherapy and psychology). Several versions were developed to enable the tool to be utilised by children of different ages as well as their parents. Parents of all children were encouraged to identify issues that concerned them about their child but also areas in which they felt they were struggling themselves regarding their child’s condition.

The completed tools were brought into the consultation for consideration by the attending clinicians and team. Where appropriate, the screening questionnaires influenced the topics of conversation within the appointment, otherwise, plans were made to contact the multi-disciplinary team separately.

The completed questionnaires formed part of the patients’ case notes. Data were also collated and used to inform wider service development within the lupus service.

**Results:** Completed screening tools were reviewed over a 10-month period. 36 were completed (19 young people, 17 parents).

Amongst the young people, the most frequently reported concerns were: fatigue (81%), appearance (58%), pain (53%) and exercise (53%). Parents were most concerned for their children regarding: fatigue (65%), pain (65%), emotions (59%), sleep (59%) and support from school or college (53%). In terms of areas in which parents were concerned about themselves, most frequently reported were: concerns regarding the future (82%), feeling upset regarding their child’s condition (59%), supporting their child (59%) and having adequate support from others (59%).

**Conclusion:** The questionnaires have received positive feedback from patients, parents and staff in terms of their ease of completion and their utility in terms of highlighting issues for further discussion either within clinic or otherwise. They have also helped especially to normalise the role of psychological well-being in the management of lupus.

The data have been used to influence service development including:

- Development of a ‘health passport’ document to facilitate communication between family, hospital and school
- Development of a workshop for patients with lupus and their families focussing on fatigue management
- Development of a future workshop for patients and their families focussing on transition, the future and becoming a young adult with lupus.

**Disclosure of Interest**: None Declared

### P395 “On the journey diagnosed with juvenile idiopathic arthritis (JIA)”– a bag with relevant information that generates trust

#### AnnaCarin Horne, Karin Palmblad, Malin Höglund

##### Pediatric rheumathology, Karolinska Hospital, Stockholm, Sweden

###### **Presenting author:** Malin Höglund

**Introduction:** When a child is diagnosed with juvenile idiopathic arthritis (JIA) a lot of questions evolve among family members and the social surrounding. The initial information provided by the doctor may be overwhelming and it may be hard to take in all the details. Many seek additional information from literature or from the internet but feel overwhelmed by all the different information available.

**Objectives:** To provide parents and children with correct information included in an information bag and to evaluate the response on this initiative.

**Methods:** “On the journey diagnosed with JIA” is a information bag created in collaboration between the pediatric rheumatology unit at Astrid Lindgren Children’s Hospital, the Swedish Rheumatism Association, Young Rheumatic Patients, and AbbVie Sweden. The information bag contains informative brochures, lists of adequate web sites with balanced information, and information from parent and patients associations. In addition, a calendar is provided to encourage self-management of the disease. All information is carefully checked by the clinic and based on medically correct information. The bag is a compliment to the information that the family receives at the clinic; a way for the patient and family to continue the journey at their own pace back home. The pilot project consisted of 20 bags that were handed out at the pediatric rheumatology clinic at Astrid Lindgren Children’s Hospital to children newly diagnosed with JIA. The children were between 2-17 years old. After a month an assistant nurse at the clinic contacted the family by mail or by phone to evaluate how the bag had been used. The families were asked whether the information added value and if they found answers to any questions that arose after the doctor’s visit.

**Results:** All of the 20 families were positive to the bag and its content. They found that the information helped them to explain about JIA to people around them, in school or preschool, or on activities after schools. The content of the bag had increased understanding and knowledge of what the disease means for the child in everyday life. The material had also helped the parents to get a good basic information so that they then had a better perspective on all the information online and in social media.

**Conclusion:** The information bag has been an appreciated complement to the information given at the hospital and will now be implemented as a tool provided to children diagnosed with JIA at Astrid Lindgren Children’s Hospital.

**Disclosure of Interest**: A. Horne: None Declared, K. Palmblad: None Declared, M. Höglund Grant / Research Support from: Travel grant from AbbVie

### P396 Psychological peculiarities in patients with cryopyrin-associated periodic syndromes and systemic juvenile idiopathic arthritis

#### Natalia Stepanenko, Svetlana Salugina, Evgeny Fedorov, Irina Nikishina, Maria Kaleda

##### Nasonova Research Institute of Rheumatology, Moscow, Russian Federation

###### **Presenting author:** Natalia Stepanenko

**Introduction:** Cryopyrin-associated periodic syndromes (CAPS) and systemic juvenile idiopathic arthritis (sJIA) formally belong to the same group of autoinflammatory diseases (AID) and have a number of clinical similarities, and approaches to therapy. At the same time CAPS (cryopyrin-associated periodic syndromes) refer to AID group, associated with NLRP3 gene mutation with multiorgan damage and involvement of the central nervous system (CNS) that can result in the development of cognitive functions and determine certain psychological peculiarities.

**Objectives:** to carry out a fundamental psychological testing aimed at identifying peculiarities of the emotional sphere and cognitive deficits in patients with CAPS and systemic juvenile idiopathic arthritis (sJIA).

**Methods:** A detailed psychological examination was performed under a single diagnostic plan: 12 patients consecutively admitted to the clinic: 6 patients with CAPS (4 - MWS, 2 - CINCA-NOMID), aged from 9 to 17 years, 4 males, 2 females; average disease duration of 9 years and 6 patients with sJIA aged from 7 to 16 years, 4 males, 2 females; average disease duration of 7 years.

**Methodology:** clinical interview, 8-Luscher color test, Spielberger - Khanin test to identify the level of anxiety, CMAS, methods of pathodiagnostic survey.

**Results:** CAPS patients showed a reduction of attention functions in a part of concentration and distribution (by 66.7%), insufficient attention efficacy (increase in the time consumed in vigorous activities) in 83.3%, the low-speed entry (50%), reduced level of performance efficiency in 83.3%. Irregularities in the process of memorization, short-term memory (16%) and storage of data (long-term memory, LTM) were observed in a half. The concreteness of ideation which did not meet the age norm and reduced level of factuality were observed (50%).

An increased level of personal anxiety (66.7%), dissatisfaction with their appearance (50%), difficulty in communicating with agemates (50%), reduced level of social adaptation were observed in the emotional sphere (7%). Patients with sJIA showed the reduction of focus functions - in a part of concentration (50%), the distribution of attention (16.7%), insufficient attention efficacy (33.3%), and low speed entry (16.7%). Abnormalities in the short-term memory link were observed in 33.3%, long-term – in 16.7%. The thinking processes did not show reducing level of factuality in 33.3% and specificity of ideation inconsistent with age norm. In the emotional sphere there were revealed an increased level of personal anxiety (50%), difficulty in communicating with agemates (83.3%), dissatisfaction with appearance and reduced level of social adaptation - in a half.

**Conclusion:** Differences in the psychological status of patients with CAPS and sJIA were revealed. CAPS patients showed the most dramatic abnormalities in the cognitive functioning - in all functions of attention, memory and thinking, which may be indicative of the major organ damage. Disturbances in the emotional sphere are dominated in patients with sJIA (difficulty in communicating with agemates and low social adaptation). Dissatisfaction with their own appearance is equally characteristic of both groups. Clinical and psychological status of patients with CAPS and sJIA requires further study at a greater volume of material to identify sustainable patterns of regularities, assess the impact of targeted therapies and develop psychocorrective programs for cognitive and emotional disorders.

**Disclosure of Interest**: None Declared

### P397 The perceptions of patients with juvenile idiopathic arthritis about “having a rheumatic disease” and “doing exercise” via metaphors

#### Nilay Arman^1^, Ela Tarakci^1^, Kenan Barut^2^, Amra Adrovic^2^, Sezgin Sahin^2^, Ozgur Kasapcopur^2^

##### ^1^Faculty of Health Sciences, Division of Physiotherapy and Rehabilitaiton, Medical Faculty of Cerrahpasa, Istanbul University, Istanbul, Turkey; ^2^Department of Pediatric Rheumatology, Medical Faculty of Cerrahpasa, Istanbul University, Istanbul, Turkey

###### **Presenting author:** Nilay Arman

**Introduction:** A metaphor is considered the strongest device for an individual to comprehend and explain a hypothetical or an abstract, complex fact in a high level. Metaphors shape our perceptions, beliefs, attitudes and thoughts and they may influence the actions we take in the real world. Metaphors are among the most powerful cognitive tools to understand children with chronic disease. Juvenile idiopathic arthritis (JIA) affects social, emotional, and cognitive development of children, because it is a chronic disease in childhood. Understanding the perceptions about their disease helps to accomplish cognitive barriers of the rehabilitation in children. However, in the literature, no study has been found about perceptions about their disease in children with arthritis.

**Objectives:** The aim of the study was to understand the perceptions of patients with JIA about having a rheumatic disease and doing exercise via metaphors.

**Methods:** The population of the study included 20 (16 girls and 4 boys) patients with JIA. Each patient with JIA was asked to complete the blanks in the sentences, “Having a rheumatic disease is like a/an.....................because..................” and “Doing exercise is like a/an.....................because..................” The data were analysed using qualitative (content analysis) method.

**Results:** The mean age of the patients was 11.35 ± 2.20 (age range 8-18). According to the findings of the study, patients with JIA identified 24 metaphors (15 metaphors for “having a rheumatic disease”, 9 metaphors for “doing exercise”) in total. The metaphors for “having a rheumatic disease” developed by patients with JIA were analysed and interpreted at two categories: animals and objects. While the animal metaphors indicated three characteristics as “slow-moving”, “coping difficult” and “frightening” for “having a rheumatic disease”. The object metaphors indicated five characteristics as “Chaotic”,“ Stiffness”, “Formidable”, “Puffy” and “Uncertainty” for it. The metaphors for “doing exercise” indicated three characteristics as “Beneficial for Health”, “Tedious” and “Hard”.

**Conclusion:** As a result, the metaphors can be used as a strong research tool in understanding, revealing and explaining the cognitive images of patients with JIA about their disease and doing exercise. This study showed that patients with JIA prefered different metaphors for explaining their perceptions of disease and exercise but the reasons were similar. According to their metaphors, “Having a rheumatic disease” is similar to solving a difficult problem, “Doing exercise” is necessary for getting better but it requires the responsibility.

**Disclosure of Interest**: None Declared

### P398 The effects of kinect based dance therapy on lower extremity functions of a patient with oligo articular jia: case report

#### Nilay Arman^1^, Ela Tarakci^1^, Ozgur Kasapcopur^2^

##### ^1^Faculty of Health Sciences, Division of Physiotherapy and Rehabilitaiton, Istanbul University, Istanbul, Turkey; ^2^Department of Pediatric Rheumatology, Medical Faculty of Cerrahpasa, Istanbul University, Istanbul, Turkey

###### **Presenting author:** Nilay Arman

**Introduction:** Juvenile idiopathic arthritis (JIA) is characterized by joint pain, swelling and a limitation of movement caused by inflammation. Subsequent joint damage can lead to disability and activity restriction. In the literature, some evidence reports that patients with JIA are physically less active when compared with healthy children. Active video gaming such as Xbox 360 Kinect is a promising physical activity alternative given that children are encouraged to move whilst engaging in an activity they enjoy.

**Objectives:** The purpose of this case report is to describe effects of Kinect based dance therapy (KBDT) on lower extremity functions of a patient with oligo articular JIA.

**Methods:** The patient was 18-year-old girl with oligo articular JIA. She had left hip arthritis. So, she had dissatisfaction about her appearance during the walking because of limitation of hip range of motion and increased anterior pelvic tilt. Also, she complained of pain and fatigue at rest and during the walking. Pain during rest and activity was assessed using a 0-to-10 Numeric Rating Scale (NRS). Fatigue severity was also measured by NRS with higher scores indicating high fatigue, Active and passive Range of motion (ROM) of lower extremity was measured using a standard goniometer. Muscular strength was estimated at maximal isometric force for the muscles of the lower extremities by using a portable digital handheld dynamometer. Static balance was measured by using the Functional Reach Test (FRT) and Lateral Reach Test (LRT). Functional ability was assessed by Childhood Health Assessment Questionnaire (CHAQ). Functional capacity was assessed by 6 minutes walking test (6MWT). The total distance was recorded during walking for 6 minutes. Our KBDT program was composed of “Dance Central 2”. Dance Central 2 is a rhythm game developed exclusively for the Xbox 360 Kinect. During the game, players perform given dance moves similar to a mirror, which are tracked by the Kinect. The more accurately the player performs the move, the more points he/she scores. Higher difficulties increase move complexity. 4-5 different dances that included weight bearing and balance actions for lower extremity were selected for our KBDT program. She completed 8 weeks (3 times in a week) of her specific KBDT program.

**Results:** Pain scores of NRS-rest and NRS-activity and NRS-fatigue were 5 point before the treatment. After treatment NRS-activity was 1 point, both NRS-rest and NRS-fatigue were 0 point, Active ROM of Hip flexion was 70, hip extension 20, abduction 20, internal rotation 30, and external rotation was 30 degrees before the treatment. After the treatment, Active ROM hip flexion was 90, hip extension 35, abduction 45, internal rotation 45, and external rotation was 40 degrees. FRT scores of pre and post-treatment were 36 and 45 cm respectively. LRT scores of pre and post-treatment were 22 and 30.5 cm respectively. Pre and post-treatment scores of 6MWT were 406 and 522 meters, respectively. CHAQ total, CHAQ-walking, CHAQ- activities scores were 1.12, 2 and 2 before the treatment respectively. All CHAQ scores dropped to 0 after the treatment. Also, all muscle strength scores of both lower extremities were improved after the treatment.

**Conclusion:** In this study, we found clinically significant improvements of lower extremity functions, functional capacity, pain and fatigue for a patient with oligo articular JIA. This indicates that repeated actions with visual and auditory feedback provided by Kinect-based virtual reality may increase patient focus and motivation and thereby improving the quality of movements. This case report describes a novel approach to rehabilitation of patients with JIA but further investigation into the effectiveness of KBDT for children with JIA is warranted.

**Disclosure of Interest**: None Declared

### P399 AJIados, a francophone patient association for adolescents and young adults with JIA

#### Laurence Toumoulin^1^, Johnny Frossard^1^, Stephanie Archimbaut^1^, Anne Paitier^1^, Rolande Guastalli^1^, Severine Guillaume Czitrom^2^

##### ^1^AJIADOS, Le Kremlin Bicetre Cedex, France; ^2^Service de Medecine des Adolescents, CHU DE BICETRE, Le Kremlin Bicetre Cedex, France

###### **Presenting author:** Severine Guillaume Czitrom

**Introduction:** Adolescent departments in France are usually devoted to the care of mental health disorders. Yet, there is a huge need for chronic organ-diseases care, focused on adolescents specifically. Adolescence is often a difficult period but chronic organ-disease as JIA, may amplify the troubles, thus requiring a particular effort.

**Objectives:** Improve the daily life of adolescents and young adults with JIA with practical actions.

**Methods:** We chose to start with the improvement of patient education in adolescents and young adults having juvenile idiopathic arthritis in France. We created the first patient association “AJIados” involved in adolescent/ young adults in France.

**Results:** AJIados supports different actions : (i) collective meetings between the young people, between their parents/families, between them and caregivers. We organize 4 times a year, collective assemblies on various themes, generally chosen by the patients; all together, we create small documents to help solving practical problems (school, physiotherapy, living with a uveitis…) and distribute them across our country as well as in the Maghreb. We also send to interested people our newsletter every 3 months. Our efforts have had a great success with always more adolescents and families; (ii) we organized nice encounters with key figures in french sport and music, who are vectors of hope and will go on this way; (iii) we created a web-site (www.ajiado.org) to help patients in their daily life: there are 5 modules (a forum, a tool box, news on research as well as surveys, an agenda with referral caregivers that is built and improved by the patients themselves and a growing part devoted to patient education).

**Conclusion:** Presently, the success is « au rendez-vous » with the adolescents and young adults, justifying to pursue all our efforts.

**Disclosure of Interest**: None Declared

### P400 Health-related quality of life in Thai children with juvenile idiopathic arthritis

#### Sirirat Charuvanij, Chollada Chaiyadech

##### Department of Pediatrics, Siriraj Hospital, Mahidol University, Bangkok, Thailand

###### **Presenting author:** Sirirat Charuvanij

**Introduction:** Juvenile idiopathic arthritis (JIA) is a chronic disease resulting in pain and disability. There has been no study focusing on health-related quality of life (HRQOL) among Thai children with JIA.

**Objectives:** To describe HRQOL and determine factors influencing the HRQOL of Thai JIA patients.

**Methods:** A cross-sectional descriptive study was conducted in JIA patients and their parents followed up at a pediatric rheumatology clinic, Siriraj Hospital, Bangkok, Thailand. HRQOL was measured by using the Pediatrics Quality of Life (PedsQL) 4.0 generic core scale, Thai version, during January 2015-December 2015. Suboptimal HRQOL is defined as a PedsQL total mean score of < 78.6.^1^

**Results:** Sixty-five patients and their parents were included. Thirty-three patients were female (50.8%). Mean age of patients was 9.6 ± 3.9 years and the median of duration of the disease was 1.1 (0.0-9.43) years. Systemic JIA was the most common JIA subtype (40%). The median of Juvenile Arthritis Disease Activity Score-71 (JADAS-71) was 7 (0-27.2). Forty-nine (75.4%) patients were having active disease. The median of total PedsQL score reported by patients was 80.6 (36.9, 100); physical functioning score 78.1 (34.4, 100), emotional functioning score 85 (35, 100), social functioning score 90 (30, 100), school functioning score 80 (25, 100). There were 25 (45.4%) patients classified as having suboptimal HRQOL. The median of total PedsQL scores reported by parents was 71.7 (33.3, 100); physical functioning 75.0 (0, 100), emotional functioning 80 (40, 100), social functioning 85 (25, 100) and school functioning 65 (25, 100). Suboptimal HRQOL was identified in 39 (60%) patients based on parent reports. There was positive correlation between total PedsQL scores reported by children and their parents (r_s_ = 0.662). There were no statistically significant factors associated with suboptimal HRQOL reported by children. Non- oligoarthritis; OR 3.8 (95%CI: 1.0, 14.6), joint pain; OR 4.1 (95%CI: 1.4, 11.5), limping; OR 4.7 (95%CI: 1.2, 18.7), wrist arthritis; OR 8.6 (95%CI: 1.0, 72.1), elevated erythrocyte sedimentation rate: OR 2.8 (95%CI: 1.0, 7.9), elevated C- reactive protein: OR 3.2 (95%CI: 1.1, 9.2), active disease; OR 4.9 (95%CI: 1.2, 16.8), high disease activity (JADAS-71 ≥ 10.5); OR 12.6 (95%CI: 2.6, 60.9) and oral steroid use; OR 3.2 (95%CI: 1.0, 10.3) were associated with suboptimal HRQOL reported by parents. However, only high disease activity influenced on the suboptimal HRQOL by multiple logistic regression analysis; adjusted OR 20.2 (95%CI: 1.4, 291.7).

**Conclusion:** Almost half of Thai JIA patients had suboptimal HRQOL. The physical functioning score was the lowest aspect. The factor associated with suboptimal HRQOL reported by parents was high disease activity.

**Trial registration identifying number:** N/A

**Disclosure of Interest**: None Declared

### P401 Attitude surveys of pediatric and non-pediatric rheumatologists regarding transition of care

#### Takako Miyamae, Hisashi Yamanaka

##### Institute of Rheumatology, Tokyo Women’s Medical University, Tokyo, Japan

###### **Presenting author:** Takako Miyamae

**Introduction:** The goal of planned adolescent healthcare transition procedures is to optimize the functioning and well-being of all young people, including those with special healthcare needs. In this regard, the transitioning of young people with childhood-onset rheumatic diseases to adult healthcare is increasingly important, as many of these patients might continue to have active disease or considerable sequelae well into their adult lives.

**Objectives:** Attitude surveys of pediatric and non-pediatric rheumatologists about transition procedures were performed.

**Methods:** Overall, 28 operation commissioners of the Pediatric Rheumatology Association of Japan and 37 non-pediatric rheumatologists belonging to the Institute of Rheumatology, Tokyo Women’s Medical University participated in the surveys. Experiences of adult patients with childhood-onset rheumatic diseases, ideal medical care for adults, and factors contributing to challenges associated with the transition to adult care were examined.

**Results:** In total, 19% of pediatric rheumatologists still see their patients as adults, whereas 62% of non-pediatric rheumatologist have experiences with childhood-onset patients. Transition to non-pediatric medical care was supported by 81% and 95% of pediatric and non-pediatric rheumatologists, respectively. A preparatory step of visiting both a pediatrician and a non-pediatrician is recommended by some physicians. Two major factors, incomplete facilitation of personal independence to make the transition to adult care and the sharing of pediatric rheumatology knowledge and skills with non-pediatric rheumatologists, particularly with respect to autoinflammatory disorders, were identified as contributing to challenges associated with successful transition. Overall, 33 of 37 (89%) non-pediatric rheumatologists required education about childhood-onset rheumatic diseases.

**Conclusion:** Key elements for effective transitions include the development of education programs to help pediatric patients manage their illness independently and to share pediatric rheumatology knowledge within the non-pediatric rheumatology community.

**Disclosure of Interest**: None Declared

## Poster Session: New diseases

### P402 Interstitial lung disease with TMEM173 mutations is associated with a strong IFN-stimulated protein induction in situ

#### Cecile Picard^1,2^, Guillaume Thouvenin^3^, Caroline Kannengiesser^4^, Jean-Christophe Dubus^5^, Nadia Jeremiah^6^, Frédéric Rieux-Laucat^6^, Bruno Crestani^7^, Véronique Secq^8^, Christelle Ménard^9^, Martine Reynaud-Gaubert^10^, Françoise Thivolet-Bejui^1^, Philippe Reix^11^, Alexandre Belot^2,12^

##### ^1^Pathology, Hospices civils de Lyon, Hôpital Femme-Mère-Enfant, Lyon, France; ^2^Inserm U1111, Lyon, France; ^3^Pediatric Pulmonology, Assistance publique-Hôpitaux de Paris, Paris, France; ^4^Genetic, Assistance publique-Hôpitaux de Paris, Paris, France; ^5^Assistance publique-Hôpitaux de Marseille, Marseille, France; ^6^Institut Imagine, hôpital Necker, Paris, France; ^7^INSERM, Unité 1152, Université de Paris Diderot, Paris, France; ^8^Pathology, Assistance publique-Hôpitaux de Marseille, Hôpital de la Timone, Marseille, France; ^9^Pediatric Pulmonology, Assistance publique-Hôpitaux de Paris, hôpital Armand Trousseau, Paris, France; ^10^Pulmonology Department, CHU Nord, Assistance publique-Hôpitaux de Marseille, Marseille, France; ^11^Pediatric Pulmonology, Nephrology and Dermatology, Hospices civils de Lyon, Hôpital Femme-Mère-Enfant, Lyon, France; ^12^Pediatric Rheumatology, Nephrology and Dermatology, Hospices civils de Lyon, Hôpital Femme-Mère-Enfant, Lyon, France

###### **Presenting author:** Cecile Picard

**Introduction:** Inherited inflammatory syndromes related to the transmembrane protein 173 (*TMEM173*) gene gain-of-function mutation were described so far by the triad early-onset systemic inflammation, marked cutaneous vasculopathy and pulmonary fibrosis, with variable clinical expression even in familial cases (1,2). Recently, we have reported that *TMEM173* mutations can be associated to inaugural lung interstitial disease (ILD) with late-onset systemic symptoms (3).

**Objectives:** To investigate pathological features of the lung disease of STING-associated ILD.

**Methods:** Clinical and biological data, chest HRCT and lung biopsies were collected retrospectively in 2 children and one adult with *TMEM173* mutation. Paraffine-embedded tissues were stained for IFNa (clone MMHA-2), CXCL10, IFNAR1 and MxA and compared to healthy controls and non genetic ILD (systemic sclerosis).

**Results:** Initial presentation was related to symptoms suggestive of pulmonary fibrosis for all patients. Few systemic features appeared later on life and skin damage was mild, delaying the evocation of a systemic disease. Classical causes of familial interstitial lung disease were all excluded genetically and all three patients had an already known genetic variant of *TMEM173*. Cystic lesions on HRCT predominated in upper lobes with relative preservation of lower lobes. Immunohistological analysis showed fibrosis with destruction of alveolar architecture associated with follicular lymphoid infiltrates (neolymphogenesis) containing a majority of B cells CD20+. The overall architecture differs from other common ILD. Intra-alveolar infiltrate was made of macrophages and cholesterol crystal clefts was noticed for the 3 patients. Interestingly, there was no evidence for vasculopathy or microangiopathy. By contrast, a huge induction of IFN-related protein in the lung section of patients was observed as compared with controls. A strong interferon signature was found in all three cases.

**Conclusion:** The diagnosis of STING-related lung fibrosis should be considered as a differential diagnosis of ILD in children even with no or mild systemic features. The pathological study may represent a useful procedure to support the diagnosis.

Written informed consent were obtained from all the participants of the study.

**References**

1. Liu Y, Jesus AA, Marrero B, Yang D, Ramsey SE, Montealegre Sanchez GA, et al. Activated STING in a vascular and pulmonary syndrome. N Engl J Med. 2014 Aug 7;371(6):507–18.

2. Crow YJ, Casanova J-L. STING-associated vasculopathy with onset in infancy--a new interferonopathy. N Engl J Med. 2014 Aug 7;371(6):568–71.

3. Picard C, Thouvenin G, Kannengiesser C, Dubus JC, Jeremiah N, Rieux-Laucat et al. Severe pulmonary fibrosis as the first manifestation of interferonopathy (TMEM173 mutation). Chest. 2016 Accepted/In Press

**Disclosure of Interest**: None Declared

### P403 The characteristic features of the patients with deficiency of adenosine deaminase 2 (DADA2)

#### Ezgi Deniz Batu^1^, Hafize Emine Sonmez^1^, Abdulsamet Erden^2^, Ekim Z. Taskiran^3^, Omer Karadag^2^, Umut Kalyoncu^2^, İbrahim Oncel^4^, Berkan Kaplan^5^, Zehra Serap Arici^1^, Cagri Mesut Temucin^5^, Haluk Topaloglu^4^, Yelda Bilginer^1^, Mehmet Alikasifoglu^3^, Seza Ozen^1^

##### ^1^Department of Pediatrics, Division of Rheumatology, Ankara, Turkey; ^2^Department of Internal Medicine, Division of Rheumatology, Ankara, Turkey; ^3^Department of Medical Genetics, Ankara, Turkey; ^4^Department of Pediatrics, Division of Neurology, Ankara, Turkey; ^5^Department of Neurology, Hacettepe University Faculty of Medicine, Ankara, Turkey

###### **Presenting author:** Ezgi Deniz Batu

**Introduction:** Deficiency of adenosine deaminase 2 (DADA2) is an autosomal recessive autoinflammatory disease resulting from a loss-of-function mutation in *CERC1* gene encoding for ADA2 protein. The patients present with systemic inflammation and vasculopathy.

**Objectives:** We aimed to present the characteristics of the pediatric DADA2 cases.

**Methods:** The clinical and laboratory features of thirteen pediatric patients (8 M, 5 F), who were diagnosed with DADA2 at Hacettepe University Pediatric Rheumatology Department between 2014-2016, have been summarized. Mutations in *CECR1* were detected by Sanger sequencing.

**Results:** Eleven patients were homozygous for G47R mutation in *CECR1* gene, one was compound heterozygous for G47R and G47V while one patient was heterozygous for G47R. Seven of these patients had been followed up in our clinic with the diagnosis of polyarteritis nodosa (PAN). There was consanguinity in four cases, and two patients were siblings. The median (min-max) age at onset of the symptoms and diagnosis was 5.5 (1.4-19) and 120 (36-211) months, respectively. All patients suffered from recurrent episodes of fever with elevated acute phase reactants. Skin manifestations were as follows: livedo reticularis (n = 13), erythema nodosum (n = 4), and necrotic ulcers (n = 2). Neurological and musculoskeletal involvement was in the form of myalgia (n = 5), arthralgia (n = 10), arthritis (n = 5), stroke (n = 8), strabismus (n = 3), peripheral neuropathy (n = 6), and spinal cord atrophy (n = 1). One patient had been diagnosed with core myopathy through muscle biopsy which was thought as a co-incidental finding. Optic neuritis was detected in one patient. There was Raynaud’s phenomenon in two patients. Four patients had aneurysms in different middle-sized arteries (hepatic artery, renal artery, and superior mesenteric artery). One patient had intestinal perforation and ileostomy at the age of eight. As renal involvement, one patient had focal segmental glomerulosclerosis (collapsing variant), one mesangial proliferative glomerulonephritis (renal subcapsular hematoma after renal biopsy), and one had renal amyloidosis. There was testicular torsion in one patient. Three patients had positive antinuclear antibody while there was low levels of immunoglobulin M in two patients. Two adult patients died soon after diagnosis of DADA2. One patient was asymptomatic on only colchicine for almost 10 years. One responded to mycophenolate mofetil treatment. Rest of the patients responded well to anti- tumor necrosis factor therapy (etanercept).

**Conclusion:** ADA2 protein is a growth factor for endothelial cells and leukocytes and is important for stabilization of endothelial cells. For this reason, it causes autoinflammatory symptoms as well as vasculitis symptoms similar to PAN. ADA2 deficiency is a recently defined disease and the data about its phenotype increases with the introduction of new cases with different symptoms. This is the first description of spinal cord atrophy, core myopathy, and mesangial proliferative glomerulonephritis in DADA2 patients. Similar to the patients in the literature, most of our patients responded to etanercept therapy. Our cases suggest the diverse phenotype of DADA2.

**Disclosure of Interest**: None Declared

### P404 Pyrin-associated autoinflammation with neutrophilic dermatosis (PAAND): preliminary results of a proof-of-concept trial with anakinra

#### Lien Van Eyck^1,2^, Ellen De Langhe^3^, Isabelle Jéru^4,5^, Erika Van Nieuwenhove^1,2^, Vasiliki Lagou^2,6^, Paul J. Baker^7,8^, Jocelyn Garcia-Perez^1,2^, James Dooley^1,2^, Lien De Somer^9^, Raf Sciot^10^, Pierre-Yves Jeandel^11^, Julia Ruuth-Praz^4,5^, Bruno Copin^5^, Myrna Medley-Hashim^12^, Andre Megarbane^13^, Sinisa Savic^14^, An Goris^6^, Serge Amselem^4,5^, Adrian Liston^1,2^, Seth Masters^7,8^, Carine Wouters^1,9^

##### ^1^Department of Immunology and Microbiology, University of Leuven, Leuven, Belgium; ^2^Translational Immunology Laboratory, VIB, Leuven, Belgium; ^3^Department of Rheumatology, University Hospitals Leuven, Leuven, Belgium; ^4^Université Pierre et Marie Curie-Paris, UMR_S933, Paris, France; ^5^Service de Génétique et d’Embryologie médicales, Assistance Publique Hôpitaux de Paris, Hôpital Trousseau, Paris, France; ^6^Department of Neurosciences, University of Leuven, Leuven, Belgium; ^7^Inflammation division, The Walter and Eliza Hall Institute of Medical Research, Parkville, Australia; ^8^Department of Medical Biology, University of Melbourne, Parkville, Australia; ^9^Department of pediatrics, University Hospitals Leuven, Leuven, Belgium; ^10^Department of pathology, University Hospitals Leuven, Leuven, Belgium; ^11^Département de Médecine Interne, Hôpital Archet 1, Nice, France; ^12^Department of Life and Earth Sciences, Lebanese University, Beirut, Lebanon; ^13^Al-Jawhara Center, Arabian Gulf University, Manama, Bahrain; ^14^Department of Clinical Immunology and Allergy, St James’s University Hospital, Leeds, United Kingdom

###### **Presenting author:** Lien Van Eyck

**Introduction:** Recently, we identified a S242R mutation in pyrin in 12 patients from a three-generation Belgian family suffering from childhood-onset recurrent episodes of severe neutrophilic dermatosis, arthralgias/myalgias and systemic inflammation. The mutation results in the loss of a 14-3-3 binding motif at phosphorylated S242, resulting in continuous pyrin-inflammasome activation and excessive IL-1β production. Because of the genetic causation by pyrin mutation and the characteristic features of systemic inflammation and dermatosis, we termed this disease pyrin-associated autoinflammation with neutrophilic dermatosis (PAAND). We found additional patients in Lebanon, France and UK, where one patient received treatment with the IL-1 receptor antagonist monoclonal antibody (mAb) anakinra leading to a resolution of symptoms and normalisation of inflammatory markers.

**Objectives:** To assess the efficacy of the IL-1 receptor antagonist mAb anakinra (Sobi) in controlling the clinical and biochemical consequences of constitutive activation of the pyrin inflammasome in patients with PAAND.

**Methods:** Three Belgian patients with the S242R pyrin mutation are treated with daily subcutaneous injections of Anakinra 100 mg for 12 weeks. Previous therapy with TNF-antagonists was stopped and concomitant immunosuppressive treatment was limited to low-dose corticosteroids. Clinical cutaneous, articular/muscular manifestations and inflammatory markers are assessed at baseline, and after 2, 4, 8 and 12 weeks. In addition presence of cytokines and activated caspase1 as well as inflammasome activation *in vitro* are assessed at baseline, 4 and 12 weeks.

**Results:** At baseline, all three patients manifested neutrophilic dermatosis in the form of severe cystic acne and sterile abscesses, diffuse myalgias and arthralgias, fatigue and night sweats, and abdominal pain associated with diarrhea. Inflammatory markers were increased and mild anemia was present.

At the time of writing, i.e. two weeks after the start of anakinra treatment inflammatory markers were normalized in all three patients. All patients showed manifest improvement of the cutaneous manifestations, reduction of the gastro-intestinal pain with normalization of the stools and a reduction of the musculoskeletal pain. Night sweats had disappeared, but fatigue was still present.

**Conclusion:** PAAND is a new autosomal dominant autoinflammatory disease caused by a specific S242R mutation in *MEFV* leading to constitutive activation of the pyrin-inflammasome. The clinical phenotype is dominated by severe neutrophilic dermatosis and systemic inflammation. Treatment with Anakinra resulted in suppression of clinical manifestations and control of inflammatory markers, endorsing the key pathogenic role of IL-1 in PAAND.

**Disclosure of Interest**: None Declared

### P405 A fourteen-year-old girl with immunoglobulin G4-related disease

#### Nami Okamoto^1^, Yuko Sugita^2^, Kousuke Shabana^2^, Takuji Murata^2^, Hiroshi Tamai^1^

##### ^1^Department of Pediatrics, Graduate School of Medicine, Takatsuki-city, Japan; ^2^Pediatrics, Osaka Medical and Pharmaceutical University, Takatsuki-city, Japan

###### **Presenting author:** Nami Okamoto

**Introduction:** We will report a girl with immunoglobulin G4-related disease (IgG4-RD), a newly recognized disorder that share particular pathologic (infiltration of IgG4-rich plasma cells or fibrosis), serologic (elevated serum IgG4), and clinical features (multifocal phymatoid or hypertrophic lesions).

**Objectives:** Child patient with IgG4-RD is very rare and its pathophysiology is thought to be different from adult cases^1)^. We will report our patient whom treated with mycophenolate mofetil (MMF) for the purpose.

**Methods:** Retrospective analysis based on charts.

This report was approved by Ethics Committee of Osaka Medical and Pharmaceutical University.

**Results:** She complained exophthalmos when she was twelve years old. MRI was performed at former hospital that revealed tumefaction of lacrimal gland and salivary gland. Serum IgG4 concentration was significantly high (1090mg/dl) and biopsy specimen from salivary gland showed infiltration of IgG4-positive plasma cells (>50%). She was diagnosed as IgG4-RD and oral prednisolone (1mg/kg) was administered. Because of steroid-induced glaucoma, the disease control was difficult to manage and she was introduced to our hospital at age of thirteen. Laboratory data: WBC 10020/μl, PLT 31万/μl, CRP 0.01mg/dl, ESR 23mm/1h, IgG 2960mg/dl, IgA 45mg/dl, IgM 115mg/dl, IgE >5000Iu/ml, IgG4 1370mg/dl, CH50 41 U/ml, C3 90mg/dl, C4 15.6 mg/dl, AST/ALT 13/22U/L, γGTP 19U/L, BUN 15mg/dl, CRN 0.54mg/dl, autoantibodies negative, no abnormality in urine test. Abdominal CT and MRI were performed that showed IgG4-related cholangitis, cholecystitis and nephropathy. Bile duct wall was edematous and wall thickening was exhausted, so we diagnosed her status as CS-resistant. We prescribed MMF (800mg/kg) and she was responded clinically and serologically. Her disease activity has been controlled well since then. IgG4-RD is a male dominant disease and peak age is between forties and seventies in adults. Among pediatric patient of IgG4-RD, girls are double to boys and their age ranged from two to seventeen. Despite the recognition that pediatric IgG4-RD shows different characteristics, little was known about the pathogenesis, adequate therapy and prognosis yet. Though corticosteroid (CS) therapy was effective for most patients, half of them experienced relapse or recurrence with reducing CS^2)^. CS-sparing agents that include azathioprine, rituximab, cyclophosphamide and MMF are required for them. But it is not clear when it should be started and which agent would be adequate as a maintenance therapy.

**Conclusion:** Our patient with pediatric IgG4-RD who was resistant to CS monotherapy responded to MMF. Further study about children with this rare disorder and confirming the response to maintenance therapy is necessary in worldwide.

**References:**

 1) Karim F, et al. IgG4-related disease: a systematic review of this unrecognized disease in pediatrics. Ped Rheumatol. 2016;14:18.

2) Sekiguchi H, et al. Immunoglobulin G4-related disease: Retrospective analysis of 166 patients. Arthritis Rheumatol. 2016 Mar 18. doi:10.1002/art.39686. [Epub ahead of print]

**Disclosure of Interest**: None Declared

### P406 Autoimmune damage of CNS - a newly recognized clinical manifestation of autoimmune polyendocrine syndrome type 1?

#### Juliana Ferenczová, Erika Banóova, Pavol Mrážik, Veronika Vargova

##### Department of Paediatrics and Adolescent Medicine, Šafárik University and children faculty hospital in košice, Košice, Slovakia

###### **Presenting author:** Veronika Vargova

**Introduction:** Autoimmune polyendocrine syndrome (APS) type 1, also known as autoimmune polyendocrinopathy-candidiasis-ectodermal dystrophy (APECED), is a rare autosomal recessive disease. The classic features are chronic mucocutaneous candidiasis, hypoparathyroidism and adrenocortical failure. Several non-classic presentations of the disease have been described over the last few years.

**Objectives:** Authors present a case of 14-year old girl with prolonged seizures as a new serious non-classic presentation of APS type 1.

**Methods:** case report

**Results:** Patient was diagnosed with a mucocutaneous candidiasis and hypoparathyroidism at the age of 3 years. APS 1 was diagnosed at 9 years of age shortly after diabetes mellitus type 1 had been recognized. Mutational analysis of the AIRE gene showed R257X (c.769C > T) mutation. Typical symptoms of Addison disease - weakness, fatigue and hyperpigmentation - developed at 13 years of age. At 14 years she was admitted to hospital for prolonged seizures. Hypoglycemia and hypocalcemia were excluded as a cause of the seizures. EEG showed finding non-specific for epilepsy. Examination of the CSF was performed to exclude infection (herpetic viruses, etc.). MRI angiography did not show vasculitis or other organic changes of cerebral vessels. Specific autoantibodies for autoimmune encephalitis turned out to be positive in CSF (antiGAD65 > 2000 MU/L) as well as in blood (>2000 MU/L). Immunosuppressive treatment (glucocorticoids and i.v. immunoglobulins) in combination with antiepileptics resulted in a clinical course without seizures.

**Conclusion:** Differential diagnosis of autoimmune damage of CNS is a new challenge for pediatric rheumatologists. Autoimmune GAD65-positive encephalitis should be considered a new component of the clinical spectrum of APS in patient with new onset neuropsychiatric symptoms.

**Disclosure of Interest**: None Declared

## Poster Session: Spondyloarthritis (SpA) and enthesitis related arthritis (ERA)

### P407 Color Doppler us as an alternative to MRI for detecting sacroiliitis - a pilot study

#### Dubravko Bajramovic^1^, Ksenija Stekic Novacki^1^, Kristina Potocki^1^, Marijan Frkovic^2^, Marija Jelusic^2^

##### ^1^Department of radiology, University Hospital Center Zagreb, Zagreb, Croatia; ^2^Department of pediatrics, University Hospital Center Zagreb, Zagreb, Croatia

###### **Presenting author:** Dubravko Bajramovic

**Introduction:** Considering that the ultrasound is a validated method for assessing inflammation activity in peripheral joints our aim in this pilot study was to determine whether the ultrasound/Color Doppler could be used to diagnose sacroiliitis in juvenile SpA patients.

**Objectives:** To determine the value of ultrasound/Color Doppler in diagnosing acute sacroiliitis in comparison with the established method which is considered a standard - the MRI. To compare data collected from our jSpA patients with previously published predisposing factors for the development of sacroiliitis.

**Methods:** We performed ultrasound/Color Doppler and MRI of sacroiliac joints using a standardized protocol on 112 patients observed for jSpA. All patients had low back pain and clinically suspected acute sacroiliitis. Out of 112 patients 43 were male and 69 female with average age of 13.9. Patients age at the beginning of the disease, number and sequence of joint involvement, history of rheumatic diseases in the family and results of HLA serotyping were recorded. All ultrasound and MRI examinations were performed by two MSK radiologists with an experience in imaging methods and protocols for rheumatic diseases.

**Results:** Active disease or acute sacroiliitis was noted using ultrasound/Color Doppler examination in 31 patients while 81 showed no signs of synovitis. MRI detected 28 patients with active disease. Only eight patients had ultrasound signs of disease activity confirmed on MRI. Based on preliminary results sensitivity of ultrasound/Color Doppler examination in detecting acute sacroiliitis in jSpA patients is 28.6% and specificity is 72.6%. Positive predictive value is 0.26 while negative predictive value is 0.75. Out of 41 patients with sacroiliitis confirmed on the MRI (both active and chronic) 27 of them had positive HLA- B27 serotyping, 9 had hip joint inflammation at the disease onset and only 5 had positive family history of rheumatic diseases.

**Conclusion:** MRI is a method of choice for detection of acute sacroiliitis but due to the waiting lists it can take a substantial amount of time before the examination can be performed. In order to get the diagnostic information as soon as possible ultrasound examination is often indicated. Ultrasound is an available, cheap and relatively easy method to perform and it doesn’t have the harmful effects of radiation which is especially important in the pediatric population. In sacroiliac joints imaging ultrasound has serious limitations due to the complex joint anatomy and a relatively small imaging “window” that enables us to visualize and analyze only the dorsal aspect of the joint. Evaluator has to make sure to that the Doppler signals acquired are the result of the increased vascularization within the synovia in the intraarticular space and not the result of imaging of the blood vessels in the periarticular soft tissues. Optimizing the image parameters is extremely important as well as imaging experience. As our preliminary results show using ultrasound CD as an alternative to MRI is questionable due to the low sensitivity and specificity of the method resulting from the limitations mentioned. Ultrasound is not the adequate method for demonstrating acute sacroiliitis and patients with high clinical suspicion of sacroiliitis should perform MRI in order to confirm the diagnosis.

**Disclosure of Interest**: None Declared

### P408 Longterm experience of biologics therapy in juvenile spondyloarthritis: focus for the drug survival

#### Irina Nikishina, Olga Kostareva, Svetlana Arsenyeva, Maria Kaleda, Anna Shapovalenko

##### V.A.Nasonova research Institute of Rheumatology, MOSCOW, Russian Federation

###### **Presenting author:** Irina Nikishina

**Introduction:** Juvenile spondyloarthritis (JSpA) included several subtypes of juvenile arthritis, the main of there are follows: juvenile onset of ankylosing spondylitis (JAS), enthesitis-related arthritis (ERA), juvenile psoriatic arthritis (JPsA). Biologics, TNF-inhibitor mostly, provided excellent progress in JSpA outcome for the last 15 years. Drug survival is a general marker of the treatment success as it depends of its efficiency and safety.

**Objectives:** To compare drug survival of biologics depending on the subtype in JSpA patients (pts) in real clinical practice of single center.

**Methods:** The study involved a prospective cohort of JSpA pts treated by different TNF-inhibitors in our clinic from 2004 to 2016. Analyze included drug survival with Kaplan-Meier, reasons of withdrawals and adverse event (AE) rates.

**Results:** 177 JSpA pts, treated with one or more biologics were analyzed. At the start of biologics pts average age was 13.19 ± 3.7 years (range 3.58-17.9); mean disease duration was 50,4 month (range 2-163). JSpA subtypes were as follows: JAS, fulfilled to modified New-York criteria - 48(26%), ERA - 106(60%) and JPsA - 23(13%), diagnosed according to ILAR (2001) criteria, 149(83%) were HLA-B27positive. 24(13,4%) had uveitis. In total, 205 treatment series, including 79 for etanercept (185 patient-year PY), 73-adalimumab (160 PY), 53-infliximab (182 PY) were evaluated. There were 62 cases of withdrawals. Reasons for withdrawals were AE in 7,32% (95%CI 4.49-11.72), inefficacy -i10.73% (95%CI 7.19-15.71), others – 11,71% (95% CI 8-16,83), basically due to organizational difficulties of biologics access in adult life. AE were developed after at mean 1.63 years (range 0.74-3,36). AE were included: etanercept – 3 cases of uveitis de novo (1.62 per 100 PY); infliximab – 6 infusion reactions (3.3 per 100 PY), 1 psoriasis de novo, 1 severe skin disorder, 1 toxic hepatitis, 2 tuberculosis; adalimumab – 2 psoriasis de novo, 1 tuberculosis. AE as a reason of Infliximab withdrawal was observed more often than under adalimumab or etanercept (18.9% vs 2.74% and 3.8% respectively, p < 0.05). Due to inefficacy Infliximab was cancelled more often than adalimumab (16.9% vs 6.85%, p < 0.05). Etanercept was rare withdrawn due to organization problem in compare to infliximab and adalimumab (6.3% vs 16.98% and 13.7%, p < 0.05). Long-term drug survival of TNF-inhibitors was 78% for ERA, 64% for JAS and 57% for JPsA. Drug survival for etanercept were 95% and 35% after 1 and 5 years, infliximab – 90% and 38%, adalimumab – 97% and 32%, respectively. The average survival rate of etanercept and adalimumab was higher in ERA vs JAS and JPsA (84% vs 68% and 67% respectively), but for infliximab it was similar in all JSpA subtypes.

**Conclusion:** Our experience of long-term study of TNF-inhibitors in JSpA demonstrated the best rate of drug survival for etanercept in pts with different subtype of JSpA, especially in ERA. Infliximab mostly withdrawn due to AE and inefficacy, but adalimumab cancelled more often because of organizational reasons, especially in adult life.

**Disclosure of Interest**: None Declared

### P409 Concomitant MRI features of sacroiliitis increase specificity for diagnosis of juvenile spondyloarthritis

#### Lennart Jans^1^, Nele Herregods^1^, Jacob Jaremko^2^, Rik Joos^1^, Joke Dehoorne^1^

##### ^1^Ghent University Hospital, Gent, Belgium; ^2^University of Alberta, Edmonton, Canada

###### **Presenting author:** Lennart Jans

This abstract is not included here as it has already been published.

### P410 Is contrast necessary to detect sacroiliitis on MRI in juvenile spondyloarthritis?

#### Nele Herregods^1^, Jacob Jaremko^2^, Xenofon Baraliakos^3^, Joke Dehoorne^4^, Rik Joos^4^, Lennart Jans^1^

##### ^1^Department of Radiology and Medical Imaging, Ghent University Hospital, Ghent, Belgium; ^2^Department of Radiology & Diagnostic Imaging, University of Alberta Hospital, Edmonton, Canada; ^3^Rheumazentrum Ruhrgebiet, Ruhr-University Bochum, Herne, Germany; ^4^Department of Pediatric Rheumatology, Ghent University Hospital, Ghent, Belgium

###### **Presenting author:** Nele Herregods

This abstract is not included here as it has already been published.

### P411 Performance of disease activity measures in juvenile spondyloarthritis in a placebo controlled trial with infliximab

#### Sofia Ramiro^1^, Julio C. Casasola-Vargas^2^, Désirée van der Heijde^1^, Robert Landewé^3^, Ruben Burgos-Vargas^2^

##### ^1^LUMC, Leiden, Netherlands; ^2^Hospital General de Mexico, Mexico, Mexico; ^3^ARC, Amsterdam, Netherlands

###### **Presenting author:** Ruben Burgos-Vargas

**Introduction:** Several outcome measures in trials with juvenile-onset spondyloarthritis (Jo-SpA) have been borrowed from trials in juvenile idiopathic arthritis and from adult spondyloarthritis, but a proper psychometric analysis has never been conducted in patients with Jo-SpA.

**Objectives:** To assess discriminatory aspects of several disease activity outcome measures and response criteria for Jo-SpA.

**Methods:** Data from a previously reported 12-week RCT comparing infliximab (IFX) and placebo (PBO) in patients <18 years with Jo-SpA and onset <16 years of age were analyzed. The primary endpoint of the trial was the number of active joints (both swollen and tender). Several other disease activity measures and response criteria were also tested (Table 5). Statistics to determine how well disease activity measures could discriminate between IFX and PBO included ‘standardized mean difference’ (SMD) and ‘Guyatt’s effect size. Both statistics are standardized measures to compare change from baseline per group. For categorical response criteria, the chi-square test (χ^2^) was used. Higher numbers indicate better discriminatory capacity.

**Results:** Patients were randomised to IFX (n = 12) and PBO (n = 14). Of the continuous measures, the ASDAS showed the best and very good discrimination between IFX and PBO (SMD:1.98; Guyatt: 4.28) (Table 5). The physician’s global, CRP, JADAS and JSpADA also discriminated well. The BASDAI (or its separate items), BASFI and spinal mobility measures performed worse. Of the response criteria ASAS40 (IFX 55% vs PB = 0%, χ^2^ 10.05) and ACR Pedi 90 (IFX 67% vs PBO 7%, χ^2^ 10.12) discriminated best between IFX and PBO. ASDAS response criteria (ASDAS-MI: IFX 63% vs PBO 0%, χ^2^ 8.65, ASDAS-CII IFX 88% vs PBO 20%, χ^2^ 8.10) and ACR Pedi 30-70 also performed well.

**Conclusion:** Of all continuous measures tested in adult axial SpA the ASDAS discriminates best between active treatment and PBO in patients with Jo-SpA. But the child specific JSpADA also performs well.

Of all response criteria tested the child-specific ACR Pedi 30 to 90, as well as the adult ASAS40 and ASDAS response criteria work well. One of these measures should be used as primary endpoint in trials with Jo-SpA.

**Disclosure of Interest**: None Declared

**Table 5 (abstract P411).** Discrimination between patients on infliximab and placebo at week 12

	Infliximab mean change (SD)	PBO mean change (SD)	Guyatt’s effect size	SMD
ASDAS	2.4 (1.3)	0.5 (0.6)	4.28	1.98
Physician’s global assessment, 0-10 mm VAS	5.2 (2.4)	1.6 (2.2)	2.34	1.56
JADAS27 (0-57)	12.7 (5.9)	4.3 (5.7)	2.22	1.46
JSpADA (0-8)	2.8 (1.2)	0.5 (1.4)	1.98	1.73
CRP (mg/L)	21.1 (8.4)	2.3 (10.9)	1.93	1.90
Total enthesitis (0-51)	8.5 (10.6)	1.6 (5.0)	1.71	0.85
Patient’s global assessment, 0-10 mm VAS	4.3 (3.8)	0.8 (2.8)	1.55	1.08
BASDAI total (0-10)	3.3 (3.1)	0.9 (2.3)	1.41	0.90
Active joint count (0-72)	4.4 (1.7)	2.6 (4.6)	0.96	0.51

### P412 Efficacy and safety of adalimumab in pediatric patients with enthesitis related arthritis

#### Ruben Burgos-Vargas^1^, Shirley M. Tse^2^, Gerd Horneff^3^, Kristina Unnebrink^4^, Jaclyn K. Anderson^5^

##### ^1^Hospital General de Mexico, Universidad Nacional Autonoma de Mexico, Mexico City, Mexico; ^2^University of Toronto, The Hospital for Sick Children, Ontario, Canada; ^3^Asklepios Klinik Sankt Augustin, Sankt Augustin, Germany; ^4^AbbVie Deutschland GmbH & Co. KG, Ludwigshafen, Germany; ^5^AbbVie Inc, North Chicago, IL, United States

###### **Presenting author:** Ruben Burgos-Vargas

This abstract is not included here as it has already been published.

### P413 Evaluation of DMARDs effectiveness on ERA patients

#### Aysenur Paç Kisaarslan^1^, Betül Sözeri^1^, Zübeyde Gündüz^1^, Gökmen Zararsız^2^, Hakan Poyrazoğlu^1^, Ruhan Düşünsel^1^

##### ^1^Pediatric Rheumatology, Erciyes University, Kayseri, Turkey; ^2^Bioistatistic, Erciyes University, Kayseri, Turkey

###### **Presenting author:** Aysenur Paç Kisaarslan

**Introduction:** Therapeutic options for Enthesitis Related Arthritis (ERA) include monotherapy or combination therapy with non-steroidal anti-inflammatory drugs, disease-modifying anti-rheumatic drugs (DMARDs), and biological agents. Until today, the approach to managing ERA is guided in large part by evidence from studies in adults with SpA and other forms of JIA.

**Objectives:** This study aim is determined whether effectiveness of DMARDs in ERA patients.

**Methods:** Fiftytwo ERA patients who satisfied ILAR criteria were enrolled in the study. The patients with psoriasis, inflammatory bowel disease, reactive arthritis and undifferentiated arthritis were exculuded. Patient information was obtained from the records. Registered data included demographic features, medical history, initial and following physical examination, initial juvenile spondyloarthritis disease activity index(JSpADA), initial laboratory tests, radiographic tests, JADI -A (Juvenil Arthritis Demage Index-articulary) and JADI-E(extraarticulary) on last admission, and all of medical treatment.

**Results:** We evaluated response of DMARDs in 52 patients. Twenty-seven patients (52%) achieved remission with DMARDs, while 25(48%) patients were not achived remission with DMARDs. Diagnosis of age, gender, family history of AS, inflammatory back pain, shoulder, hip, small joint involvement, sacroileitis with physical examination, enthesitis, presence of uveitis, presence of HLA-B27, initial high AFR levels, initial JSpADA score, last JADI-A score did not determinated risk factor on unresponsiveness of DMARD(HR:1, p > 0.05). Although it was not statistically significant, sacroileitis determinated with MRI increased 2.84 fold on unresponsiveness of DMARD with cox-regression analysis (p > 0.05). DMARD effectiveness decreased 50% at 20^th^ month in this group with Kaplan-Meier analysis.

**Conclusion:** There are no significant differencies periferic between axial involvement treated with DMARD in our study. Therefore we believe that DMARDs kept up to date.

**Disclosure of Interest**: None Declared

### P414 Assessment of entheseal ultrasonography in patients with juvenile fibromyalgia

#### Kazutaka Ouchi, Shinji Akioka, Hiroshi Kubo, Norio Nakagawa, Hajime Hosoi

##### Pediatrics, Kyoto Prefectural University of Medicine, Kyoto, Japan

###### **Presenting author:** Kazutaka Ouchi

**Introduction:** Fibro myalgia (FM) is characterized by widespread chronic musculoskeletal pain with fatigue, poor sleep, frequent psychological difficulties, and multiple tender points on physical examination. In adult cases, American College of Rheumatology 2010 (ACR2010) criteria was proposed as clinically useful tool, and the analyses of the underlying disease of FM based on this criteria demonstrated that some FM cases are complicated with SpA, one of which pathogenesis is enthesitis. Here, we examined ultrasound findings on entheses of juvenile FM patients.

**Objectives:** To identify the ultrasound (US) picture of peripheral entheses in juvenile patients with FM.

**Methods:** A single-center study was performed in 8 patients with definite juvenile FM according to suggestive symptom (a widespread pain resulting in school absenteeism). US assessment of the following four entheses was performed bilaterally by a senior rheumatologist: distal Achilles tendon, distal and proximal patellar tendon, and quadriceps tendon.

**Results:** The patients were 6 women and 2 men with a mean age of 14 ± 1 years. Total 64 entheses was examined. Inflammatory enthesitis was detected in 36 entheses (56.3%), and enthesopathy detected in 8 entheses (12.5%). All of patients had at least one lesion: inflammatory enthesitis was detected in 8 patients (100.0%), and enthesopathy detected in 6 patients (75.0%).

**Conclusion:** All juvenile FM cases were found to have enthesitis by US. Our result showed that a part of juvenile FM patients are probably complicated with enthesitis.

**Disclosure of Interest**: None Declared

### P415 Epigenetic regulation of PTPN12 gene could influence the immunopathogenesis of juvenile spondyloarthritis and give rise to new therapeutic options

#### Lovro Lamot^1,2^, Fran Borovecki^2^, Sanja Kapitanovic^3^, Kristina Gotovac^2^, Mandica Vidovic^1^, Mirta Lamot^1^, Edi Paleka Bosak^1^, Miroslav Harjacek^1^

##### ^1^Clinical Hospital Center Sestre Milosrdnice, Zagreb, Croatia; ^2^University of Zagreb School of Medicine, Zagreb, Croatia; ^3^Ruđer Bošković Institute, Zagreb, Croatia

###### **Presenting author:** Lovro Lamot

**Introduction:** Gene expression profiling of juvenile spondyloarthritis (jSpA) patients revealed aberrant expression of some genes, but the mechanism of this expression alterations remained unknown (1).

**Objectives:** To discover epigenetic modifications with effect on regulation of 4 genes (TLR4, NLRP3, CXCR4, PTPN12) differentially expressed in jSpA patients.

**Methods:** DNA methylation analysis was performed in promotor region of differentially expressed genes by methylated DNA Immunoprecipitation (meDIP). Based on literature search, 4 microRNAs (miR-150, miR-146a, miR-181a, miR-223) included in regulation of differentially expressed genes were selected and their expression was analyzed using RT-PCR with predeveloped Taqman microRNA assays. Both analysis were performed in 7 newly diagnosed untreated jSpA patients and 7 matched healthy children.

**Results:** Statistical analysis revealed no difference in methylation of promotor sites for examined genes, but PTPN12 showed trend towards higher methylation (p = 0,076), compared with control group. There was no statistical difference in expression of selected miRs between two groups.

**Table:** 

PATIENTS	1	2	3	4	5	6	7
PTPN12 (*fold enrichment*)	0,43	1,01	0,54	1,84	1,06	5,08	0,55
	0,95	0,57	1,08	0,81	0,886	1,26	0,586
CONTROLS	1	2	3	4	5	6	7

**Conclusion:** jSpA is a multifactorial disease in which a complex interplay occurs between the immune system and environmental factors on a predisposing genetic background. One of the most important mechanisms by which environment can influence processes inside of an organism is gene regulation via epigenetic modifications. Therefore, we investigated possible influence of DNA methylation and posttranscriptional modifications on the genes differentially expressed in jSpA patients. The results didn’t indicate any statistically significant abundance of miRs with described role in expression of examined genes, nor hypermethylation of their promotor sites, but there was a clear trend of higher methylation of PTPN12 in jSpA patients, while lack of statistical significance could be attributed to small number of study participants. This observation can easily explain decrease of PTPN12 expression in treated and untreated jSpA patients. PTPN12 expressed in dendritic cells is identified as a key regulator of dendritic cell migration as well as T cell-dependent immunity and autoimmunity (2). It is also a negative regulator of inflammation and intestinal cell migration, as well as a positive regulator of osteoclast activity, all of which are processes important for the pathophysiology of jSpA. Results of our study are in concordance with previous epigenetic studies of this gene in human breast cancer patients, which showed PTPN12 can be silenced by methylation. Interestingly, more recent study pointed to the potential of 5-Azac, a DNA hypomethylating agent, in increasing PTPN12 expression, and highlighted the therapeutic potential of this mechanism in treatment of breast cancer (6). Therefore, it is tempting to speculate the results of our study could after confirmation in larger cohorts give rise for new treatment options in jSpA patients as well.

**References:**

1. Lamot L, et al. Aberrant expression of shared master-key genes contributes to the immunopathogenesis in patients with juvenile spondyloarthritis. PLoS One. 2014 Dec 15;9(12):e115416.

2. Inmoo Rhee, et al. Control of dendritic Cell Migration, T Cell-Dependent Immunity, and Autoimmunity by Protein Tyrosine Phosphatase PTPN12 Expressed in Dendritic Cells. Mol Cell Biol. 2014 Mar;34(5):888-99.

3. Thummuri D, et al. Epigenetic regulation of protein tyrosine phosphatase PTPN12 in triple-negative breast cancer. Life Sci. 2015 Jun 1;130:73-80.

**Disclosure of Interest**: None Declared

### P416 Anti-tumor necrosis factor therapy in patients with enthesitis-related arthritis

#### Ricardo A. Russo, María M. Katsicas

##### Immunology & Rheumatology, Hospital de Pediatría Garrahan, Buenos Aires, Argentina

###### **Presenting author:** Ricardo A. Russo

**Introduction:** Enthesitis-related arthritis (ERA) is a category of Juvenile idiopathic arthritis considered to be a form of Juvenile Spondyloarthropathy (jSpA). Tumor necrosis factor (TNF)-blocking strategies have proven effective in the treatment of jSpA^1-2^.

**Objectives:** To assess effectiveness of anti-TNF treatment in a cohort of patients with ERA; to search for associations between clinical features at treatment onset and inactive disease (ID).

**Methods:** Retrospective review of prospectively collected data. Patients with ERA (according to ILAR criteria) continuously treated with anti-TNF agents for ≥ 3 months were included. At visit 0 (baseline) and every 3 months thereafter Information was collected on therapeutic agents used and the following disease activity measures: joint count, pain score (0-10), presence of active enthesitis (AE), sacroiliac pain (SIP) or lumbar limitation (LL), wellbeing according to the patient using a visual analogue scale (VASp, 0-10), disease activity according to the physician (VASphy, 0-10), JADAS-10^2^, JSpADA^3^, and ESR. Functional capacity was also assessed through the use of CHAQ. Occurrence of ID (defined as JADAS-10 £ 1 or as per Wallace et al^4^) and clinical remission (CR)^4^ was recorded. ANOVA and Mann-Whitney U test were used for comparisons between visits, while associations were analyzed with chi square and Sperman’s rank correlation.

**Results:** 30 patients fulfilled inclusion criteria. Patients were followed at a specialized clinic during the period 2002-2015.They were all male, 43% HLA-B27 positive; median age at start of TNF-blocker therapy was 13 years, disease duration 3.5 years; median observation period was 18 months. Twenty-seven (90%) children showed peripheral arthritis while 22 (73%) exhibited sacroiliitis on MRI/Xrays at start of therapy. Patients received etanercept (23, 2 were later switched to adalimumab and 1 to infliximab), adalimumab (5), or infliximab (2). At baseline patients showed (medians): active joints (AJ) 4, pain 1.5, VASp 2, VASphy 2, JADAS-10 9.75, JSpADA 2.5, ESR 16.5 mm/h, and CHAQ 0.25. AE was present in 4 (13%), SIP in 7 (23%), and LL in 12 (40%) children. All patients showed JSpADA > 0 and 29 (97%) children had JADAS > 1 at entry. At 3 months JADAS-10 decreased to 5.5 (p = 0.007) and JSpADA to 1.25 (p = 0.002); 6 (21%) patients achieved ID. At 12 months patients showed AJ 0 (p = 0.01), pain 0 (p = 0.009), VASp 0 (p = 0.0005), VASphy 0.75 (p = 0.0001), JADAS-10 3 (p = 0.0001), JSpADA 1 (p = 0.0004), ESR 6 mm/h (p = 0.02), and CHAQ 0 (p = 0.12). AE was present in 0 (p = 0.08), SIP in 0 (p = 0.01), and LL in 6 (24%) (p = 0.03) patients. ID was recorded in 22 (73%) and CR in 9 (30%) children during the observation period. Achievement of ID at 12 months was associated with baseline presence of active sacroiliitis (p = 0.02).

**Conclusion:** In this retrospective cohort of ERA patients on TNF-blockers a significant and progressive reduction in disease activity measures was observed. Patients with baseline axial involvement appear to benefit most from this therapy.

**References**

1. Hugle B, Burgos-Vargas R, Inman RD, et al. Long-term outcome of antitumor necrosis factor alpha blockade in the treatment of juvenile spondyloarthritis. Clin Exp Rheumatol 2014;32:424-31.

2. Consolaro A, Ruperto N, Bazso A, et al. Development and validation of a composite disease activity score for Juvenile Idiopathic Arthritis. Arthritis Care Res 2009;658-66.

3. Weiss P, Colbert RA, Xiao R, et al. Development and Retrospective Validation of the Juvenile Spondyloarthritis Disease Activity Index. Arthritis Care Res 2014;66:1775-82.

4. Wallace CA, Ruperto N, Giannini EH. Preliminary criteria for clinical remission for select categories of juvenile idiopathic arthritis. J Rheumatol 2004;31:2290-4.

**Disclosure of Interest**: None Declared

### P417 An audit to determine the characteristics of axial symptoms and imaging studies in children/adolescents with juvenile-onset spondyloarthritis/enthesitis related arthritis described in the literature

#### Ruben Burgos Vargas^1^, Ana L. Ortiz-Peyegahud^2^

##### ^1^Rheumatology, Mexico, Mexico; ^2^Hospital General de Mexico, Mexico, Mexico

###### **Presenting author:** Ruben Burgos Vargas

**Introduction:** Peripheral arthritis and enthesitis characterize the onset and course of JoSpA/ERA. Axial symptoms (ax-symp) are rare at onset and increasingly frequent in patients at risk of ankylosing spondylitis (AS). Unfortunately, most papers, including those mentioning the distinctive term “inflammatory back pain” (IBP) lacked of precision about symptoms.

**Objectives:** To identify the elements that define ax-symp in children and adolescents with joSpA/ERA.

**Methods:**

We extracted information regarding ax-symp, clinical signs, and imaging studies referring to spinal and sacroiliac joints and entheses in joSpA/ERA patients from series of cases, case-control and cohort studies appeared in PubMed from 1966 to 2016.

**Results:** 58/1405 articles mentioned scarce, ambiguous, and non-specific clinical and imaging data about the involvement of the spine and sacroiliac joints and entheses. Only nine studies described timing; five referred to IBP characteristics, and four to imaging data. The analysis suggests ax-symp occur within five years from onset in up to 50% of the patients; radiographic sacroiliitis is seen and AS diagnosis is made 7.5 to 15 years after onset; syndesmophytes are rare before the of 20 years. MR studies seem to add nothing new yet.

**Conclusion:** Few papers describe timing and characteristics of ax-symp and radiographic sacroiliitis in children with joSpA/ERA evolving to AS. In contrast, most papers, including AS, do not present enough support to confirm ax-symp in joSpA/ERA patients, including those with AS; the effect of these factors may increase the proportion of false positives in the clinic and in clinical trials.

**Disclosure of Interest**: None Declared

### P418 Juvenile-onset Ankylosing Spondylitis(JAS) and its bone metabolism

#### Zhang Pingping^1^, Mou Yikun^1^, Qi Jun^2^, Jiang Yutong^2^, Gu Jieruo^2^

##### ^1^Pediatrics, The Third Affiliated Hospital of Sun Yat-Sen University, Guangzhou, China; ^2^Rheumatology, The Third Affiliated Hospital of Sun Yat-Sen University, Guangzhou, China

###### **Presenting author:** Zhang Pingping

**Introduction:** Ankylosing spondylitis (AS) is a chronic systemic inflammatory disease which characteristically involves the axial skeleton, enthesis regions or peripheral joints.As a chronic disease, AS generate high socioeconomic costs and AS cases require lifelong treatment. Severe pain and spine destruction causes high prevalence of work-related disabilities and a decrease in quality of life, which is a huge burden for the case, their families and the society.According to their onset age,the patients was classified to, Juvenile-nset Ankylosing Spondylitis (JAS), whose onset symptoms before the age of 16 years, and Adult-onset Ankylosing Spondylitis(AAS). Clinical phenotype and prognosis of JAS is different from AAS, but up to now scarce information about JAS was reported, especially the related bone metabolism mechanism.

**Objectives:** To analyse bone metabolic index(BMI) changes on patients with Juvenile-onset Ankylosing Spondylitis(JAS)and their relations with clinical index.

**Methods:** To collect the clinical data of JAS,such as C-reactive protein (CRP), blood sedimentation (ESR),the disease activity index (BASDAI),functional index (BASFI) and bone mineral density (BMD), detect the serum bone metabolism index,such as Wnt-3a, BMP-2, Dkk-1, 25 (OH) D and ICTP, then group these data and do statistical analysis.

**Results:** Osteopenia and VitD lack were widespread in JAS. Compared with controls, The elevated Wnt-3a and reduced Dkk-1 levels were not significant in JAS (P > 0.05), and there was no significant correlation between the two indexes and other BMI, course, clinical inflammation index, BASDAI and BASFI.However,the serum ICTP and BMP-2 were increased significantly in JAS, and both the CRP and BMP-2 were positive related with serum Wnt-3a(r was 0.271 and 0.250 respectively, P < 0.05).Correlation analysis shew there was positive correlation between serum 25 (OH) D and hip BMD in JAS(r = 0.383, P < 0.01), and there was negatively correlation between ICTP and BASDAI (r = -0.405 and -0.273 respectively, P < 0.05).Besides,regression analysis shew that both serum 25 (OH) D and the disease course were independent prognostic factors of patients’ hip BMD decreasing, where as CRP and ICTP were also independent prognostic factors of patients’ BASDAI. BMI in JAS with different clinical phenotypes were compared, BMP-2 level was much higher in JAS with hip joint involvement than other groups(P = 0.032), while there was no significant difference in serum 25(OH)D,ICTP,Wnt-3a and Dkk-1 level in all groups(P > 0.05).

**Conclusion:** Bone metabolism imbalance exists in JAS,and BMP pathway played an important role in the JAS with hip lesions and deserves further research.

**Disclosure of Interest**: None Declared

## Poster Session: Systemic JIA II

### P419 Identification of best cut-off points and clinical signs for discrimination of rheumatic mask of hematooncology disease and systemic onset of juvenile idiopathic arthritis

#### Mikhail M. Kostik^1^, Shilova Ekaterina^1^, Ilia Avrusin^1^, Yuriy Korin^1^, Olga Kopchak^2^, Eugenia Isupova^1^, Irina Chikova^1^, Panova Tatyana^3^, Margarita Dubko^1^, Vera Masalova^1^, Ludmila Snegireva^1^, Tatyana Kornishina^1^, Olga Kalashnikova^1^, Vyacheslav Chasnyk^1^

##### ^1^Hospital Pediatry, Saint-Petersburg State Pediatric Medical University, Saint-Petersburg, Russian Federation; ^2^Pediatry, Kirov’s regional children’s hospital, Kirov, Russian Federation; ^3^Pediatry, Leningrad regional children’s clinical hospital, Saint-Petersburg, Russian Federation

###### **Presenting author:** Mikhail M. Kostik

**Introduction:** Systemic onset of juvenile idiopathic arthritis (SoJIA) is a diagnosis of exclusion and required a broad spectrum of differential diagnosis. Patients with malignant hematooncology diseases (HOD) can have the similar to SoJIA symptoms as fever, lymphadenopathy, hepatosplenomegaly, joints pain and arthritis, increased ESR, CRP, anemia and should be discriminated from SoJIA. Due to presence of rheumatic masks children with HOD can be admitted to rheumatologic department.

**Objectives:** The aim of our study was to find the best cut-off points and clinical signs allowed to discriminate patients who have the rheumatic masks of HOD from patients with SoJIA.

**Methods:** In the retrospective study were included 86 patients with SoJIA and 21 children with HOD who were admitted in the rheumatology department due to rheumatic masks with initial provisional diagnosis - SoJIA. The HOD group was presented with patients with acute lymphoblastic leukemia (n = 18), neuroblastoma (n = 1) and lymphomas (n = 2). We evaluated the presence of main clinical signs in both groups and detected the best cut-off points of qualitative variables with ROC-analysis and analysis of sensitivity and specificity. We calculated the diagnostic odds ratios (DOR) for identification the best cut-off parameters. For comparison of two independent groups was used Mann-Whitney test, chi-square test and Fisher’s exact test.

**Results:** There were no differences in onset age, presence of hepatosplenomegaly, lymphadenopathy, lung, CNS involvement, levels of hemoglobin, AST, LDH, ALP, GGTP, ferritin and sodium levels between both groups (table). The main diagnostic signs, allowed to discriminate HOD from SoJIA were: number of active joints ≤3 (DOR = 4.4 (95%CI:1.5-13.2), p = 0.005), CRPБ < 15 mg/l (DOR = 5.6 (95%CI:1.7-18.4), p = 0.002), PLT ≤ 307x10^9^/l (DOR = 22.9 (95%CI:4.9-107.0), p = 0.0000001), WBC ≤ 8.9x10^9^/l (DOR = 50.2 (95%CI:6.3-401.3), p = 0.0000001), albumin > 43.3% (DOR = 28.8 (95%CI:5.6-149.2), p = 0.000001), no fever (DOR = 37.3 (95%CI:7.2-192.8), p = 0.0000001), no rash (DOR = 39.8 (95%CI:8.4-188.5), p = 0.0000001), night bone pain (DOR = 2364.3 (95%CI:92.9-60169.9), p = 0.0000001), any bone and joint pain (DOR = 227.6 (95%CI: 12.5-4158), p = 0.0000001), pathologic fractures (DOR = 32.7 (95%CI:1.6-661.0), p = 0.0004).

**Table:** 

Parameters	HOD (n = 21)	SJIA (n = 86)	p
WBC, x10^9^/l	7.4 (5.3-8.3)	14.6 (9.3-18.5)	0.002
PLT, x10^9^/l	175 (109.5-249)	436 (256-583)	0.00001
CRP, mg/l	12.3 (5.9-48.8)	57 (18-113)	0.005
Active joints, n	1 (1-3)	4 (2-13)	0.006
Fever, n (%)	11/21 (52.4)	82/84 (92.6)	0.0000001
Rash, n (%)	2/21 (9.5)	67/83 (80.7)	0.0000001
Night pains, n (%)	12/21 (57.1)	0/86 (0)	0.0000001
Bone pains, n (%)	20/21 (95.2)	0/86 (0)	0.0000001
Pathological fractures, n (%)	3/21 (14.3)	0/86 (0)	0.0004

**Conclusion:** The found diagnostic criteria can help physicians to discriminate HOD patients with “rheumatic masks” into the group of patients with suspected SoJIA.

**Disclosure of Interest**: None Declared

### P420 The ability of tocilizumab induces the remission in systemic juvenile idiopathic arthritis: the main predictors

#### Mikhail M. Kostik, Irina Chikova, Eugenia Isupova, Margarita Dubko, Vera Masalova, Ludmila Snegireva, Tatyana Kornishina, Tatyana Likhacheva, Olga Kalashnikova, Vyacheslav Chasnyk

##### Hospital Pediatry, Saint-Petersburg State Pediatric Medical University, Saint-Petersburg, Russian Federation

###### **Presenting author:** Mikhail M. Kostik

**Introduction:** Systemic juvenile idiopathic arthritis (SJIA) is one of the most striking forms of juvenile arthritis, required biologic administration due to failure of corticosteroids (CS) and DMARDs. Currently there are two strategies in treatment SJIA with biologic: blockade of IL-1 and IL-6. Despite similar efficacy and safety profile in latest ACR recommendations blockade of IL-6 recognized as second-line biologic treatment after using the anakinra. The question about first line biologic treatment in SJIA is still open.

**Objectives:** The aim of our study was to evaluate outcomes and find predictors of remission in SJIA children, receiving TCZ therapy.

**Methods:** Our retrospective study was included 48 active SJIA children who fall CS, methotrexate (MTX), cyclosporine A (CsA) and their combination in whom TCZ was initiated in dosage 12 mg/kg if weight was < 30 kg and 8 mg/kg if weight ≥ 30 kg. The duration of study was limited the 1^st^ and last TCZ infusions. We evaluated clinical and laboratorial signs, attributed to SJIA, such as presence of fever, hepatosplenomegaly, serositis, rash, lymphadenopathy, active joints, the levels of Hb, WBC, PLT, granulocytes, LDH, and macrophage activation syndrome (MAS). We check the granulocytes levels throw 1 and 2 after 1^st^ TCZ infusion. The efficacy of TCZ was measured throw changes of SJIA attributed signs, symptoms, dynamic of concomitant treatment and achievement the remission according to C. Wallace (2004) criteria.

**Results:** The main demographic parameters (Me; IQR) included the age-9.9 (5.-12.7) years and delay of TCZ-27.0 (5.9-89.7) months. The treatment before TCZ included CS-38 (79.2%), MTX-40 (83.3%), CsA-18 (37.5%) and their combination. The macrophage activation syndrome (MAS) in past medical history before TCZ was in 14 (29.2%).

During the trial CS successfully discontinued 25 (65.8), CsA 8/18 (44.4%), MTX 12/40 (30.0%) patients. In 7 children TCZ was discontinued due to stable remission with median duration 640 days. After TCZ initiation 6 children have experienced MAS, but all of them had MAS before TCZ, so no “new cases” were observed on TCZ. 5 children early withdrew during the trial due to adverse events (infusion reaction, MAS) and 2 children died (1 severe uncontrolled MAS, 1 amyloidosis).

During the TCZ treatment 40 (83.3%) achieved the remission in 138.5 (56.0; 255.0) days. Patients, who achieved remission had milder disease course, presented in less frequent hepatosplenomegaly, lung, heart and CNS involvement, hemorrhagic syndrome and MAS. They had higher Hb (p = 0.02) and lower WBC (p = 0.048), granulocytes (p = 0.015), ESR (p = 0.034), CRP (p = 0.05), LDH (p = 0.0003), ferritin (p = 0.0007). The main predictors of achievement inactive disease, calculated with Cox-regression models, presented in the table.

**Conclusions:** We found clinical and laboratorial criteria for SoJIA remission during the tocilizumab treatment.

**Table:** 

Parameters	**OR (95% CI)**	**p**	**HR**	**p**
CRP ≤ 82.0 mg/l**	7,9 (1,4-45,3)	0,016*	1,17	0,66
ESR ≤ 32 mm/h**	17,0 (0,9-314,3)	0,014*	0,85	0,62
Ferritin ≤ 273ng/ml**	56,5 (2,8-1124,9)	0,0001*	2,6	**0,02**
LDH ≤ 676 U/l**	113,6 (5,3-2451,8)	0,000014*	3,18	**0,029**
PLT > 335x109/l**	5,0 (0,9-28,9)	0,11*	2,54	**0,007**
Age of 1^st^ TCZ infusion ≤ 11y.**	2,6 (0,6-12,4)	0,24*	1,44	0,3
Decreaed WBC in 2 weeks > 11%**	13,0 (1,4-124,3)	0,03*	6,03	**0,019**
Decreased Granulocytes in2 weeks > 12%**	14,0(1,1-185,5)	0,05*	4,7	0,13
MAS before TCZ	0,17 (0,04-0,87)	0,037*	0,7	0,34

Me (IQR), * Fisher’s exact test, ** AUC – area under the curve

**Conclusion:** We found clinical and laboratorial criteria for SoJIA remission during the tocilizumab treatment.

**Disclosure of Interest**: None Declared

### P421 Efficacy and safety of canakinumab in patients with systemic juvenile idiopathic arthritis: results from an open-label long-term follow-up study

#### N. Ruperto^1^, H. I. Brunner^2^, P. Quartier^3^, T. Constantin^1^, E. Alexeeva^1^, R. Schneider^2^, I. Kone-Paut^1^, K. Schikler^2^, K. Marzan^2^, N. Wulffraat^1^, S. Padeh^1^, V. Chasnyk^1^, C. Wouters^1^, J. B. Kuemmerle-Deschner^1^, T. Kallinich^1^, B. Lauwerys^4^, E. Haddad^2^, E. Nasonov^1^, M. Trachana^1^, O. Vougiouka^1^, K. Leon^5^, A. Speziale^6^, K. Lheritier^6^, E. Vritzali^6^, A. Martini^1^, D. Lovell^2^ and PRINTO/PRCSG

##### ^1^PRINTO-Istituto Gaslini, Genoa, Italy; ^2^PRCSG, Cincinnati, OH, United States; ^3^Necker-Enfants Malades Hospital, Paris, France; ^4^Cliniques Universitaires Saint-Luc and Université Catholique de Louvain, Brussels, Belgium; ^5^Novartis Pharmaceuticals Corporation, East Hanover, NJ, United States; ^6^Novartis Pharma AG, Basel, Switzerland

###### **Presenting author:** N. Ruperto

**Introduction:** Canakinumab (CAN), a highly selective human anti-IL1 β monoclonal antibody, had demonstrated its efficacy and safety in patients (pts) with active systemic juvenile idiopathic arthritis (SJIA) in a comprehensive global clinical program consisting of one phase II and two phase III trials.^1,2^ However, limited data was available on long-term efficacy and safety of CAN in SJIA.

**Objectives:** To assess long-term efficacy and safety of CAN treated SJIA pts over a 5-year (yr) follow-up observational period.

**Methods:** This was an open-label extension (OLE) study of SJIA pts participating in the global clinical trials of CAN.^3^ Pts, 2 to <20 yrs of age at the time of enrollment in study, received subcutaneous CAN 4 mg/kg every 4 weeks. Baseline was defined as the starting point of the extension trial. Efficacy assessments were done every 3 months, including adapted paediatric response criteria (aACR), clinical inactive disease and clinical remission on medication (continuous 12 months of clinical inactive disease). Safety assessments included adverse events (AEs) and serious AEs (SAEs).

**Results:** Overall, 147 pts to the OLE study had a median treatment duration of 3.2 yrs; total treatment exposure was approximately 365 pt-yrs. Of 147 pts, 100 (68%) completed 96 weeks of treatment, whereas 47 (32%) pts discontinued the study. Another 25 pts (17%) discontinued the study after Week 96. Of the 107 pts with an aACR 30 at entry to the OLE study, 61.7%, 79.4% and 86.0% have had aACR 100, 90 and 70 responses, respectively at last assessment. At baseline, 32.7% of patients were with inactive disease which increased up to 60% - 70% between Week 36 and Week 168. Clinical remission on medication was achieved in 43% pts. In total, 137 (93.2%) pts reported at least 1 AE during the 3.2 years median exposure in the study corresponding to 2.009 AEs/100 pt-days (733.6 AEs/100 pt-years) with infections (202.7 per 100 pt-years) being the most common AE. Overall, 47 (32.0%) pts had at least 1 SAE corresponding to 0.089 SAE/100 pt-days (32.6 SAE/100 pt-years) with the most common being JIA (14 pts) denoting disease flares or worsening of SJIA. Ten patients (6.8%) with a total of 12 MAS events were reported as SAE and 7 patients among them discontinued the study. No deaths were reported.

**Conclusion:** In patients previously treated with CAN in pivotal trials, response to treatment was sustained or improved during long-term treatment in the OLE study. Safety profile of CAN was consistent with safety findings from previous studies.

**References:**

1. Ruperto N, et al. *N Engl J Med*. 2012;367(25):2396-406.

2. Ringold S, et al. *Arthritis & Rheum*. 2013;65(10):2499-512.

3. Ruperto N, et al. *Ann Rheum Dis*. 2015;74(2):608.

**Trial registration identifying number:** NCT00891046

**Disclosure of Interest**: N. Ruperto Grant / Research Support from: Abbott, BMS, “Francesco Angelini”, GlaxoSmithKline (GSK), Hoffman-La Roche, Italfarmaco, Janssen, Novartis, Pfizer, Sanofi Aventis, Schwarz Biosciences, Sobi, Xoma and Wyeth, Speaker Bureau of: Abbott, AbbVie, Amgen, Biogenidec, Astellas, Alter, AstraZeneca, Boehringer, BMS, CDPharma, Celgene, CrescendoBio, EMD Serono,Hoffman-La Roche, Italfarmaco, Janssen, MedImmune, Medac, Novartis, Novo Nordisk, Pfizer, Sanofi Aventis, Servier, Takeda and Vertex, H. Brunner Consultant for: Novartis, Roche, Pfizer, UCB, Celgene, Regeneron, Amgen, Astrazeneca, GSK and BMS, Speaker Bureau of: Novartis and Roche, P. Quartier Grant / Research Support from: Abbvie, BMS, Chugai-Roche, Novartis, Pfizer and SOBI, Consultant for: Abbvie, Novartis, Servier and SOBI, Speaker Bureau of: Abbvie, BMS, Chugai-Roche, Medimmune, Novartis, Pfizer and SOBI, T. Constantin: None Declared, E. Alexeeva Grant / Research Support from: Roche, Abbott, Novartis, Pfizer, Bristol-Myers Squibb and Centocor, Speaker Bureau of: Roche, Novartis, Merck Sharp Dohme, Bristol-Myers Squibb, Medac and Pfizer, R. Schneider Consultant for: Novartis, Novimmune, Sobi and Innomar Strategies, I. Kone-Paut Grant / Research Support from: SOBI, Novartis and Roche, Consultant for: Novartis, SOBI, Pfizer, Abbvie and Roche/Chugai, K. Schikler Consultant for: Novartis, Novimmune, SOBI and Innomar Strategies, K. Marzan Grant / Research Support from: Novartis, Abbvie, N. Wulffraat Grant / Research Support from: Novartis, S. Padeh: None Declared, V. Chasnyk: None Declared, C. Wouters Grant / Research Support from: GSK, Novartis and Roche, J. Kuemmerle-Deschner Grant / Research Support from: Novartis, Consultant for: Novartis, SOBI and Baxalta, T. Kallinich Grant / Research Support from: Novartis, Speaker Bureau of: SOBI, Novartis, BMS and Roche, B. Lauwerys: None Declared, E. Haddad: None Declared, E. Nasonov: None Declared, M. Trachana Grant / Research Support from: Novartis, Abbvie, Bristol Meyers, Consultant for: Novartis, Roche and Pfizer, O. Vougiouka: None Declared, K. Leon Employee of: Novartis, A. Speziale Employee of: Novartis, K. Lheritier Shareholder of: Novartis, Employee of: Novartis, E. Vritzali Employee of: Novartis, A. Martini Grant / Research Support from: Abbott, BMS, “Francesco Angelini”, GlaxoSmithKline (GSK), Hoffman-La Roche, Italfarmaco, Janssen, Novartis, Pfizer, Sanofi Aventis, Schwarz Biosciences, Sobi, Xoma and Wyeth, Speaker Bureau of: Abbott, Abbvie, Amgen, Biogenidecm Bristol MyersSquibb, Astellas, Behringer, Italfarmaco, Janssen, MedImmune, Novartis, NovoNordisk, Pfizer, Sanofi, Roche, Servier and Takeda, D. Lovell Grant / Research Support from: National Institutes of Health, Consultant for: Astra-Zeneca, Bristol Meyers Squibb, AbbVee, Pfizer, Roche, Novartis, UCB, Forest Research Institute, Horizon, Johnson & Johnson, Biogen, Takeda, Genentech, Glaxo Smith Kline, Boehringer Ingelheim, Celgene and Jannsen, Speaker Bureau of: Genentech, Roche and Novartis

### P422 Phagocyte involvement in systemic onset juvenile idiopathic arthritis

#### Nienke Ter Haar^1,2^, Rianne Scholman^1^, Wilco de Jager^1^, Tamar Tak^1^, Pieter Leliefeld^1^, Bas Vastert^2^, Sytze de Roock^1,2^

##### ^1^Laboratory for Translational Immunology, University Medical Center Utrecht, Utrecht, Netherlands; ^2^Pediatric Rheumatology, University Medical Center Utrecht, Utrecht, Netherlands

###### **Presenting author:** Nienke Ter Haar

**Introduction:** Systemic onset Juvenile Idiopathic Artritis (sJIA) is a systemic autoinflammatory disease, characterized by arthritis, spiking fever and rash and elevation of serum S100-proteins and interleukin(IL)-18, reflecting IL-1 pathway activation. The exact role of monocytes and neutrophils in the inflammatory cascade of sJIA is still to be unravelled.

**Objectives:** To dissect the role of monocytes and neutrophils in the inflammatory cascade of sJIA.

**Methods:** In a prospectively followed cohort of 23 new-onset sJIA patients, we evaluated cell counts, serum levels of cytokines, chemokines and other analytes at disease onset and during inactive disease. Cytokine concentrations in supernatant and serum were determined by multiplex immunoassay. We determined neutrophil activation *ex vivo* (phenotype and cell membrane markers) and after stimulation (ROS-production and degranulation) of cells derived from sJIA with active disease (n = 10) or in clinically inactive disease (n = 6), compared to healthy donors (HDs, n = 16). To investigate the role of monocytes, we assessed cytokine production of peripheral blood derived mononuclear cells (PBMC) from active and remission sJIA patients and HDs (n = 6 for all groups) after stimulation with TLR-4 activating S100-proteins (+/- ATP) or other TLR-ligands.

**Results:** Twenty-one of 23 patients with onset sJIA had elevated neutrophil counts, while monocyte counts were elevated in only 5/23 patients. Among the inflammatory markers that were significantly elevated in serum of onset sJIA patients, we observed multiple neutrophil specific proteins like elastase and neutrophil collagenase, indicating the importance of this cell type.

Neutrophils from active sJIA patients showed an activated phenotype, reflected by higher *ex vivo* cell membrane expression of FC-gamma receptors (CD32 and CD64), markers of secretory vesicles (CD35) and specific granules (CD66b). Further, on cytospins of acute onset patients, this hyperactivated state was confirmed, including granulocyte precursors, vacuolization and toxic granules. ROS production and degranulation were also enhanced in active sJIA, both with medium control and after short *in vitro* stimulation. This activated phenotype was most prominent in patients with a short, fever-dominant disease, while it was less prominent in patients with >1 month active disease, or with a more articular phenotype at disease onset. Neutrophil phenotype normalized when patients improved to clinically inactive disease.

In contrast to the hyperactivated status of neutrophils in active sJIA, PBMC from these patients produced less Il-1b, IL-18, IL-6 and TNF-a upon TLR-stimulation compared to PBMC from patients with clinically inactive disease or HDs, suggesting tolerance after exposure to high TLR4 stimulating S100-levels *in vivo*. RNA-sequencing of FACS sorted monocytes *ex vivo* and after LPS stimulation is currently undertaken and will reveal pathways involved in this ‘tolerance’ phenotype.

**Conclusion:** We show here that monocytes from active new-onset sJIA patients produce less cytokines upon TLR stimulation, while the neutrophils are hyperactivated, reflected by increased cell membrane activation markers, ROS production and degranulation. The exact role of both cell types in relation to the increased S100 protein levels and the IL-1 / IL-18 activaion is currently under investigation.

**Disclosure of Interest**: None Declared

### P423 Biomarkers in systemic onset juvenile idiopathic arthritis: prediction of therapy response

#### Nienke Ter Haar^1,2^, Rianne Scholman^1^, Wilco de Jager^1^, Ariane de Ganck^3^, Nadia Ryter^4^, Miha Lavric^5^, Dirk Foell^5^, Sytze de Roock^1,2^, Bas Vastert^2^

##### ^1^Laboratory for Translational Immunology, Utrecht, Netherlands; ^2^Pediatric Rheumatology, UMCU, Utrecht, Netherlands; ^3^Biogazelle NV, Zwijnaarde, Belgium; ^4^BÜHLMANN Laboratories AG, Basel, Switzerland; ^5^Pediatric Rheumatology and Immunology, University of Muenster, Muenster, Germany

###### **Presenting author:** Nienke Ter Haar

**Introduction:** Systemic onset juvenile idiopathic arthritis (sJIA) is an autoinflammatory disease, characterized by fever, rash and arthritis. The IL-1 pathway and IL-6 pathway are crucial disease mechanisms in sJIA, exemplified by the excellent responses to IL-1 and IL-6 blocking therapy. However, so far it has not been feasible to base treatment choice on the individual inflammatory characteristics of a patient. In addition, we cannot predict in which patients we can safely stop therapy when inactive disease is reached.

**Objectives:** We aim to find biomarkers to predict: which sJIA patients will respond to IL-1 blockade at disease onset, and which patients will maintain clinical remission when IL-1 blockade is stopped after reaching clinically inactive disease.

**Methods:** In our center, patients are treated with recombinant human IL-1 receptor antagonist (rhIL1RA) as first-line therapy. If the response to rhIL1RA is insufficient, either low dose corticosteroids are added or the patients are switched to alternative biologicals. If patients are in inactive disease at time point three months after start of rhIL1RA, we attempt to taper and stop rhIL1RA.

Biomarker discovery was performed in the serum of patients at onset (before start of rhIL1RA) and at time point 3 months in patients with clinically inactive disease on rhIL1RA, using cytokine analysis (Luminex multiplex assay), determination of S100A12 and Mrp8/14 (ELISA) and miRNAs (qPCR). To determine differences between the groups, we used the Mann-Whitney U test, with false discovery rate (FDR) correction by Benjamini-Hochberg methodology.

**Results:** In total, serum of 19 patients at disease onset and 20 patients at inactive disease was available. Fifty-five proteins and 10 miRNAs were significantly different between patients at onset and at inactive disease after FDR-correction. Proteins included known biomarkers as S100A12, Mrp8/14, IL-6 and IL-18, but also novel markers including metalloproteinases and angiogenesis-related proteins.

Within the onset patients, thirteen patients had a complete response with rhIL1RA monotherapy (GR), while five patients needed additional therapy/switch of therapy (NR) because of persistent fever (n = 2), arthritis (n = 2) or both fever and arthritis (n = 1). One patient had a partial response on rhIL1RA monotherapy. On univariate (uncorrected) testing, 3 proteins and 35 miRNAs were significantly different between GR and NR. After FDR-correction none remained significant, which can be explained by the low number of patients, especially in the NR group.

In the stop-prediction part of this study, ten patients flared during tapering or after stopping of therapy, while nine patients remained in remission. One patient preferred to continue rhIL1RA and could therefore not be categorized in a response group. The PCA of the Luminex data showed that patients that will flare cluster separately from patients that will maintain clinical remission. On univariate analysis, 16 proteins and 2 miRNAs were significantly different, however none were significant after FDR correction.

**Conclusion:** This study aimed to find novel biomarkers for the prediction of treatment response to rhIL1RA in new-onset sJIA patients. Although no markers were significant after FDR correction, the results still provide potentially interesting biomarkers that need further analysis and modeling. A soon-to-start prospective study on optimisation of stop-strategy of rhIL1RA, will provide the opportunity to validate the predictive value of these biomarkers.

**Disclosure of Interest**: None Declared

### P424 Impact of systemic juvenile idiopathic arthritis/Still’s disease on adolescents as evidenced through social media posting

#### Renee F. Modica^1^, Kathleen G. Lomax^2^, Pamela Batzel^3^, Armelle Cassanas^3^, Melissa E. Elder^1^

##### ^1^Department of Pediatrics, University of Florida, Gainesville, FL, United States; ^2^Immunology and Dermatology Medical Affairs, Novartis Pharmaceuticals Corp, East Hanover, NJ, United States; ^3^Treato, Princeton, NJ, United States

###### **Presenting author:** Renee F. Modica

**Introduction:** Systemic juvenile idiopathic arthritis (SJIA)/Still’s disease is a rare form of chronic arthritis in paediatrics. The patient perspective of living with the disease is not well understood, particularly among adolescent age patients.

**Objectives:** The objective was to understand the adolescent SJIA experience as shown by their own social media posts.

**Methods:** English posts from SJIA patients were reviewed on public social media sites.

**Results:** 71 posts with a date range of 2009-2015 on 15 sites were reviewed in Nov 2015. 24 unique authors were identified: 17 SJIA patients (40 posts) and 7 mothers of SJIA patients (12 posts). Patients were aged 13-20 years. Several patients posted about similar diagnostic experiences marked by 5 stages: (1) misunderstood with their pain and fatigue being overlooked until a crisis occurs, (2) dismissed as ‘fakers,’ where their initial misdiagnosis is often ‘growing pains’ or ‘fake pains’, (3) misdiagnosis, often as cancer, when the symptoms acutely worsen (4) testing stage that leads to an SJIA diagnosis, and (5) focus on the difficulties of dealing with a chronic invisible disease where they feel ashamed of their arthritis and distressed at being different from their peers. Many adolescent patients, looking back at the onset of the disease when they were children, describe themselves as a “sleeping child” rather than the typical active, playing child. Patients describe trying to hide their illness from friends, but express their concerns more openly online. Patients also describe anger directed at SJIA which is described as a powerful external enemy attacking their body, using terms like “bulldozer,” “dragon,” and “monster.” Many posters used superhero language or imagery in their social media posts to help them “fight” the disease and their struggle. Mothers of SJIA patients also used warrior-child imagery and language in their posts. Some SJIA patients also posted about the risk of death, or shared stories about other SJIA patients who died which is a distinct difference from non-SJIA patients. Many patients also have adopted the term “spoonie” to describe themselves as living with a chronic disease, a term that originated in the autoimmune community to refer to how people with chronic conditions manage their energy throughout the day. Only the older teenagers used the term Still’s.

**Conclusion:** Adolescent SJIA patients posted openly about the difficulties of their disease causing them to be different from their healthy friends, whereas in the real world they tried to minimise or hide the effects of their disease. They frequently used superhero words and images in posts in describing their fight for health. Physicians can use these insights when counselling adolescent SJIA patients to provide a narrative that meshes with the patients’ worldview and perhaps, by speaking a similar language, could increase treatment adherence.

**Disclosure of Interest**: R. Modica: None Declared, K. Lomax Employee of: Novartis, P. Batzel: None Declared, A. Cassanas: None Declared, M. Elder: None Declared

### P425 Influence of tocilizumab treatment on bone mineral density in patients with systemic juvenile idiopathic arthritis

#### Rina Denisova^1^, Ekaterina Alexeeva^1^, Saniya Valieva^1^, Tatyana Bzarova^1^, Kseniya Isayeva^1^, Tatyana Sleptsova^1^, Olga Lomakina^1^, Alexandra Chomahidze^1^, Margarita Soloshenko^1^, Meyry Shingarova^2^, Elena Kachshenko^1^

##### ^1^Rheumatology, Scientific Center of Children’s Health, Moscow, Russian Federation; ^2^I.M. Sechenov First Moscow State Medical University, Moscow, Russian Federation

###### **Presenting author:** Rina Denisova

**Introduction:** The progression of destructive changes in the joints and development of bone resorption in rheumatic diseases are closely pathogenetic relationship. An important factor in the risk of osteoporosis is a glucocorticosteroid therapy (GC). The issue of drug therapy in patients with these complications are still relevant.

**Objectives:** To investigate the influence of tocilizumab therapy on bone mineral density in patients with systemic JIA option.

**Methods:** A retrospective analysis of 49 medical records of patients diagnosed with systemic JIA observed in the rheumatology department in one center, 25 boys and 24 girls, mean age 14 years (7; 21), the average age of disease onset - 4 years (1; 14).

**Results:** The high disease activity was observed in 100% of children, lack of physical activity in 23/49 (47%), fractures in 5/59 (11%). Before tocilizumab treatment 36/49 (73%)patients received oral GC, 32/49 (65%) patients pulse therapy of methylprednisolone. Status of bone mineral density (BMD) of the lumbar spine (L1-L4) according to densitometry were observed: osteoporosis (reduction of Z-score > -2) -in 15/49 (30%) children, osteopenia (Z-score between -1 and -2) - in 14/49 (29%), normal values (Z-score, <-1) - in 20/49 (41%). After a year of tocilizumab treatment osteoporosis was registered in 8/49 (16%) children,osteopenia - in 13/49 (27%), and normal values - in 28/49 (57%). The analysis of the effect of treatment with monoclonal antibodies to IL-6 on BMD in children with systemic JIA showed significant (p <0.001) increase in indicators Z-score and BMD.

**Conclusion:** The analysis of the effect of treatment with monoclonal antibodies to IL-6 on BMD in children with systemic JIA showed significant (p <0.001) increase in indicators Z-score and BMD. The results indicate a decrease in the degree of severity of osteoporosis.

**Disclosure of Interest**: None Declared

### P426 Recombinant IL-1RA restores the IL-18-NK cell axis in steroid naïve systemic juvenile idiopathic arthritis patients

#### Wilco De Jager, Sebastiaan J. Vastert, Gerdien Mijnheer, Berent J. Prakken, Nico M. Wulffraat

##### Pediatric Immunology, University Medical Center Utrecht, Utrecht, Netherlands

###### **Presenting author:** Wilco De Jager

**Introduction:** Systemic onset juvenile idiopathic arthritis (sJIA) is a severe, acquired IL-1 mediated auto-inflammatory disease. However, not all disease manifestations can be fully explained by IL-1 activation and other cytokines, like IL-6 and especially IL-18 are strikingly elevated at disease onset. We previously showed that NK cell activation by IL-18 is disturbed in sJIA patients due to defective phosphorylation of the IL-18 receptor beta chain. However, the exact role of IL-18 in the immune pathogenesis and disease manifestations of sJIA is still unclear.

**Objectives:** To evaluate both clinical and immunological effects of anakinra treatment prior to steroids in newly diagnosed SoJIA patients.

**Methods:** Twenty consecutive patients with newly diagnosed systemic onset JIA were included. Clinical response was evaluated using the validated core set parameters for JIA. Disease activity was given as percentages improvement compared to baseline values (ACR-Ped scores). Biochemical parameters of disease activity (ESR, CRP, Ferritin, NK cell function, sIL-2R) were collected at standardized timepoints. IL-18 interactions were studied using the IL-18 sensitive myelomonocytic cellline (KG-1) using westernblotting and immunoprecipitation. Furthermore we characterized the response to anakinra evaluating cytokine profiles, NK cell phenotype and function, Caspase 1 activity.

**Results:** We show that sJIA patients have increased inflammasome activation contributing to elevated IL-18 levels. Treatment with rIL-1RA effectively down-regulated IL-18 levels through suppression of inflammasome activation both *in vitro* and *in vivo*. Furthermore, in sJIA patients treated early in their disease course with rIL-1RA, we observed a restoration of the defective IL-18-NK cell axis within 3 weeks of treatment with rIL-1RA. *In vitro* experiments, using a human myelomonocytic IL-18 reporter cell line as well as healthy control monocytes, showed direct binding of rIL-1RA to the IL-18 receptor alpha unit.

**Conclusion:** rIL-1RA is able to target both the IL-1 and IL-18 pathway, explaining the complete therapy response in most sJIA patients when used early in the disease course.

**Disclosure of Interest**: None Declared

## Poster Session: Systemic lupus erythematosus and antiphospholipid syndrome II

### P427 Gastrointestinal symptoms in juvenile systemic lupus erythematosus patients

#### Hafize E. Sönmez^1^, Asuman N. Karhan^2^, Ezgi D. Batu^1^, Yelda Bilginer^1^, Zehra S. Arıcı^1^, Ersin Gümüş^2^, Hülya Demir^2^, Aysel Yüce^2^, Seza Özen^1^

##### ^1^Department of Pediatrics, Division of Rheumatology, Hacettepe University Faculty Of Medicine, Ankara, Turkey; ^2^Department of Pediatrics, Division of Gastroenterology and Hepatology, Hacettepe University Faculty Of Medicine, Ankara, Turkey

###### **Presenting author:** Hafize E. Sönmez

**Introduction:** Systemic lupus erythematosus (SLE) is an autoimmune disease with multiple organ involvement. SLE is affecting 10-20% of patients in childhood or puberty. Atypical presentations may occur in the childhood such as gastrointestinal system (GIS) involvement being present in the early stages of the disease. The GIS involvement in the SLE can occur as an assosiation, as a disease manifestation or as a result of the advers effects of drugs.

**Objectives:** We aimed to present GIS symptoms of the juvenile SLE (jSLE) patients followed in our department.

**Methods:** We have reviewed the medical files of 69 children with SLE who were followed at Department of Pediatric Rheumatology in Hacettepe University between January 2011 and January 2016. All patients were fulfilling the Systemic Lupus International Collaborating Clinics criteria. All SLE patients (≤18 years of age) who had gastrointestinal system manifestations were included in study.

**Results:** GIS manifestations were observed in 19 (27.5%) out of 69 SLE patients and present at the time of SLE diagnosis in 13 (68.4%) patients. There was no significant difference in clinical or serological features between patients with and without GIS involvement. The median (min-max) age of SLE diagnosis was 120 (60-192) months; the age of the onset of the GIS symptoms was 156 (60-204) months. The median time from the SLE diagnosis to the onset of the GIS symptoms was 0 (0-96) months. The SLE GIS manifestations were autoimmune hepatitis (AIH) (n = 8) and lupus enteritis (n = 1). SLE associations were hepatomegaly and hypertransaminasemia associated with macrophage activation syndrome (MAS) (n = 3) and hepatic steatosis (n = 1). GIS manifestations as a result of the adverse events of drugs were as follows: toxic hepatitis (n = 3; associated with methotrexate and nonsteroidal anti-inflammatory drugs in one patient, methotrexate in another patient, and azathioprine in another patient), azathioprine induced cholestatic hepatitis (n = 1), and gastritis associated with corticosteroid (n = 1). In one patient, acute appendicitis occurred as a co-incidental manifestation. At the time of presentation with GIS symptoms, the median (min-max) erythrocyte sedimentation rate was 40 (4-81) mm/hour and SLE activity index was 8 (4-26). The median AIH score of the AIH patients was 18 (15-22).

For treatment, the drug of interest was discontinued for the patients with drug-related hepatitis. Seven out of eight patients with AIH received corticosteroids and azathioprine for treatment. One of these patients received ursodeoxycholic acid in addition to the immunosuppressive treatment. One AIH patient was treated with corticosteroid. Two patients with MAS received corticosteroid and cyclosporine treatment while one got corticosteroid and intravenous immunoglobulin. Corticosteroid was given to the patients with lupus hepatitis and lupus enteritis. Proton pump inhibitor was prescribed to the patient with gastritis. Dietary modifications and weight control were advised to the patient with hepatosteatosis. The GIS manifestations improved in all patients with treatment.

**Conclusion:** In this study, we have demonstrated that one out of every five JSLE patients of ours had GIS-related signs and symptoms during SLE course and GIS involvement may occur as an initial manifestation of the disease. Thus, it is important to consider SLE in the differential diagnosis of GIS manifestations in children. Furthermore, the criteria for SLE diagnosis may include GIS involvement since it is probably more common than anticipated.

**Disclosure of Interest**: None Declared

### P428 Reference ranges for the antiphospholipid antibodies in children are different from those in adults- the North Indian experience

#### Jasmina Ahluwalia^1^, Bhavneet Bharti^2^, Sweta Rajpal^1^, Varun Uppal^1^, Alaknanda Walia^1^, Surjit S. Samlok^2^, Narender Kumar^1^

##### ^1^Hematology, Chandigarh, India; ^2^Pediatrics, Postgraduate Institute of Medical Education and Research, Chandigarh, India

###### **Presenting author:** Jasmina Ahluwalia

**Introduction:** Antiphospholipid antibody syndrome (APS) is an autoimmune disorder characterised by thrombosis and /or recurrent pregnancy losses and positivity for the lupus anticoagulant (LA), anticardiolipin (ACA) or anti beta 2 glycoprotein 1 (aβ_2_GP1) antibodies. Current guidelines recommend that values more than the 99th centile of the antibody levels found in the normal population be used to classify positivity for APS. APS has been reported in children. Pediatric controls subjects are difficult to obtain and laboratories may have to use the adult cut off values to report test positivity.

**Objectives:** We screened healthy children and adult volunteers in Chandigarh, India to compare the reference values of the 99^th^centiles of ACA and aβ_2_GP1 antibodies.

**Methods:** Children aged between 1.5 months and 18 years and adults between 19 and 60 years were screened once for the IgG and IgM isotypes of ACA and aβ_2_GP1 antibodies by ELISA kits (Orgentec GmBh). Subjects with history of recent illness or drug intake in the preceding 15 days were excluded from sampling The pediatric samples were grouped as: Group I-aged <1year; II-1 to 5 years, III -6 to 10 years and IV- >10 years old. The 99^th^ centiles for the two populations and 4 groups in the children were determined and compared.

**Results:** 129 children and 144 adults were screened. Three children and 2 adults were excluded from analysis since they were positive for more than 1 antibody**.** Data for 126 children (71 males, 55 females) and 142 adults (88 males, 54 females) were available for analysis. There were 27, 33, 27 and 39 children in groups I, II,III and IV respectively. The data distribution for levels of all isotypes tested was skewed. The 99^th^ centile values in children vs adults were as follows ACA IgG-8.22 vs.15.41; for ACA IgM -3.2 vs. 6.32; aβ2GP1 IgG-18 vs. 4.39; aβ_2_GP1 IgM-8.75 vs. 5.94. The group wise 99th centile values for the antibodies are depicted in the table.

**Table:** 

		**99 th Centile values**			
**Subject group**		**ACA IgG**	**ACA IgM**	**aβ**_**2**_ **GP1 IgG**	**aβ**_**2**_ **GP 11 IgM**
Adults	(n = 142)	15.41	6.32	4.39	5.94
Pediatric controls	n = 126)	8.22	3.2	18	8.75
Pediatric Group I (<1 year)	(n = 27)	8.78	1.53	16.6	2.57
Pediatric Group II (1-5 years)	(n = 33)	7.03	2.58	24.71	7.23
Pediatric Group III (6 -10 years)	(n = 27)	3.03	1.35	5.05	6.51
Pediatric Group IV (11-18 years)	(n = 39)	5.16	3.99	4.34	12.16

**Conclusion:** The 99^th^ centile values for adults and children differed significantly, with adults exhibiting higher cut offs for ACA isotypes, whereas for aβ_2_GP1 antibodies the cut off was higher among children. The IgG isotype cut off levels were higher than the IgM isotypes for ACA, though such a pattern was not seen with aβ_2_GP1. The IgG isotypes of both ACA and aβ_2_GP1 antibodies were highest in infants and showed a decreasing trend with increasing age. A variable pattern was observed for IgM isotype of aβ_2_GP1 antibodies. Despite the limitations of single determinations and using only one commercial kit, the data support the fact that determining reference ranges for the APS antibodies in children is important to avoid potential misclassification of APS. With the widespread use of commercial ELISA kits for establishing APS in children, provision of pediatric reference ranges by the kit manufacturers would be invaluable for small laboratories with limited access to pediatric controls.

**Disclosure of Interest**: None Declared

### P429 Anti-ribosomal P antibody: a multicenter study of 228 childhood-onset systemic lupus erythematousus patients

#### Clarissa C. Valões^1^, Beatriz C. Molinari^1^, Ana Claudia G. Pitta^1^, Natali W. Gormezano^2^, Sylvia C. Farhat^1^, Kátia Kozu^1^, Adriana M. Sallum^1^, Simone Appenzeller^3^, Ana Paula Sakamoto^4^, Maria T. Terreri^4^, Rosa M. Pereira^2^, Claudia S. Magalhães^5^, Cássia Maria Barbosa^6^, Francisco Hugo Gomes^7^, Eloisa Bonfá^2^, Clovis A. Silva^1^

##### ^1^Pediatric Rheumatology Unit, Faculdade de Medicina d Universidade de São Paulo, São Paulo – SP, Brazil; ^2^Pediatric Rheumatology Division, Faculdade de Medicina d Universidade de São Paulo, São Paulo – SP, Brazil; ^3^Pediatric Rheumatology Unit, UNICAMP, Campinas-SP, Brazil; ^4^Pediatric Rheumatology Unit, UNIFESP, São Paulo – SP, Brazil; ^5^Pediatric Rheumatology Unit, UNESP, Botucatu-SP, Brazil; ^6^Pediatric Rheumatology Unit, Hospital Darcy Vargas, São Paulo – SP, Brazil; ^7^Pediatric Rheumatology Unit, USP-Ribeirão Preto, Ribeirão Preto, SP, Brazil

###### **Presenting author:** Kátia Kozu

**Introduction:** Anti-ribosomal P (anti-P) antibody is highly specific for systemic lupus erythematosus (SLE) and recognizes three ribosomal phosphoproteins. Evaluation of this autoantibody in childhood-onset SLE (cSLE) populations is limited to few small series hampering the interpretation of the diverse standardized 19 neuropsychiatric syndromes case definition for this specificity.

**Objectives:** The objective of this multicenter cohort study was to evaluate demographic, clinical and laboratorial features in cSLE patients with and without the presence of anti-P antibody.

**Methods:** This is a retrospective multicenter study performed in 10 Pediatric Rheumatology services of São Paulo state, Brazil. Anti-P antibody was measured by enzyme-linked immunosorbent assay (ELISA) in 228 cSLE patients. Demographic data, cumulative clinical and laboratorial features and disease damage (SLICC/ACR-DI) at last visit were evaluated.

**Results:** Anti-P antibody was observed in 61/228 (27%) cSLE patients. Frequencies of cumulative lymphadenopathy (29% vs. 15%,p = 0.013), acute confusional state (13% vs. 5%,p = 0.041), mood disorder (18% vs. 8%, p = 0.041), autoimmune hemolytic anemia (34% vs. 15%,p = 0.001), as well as anti-Sm (67% vs. 40%,p = 0.001), anti-RNP (39% vs. 21%,p = 0.012) and anti-Ro/SSA antibodies (43% vs. 25%,p = 0.016) were significantly higher in cSLE patients with anti-P antibodies compared to those without this autoantibody. Multiple regression model revealed that anti-P antibody was associated with autoimmune hemolytic anemia (OR = 2.719, 95%IC:1.365-5.418,p = 0.004) and anti-Sm antibody (OR = 2.758, 95%IC:1.304-5.833,p = 0.008). SLICC/ACR-DI was comparable in patients with and without anti-P antibodies (p = 0.780).

**Conclusion:** We describe a novel association of anti-P antibody and autoimmune hemolytic anemia in childhood onset SLE. The additional observation of an association of anti-P and anti-Sm antibodies is further supported by the same finding in lupus animal model.

**Disclosure of Interest**: None Declared

### P430 Fresh frozen plasma treatment for Systemic Lupus Eritematozus cases associated with complement deficiency

#### Kubra Ozturk, Zelal Ekinci

##### Department of Pediatrics Rheumatology, Kocaeli University Faculty of Medicine, Kocaeli, Turkey

###### **Presenting author:** Kubra Ozturk

**Introduction:** Systemic Lupus Eritematozus (SLE) is a multifactorial autoimmune disease whose etiology is unknown. Genetic background, immunological abnormalities and environmental factors are included in the pathogenesis. In case of hereditary deficiency of early complement components, SLE frequently develops in the early childhood.

**Objectives:** The aim of this report is to share our experiences gained during the course of five cases diagnosed as SLE with hereditary deficiency of early complement components.

**Methods:** Five cases from three different families diagnosed as SLE with hereditary deficiency of early complement components and treated successfully by fresh frozen plasma (FFP) are presented in this paper. All patients have been followed up in the Kocaeli University School of Medicine Department of Pediatric Rheumatology.

**Results:** Three female and two male patients are included in this report. Parents of all patients had consanguinity. There was no other individual diagnosed as SLE in the families mentioned. Initial complaint of all cases were skin findings while one had mucosal involvement and two had joint involvement. They did not have any other organ involvement. While antinuclear antibody was detected positive in all cases, low titer anti ds DNA positivity was detected in only one case. Prior to refering our hospital, four patients had been treated with steroids and hydroxychloroquine. In addition, two patients had been treated with methotrexate while two patients had been treated with azathioprine whereas one patient had not been treated previously. It was learned that a few patients have responded only partially to these treatments, while some have not at all. Thus, hereditary deficiency of early complement components was taken into consideration. While one patient had complement C1q deficiency, other four were considered to have deficiency of early complement component of classical pathway due to low CH50 level. All symptoms of all cases improved by FFP infusion, with a frequency of three days in a week at first, but once skin findings got calmed, the infusion was arranged with a frequency that would prevent the exacerbation of symptoms. Laboratory features and treatment modalities of the patients are presented in Table 6.

**Conclusion:** In early onset SLE cases in which skin findings are significant, hereditary deficiency of early complement components must be considered. It is known that in such cases, severe skin findings can not be controlled with conventional immunosuppressive treatment. For the present, regular FFP infusions seem to be a good option for remission. However, the duration of this treatment and its complications that may arise in the long term are debatable.

**Disclosure of Interest**: None Declared

**Table 6 (abstract P430).** Laboratory features and treatment modalities of the patients

	A	B1	B2	C1	C2
CH50	35% (70-140)	29% (40-130)	27% (40-130)	<3 U/ml (30-75)	<3 U/ml (30-75)
C1q	<6 mg/L (118-238)	126% (70-130)	150% (70-130)	Not evaluated	25 mg/dl (12-22)
C2	Not evaluated	Not evaluated	40 mg/L (14-25)	Not evaluated	3.1 mg/dl (1.13)
C3	175 mg/dl (90-180)	104 mg/dl (90-180)	152 mg/dl (90-180)	119 mg/dl (90-180)	95 mg/dl (90-180)
C4	37 mg/dl (10-40)	31 mg/dl (10-40)	38 mg/L (10-40)	30.4 mg/dl (10-40)	26mg/dl (10-40)
Prior treatment	Steroid	Steroid	Steroid	Steroid	No treatment
	Azathioprine	Methotrexate	Methotrexate	Azathioprine	
	Hydroxychloroquine	Hydroxychloroquine	Hydroxychloroquine	Hydroxychloroquine	
Present treatment	Hydroxychloroquine	Hydroxychloroquine	Hydroxychloroquine	Hydroxychloroquine	Hydroxychloroquine
	FFP	FFP	FFP	FFP	FFP
Time of FFP treatment	37 months	28 months	30 months	7 months	7 months
Frequency of FFP	3 weekly	4 weekly	3 weekly	Once a week	Once a week

### P431 Clinical value of 24-hour protein in urine for Nephritis activity in pediatric patients with SLE in comparison to anti-dsDNA

#### Maie Helal

##### Alexandria University, Alexandria, Egypt

**Introduction:** Systemic lupus erythematosus (SLE) is a prototypic multisystem autoimmune disorder. Lupus Nephritis(LN) is the most common form of affection in SLE. Clinical and laboratory monitoring of cases with LN is crucial to avoid relapse and ensure proper adjustment of therapy. While monitoring has been the corner stone for assessing disease activity, laboratory tests are helpful in systems that cannot be assessed clinically. Antibodies to (ds) DNA are found at some point during the course of the disease while, 24-hour protein in urine is widely regarded as the gold standard for laboratory assessment of proteinuria in LN.

**Objectives:** To evaluate 24-hour protein in urine in pediatric systemic lupus erythromatosus (pSLE) and determine its clinical and statistical association in nephritis activity in comparison to anti-dsDNA

**Methods:** 44 pSLE patients with LN and 30 healthy individuals were enrolled. Patient records were evaluated for clinical and laboratory associations. Disease activity assessment was performed using SLEDAI, Anti-dsDNA,renal biopsies and 24-hour protein in urine.

**Results:** Proteinuria correlated significantly with renal biopsies and decreased complement levels (p < 0.05). Proteinuria were significantly elevated in patients with class III/IV nephritis compared II/V nephritis. pSLE patients with active nephritis at the time of sample collection demonstrated significant proteinuria compared to those without active nephritis. 24-hour protein in urine was more sensitive and specific than anti-dsDNA in monitoring disease activity pSLE with LN.

**Table:** 

**24-hour protein in urine**	**SLEDAI**				**Test of sig.**	***p***	**OR**	**95% CI (LL-UL)**
	**≤4 (n = 36)**		**>4 (n = 28)**					
	**No.**	**%**	**No.**	**%**				
Abnormal ≥500 Normal	22 14	61.1 38.9	1 27	3.6 96.4	r = 0.65	<0.001^*^	42.429^*^	5.168-348.357

**Conclusion:** This study indicates the importance of monitoring 24-hour protein in urine in follow up visits of pSLE patients with LN. Elevated 24-hour protein in urine may be more indicative and specific for detecting disease activity, showing significant active nephritis than anti-DNA antibodies.

**Disclosure of Interest**: None Declared

### P432 Serum calprotectin is not associated with renal flare in juvenile systemic lupus erythematosus

#### Natalia Cabrera^1^, Alexandre Belot^1^, Jean Christophe Lega^2^, Jocelyne Drai^3^, Rene Ecochard^4^

##### ^1^Hôpital Femme Mère Enfant, Bron, Lyon, France; ^2^Internal Medicine and Vascular Department, CHU Lyon Sud, Lyon, France; ^3^Laboratory of Biochemistry and Molecular Biology, Lyon Sud, Lyon, France; ^4^Biostatistics Health Laboratory. “Claude Bernard” University of Lyon 1, UCBL1, Lyon, France

###### **Presenting author:** Natalia Cabrera

**Introduction:** Systemic lupus erythematosus (SLE) is characterized by a deregulation of the innate and adaptive immune system. SLE is the paradigm of tolerance breakthrough and underlying mechanisms are multiple, including defects in apoptosis, production of autoantibodies and formation of immune complexes within tissues (particularly renal parenchyma). Juvenile SLE (jSLE) is a rare disease that is more severe that in adult’s onset, mainly because of the more frequent kidney and neurological involvement. In the pathogenesis of SLE, the neutrophils have been recently identified as key players participating in a special active process of cellular death so called NETosis by releasing outside the cell DNA, histones and pro-inflammatory proteins such as MRP8/14 (named also calprotectin). Calprotectin is a key protein in the perpetuation of the inflammatory response by participation in the activation of intracellular signaling pathways; with DAMP role (damage-associated molecular patterns) in binding to TLR4 receptors granulocytes, involved in the production of interferon alpha (IFNα).

**Objectives:** Increased serum calprotectin levels have been reported increased in chronic rheumatic diseases including SLE, and could represent a biomarker of renal flare.

**Methods:** We retrospectively collected clinical and biological data from 30 patients with jSLE. This study was set up in a single academic center (Pediatric Nephrology and Rheumatology Department of University Hospital of Lyon). Forty frozen samples were analyzed for studying correlation of renal involvement.

**Results:** All 30 patients with jSLE from the retrospective cohort participated in the study. The average age at diagnosis was 13.4 (±4) years, with a sex ratio of 1/6. The *Systemic Lupus Erythematosus Disease Activity Index* (SLEDAI) score in jSLE was increased, mean 12 ± 8.6 (2 – 34). Twenty-two patients displayed a lupus nephritis (LN) in the course of the disease. LN is inaugural in half of cases. Delay of serum calprotectin measure was, in mean, 5 ± 5.2 years after beginning of jSLE; values varies from 0 to 18 years. We did not find any correlation with the title of anti-DNA antibodies (measured by *Farr* assay test), complement proteins (C3, C4 and CH50) and creatinine levels. The correlation analysis between serum calprotectin and the glomerular filtration rate (GFR calculated by Schwartz 2009 formula) was not significant (p = 0.6). Calprotectin is not different in patients with kidney disease (8.8 ± 12.2 mg/ml) compared to patients without renal involvement (7.4 ± 7.6 mg/ml).

**Conclusion:** We found no association between serum calprotectin dosage and clinic-biological parameters including: SLEDAI score, C3, and C4, creatinine levels, proteinuria and GFR. Serum calprotectin values were not significantly different between children with and without proteinuria. The calprotectin does not seem a biomarker of renal involvement in jSLE.

**Disclosure of Interest**: None Declared

### P433 Thrombosis in children with systemic lupus erythematosus

#### O. V. Shpitonkova, N. S. Podchernyaeva, Y.O. Kostina, N. G. Dashkova, M. K. Osminina

##### Pediatric Department, I.M. Sechenov First Moscow State Medical University, Moscow, Russian Federation

###### **Presenting author:** O. V. Shpitonkova

This abstract is not included here as it has already been published.

### P434 Preliminary validation of the Turkish Simple Measure of Impact of Lupus Erythematosus in Youngsters (SMILEY) in a single center

#### Gozde Yucel^1^, Sezgin Sahin^1^, Amra Adrovic^1^, Kenan Barut^1^, Ela Tarakci^1^, Ahmet Arvas^2^, Nandini Moorthy^3^, Ozgur Kasapcopur^1^

##### ^1^Pediatric Rheumatology, Istanbul University, Cerrahpasa Medical Faculty, Istanbul, Turkey; ^2^Pediatrics, Istanbul University, Cerrahpasa Medical Faculty, Istanbul, Turkey; ^3^Pediatric Rheumatology, Rutgers University, Robert Wood Johnson Medical School, Liverpool, United Kingdom

###### **Presenting author:** Ozgur Kasapcopur

**Introduction:** Juvenile systemic lupus erythematosus (SLE) is a chronic multisystemic disease with an episodic course which is prevalent in all cultures with wide-ranging effects on their health-related quality of life (HRQOL). In order to detect the effect of SLE on pediatric population and their parents; a disease specific HRQOL tool, called Simple Measure of Impact of Lupus Erythematosus in Youngsters (SMILEY), was developed, translated into different languages and validated in several languages.

**Objectives:** To determine the validity and reliability of the Turkish SMILEY in our center. Here we are presenting the preliminary data of our single-center research.

**Methods:** In our cross-sectional study, Turkish children and adolescents 8–18 years of age with SLE and their parents were enrolled. Pediatric SLE patients and parents completed child and parent reports of Turkish SMILEY and Turkish Pediatric Quality of Life Inventory (PedsQL^TM^) Generic module. Disease activity was estimated by examining physicians with usage of the SLE disease activity index (SLEDAI) and Physician’s Global Assessment of disease activity (PGA); chronic damage with the Systemic Lupus Erythematosus International Collaborating Clinics ACR Damage Index (SDI). Test-retest reliability, agreement between child and parent reports of the Turkish SMILEY and validity modalities were examined.

**Results:** 70 children with SLE (Male/Female 11/59; mean age at investigation 15.4 ± 2.8 years, mean disease duration 41.4 ± 29.4 months) were recruited into the study. Our patients have a median SLEDAI of 4 (range 0-23), and median SDI of 0 (0-5), and median PGA was 1 (0-4) and 80% of these were active (SLEDAI > 0).

Out of 70 children, only one child didn’t complete the child report SMILEY scale; but all of seventy parents were able to fulfill their corresponding report of the Turkish SMILEY. 59 child subjects and 60 parent subjects solved the Turkish SMILEY again 14 days later. The ICC for all domains and total scores of child report (0.7-0.9, *P* < 0.001) and parent report (0.6-0.9, *P <* 0.001) was significant, thus confirming excellent test-retest reliability.

Agreement between children and their parents was found to be favoring. For the existing child-parent pairs (n = 69), moderate rho (0.4-0.6, *P* < 0.001) and significant ICC (0.6 - 0.8, *P* < 0.001) values were seen between the child and parent SMILEY total and domain scores.

For children; the mean SMILEY total score was 70.3 ± 13.4 and the mean PedsQL^TM^ Generic module score was 78 ± 15.7. For the parents they were calculated as 70.1 ± 13.4 and 77 ± 16.5 respectively. There was a significant correlation between these scores both for the children (r = 0.5, *P* < 0.001) and the parents (r = 0.4, *P* < 0.001).

In our study; important Spearman’s correlations were found between child report of Turkish SMILEY and factors affecting morbidity, mortality. The child SMILEY total score (n = 69) correlated remarkably with the PGA (r = 0.7, *P* < 0.001), SLEDAI (r = 0.4, *P* < 0.001), and SDI (r = 0.5, *P* < 0.001).

The children with lower disease activity and damage were discovered to have higher scores in their corresponding SMILEY report, hereby emphasizing better QOL. This relationship was especially noticeable in total score and the limitation domain of SMILEY.

**Conclusion:** This first validation study about HRQOL of pediatric SLE patients in Turkey showed that; Turkish SMILEY is a useful, valid and a reliable disease-specific questionnaire which can be further used as a beneficial research tool in our country.

**Disclosure of Interest**: None Declared

### P435 Juvenile-onset lupus nephritis and local kidney involvement: gaining insight into the role of the glomerular endothelial cells in urinary biomarker production

#### Paraskevi Dimou^1^, Angela Midgley^1^, Matthew Peak^1,2^, Simon C. Satchell^3^, Rachael D. Wright^1^, Michael W. Beresford^1,2^

##### ^1^Women’s and Children’s Health, Institute of Translational Medicine, University of Liverpool, Liverpool, United Kingdom; ^2^Alder Hey Children’s Hospital, Liverpool, United Kingdom; ^3^Academic Renal Unit, University of Bristol, Bristol, United Kingdom

###### **Presenting author:** Paraskevi Dimou

**Introduction:** Lupus nephritis (LN) is a serious clinical manifestation of juvenile systemic lupus erythematosus (JSLE) causing chronic renal inflammation and the loss of high amounts of proteins in the urine (proteinuria), which can prove to be useful biomarkers for non-invasive renal disease monitoring.

**Objectives:** Our study aimed to investigate whether the glomerular endothelial cells (GEnCs), which are essential for the processes of blood filtration and urine production, are capable of producing Monocyte Chemoattractant Protein-1 (MCP-1) and Vascular Cell Adhesion Molecule-1 (VCAM-1); two candidate novel urinary protein biomarkers.

**Methods:** Conditionally immortalized human GEnCs (ciGEnCs) were treated for 48 hours at 37°C with 5% sera from JSLE patients with a history of renal involvement (n = 5) and age- and sex-matched healthy controls (n = 5). ciGEnCs in culture medium with 5% FBS were used as negative controls. ciGEnCs were also stimulated with 10ng/ml interferon-alpha (IFNα), a cytokine central to JSLE pathogenesis. After 48 hours, culture media were collected and total RNA was isolated. Quantitative real-time PCR (qRT-PCR) for MCP-1 and VCAM-1 mRNA and ELISA’s for MCP-1 and soluble VCAM-1 (sVCAM-1) proteins were performed. JSLE and healthy control serum samples were included in the ELISA assays in order to determine whether MCP-1 and sVCAM-1 were secreted by the ciGEnCs or already present in the sera. Data analysis was performed using Friedman’s test with Dunn’s post-hoc test.

**Results:** All treatment groups including negative controls expressed the MCP-1 and VCAM-1 mRNAs. However, qRT-PCR analysis did not reveal any statistically significant differences in mRNA expression. MCP-1 protein was secreted exclusively by the ciGEnCs whereas no detectable levels were found in the serum samples. JSLE sera augmented MCP-1 secretion (552.8 pg/ml; 197.4-1186) compared to healthy control sera (271.8 pg/ml; 258.4-1524) but this increase did not reach statistical significance. On the contrary, sVCAM-1 was mainly detected in JSLE and healthy control sera whereas the ciGEnCs secreted only minor amounts of sVCAM-1.

**Conclusion:** The ciGEnCs have the ability to express MCP-1 and VCAM-1. As there are not significant differences in mRNA and protein amounts for MCP-1, the ciGEnCs might not be the sole source of this biomarker in the kidney; other resident renal cells could be also activated to produce MCP-1. Furthermore, MCP-1 production might be secondary to other contributors to renal inflammation in LN. The fact that the ciGEnCs do not secrete significant amounts of sVCAM-1 highlights a potentially important role for the membrane-bound VCAM-1 in LN. The highly inflammatory environment of the lupus kidney could activate the GEnCs to express VCAM-1 on their surface and secrete MCP-1; membrane VCAM-1 and MCP-1 could in turn promote immune cell infiltration into the glomerulus, resulting in sustained renal inflammation. sVCAM-1 could be an indicator of systemic JSLE disease activity but not specific for LN. Finally, MCP-1 in combination with other candidate novel urinary biomarkers could prove to be a potentially important indicator of local kidney involvement in LN.

**Disclosure of Interest**: None Declared

### P436 Working towards a clinical trial of urine biomarker led monitoring in lupus nephritis: can patients send their urine to hospital through the post?

#### Rachel Corkhill, Eve M. Smith, Michael W. Beresford

##### Woman and Childrens Health, Liverpool University, Liverpool, United Kingdom

###### **Presenting author:** Rachel Corkhill

**Introduction:** Lupus nephritis (LN) affects up to 80% of Juvenile-onset Systemic Lupus Erythematosus (JSLE) patients. Conventional markers of JSLE disease activity are poor at adequately detecting LN flares and the role of renal biopsy in LN monitoring is limited by it’s invasive nature. A urine ‘biomarker panel’ comprising of vascular cell adhesion molecule-1 (VCAM-1), monocyte chemoattractant protein 1 (MCP-1), lipocalin like prostaglandin D synthase (LPGDS), transferrin, ceruloplasmin and alpha-1-acid glycoprotein (AGP) has been shown to perform to a ‘excellent’ level for identification of active LN on a cross sectional basis within the UK JSLE Cohort Study. The ability of this panel to predict flare, remission, treatment response and prognosis has not been fully elucidated. To this end, a prospective study collecting serial, frequent JSLE patient urine samples is warranted. To reduce the impact of such a study on patients lives, these samples would ideally be sent to hospital through the post.

**Objectives:** To assess the stability of urine biomarkers in samples which have been processed using accepted standard techniques compared with those that undergo delayed processing but are transported in the presence of additives (protease inhibitors or boric acid) and an ice-pack.

**Methods:** Participants of the UK JSLE Cohort Study, aged < 16 years at diagnosis were included. Urine samples were obtained at routine clinics. One aliquot underwent standard processing (centrifuged at 2000rpm for 5 minutes, stored at -80°C) and four more aliquots were taken and added to eppendorfs containing protease inhibitor 1 (condition 1), protease inhibitor 2 (condition 2), protease inhibitor 1 and boric acid (BA, to inhibit bacterial growth, condition 3), protease inhibitor 2 and BA (condition 4). After 48 hours, the samples were centrifuged at 2000rpm for 5 minutes and stored at -80°C. Novel urinary biomarkers VCAM-1, MCP-1, LPGDS, transferrin, ceruloplasmin and AGP were quantified by enzyme-linked immunosorbent assays. Biomarker levels in samples exposed to conditions 1-4 were compared to those processed in the standard fashion. The study had full ethical approval in place.

**Results:** Six JSLE patients and two healthy controls were included, 7 female / 1 male, median age 15.6 years (IQR 12.1-16.6) and length of disease 1.8 years (IQR 13.2-16.2). Condition 1, including protease inhibitor 1 and ice proved to be better than other conditions for maintaining biomarker levels. Compared with standard processing, biomarkers in the presence of condition 1 were as follows; VCAM-1 111%, AGP 87%, CP 120%, TF 99%, LPGDS 108%, MCP-1 106%. With condition 1, all biomarker levels were within 20% of the standard urine processing levels. The second best condition was condition 3, which included protease inhibitor 1 & BA & ice (see Table below).

**Table:** 

**Urine processing conditions**	**% of Standard Processing**					
	**VCAM-1**	**AGP**	**CP**	**TF**	**LPGDS**	**MCP-1**
**Protease inhibitor 1 + ice**	111	87	120	99	108	106
**Protease inhibitor 2 + ice**	114	95	122	85	116	62
**Boric acid + protease inhibitor 1 + ice**	114	104	116	106	106	78
**Boric acid + protease inhibitor 2 + ice**	116	121	113	171	117	426

**Conclusion:** Use of protease inhibitor 1 and ice-packs with samples being transported through the post provides an opportunity for a clinical study which will provide very close monitoring of urine biomarker levels without having to bring the patient to hospital very frequently, reducing the impact of the study on their lives. Such a study will be able to accurately identify if such markers are able to predict flare, remission, treatment response, informing a study of urine biomarker led monitoring.

**Disclosure of Interest**: None Declared

### P437 Complement deficiency in early-onset lupus: experience from a tertiary care centre in North India

#### Sagar Bhattad^1^, Amit Rawat^1^, Surjit Singh^1^, Anju Gupta^1^, Deepti Suri^1^, Martin de Boer^2^, Taco Kuijpers^2^

##### ^1^Pediatrics, PGIMER, Chandigarh, India, Chandigarh, India; ^2^Blood lab, Sanquin Blood Supply Foundation, Amsterdam, Netherlands

###### **Presenting author:** Sagar Bhattad

**Introduction:** Inherited deficiencies of early complement components of the classical pathway (C1q, C4 and C2) are the strongest risk factors for monogenic forms of lupus. This form of lupus characteristically has a young age at onset (1), predominant cutaneous manifestations and unique management strategies (2).

**Objectives:** To assay complement C1q, C2, C3 and C4 and CH50 in children with early-onset lupus (onset below 5 years).

**Methods:** Children with systemic lupus erythematosus (SLE) attending the Pediatric Rheumatology Clinic at our institute were enrolled after written informed consent. Records of those who had an onset of SLE below 5 years of age were analyzed in detail. Complement components C1q and C2 levels were estimated by enzyme-linked immunosorbent assay (ELISA) whereas C3 and C4 were measured by end-point nephelometry. Functional assay for the classical pathway (CH50) was carried out by ELISA. Mutation analysis of the classical complement pathway proteins was carried out in children with depressed complement levels.

**Results:** Five children with onset of SLE below 5 years were noted. Male:female ratio was 3:2. Mean age of onset was 1.9 years. Mean duration of illness at the time of enrollment was 4.6 years (0.2 to 9.5 years). Rash was noted in 80%, renal involvement in one and neurological involvement was documented in one. Anti-nuclear antibodies were detected in all and they were of speckled pattern on indirect immunofluorescence (IIF). Anti-dsDNA antibodies were negative in all, except one.

All children had markedly reduced CH50, 3 had low C1q, while 2 had low C4. Amongst children with low C1q, mutation analysis of C1qA gene revealed a homozygous nonsense mutation: C1QA (NM_015991) c.622C > T, p.Q208X in *Patient 1*. A homozygous acceptor splice site mutation at the −2 position of intron 2 of C1QA (c.164-2A > C) was detected in *Patient 2.* In the third child with C1q deficiency, anti-C1q antibodies were absent.

*Patient 1* had lost a sibling due to probable lupus. On screening of family members, younger brother was noted to have markedly reduced CH50, low C1q and mutation analysis revealed homozygous mutation in the same gene. He, however, is asymptomatic at present. Details have been tabulated below:

**Conclusion:** Children with C1q deficiency developed SLE at a young age and presented predominantly with cutaneous manifestations. Immunological investigations revealed negative anti-dsDNA and low CH50. Mutations in C1QA gene were noted in 2 children with early onset SLE. One child with C1QA gene mutation is asymptomatic and is under close follow-up.

**References:**

1. Stegert M, Bock M, Trendelenburg M. Clinical presentation of human C1q deficiency: how much of a lupus? Mol Immunol 2015;67(1):3–11

2. Arkwright PD, Riley P, Hughes SM, Alachkar H, Wynn RF. Successful cure of C1q deficiency in human subjects treated with hematopoietic stem cell transplantation. J Allergy Clin Immunol 2014 ;133(1):265-7

**Disclosure of Interest**: None Declared

**Table 7 (abstract P437).** Details of children with early onset lupus

Sl. No	Age at onset	Predominant clinical manifestations	ANA	Anti- dsDNA (IU/ml)	C1q	C3	C4	CH 50	Mutation analysis
1	2.5	Cutaneous, CNS	4+ (s)	1.5	0.24	1610	420	0	C1QA (NM_015991) c.622C > T, p.Q208X
Brother of 1	^a^	Asymptomatic	3+ (s)	1.0	0.27	1970	400	0	C1QA (NM_015991) c.622C > T, p.Q208X
2	1.5	Cutaneous	3+ (s)	7.7	0.37	2500	500	0.09	C1QA (c.164-2A > C)
3	2	Muco-cutaneous	4+ (s)	6	3.3	156	36	0.1	-
4	3	Renal	4+ (s)	16	30	47	<3	0.33	-
5	0.7	Cutaneous	3+ (s)	109	164	86	10	1.6	-

^a^ Asymptomatic, s- speckled pattern on IIF

### P438 Neuropsychiatric lupus with hepatitis in a child with CMV co-infection: chicken first or the egg?

#### Sagar Bhattad, Amit Rawat, Anju Gupta, Deepti Suri, Vignesh Pandiarajan, Surjit Singh

##### Pediatrics, PGIMER, Chandigarh, India, Chandigarh, India

###### **Presenting author:** Sagar Bhattad

**Introduction:** Systemic lupus erythematosus (SLE) is a multi-systemic disease with multifactorial etiology. Cytomegalovirus (CMV) infection at first presentation in pediatric SLE (pSLE) is a rare phenomenon. In a study of 670 patients with pSLE (1), CMV infection at the time of diagnosis was noted in 7 patients (incidence: 1.04%). This is the largest series reporting CMV in pSLE.

**Objectives:** To report an adolescent girl with SLE presenting with neurological and hepatic manifestations and CMV co-infection.

**Methods:** 12-year old girl presented to our department with history of low grade intermittent fever for 7 months, maculopapular rash over trunk and photosensitive malar rash for 5 months and jaundice for 1 month. She had altered behavior with agitation, disorientation, fluctuating consciousness, hallucinations and altered sleep. There was no significant past history and her development was age-appropriate. On examination, she had a small head, malar rash, icterus and hepatosplenomegaly. She also had catatonia, mutism, would stare intermittently, had low speech output and psychomotor retardation with rigidity. There was no focal deficit. Investigations revealed pancytopenia, transaminitis, conjugated hyperbilirubinemia, normal renal functions. Immunological tests: anti-nuclear antibody (ANA) - strongly positive (homogenous pattern on IIF), high anti-dsDNA with hypocomplementemia. Tests have been tabulated.

She was evaluated extensively for cause of hepatitis (Table 8). Liver biopsy revealed steatosis with hepatitis. Screen for infections was negative, except CMV. Very high levels of CMV DNA in blood were noted on PCR. It was a clinical dilemma as to whether CMV was causative, co-infection or a re-activation due to immunosuppression.

For neuropsychiatric manifestations, CSF analysis was performed which was normal. Magnetic resonance imaging (MRI) of brain showed cortical atrophy. There was no evidence of any vascular involvement.

**Results:** Child was treated with intravenous (IV) methylprednisolone, IV cyclophosphamide pulses and ganciclovir. CMV hyper-immune globulin could not be administered due to financial constraints. Over next 6 weeks, she had improvement in neurological status. Hallucinations, agitation and rigidity had become passive, while there was mild improvement in psychomotor retardation. Transaminitis gradually recovered, bilirubin normalized and on repeat testing, CMV was undetectable. She is now doing well on maintenance immunosuppression and oral valganciclovir.

**Conclusion:** We present a challenging case of SLE with neuropsychiatric and hepatic manifestations along with CMV co-infection. Whether CMV came first or SLE remains unresolved. The ideal strategy of managing such patients is yet to be defined.

**References**

1. Rozenblyum EV, Levy DM, Allen U, Harvey E, Hebert D, Silverman ED. Cytomegalovirus in pediatric systemic lupus erythematosus: prevalence and clinical manifestations. Lupus 2015;24(7):730-5

**Disclosure of Interest**: None Declared

**Table 8 (abstract P438).** Investigations in the index child

Investigation	Result	Investigation	Result
Hemoglobin (g/L)	87	ANA	3+ (homogenous)
Leucocyte counts (x 10^9^/L)	6	Anti-dsDNA	733 IU/ml (<40)
Platelet counts (X 10^9^/L)	20	C3 C4	68 (50-150) 6 (20-50)
Urea/creatinine	15/0.6	DCT	Anti-IgG +, C3d -
Serum protein/albumin	5.8/2.8	APL work up	negative
Total bilirubin/ direct	13/12	Serologies for HAV, HCV, HEV, HIV, EBV	negative
AST/ALT/ALP	1650/120/455	CMV PCR	8160 copies/ml
PTI	50%	Anti-LKM/ SMA/ PCA	negative
apTT	57 (<28)	MRI Brain	Cerebral atrophy

*DCT* Direct Coombs test, *APL* anti-phospholipid, *HAV* Hepatitis A, *HCV* Hepatitis C, *HEV* Hepatitis E, *HIV* Human Immunodeficiency Virus, *EBV* Epstein Barr Virus

### P439 Soluble Receptor Activator of Nuclear Factor κ B ligand (s RANK-L) levels in pediatric onset SLE

#### Sapna Sandal, Amit Rawat, Anju Gupta, Surjit Singh

##### Pediatrics, PGIMER, Chandigarh, Chandigarh, India

###### **Presenting author:** Sapna Sandal

**Introduction:** Receptor Activator of Nuclear Factor κ B (RANK), its ligand (RANKL) and osteoprotegerin are the key mediators of bone remodeling and the final effector pathway in osteoclast development and differentiation. The data on RANKL axis in pediatric Systemic Lupus Erythematosus (SLE) is lacking.

**Objectives:** To estimate serum sRANKL levels in pediatric SLE and to evaluate the correlation of sRANKL levels with the SLE disease activity.

**Methods:** Consecutive children with SLE attending Pediatric Rheumatology Clinic of Advanced Pediatrics Centre, PGIMER, Chandigarh were enrolled. The study group was divided into active (with ongoing disease activity) and inactive (no disease activity) subgroups based on SLE disease activity index (SLEDAI) scores. The sRANKL ligand levels were measured using an enzyme- linked immunosorbent assay (sRANKL – ELISA MyBioSource^@^, USA).

**Results:** Thirty-one children (12 boys) with a mean age of 13.4 ± 3.2 years were included. The median (interquartile range) sRANKL level of the cohort was 52.3 (24.1, 66.4) pg/mL. Serum RANKL levels were not significantly different in active and inactive disease subgroups [median (interquartile range): 55.2 (21.3, 66.4) pg/mL versus 53.3 (29.3, 64.9) pg/mL, respectively] (p = 0.89). There was no statistically significant correlation between sRANKL levels and SLEDAI scores, Spearman correlation coefficient r_s_ = 0.083, p = 0.65.

**Conclusion:** There was no statistically significant difference in sRANKL levels between the inactive and active disease group which is further reflected in a lack of correlation between sRANKL and SLEDAI scores.

**Disclosure of Interest**: None Declared

### P440 Heart valve damage in patients with lupus and antiphospholipid syndrome

#### Sebastian Giraldo^1,2^, Roy Sanguino^3^, Adriana S. Diaz^4,5^

##### ^1^Rheumatology and Inmunology, Hospital Militar Central, Bogota, Colombia; ^2^School of Medicine, Universidad Militar Nueva Granada, Bogota, Colombia; ^3^Cardiology Department, Hospital de La Misericordia, Bogota, Colombia; ^4^Rheumatology, Hospital de La Misericordia, Bogota, Colombia; ^5^Rheumatology, Care for Kids, Bogota, Colombia

###### **Presenting author:** Sebastian Giraldo

**Introduction:** Heart valve disease is the most common manifestation in systemic lupus erythematosus (SLE), even more so when the association with antiphospholipid syndrome (APS) is present, showing a prevalence of valvular commitment to 50%, which is a common cause for morbidity and mortality in these patients. Manifestations include thickening, stenosis or aseptic thrombus on the surface of the valve (Libman-Sacks endocarditis). The mitral valve is involved in 24% of cases, and the aortic valve and the tricuspid valve or pulmonary up to 5%. It is more common in female.

**Objectives:** Valvular heart disease is common in systemic lupus erythematosus (SLE) associated with antiphospholipid syndrome (APS) in children. Important entity in the initiation of immune pathogenesis. Information on the role of antiphospholipid antibodies has been inconsistent, particularly with regard to valvular lesions and associated SLE. Although the diagnosis by echocardiography on time is essential to prevent progression of this type of injury, still it remains a real challenge to the therapeutic approach to this entity given the risk of valve replacement and / or chronic anticoagulation therapy.

**Methods:** Three cases of valve disease are related in the context of SLE and APS association with different presentation and management of symptoms, who were diagnosis and followed at the Hospital La Misericordia, Bogota-Colombia.

**Results:** Two female patients 12 and 11 years old with positive immunological profile for SLE and APS, commitment largest organ kidney and heart involvement. In one clinical improvement of their valvular involvement (severe tricuspid regurgitation, pulmonary hypertension and right heart failure) once management is done with systemic steroids (methylprednisolone), and cyclophosphamide, in maintenance therapy with prednisolone, Azathioprine, Chloroquine, Enalapril and spironolactone. The second case, despite stabilization of autoimmune disease therapy Methylprednisolone and Cyclophosphamide continued progression of their cardiac involvement (aortic and mitral insufficiency) and therefore requires surgery. The third case, a man seven years old with a diagnosis of SLE and associated APS, who makes kidney and central nervous system involvement. It requires pulses of intravenous steroid, cyclophosphamide, mycophenolate mofetil, plasmaferisis and renal replacement therapy. Cardiac involvement (tricuspid regurgitation) is documented, who does two episodes of disease activity occurring in less than two years. Severe tricuspid valvular commitment and refractory heart failure without achieving stabilization and death finally.

**Conclusion:** The treatment of valvular manifestation in SLE associated with APS depends on the type and severity of involvement. The important valvular dysfunction can influence hemodynamic compromise so the treatment is initially aimed at conservative therapy including ACE inhibitor, beta blocker, diuretic, immunosuppression, anticoagulation therapy and prophylaxis of endocarditis) in some patients undergoing chronic anticoagulation therapy until the need for surgical approach with possible complications with biological and mechanical valve replacement.

**Disclosure of Interest**: None Declared

### P441 The impact of Juvenile Systemic Lupus Erythematosus on psycho-social status

#### Selcuk Uzuner^1^, Sezgin Sahin^2^, Gizem Durcan^3^, Amra Adrovic^2^, Kenan Barut^2^, Ali Guven Kilicoglu^4^, Ayhan Bilgic^5^, Kayhan Bahali^4^, Ozgur Kasapcopur^2^

##### ^1^Pediatrics, Bezmialem University, Istanbul, Turkey; ^2^Pediatric Rheumatology, Istanbul University, Cerrahpasa Medical School, Istanbul, Turkey; ^3^Child and Adolescent Psychiatry, Istanbul University, Cerrahpasa Medical School, Istanbul, Turkey; ^4^Child and Adolescent Psychiatry, Bakirkoy Research and Training Hospital for Psychiatry, Neurology and Neurosurgery, Istanbul, Turkey; ^5^Child and Adolescent Psychiatry, Necmettin Erbakan University, Meram Medical Faculty, Konya, Turkey

###### **Presenting author:** Selcuk Uzuner

**Introduction:** There is very little documentation about the association between peer victimization, psychological status, and quality of life (QOL) in adolescents with Juvenile Systemic Lupus Erythematosus (JSLE).

**Objectives:** Adolescent studies have generally reported that chronic conditions can negatively affect psychosocial status. The aim of this study was to evaluate the association between peer victimization, psychological symptoms and QOL in a cohort of adolescents with JSLE.

**Methods:** 31 patients participated in this study. The inclusion criteria were as follows: 12–18 years of age, no psychotropic drug treatments for at least three months prior to the study, and no other chronic diseases. The control group (n = 39) was composed of healthy adolescents from the local community. Questionnaires were used to evaluate the peer victimization, psychological status and QOL of children with JSLE and without JSLE.

**Results:** The mean (± SD) age of the sample was 15.7 (±1.6) years, and consisted of 6 (19.3%) males and 25 (80.7%) females. The mean (± SD) age of the control group was 15.6 (±1.4) years, and 31 (79.5%) of the adolescents in this group were females. No significant difference was found between study and control group for peer victimization, depression, state and trait anxiety, self-esteem, and QOL scores.

**Conclusion:** To the best of our knowledge, this is the first report on peer victimization, psychological status, and QOL data, especially focused on adolescents with JSLE, compared with a control group. The present study revealed that peer victimization, depression, anxiety, self-esteem, and QOL levels of the patients with JSLE were not worse than the control group.

**Disclosure of Interest**: None Declared

### P442 Pentraxin-3 level predicts vasculitis and mucocutaneous involvement in childhood-onset systemic lupus erythematosus

#### Sezgin Sahin^1^, Amra Adrovic^1^, Kenan Barut^1^, Sinem Durmus^2^, Hafize Uzun^2^, Ozgur Kasapcopur^1^

##### ^1^Department of Pediatric Rheumatology, Istanbul University, Cerrahpasa Medical School, Istanbul, Turkey; ^2^Department of Biochemistry, Istanbul University, Cerrahpasa Medical School, Istanbul, Turkey

###### **Presenting author:** Sezgin Sahin

**Introduction:** Systemic lupus erythematosus (SLE) is a persistent or remitting-relapsing autoimmune disease that handled in the context of connective tissue diseases and also vasculitides. Although there are commonly used biomarkers of active kidney disease and central nervous system disease, there is not a specific marker for vasculitic involvement.

Pentraxin-3 (PTX3) is derived primarily from vascular endothelium and innate immunity cells in response to local inflammation and plays an important role locally at the site of inflammation. Thus PTX3 seems to be a useful biomarker directly reflecting local inflammation and local vasculitis.

However, there are conflicting results about the role of PTX3 in SLE. Moreover, to the best of our knowledge PTX-3 was not studied in children with SLE.

**Objectives:** In the present study, we aimed to compare the concentrations of plasma PTX-3 among childhood-onset SLE (cSLE) patients and control groups and to assess the association of PTX-3 levels with SLEDAI-2K, clinical manifestations and laboratory results.

**Methods:** From October 2015 to May 2016, 82 cSLE patients were examined at our outpatient clinic of pediatric rheumatology department. One patient and also her parents did not give consent for participation so she was not enrolled to the study. Since bacterial and viral infections could interfere with pentraxin levels, 5 patients were not recruited due to signs and symptoms of active infection. Moreover, the procalcitonin level was also measured to rule out bacterial and viral infection. Finally, a total of 76 c-SLE patients without active infection sign and symptom were eligible for this cross-sectional single center study. We have also measured pentraxin-3 level in 41 healthy and age-matched controls.

Both the cumulative and current organ involvement and manifestations were recorded from patient records and from the last examination at that moment, respectively. All of the laboratory analyses were studied concurrently with PTX-3 levels. PedSDI and SLEDAI-2K scores at disease onset, at the most severe flare and at the last examination were calculated.

Serum pentraxin-3 levels were measured by a commercially available enzyme-linked immunosorbent assay kit.

**Results:** Plasma PTX3 concentrations were measured in 76 patients with c-SLE and 41 control subjects. Plasma PTX3 concentration of the SLE patients was significantly higher than that of the healthy controls (mean 10.6 ± 8.2 vs. 2.7 ± 1.3 ng/mL, p < 0.001).

The ratio of females to males with cSLE was 5.3:1. The mean SLEDAI scores decreased from 10.3 ± 4.8 (at disease onset) to 5.2 ± 5.3 (at last examination). Additionally, only 10.5% (n = 8) and 3.9% (n = 3) of the cohort were displaying the signs of active nephritis and active neuropsychiatric disease at last examination.

In patients with SLE, PTX3 concentrations were correlated with SLEDAI-2K (p < 0.001), active vasculitis (p < 0.001), Raynaud’s phenomenon (p = 0.006) and active mucocutaneous involvement (p < 0.001). PTX3 level was not associated with disease duration, anti-ds DNA antibody, decreased complement levels, PedSDI, active nephritis, active neuropsychiatric involvement, musculoskeletal involvement, hematological involvement, ESR,CRP, procalcitonin levels.

**Conclusion:** In brief, PTX3 levels were significantly correlated with SLEDAI scores in cSLE. Additionally, its levels are found to be substantially increased in the presence of Raynaud’s phenomenon and vasculitic manifestations. Thus predicting the vascular involvement early and quantitatively in a cSLE patient with presumed clinically inactive, will provide a better management of the disease. In conclusion, as in other vasculitides, PTX3 may represent a potential biomarker for vascular involvement in cSLE.

**Disclosure of Interest**: None Declared

### P443 Age-related differences in childhood-onset systemic lupus erythematosus

#### Sezgin Sahin^1^, Amra Adrovic^1^, Kenan Barut^1^, Nur Canpolat^2^, Salim Caliskan^2^, Lale Sever^2^, Ozgur Kasapcopur^1^

##### ^1^Department of Pediatric Rheumatology, Istanbul University, Cerrahpasa Medical School, Istanbul, Turkey; ^2^Department of Pediatric Nephrology, Istanbul University, Cerrahpasa Medical School, Istanbul, Turkey

###### **Presenting author:** Sezgin Sahin

**Introduction:** Only 20% of the systemic lupus erythematosus (SLE) disease emerges before 18-years old and there are conflicting conclusions about demographic characteristics, disease manifestations of this autoimmune disease based on age at disease onset.

**Objectives:** To compare the disease characteristics, SLEDAI-2K and PedSDI scores, medications, laboratory data between 2 different age groups of c-SLE.

**Methods:** This single-center cohort study is implemented in a retrospective manner by reviewing patient records and included 82 c-SLE patients from January 2003 to October 2015.

Patients were divided into two groups based on age at disease onset: group A (early onset and school-aged children that are <12 years old), group B (adolescents that are ≥12 years-old and <18 years-old).

Pearson chi-square test compared the sex differences, presence of constitutional symptoms and decrease in complement levels at disease onset, the number of ANA, anti-dsDNA and anticardiolipin antibody positivity, existence of organ involvements, Reynaud’s phenomenon, all medications that are used, and finally comorbid conditions and side effects related the drug between these groups.

Moreover, delay in diagnosis (months), the measured procalcitonin, ESR, CRP, Anti-dsDNA levels (both at disease onset and at last visit) and the other continuous variables such as number of flares, PedSDI and SLEDAI scores (at disease onset, at last examination and the peak score ever reached) were compared by means of Mann-Whitney U test.

**Results:** 39 patients had their disease onset before 12-years-old and 43 patients at or after 12-years-old. Age at diagnosis was 8.5 ± 2.6 (range 2-11) in group A and 14.4 ± 1.7 (range 12-18) in group B. We were following the Group A and B on an average for 5.5 years and 3.5 years, respectively. The ratio of females to males was 5.5:1 in Group A and 6.1:1 in Group B.

Delay in diagnosis of SLE in younger patients were significantly higher than that of the adolescents by means of months (median 8 vs. 1 months, p = 0.0016).

In Group A, constitutional symptoms were pronounced in 43.6% (n = 17) of the patients at disease onset. However, this percentage was 67.4% (n = 29) in Group B and it was also statistically reliable.

Groups were similar regarding female gender (84.6% vs. 86%, p = 0.855) and frequency of two major organ involvement of c-SLE: nephritis (25.6% vs. 32.5%, p = 0.549), neuropsychiatric involvement (15.4% vs. 18.6%, p = 0.699).

Additionally, other cumulative organ involvement frequencies, such as cardiovascular disease, hematologic and musculoskeletal involvement, pulmonary involvement were same between these two groups.

Procalcitonin, ESR, CRP, Anti-dsDNA levels (both at disease onset and at last visit) and the other continuous variables such as number of flares, PedSDI and SLEDAI scores (at disease onset, at last examination and the peak score ever reached) were not different between groups.

The mean SLEDAI-2K scores of Group A; at disease onset, at last examination and during the most severe flare did not differed from Group B (p > 0.05).

Although the ratio of damaged individuals in Group A (n = 12/39) were increased comparing to Group B (n = 12/43), this was not statistically significant (p > 0.05).

There was not any difference in number of patients that had positive anti-ds DNA antibody, anticardiolipin antibody, ANA and decreased complement levels among different age groups.

**Conclusion:** Unlike the reported data, we could not find an increased risk of disease severity and organ involvement in children less than 12-years-old. However, we conclude that diagnosing SLE seems to be more difficult in younger children.

**Disclosure of Interest**: None Declared

### P444 Ovarian tissue cryopreservation before cyclophosphamide treatment for severe lupus nephritis; two juvenile systemic lupus erythematosus cases

#### Tomomi Sato^1^, Fuminori Kimura^2^

##### ^1^Department of Pediatrics, Shiga University of Medical Science, Otsu, Japan; ^2^Department of Obstetrics and Gynecology, Shiga University of Medical Science, Otsu, Japan

###### **Presenting author:** Tomomi Sato

**Introduction:** Severe lupus nephritis, often a complication in pediatric cases of systemic lupus erythematosus (SLE), is often treated using cyclophosphamide, which seriously declines fertility, when mycophenolic acid cannot be used owing to its side effect. It is known that many female patients with various cancers opted for ovarian tissue cryopreservation before receiving alkylating antineoplastic agents and some of them have given birth after transplantation of their cryopreserved ovarian cortex tissue.

**Objectives:** Two cases of girls with severe lupus nephritis underwent ovarian tissue cryopreservation before cyclophosphamide treatment for preservation their fertility.

**Methods:** Case 1 was a 9-year-old girl with lupus nephritis class IV-S (a), complicated with nephrotic syndrome, anti-phospholipid syndrome and idiopathic thrombocytopenic purpura. Case 2 was a 14-year-old girl with lupus nephritis class III-G (a/c) and diffuse alveolar hemorrhage. Lupus nephritis was diagnosed on kidney biopsy at initial diagnosis of SLE in both cases. The patients were first treated with prednisolone (1 mg/kg/day) and mycophenolic acid (1500 mg/day); however they experienced severe stomachache with mycophenolic acid use and hence it was discontinued. They were determined to need cyclophosphamide therapy for remission from lupus nephritis. Before IVCY therapy, we suggested ovarian tissue cryopreservation to help preserve fertility. The patients and their parents agreed to cryopreserve their ovarian cortical tissue and provided written informed consent. They underwent laparoscopic right ovariectomy under general anesthesia before the initiation of cyclophosphamide pulse therapy for fertility preservation. Their ovarian cortical tissues were vitrified in liquid nitrogen and stored in our hospital.

**Results:** Laparoscopic ovariectomy was successful and cyclophosphamide pulse therapy was started two days after surgery. Pathological review of the ovarian tissue from the patient in case 1 showed stromal fibrosis, suggesting chronic ovaritis. The surgical scar was very small in both patients, and the degree of satisfaction of the patients and their parents was high. They both continue to receive oral steroid therapy and cyclophosphamide pulse therapy and are in remission.

**Conclusion:**

The survival rates of patients with lupus nephritis have dramatically improved recently due to development of treatment, so long-term damage to the ovary after cyclophosphamide therapy for severe lupus nephritis with girls has gained importance. SLE occurs frequently in young women; unfortunately, juvenile SLE is more often complicated by severe lupus nephritis, which may lead to end-stage renal failure, than adult SLE. Previous studies have suggested that laparoscopic ovariectomy under general anesthesia for cryopreservation is safe and that immature ovaries can restore hormonal function in prepubertal girls with cancer. Spontaneous full-term pregnancy after orthotropic cryopreserved ovarian tissue transplantation has been reported, and at least 40 live births after cryopreserved ovarian cortical tissue transplantation have been reported. Similar to female patients with cancer, patients with severe systemic lupus erythematosus should be provided with the option of ovarian tissue cryopreservation before the use of alkylating drugs to help preserve fertility.

**Disclosure of Interest**: None Declared

### P445 Pulmonary involvement in childhood onset systemic lupus erythematosus: a multicenter cohort report of clinical patterns and outcome

#### Wafaa Suwairi^1^, Reem Abdwani^2^, Abdulaziz Al Rowais^3^, Jubran Al qanatish^1^, Abdulrahman Al Asiri^3^

##### ^1^Pediatrics, King Abdullah specialized children hospital, Riyadh, Saudi Arabia; ^2^Pediatrics, Sultan Qaboos University Hospital, Maskat, Oman; ^3^Pediatrics, Prince Sultan Military Medical City, Riyadh, Saudi Arabia

###### **Presenting author:** Wafaa Suwairi

**Introduction:** Pulmonary manifestations may be the initial and/or life-threatening complication of childhood onset systemic lupus erythematosus (cSLE). Although pulmonary involvement is relatively frequent in adult patients; it has been infrequently reported in children with SLE.

**Objectives:** To describe the frequency of pleuropulmonary manifestation and outcome of Arab children with cSLE.

**Methods:** This is a multicenter retrospective study that included all patients diagnosed with SLE between the period of 2000 and 2015. All our patients fulfilled at least four of the 1997 revised American College of Rheumatology classification criteria. Using a standardized form, we obtained data from medical records including age of disease onset, disease duration, sex, clinical manifestations of SLE, and details of pleuropulmonary involvement, treatment and outcome

**Results:** A total of 110 patients with cSLE were included from 3 centers in 2 Arab countries; (n = 65)59% from Sultanate of Oman and (n = 45) 41% from Saudi Arabia. The mean range of follow up is 4.6 years (range 1-10 years). Pleuro-pulmonary involvement was observed in (n = 27) 24.5% of the cases; diffuse alveolar hemorrhage (DAH) (n = 13) 12%, serositis (n = 11) 10% pulmonary infection (n = 8) 7%, interstitial pneumonitis (n = 4) 3.6% and shrinking lung syndrome (n = 1) 0.9%. Outcome of patients included mechanical ventilation (n = 8) 7.0% and death (n = 1) 0.9% in a patient with both DAH and catastrophic anti-phsopholipid syndrome (CAPS). The demographic, clinical manifestations, disease activity treatment and outcome of patients with DAH in our patient cohort will be described in **Conclusion:** DAH is a rare pleuro-pulmonary manifestation in cSLE, however, in an Arab cohort it was the most common clinical manifestaion. However, despite its major impact on morbidity and mortality, our patients had an overall good outcome with aggressive treatment and supportive care. The cause of the differences in the clinical expression of SLE and outcome around the world is yet to be determined; however it can be due to interplay between genetic, environmental and ethnic factors.

**Disclosure of Interest**: None Declared

**Table 9 (abstract P445).** See text for description

Demographics	
- · Gender (M:F)	8:5
- · Mean age of onset	5.5 years (range 1.5 to 11 years)
- · Follow up duration	4.6 years
Clinical Symptoms at presentation	
- · Cough	(n = 9) 70%
- · Dyspnea	(n = 13) 100%
- · Anemia	(n = 13) 100%
- · Hemoptysis	(n = 5) 35%
- · CXR infiltrates	(n = 13) 100%
Associated SLE Clinical features	
- · Hematological	(n = 7) 54%
- · Nephritis	(n = 11) 85%
- · Arthritis	(n = 11) 85%
- · Mucocutanous	(n = 12) 92%
- · Serositis	(n = 7) 54%
- · Neuropsychiatric	(n = 5)35%
Treatment:	
- · IVM	(n = 13) 100%
- · CYC	(n = 9) 70%
- · MMF	(n = 7) 54%
- · IVIG	(n = 9) 70%
- · Plasmapharesis	(n = 6) 46%
- · Mechanical ventilation	(n = 7) 54%
Outcome:	
- · Acute DAH outcome	
Death / Survival	(n = 1) 7%
- · Long term SLE outcome	(n = 11)
Remission	(n = 7) 64%
Minimal disease activity	(n = 2)18%
Lost to follow up	(n = 2) 15%

### P446 Systemic lupus erythematosus and thrombotic microangiopathy, on account of two cases

#### Kubra Ozturk, Zelal Ekinci

##### Department of Pediatrics Rheumatology, Kocaeli University Faculty of Medicine, Kocaeli, Turkey

###### **Presenting author:** Zelal Ekinci

**Introduction:** Thrombotic microangiopathy (TMA) is the term used for diseases in which disseminated microthrombi composed of agglutinated platelets are occluding arterioles and capillaries. It can present in approximately 2% of the patients with SLE.

**Objectives:** In this report, it is aimed to emphasize the occurrence of TMA during diagnosis and follow up course of SLE in two children.

**Methods: Case 1**: A 15-year old male presented with arthralgia, weakness and fever. Physical examination was unremarkable. Laboratory workup revealed: pancytopenia (white blood cells 2.41x103/uL, platelets 163 x103/uL, haemoglobin 6.9 g/dl), increased level of serum creatinine (1.09 mg/dl) and LDH (463 IU/l). Peripheral blood smear revealed schistocytes. Due to decreased level of haptoglobin (<8mg/dl), patient was hospitalized with the diagnosis of TMA. Besides, the ADAMTS13 activation was low (<%0.2 [40–130]) and ADAMTS13 inhibitor level was high (51 U/mL [<15]). Pancytopenia, proteinuria, decreased complement C3 (32 mg/dl) and C4 (5.3 mg/dl), positive ANA and anti-dsDNA demonstrated that TMA was resulted from SLE. The kidney biopsy was consistent with class IV lupus nephritis. He was treated with 1 gr/day intravenous methylprednisolone for three days and 1 gr/m2 cyclophosphamide. Despite this treatment, microangiopathic hemolysis and trombocytopenia persisted and thus plasma exchange with fresh frozen plasma (FFP) was initiated. After 27 sessions of plasma exchange and treatment with prednisolone and mycophenolate mofetil, he improved remarkably with the normalisation of his platelet count.

**Results: Case 2**: A 7-year old male was admitted with the complaining of weakness, headache, arthralgia and rash in hands. He has been followed up with the diagnosis of SLE due to complement C1q deficiency for 3 years and he was treated with hydroxychloroquine and FFP infusion twice a month. Physical examination revealed discoid rashes in his face, hands and feet. Besides he had petechiae and purpura at his toe. Laboratory tests revealed: pancytopenia (white blood cells 2.11x103/uL, platelets 45.3 x103/uL, haemoglobin 7.07 g/dl) and decreased haptoglobin level (<8mg/dl). Additionally peripheral blood smear revealed schistocytes. When checking for the TMA selective diagnosis, it was seen that the ADAMTS13 activation was low (% 30 [40–130]) and ADAMTS13 inhibitor level was high (69 U/mL [<15]). As a result, it was confirmed that TMA was developed because of SLE. The treatment was started with intravenous methylprednisolone 30 mg/kg/day for 3 days and daily FFP infusion. Despite this treatment, hemolysis and trombocytopenia persisted and plasma exchange was initiated. After 6 sessions of plasma exchange, he improved remarkably. He was discharged with weekly FFP infusion, oral steroid and azathioprine.

**Conclusion:** SLE is a prototypic systemic autoimmune disease with heterogeneous clinical features. This report is presented to emphasize the similarities and the differences between the two cases, one of which referred with TMA symptoms and diagnosed as SLE and the other developed TMA during the course of SLE. It is important to look for laboratory findings of TMA during diagnosis and follow up course of SLE.

**Disclosure of Interest**: None Declared

## Poster Session: Uveitis

### P447 The evaluation of efficacy of adalimumab in juvenile idiopathic arthritis-associated uveitis and chronic anterior uveitis

#### Ekaterina Gaidar, Mikhail Kostik, Margarita Dubko, Vera Masalova, Elena Serogodskaya, Ludmila Snegireva, Tatyana Nikitina, Vyacheslav Chasnyk, Olga Kalashnikova, Evgenia Isupova

##### Saint-Petersburg State Pediatric Medical University, Saint-Petersburg, Russian Federation

###### **Presenting author:** Ekaterina Gaidar

**Introduction:** Juvenile idiopathic arthritis (JIA) - associated uveitis (JIA-U) and chronic anterior uveitis (CAU) are both immune-mediated chronic eye diseases, which can lead to severe impairment of visual function and blindness. Only about 75% of patients with JIA-U and CAU can reach the remission on methotrexate treatment. The rest require TNF-a inhibitors, especially adalimumab (ADA).

**Objectives:** The aim of our study was to evaluate the possibility of ADA to induce remission of uveitis, and the duration of remission and flares.

**Methods:** 39 children with JIA-U (n = 36) and CAU (n = 3) treated with ADA were included in the present[1] retrospective study. The diagnosis of JIA was made with ILAR criteria. Each patient was examined by an experienced ophthalmologist. The visits were scheduled depending upon the uveitis course. Patients could only be included in the study if they were under observation for at least 1 year before the ADA initiation. The reasons for ADA treatment were active uveitis or severe complicated uveitis course (n = 30) and active arthritis with inactive uveitis (n = 9). The median onset age of the disease was 2.9 (2.0-6.0) years, onset age of uveitis was 5.0 (3.2-7.7) years. According to the joint involvement, patients had oligoarthritis (59.1%), polyarthritis (23.1%), enthesitis-related arthritis (10.2) and no arthritis - CAU (7.7%). In 74.6% of patients arthritis occurred before uveitis, girls were 64.1%[2], ANA positivity was detected in 58.3% children. Three types of uveitis were detected: anterior (76.9%), peripheral (5.9%) and panuveitis (18.0%). The median involved eyes[3] were 2.0 (1.0-2.0). Patients with acute symptomatic uveitis were not included in the study.

**Results:** The initial uveitis remission in a patient with active uveitis was detected in 29/30 patients (96.7%) in 2.0 (2.0-12.0) weeks, only 1 still had active uveitis. Flares were experienced by 11 patients (28.2%) in the 28 (12.9-68.6) weeks: 2/11 patients were inactive before ADA and 9/11 had previously remission on ADA. The frequency of flares significantly decreased (p = 0,007) after ADA starting from 4.0 (1.0-9.0) flares/year per patient to 0.0 (0.0-1.0) and decreased the number of patients who received topical steroids from 17/28 (60.7%) to 2/28 (7.1%, p = 0.00001).

We have not found the difference in time between the start of ADA and the remission, or in the probability to achieve remission depending on gender (p = 0.22), first joint or eye involvement (p = 0.59), ANA-status (p = 0.8), type of uveitis (p = 0.5), articular course (p = 0.25), concomitant DMARD (p = 0.85) and previous biologic treatment (p = 0.18).

**Conclusion:** ADA can induce the fast and prolonged remission of JIA-U and CAU. ADA treatment is a good therapeutic option for patients with uveitis, refractory to non-biologic DMARD. Further randomized control trials required.

**Disclosure of Interest**: None Declared

### P448 Retrospective study evaluating treatment decision and outcome of non-juvenile idiopathic arthritis (JIA)-associated childhood uveitis

#### Elham Sardar^1^, Perrine Dusser^1^, Antoine Rousseau^2^, Marc Labetoulle^2^, Emanuel Barreau^2^, Bahram Bodaghi^3^, Isabelle Kone-Paut^1^

##### ^1^Paediatric Rheumatology, Paris-Sud University Hospital, APHP, Paris, France; ^2^Ophtalmology, Paris-Sud University Hospital, APHP, Paris, France; ^3^Ophtalmology, Pitie-Salpetriere Hospital,University of Paris VI, Paris, France

###### **Presenting author:** Elham Sardar

**Introduction:** Non-infectious uveitis is the most common cause of blindness in children. Most studies have evaluated diagnosis criteria and treatment of JIA-associated uveitis.

**Objectives:** We analyzed treatment, ocular complications and outcome of childhood uveitis related to other causes than JIA.

**Methods:** A retrospective chart review of pediatric uveitis in patients <16y-old seen between 2005 and 2015 at a university hospital in Paris. We excluded patients with JIA-associated uveitis. Clinical characteristics, treatment and ocular complications were collected. We used the SUN (Standardization of Uveitis Nomenclature) for the classification of uveitis, disease activity (inactive uveitis: rare cells or less) and treatment endpoints, improvement: defined as a 2-step decrease in the inflammation level or a decrease to grade 0, worsening: defined as a 2-step increase in the inflammation level or an increase from grade 3+ to 4+, or remission (inactivity without treatment for ≥ 3 months).

**Results:** Sixty patients (SR M/F: 0, 42; 107 eyes) were enrolled. Mean age at diagnosis was 10 ± 3.5 years (3 to 15 y). Mean follow-up was 4 ± 3 years. Uveitis was symptomatic in 90% of patients: eye redness (30%), decreased vision (30%) and eye pain (20%). Main diagnoses were: idiopathic (55%), Behçet’s disease (BD) (15%), sarcoidosis (7%), infectious (7%), Blau (3%) Vogt-Koyanagi– Harada syndrome (2%), and Tubulointerstitial nephritis and uveitis (TINU) syndrome (2%). Among idiopathic uveitis (n = 59 eyes), 31% were anterior and 31% were posterior. Thirty four eyes (58%) received systemic corticosteroid as first-line therapy, inducing inactivity for 10 eyes (29%) and remission for 1 eye (3%). DMARDs were needed for 21 eyes (62%). Methotrexate was used as second-line therapy in 18 eyes (53%) with 5% (n = 1 eye) and in 33% resulting in inactivity (n = 6 eyes). Infliximab was used as third-line treatment in 9 eyes with 80% of inactivity but no remissions. Ocular complications were present in 47% (n = 28) at diagnosis and they increased until 61% of patients during the follow-up. The most common were posterior synechiae (32%), macular edema (30%), cataract (20%), and band keratopathy (12%). In BD uveitis (n = 16 eyes), 56% had panuveitis. Twelve eyes received systemic corticosteroids therapy at first-line and all needed azathioprine as second-line. Azathioprine allowed (n = 4) 33% of inactivity and 16% (n = 2) of remission. Infliximab was used as third-line therapy in 7 eyes with 85% of inactivity. Thirty percent of BD patients presented complications at the beginning until 63% during the follow-up: posterior synechiae (31%), vitreous hemorrhage (25%), cataract (25%) ischemia macular (19%) and macular edema (19%). Sarcoidosis affected 5 eyes, 80% were panuveitis and all patients received systemic corticosteroids and became steroid dependent. All these patients received methotrexate as second-line therapy,which allowed 50% of improvement. Forty percent required infliximab as a third-line therapy with remission in 1 eye (20%) and inactivity in 3 eyes (60%). Posterior synechiae were present in 1 eye at the beginning and 3 eyes at the end of the follow up (60%).

**Conclusion:** Idiopathic uveitis is the most common cause of non-JIA uveitis in children and seems more severe at presentation. BD and Sarcoidosis appeared more difficult to treat because the complication rate increased during the follow-up despite treatment. Most patients required a DMARD in association with infliximab. Improvement and disease inactivity were frequently obtained but remission was rare.

**Disclosure of Interest**: None Declared

### P449 Proposal for a damage index for juvenile idiopathic arthritis related uveitis from the Multinational Interdisciplinary Working Group for Uveitis in Childhood Group (MIWGUC)

#### Ivan Foeldvari^1^, Jordi Anton^2^, Rosa Bou^2^, Sheila Angeles-Han^3^, Regitze Bangsgaard^4^, Gabriele Brumm^5^, Tamas Constantin^6^, Clive Edelsten^7^, Jens Klotsche^8^, Kirsten Minden^8^, Elisabetta Miserocchi^9^, Susan Nielsen^4^, Gabriele Simonini^10^, Arnd Heiligenhaus^11^

##### ^1^AM Schön Klinik Eilbek, Hamburg, Germany; ^2^Universitat de Barcelona, Hospital Sant Joan de Deu, Barcelona, Spain; ^3^Emory University, Atlanta, GA, United States; ^4^Juliane Marie Centret, Rigshospitalet, Copenhaven, Denmark; ^5^Universitätsklinikum Hamburg-Eppendorf, Hamburg, Germany; ^6^Semmelweis University, Budapest, Hungary; ^7^Great Ormond Street Hospital for Children NHS Foundation Trust, London, United Kingdom; ^8^German Rheumatism Research Center, Berlin, Germany; ^9^Università Vita-Salute, Scientific Institute San Raffaele, Milano, Italy; ^10^Universita degli studi Firenze, Firenze, Italy; ^11^St. Franziskus Hospital, Muenster, Germany

###### **Presenting author:** Ivan Foeldvari

**Introduction:** Juvenile idiopathic arthritis (JIA) associated Uveitis is the most common extraarticulare comorbidity of juvenile idiopathic arthritis. Nowdays it occurs about 10-15% of JIA patients. As innovative effective treatment options are emerging, it is extremely important for defining validated damage index in order to assess the effectivity of drugs to preventing damage, one of the main aims of the treatment.

**Objectives:** To develop a proposal for the damage index, that asses the effectivity of a given drug for preventing damage in JIA associated uveitis.

**Methods:** Multinational Interdisciplinary Working Group for Uveitis in Childhood Group (MIWGUC) had prospectively evaluated the validity of the proposed outcome measures(1). Based on the data, we proposed a damage index using the nominal group technique in a consensus meeting in Barcelona, Spain, in November 2015.

**Results:**

Following items were selected for assessing damage:

Vision related permanent damage per eye /per patient - yes / no

**Right eye Left eye**

1. flare
2. synechiae
3. cataract
4. maculopathy
5. Opticopathy
6. Decreased Visual acuity
7. Ocular hypertony – >21 mmHg
8. Ocular hypotony - <6 mmHg
9. Glaucomatous field loss and /or glaucomatous optic atrophy
10. Band-keratopathy
11. Epiretinal membrane formation
12. Visual deterioration – less then 0.3 in any eye
13. Uveitis related disability VAS 0-100 by ophthlamologist
14. Uveitis related disability VAS 0-100 by pediatric rheumatologist

**Conclusion:** We proposed items for assessing the damage index of JIA associated uveitis. The damage index should be a valid instrument for assessing the effectivity of a given drug in order to preventing damage. This proposal will be evaluated from the MIWGUC group in prospective study.

**References:**

1. Heiligenhaus A, Foeldvari I, Edelsten C, Smith JR, Saurenmann RK, Bodaghi B, et al. Proposed outcome measures for prospective clinical trials in juvenile idiopathic arthritis-associated uveitis: a consensus effort from the multinational interdisciplinary working group for uveitis in childhood. Arthritis Care Res (Hoboken). 2012;64(9):1365-72.

**Disclosure of Interest**: None Declared

### P450 Proposal for the definition of remission for juvenile idiopathic arthritis related uveitis from the Multinational Interdisciplinary Working Group for Uveitis in Childhood Group (MIWGUC)

#### Ivan Foeldvari^1^, Jordi Anton^2^, Rosa Bou^2^, Sheila Angeles-Han^3^, Regitze Bangsgaard^4^, Gabriele Brumm^5^, Tamas Constantin^6^, Clive Edelsten^7^, Jens Klotsche^8^, Kirsten Minden^8^, Elisabetta Miserocchi^9^, Susan Nielsen^4^, Gabriele Simonini^10^, Arnd Heiligenhaus^11^

##### ^1^AM Schön Klinik Eilbek, Hamburg, Germany; ^2^Universitat de Barcelona, Hospital Sant Joan de Deu, Barcelona, Spain; ^3^Emory University, Atlanta, GA, United States; ^4^Juliane Marie Centret, Rigshospitalet, Copenhaven, Denmark; ^5^Universitätsklinikum Hamburg-Eppendorf, Hamburg, Germany; ^6^Semmelweis University, Budapest, Hungary; ^7^Great Ormond Street Hospital for Children NHS Foundation Trust, London, United Kingdom; ^8^German Rheumatism Research Center, Berlin, Germany; ^9^Università Vita-Salute, Scientific Institute San Raffaele, Milano, Italy; ^10^Universita degli studi Firenze, Firenze, Italy; ^11^St. Franziskus Hospital, Muenster, Germany

###### **Presenting author:** Ivan Foeldvari

**Introduction:** Juvenile idiopathic arthritis (JIA) associated Uveitis is the most common extraarticulare comorbidity of juvenile idiopathic arthritis. Nowdays it occurs about 10-15% of JIA patients. As effective treatment options are emerging, validated definition criteria of of remission are warranted, which is main aim of the treatment.

**Objectives:** To develop a proposal for the definition of remission JIA-associated uveitis.

**Methods:** Multinational Interdisciplinary Working Group for Uveitis in Childhood Group (MIWGUC) had prospectively evaluated the validity of the proposed outcome measures (1). Based on the data, we proposed a definition of JIA associated uveitis remission using the nominal group technique in a consensus meeting in Barcelona, Spain, in November 2015.

**Results:** The following items were selected to define remission of JIA associated uveitis on medication or off medication. It is required, that in both eyes the following condition is reached and the condition last at least 6 months on medication, or inactive disease for ≥ 3 months after discontinuing all treatments.

1. Slit lamp total number of AC cells: no inflammatory cells

*In aphakic patients some cells may be present in the anterior vitreous

2. Absence of optic disc edema

*The presence of isolated optic disc edema may not be a sign of activity

3. Absence of macular edema

*The presence of isolated macular edema may not be a sign of activity

4. Absence of vitreous haze

*The presence of isolated vitreous haze may not be a sign of activity

5. VAS score of activity by the physician 0-100: must be 0

**Conclusion:** We proposed items to define remission of in JIA associated uveitis, which is the major goal of treatment. This proposal will be validated from the MIWGUC group in prospective study.

**References:**

1. Heiligenhaus A, Foeldvari I, Edelsten C, Smith JR, Saurenmann RK, Bodaghi B, et al. Proposed outcome measures for prospective clinical trials in juvenile idiopathic arthritis-associated uveitis: a consensus effort from the multinational interdisciplinary working group for uveitis in childhood. Arthritis Care Res (Hoboken). 2012;64(9):1365-72.

**Disclosure of Interest**: None Declared

### P451 Proposal for the definition of inactivity of juvenile idiopathic arthritis related uveitis from the Multinational Interdisciplinary Working Group for Uveitis in Childhood Group (MIWGUC)

#### Ivan Foeldvari^1^, Jordi Anton^2^, Rosa Bou^2^, Sheila Angeles-Han^3^, Regitze Bangsgaard^4^, Gabriele Brumm^5^, Tamas Constantin^6^, Clive Edelsten^7^, Jens Klotsche^8^, Kirsten Minden^8^, Elisabetta Miserocchi^9^, Susan Nielsen^4^, Gabriele Simonini^10^, Arnd Heiligenhaus^11^

##### ^1^AM Schön Klinik Eilbek, Hamburg, Germany; ^2^Universitat de Barcelona, Hospital Sant Joan de Deu, Barcelona, Spain; ^3^Emory University, Atlanta, GA, United States; ^4^Juliane Marie Centret, Rigshospitalet, Copenhaven, Denmark; ^5^Universitätsklinikum Hamburg-Eppendorf, Hamburg, Germany; ^6^Semmelweis University, Budapest, Hungary; ^7^Great Ormond Street Hospital for Children NHS Foundation Trust, London, United Kingdom; ^8^German Rheumatism Research Center, Berlin, Germany; ^9^Università Vita-Salute, Scientific Institute San Raffaele, Milano, Italy; ^10^Universita degli studi Firenze, Firenze, Italy; ^11^St. Franziskus Hospital, Muenster, Germany

###### **Presenting author:** Ivan Foeldvari

**Introduction:** Juvenile idiopathic arthritis (JIA) associated uveitis is the most common extraarticulare comorbidity of juvenile idiopathic arthritis. Nowdays it occurs in about 10-15% of JIA patients. As effective treatment options are emerging, is it extremely important to propose validated definition of inactivity, which is main aim of the treatment.

**Objectives:** To develop a proposal for the definition of inactivity.

**Methods:** Multinational Interdisciplinary Working Group for Uveitis in Childhood Group (MIWGUC) had prospectively evaluated the validity of the proposed outcome measures (1). Based on these data, we proposed a definition of inacitivity using the nominal group technique in a consensus meeting in Barcelona, Spain, in November 2015.

**Results:** The following items were selected to define inacitivity. It is required, that in both eyes the following condition is reached:

1. Slit lamp total number of AC cells: 0 inflammatory cells

*In aphakic patients,some cells may be present, in the anterior vitreous

2. Absence of Optic disc edema

*The presence of isolated optic disc edema may not be a sign of activity

3. Absence Macular edema

*The presence of isolated macular edema may not be a sign of activity

4. Absence of Vitreous haze

*The presence of isolated vitreous haze may not be a sign of activity

5. VAS score of activity physician 0-100: must be 0

**Conclusion:** We proposed items to define inactivity of JIA associated uveitis, which is a major goal of treatment. This proposal will be validated from the MIWGUC group in prospective study.

**References:**

1. Heiligenhaus A, Foeldvari I, Edelsten C, Smith JR, Saurenmann RK, Bodaghi B, et al. Proposed outcome measures for prospective clinical trials in juvenile idiopathic arthritis-associated uveitis: a consensus effort from the multinational interdisciplinary working group for uveitis in childhood. Arthritis Care Res (Hoboken). 2012;64(9):1365-72.

**Disclosure of Interest**: None Declared

### P452 Proposal for a response index for juvenile idiopathic arthritis related uveitis from the Multinational Interdisciplinary Working Group for Uveitis in Childhood Group (MIWGUC)

#### Ivan Foeldvari^1^, Jordi Anton^2^, Rosa Bou^2^, Sheila Angeles-Han^3^, Regitze Bangsgaard^4^, Gabriele Brumm^5^, Tamas Constantin^6^, Clive Edelsten^7^, Jens Klotsche^8^, Kirsten Minden^8^, Elisabetta Miserocchi^9^, Susan Nielsen^4^, Gabriele Simonini^10^, Arnd Heiligenhaus^11^

##### ^1^AM Schön Klinik Eilbek, Hamburg, Germany; ^2^Universitat de Barcelona, Hospital Sant Joan de Deu, Barcelona, Spain; ^3^Emory University, Atlanta, GA, United States; ^4^Juliane Marie Centret, Rigshospitalet, Copenhaven, Denmark; ^5^Universitätsklinikum Hamburg-Eppendorf, Hamburg, Germany; ^6^Semmelweis University, Budapest, Hungary; ^7^Great Ormond Street Hospital for Children NHS Foundation Trust, London, United Kingdom; ^8^German Rheumatism Research Center, Berlin, Germany; ^9^Università Vita-Salute, Scientific Institute San Raffaele, Milano, Italy; ^10^Universita degli studi Firenze, Firenze, Italy; ^11^St. Franziskus Hospital, Muenster, Germany

###### **Presenting author:** Ivan Foeldvari

**Introduction:** Juvenile idiopathic arthritis (JIA) associated Uveitis is the most common extraarticulare comorbidity of juvenile idiopathic arthritis. Nowdays it occurs in about 10-15% of the JIA patients. As inniovativ effective treatment options are emerging, therefore is it extremely important for defining a validated response index in order to assess the effectivity of a drug.

**Objectives:** To develop a proposal for the response index, that assesses the effectivity of a given drug for treating JIA associated uveitis.

**Methods:** Multinational Interdisciplinary Working Group for Uveitis in Childhood Group (MIWGUC) had prospectively evaluated the validity of the proposed outcome measures(1). Based on the data, we proposed a response index using the nominal group technique in a consensus meeting in Barcelona, Spain, in November 2015.

**Results:** The following items were selected for defining response to treatment:

1. Slit lamp evaluation : total number of anterior chamber cells (AC) – expressed on a VAS 0- 100

2. Slit lamp evaluation: AC cells before pupil dilatation

3. Grade of AC flare according SUN criteria

4. Change in visual acuity – without operation – (provided in logMAR, independently of the charts used)

5. Occurrence and course of structural complications:

a. occurrence new posterior synechia

b. Change of Optic disc edema

c. Change of Macular edema

d. Change of Vitreous haze

6. VAS score of 0 to 100 score for uveitis activity in the worst eye in the last 4 weeks (assessed by ophthalmologist)

7. Has your eyes caused a problems in the last 4 weeks?

expressed on a VAS score of 0-100

(assessed by patients / or under the age of 8 years by the parents)

8. How do you judge your eye disease during the last 3 months?

Lot better / something better/ stable/ something worst/ lot worst

(assessed by patients / or under the age of 8 years by the parents)

9. Change of the score of the quality of life in an Uveitis specific quality of life instrument research item

10. How did you eye improved under treatment at the last three months? Expressed VAS score of 0-100

11. Missed work/school /kindergarten days– due of the uveitis

12. Change in CHQ/PedsQl score

**Conclusion:** We proposed items to define a response index for JIA associated uveitis. This should be an important instrument for evaluating effectivity of the treatment. This proposal will be validated from the MIWGUC group in prospective study.

**References:**

1. Heiligenhaus A, Foeldvari I, Edelsten C, Smith JR, Saurenmann RK, Bodaghi B, et al. Proposed outcome measures for prospective clinical trials in juvenile idiopathic arthritis-associated uveitis: a consensus effort from the multinational interdisciplinary working group for uveitis in childhood. Arthritis Care Res (Hoboken). 2012;64(9):1365-72.

**Disclosure of Interest**: None Declared

### P453 National prospective online registry for juvenile idiopathic arthritis associated uveitis in Spain

#### Juan Manuel Mosquera Angarita^1^, Rosa Bou^1^, Carmen Garcia de Vicuña^1^, Maria Victoria Hernandez^2^, Alfredo Adan^2^, Victor Llorens^2^, Rosa Alcobendas^3^, Susana Noval^3^, Juan Carlos Lopez Robledillo^4^, Isabel Valls^4^, Mari Carmen Pinedo^5^, Alejandro Fonollosa^5^, Jaime de Inocencio^6^, Pilar Tejada^6^, Beatriz Bravo^7^, Manuel Torribio^7^, María Jesús García de Yebenes^8^, Jordi Antón^1^; Uveitis Working Group of the Spanish Pediatric Rheumatology Society

##### ^1^Hospital Sant Joan de Déu, Barcelona, Spain; ^2^Hospital Clínic, Barcelona, Spain; ^3^Hospital La Paz, Madrid, Spain; ^4^Hospital Niño Jesus, Madrid, Spain; ^5^Hospital Cruces, Bilbao, Spain; ^6^Hospital 12 de Octubre, Madrid, Spain; ^7^Hospital Virgen de las Nieves, Granada, Spain; ^8^Instituto de Salud Musculoesqueletica, Madrid, Spain

###### **Presenting author:** Juan Manuel Mosquera Angarita

**Introduction:** Anterior uveitis is the most frequent extraarticular manifestation of juvenile idiopathic arthritis (JIA). However, there is a lack of standardization in its follow-up and management. Disease registries are powerful tools for evaluation and feedback on medical care quality, and become the base for recommendations on disease management.

**Objectives:** To design an online multicentric prospective registry for JIA-uveitis in Spain, establish quality indicators and propose improvements in the follow-up and management of this disease.

**Methods:** Through different consensus meetings, pediatric rheumatologists and ophthalmologists members of the uveitis working group of the Spanish Pediatric Rheumatology Society (SERPE), designed a data collection sheet. A centered electronic database with intellectual property of SERPE was created following data safety regulation. The inclusion criteria for the registry were a known diagnosis of JIA and active uveitis at the moment of inclusion. The registry was open to the different centers in Spain willing to participate and a first data analysis was performed in February 2016. The project was approved by the local ethics committee and patients and families provided a signed informed consent.

**Results:** After a trial period, the online registry platform was launched in February 2015. 13 centers in Spain have applied to participate and in February 2016, seven centers have included a total of 91 patients from whom 331 registries were collected. Those registries correspond to: 1) Basal data (demographic, arthritis and uveitis characteristics), 2) Prospective rheumatology visits and 3) Prospective ophthalmology visits. We present the preliminary results from the basal data and first rheumatology and ophthalmology visits. Predominantly female gender (79%), caucasic origin (93%), positive ANA test (82%) and persistent oligoarticular JIA subtype (68%). Mean age at onset of arthritis symptoms is 3.3 ± 2.8 years, mean age at JIA diagnosis is 3.8 ± 3.2 years and mean age at first uveitis diagnosis is 5.8 ± 5.3 years. At first rheumatology visit the average of active arthritis was 0.4, the mean value in the global disease visual analogical scale (VAS) was 17.1 mm for the rheumatologist, 16.2 for parents and 12.2 for children; and the VAS for uveitis was 28.9 mm for the rheumatologist and 24.3 for the ophthalmologist. At first ophthalmology visit 95% were anterior uveitis and around 85% were asymptomatic. Measurement of cellularity grading was 1+ or less according SUN classification in about 70%. Mean logMAR visual acuity was 0.14 in right eye and 0.23 in left eye. Main complication was band keratopathy (21%) and methotrexate was the most frequent used medication at the first visit (50%) followed by adalimumab (32%)

**Conclusion:** Our group has managed to launch a prospective online multicentered registry of JIA associated uveitis obtaining a representative sample of patients of whom we present the preliminary data. Future steps will be the analysis of prospective data and the development of recommendations to achieve the best standards and quality indicators in JIA-uveitis. This registry represents a first but firm step for standardization of diagnosis, follow-up and management of the disease.

This project was partially funded by an unrestricted grant from Abbvie

**Disclosure of Interest**: None Declared

### P454 Anti-DFS70 antibodies: new biomarkers for uveitis in juvenile idiopatic arthritis?

#### Lorenza Maria Argolini^1^, Irene Pontikaki^1^, Maria Orietta Borghi^2^, Laura Cesana^2^, Elisabetta Miserocchi^3^, Barbara Castiglioni^4^, Maurizio Gattinara^1^, Pierluigi Meroni^1^

##### ^1^Rheumatology, Gaetano Pini Institute, Center of Pediatric Rheumatology, Chair of Rheumatology, University of Milan, Milan, Italy; ^2^Experimental Laboratory of ImmunoRheumatologic Researches, Department of Clinical Sciences and Community Health, University of Milan, IRCCS Istituto Auxologico Italiano, Milan, Italy; ^3^Ophthalmology and Visual Sciences, University Vita-Salute, Scientific Institute San Raffaele, Milan, Italy; ^4^Ophtometrist, Department of Rheumatology, JIA Uveitis Center, Gaetano Pini Institute, Milan, Italy

###### **Presenting author:** Lorenza Maria Argolini

**Introduction:** Uveitis is the most frightening extraarticular manifestation of juvenile idiopathic arthritis (JIA), if undiagnosed and untreated can lead to blindness. Historically antinuclear antibodies (ANAs) are associated with an increased risk of uveitis however as biomarkers they have a limited specificity because they are also seen in JIA without uveitis and the molecular target is still unknown. Anti-DFS70 antibodies are defined by a nuclear dense fine speckled (DFS) indirect immunofluorescence (IIF) pattern, first described in 1994. The main antigen recognized by these autoantibodies was identified as the lens epithelium-derived growth factor (LEDGF), also known as DFS70. This finding suggested that these antibodies may display a role in ocular diseases. Accordingly two recent studies described these antibodies n JIA patients with uveitis (H. Schmeling et al, J Rheumatology 2015; Vol 42, no.12; SL. Tansley et al, Rheumatology Vol 55 Suppl 1, april 2016).

**Objectives:** To investigate whether DFS70-like pattern may correlate with uvetis in a monocentric cohort of JIA patients.

**Methods:** Anti-DFS70 antibodies were investigated in 51 JIA ANA-positive patients [31 with uveitis (JIA-U) and 20 without uveitis as controls] by Nova-Lite HEp-2 Select providing a DFS70-enriched sample diluent, that allows the specific absorption of anti-DFS70 antibodies. The sera were tested by IIF before and after absorption and inhibition values higher than 30% were considered positive for the presence of anti-DFS70. Furthermore, ANA screening was also performed by QUANTA Flash CTD Screen Plus, a chemiluminescent immunoassay which includes the most relevant nuclear/cytoplasmic antigens but not DFS70 (dsDNA, Sm/RNP, Ro52, Ro60, SS-B, Scl-70, centromere, Mi-2, Ku, ThTo, RNAPol III, Pm/Scl, PCNA, Jo-1 and ribosomal-P protein).

**Results:** Before absorption, 26/31 (84%) JIA-U patients showed a DFS/homogeneous pattern. After the exposure to DFS70, 9/31 (29%) sera displayed an inhibition suggestive for anti-DFS70 positivity (>30%). In the JIA control group, 14/20 (70%) sera, displayed a DFS/homogenous pattern. However, only 3 of them (15%) were inhibited by the absorption. The CTD Screen was positive in 6/31 (19%) JIA-U and in 3/20 (15%) controls. Only one of these samples was significantly reduced after exposure to DFS70.

**Conclusion:** DFS/homogenous is the prevalent pattern in our ANA-positive JIA cohort, mainly in JIA-U. These data are in agreement with recent studies supporting a relationship between DFS/homogenous pattern and the risk of developing uveitis. The absorption with DFS70 suggests the presence of anti-DFS70 antibodies, that seems more frequent in JIA-U. However, as the DFS70-induced reduction of fluorescence intensity is only partial, a reactivity against molecules not yet identified may be suggested, in association with a DFS-like pattern. This hypothesis is also supported by CTD Screen results. Further studies aimed to identify new antibody specificities by additional techniques are needed to deeply investigate the ANA-associated risk of uveitis in JIA.

**Disclosure of Interest**: None Declared

### P455 Adjuvite: a double-blind, randomized, placebo-controlled trial of Adalimumab in juvenile idiopathic arthritis associated uveitis

#### Pierre Quartier^1^, Veronique Despert^2^, Sylvaine Poignant^3^, Amandine Baptiste^4^, Caroline Elie^4^, Isabelle Kone-Paut^5^, Alexandre Belot^6^, Laurent Kodjikian^7^, Dominique Monnet^8^, Michel Weber^9^, Bahram Bodaghi^10^

##### ^1^Unité d’Immuno-Hématologie et Rhumatologie Pédiatrique, Hôpital Necker-Enfants Malades, Paris, France; ^2^Pediatrie, CHU, Rennes, France; ^3^Pédiatrie, CHU, Nantes, France; ^4^Biostatistiques, Hôpital Necker-Enfants Malades, Paris, France; ^5^Pédiatrie, CHU, Kremlin-Bicetre, France; ^6^Pédiatrie, Hopital femme-mere-enfant, Lyon, France; ^7^Ophtalmologie, CHU, Lyon, France; ^8^Ophtalmologie, CHU Cochin, Paris, France; ^9^Ophtalmologie, CHU, Nantes, France; ^10^Ophtalmologie, CHU Pitié Salpétrière, Paris, France

###### **Presenting author:** Pierre Quartier

**Introduction:** Patients with early-onset, oligo or polyarticular juvenile idiopathic arthritis (JIA) may develop a chronic, anterior uveitis, which treatment usually requires long-term topical steroids, sometimes systemic steroids. Methotrexate often lack of efficacy. Small series suggested that a reduction of ocular inflammation and of the risk of flare could be achieved with anti-TNF alpha antibodies

**Objectives:** To asses the saffecty and efficacty of Adalimumab in patients with JIA-associated uveitis with an inadequate response to topical steroids and methotrexate

**Methods:** Elligible patients had chronic uveitis with inadequate response to topical steroids and MTX, no previous anti-TNF antibody therapy and at least one assessable eye with inflammation quantified by laser flare photometry ≥30 photons/ms. Double-blind randomization at Day 1 (D1) into 2 equal groups, one treated with placebo and one with adalimumab (24 mg/m2 in patients aged 4 to less than 13 years, 40 mg in patients ≥ 13), every other week subcutaneous injections. The primary objective was to demonstrate a higher response rate at Month 2 (M2) in the adalimumab arm versus the placebo arm. Response was defined as a 30% reduction of inflammation on laser flare photometry in the eye with the highest flare value at D1 and improvement or a stable appearance on slit lamp examination. From M2 to M12, all patients were allowed to receive adalimumab (open phase)

**Results:** 34 patients were screened and 31 received at least one injection of study treatment.

**Conclusion:** Adalimumab was effective in reducing ocular inflammation within 2 months and well tolerated over 12 months in patients with JIA-associated chronic uveitis and an inadequate response to topical steroids and MTX. Laser flare photometry is a valuable tool to assess early response to treatment in these patients

**Trial registration identifying number: NCT01385826**

**Disclosure of Interest**: P. Quartier Grant / Research Support from: Abbvie, Novartis, Pfizer, Roche, Consultant for: Abbvie, Novartis, SOBI, Speaker Bureau of: Abbvie, BMS, Novartis, PfiZer, Roche, SOBI, V. Despert: None Declared, S. Poignant: None Declared, A. Baptiste: None Declared, C. Elie: None Declared, I. Kone-Paut Grant / Research Support from: SOBI, Novartis and Roche, Consultant for: Novartis, SOBI, Pfizer, Abbvie and Roche, A. Belot: None Declared, L. Kodjikian Consultant for: Alcom, Alimera, Allergan, Bayer, Bausch&Lomb, Novartis, Thea, Speaker Bureau of: Alcom, Alimera, Allergan, Bayer, Bausch&Lomb, Novartis, Thea, D. Monnet: None Declared, M. Weber: None Declared, B. Bodaghi Consultant for: Abbvie

**Table 10 (abstract P455).** Patients characteristics at study treatment onset

	Ada (n = 16)	Placebo (n = 15)	All (n = 31)
Female, n (%)	15 (94)	13 (87)	28 (90)
Age, median value, years [ranges	10.8 [5.0-20.3]	9.2 [4.9-29.1]	
Active joints, median n [ranges]	0 [0-3]	0 [0-4]	0 [0-4]
Uveitis median duration, years [ranges]	4.4 [0.4-18.9]	4.8 [0.6-24.2]	
Laser flare (ph/ms), median [ranges]	99 [23-322]	70 [36-265]	
Bilateral uveitis, n (%)	10 (63)	14 (93)	24 (78)
Ongoing treatments at D1			
Oral steroids, patients n (%)	7 (44)	3 (20)	10 (32)
Methotrexate, patients n (%)	15 (94)	11 (73)	26 (84)

At M2, in intention-to-treat (1ary objective), there were 9/16 responders in the adalimumab group and 3/15 in the placebo group (*p* = 0.038, Chi-squared test; RR = 2.81, CI95% = [0.94-8.45] with log-binomial model estimation). Among responders to adalimumab, patients with very high laser flare photometry values showed quick improvement. One patient stopped the trial at D14 (adalimumab group, uveitis worsening) and one at M9 (ocular hypertony). 29 patients reached M12 on adalimumab. There were 6 serious adverse events in 5 patients, all in the placebo group, 5 during the open-label phase, none related to study treatment (investigator assessment)

### P456 Paediatric uveitis in french referral ophthalmologic/rheumatologic centers: a descriptive analysis of 61 children

#### Laura Moal^1^, Antoine Rousseau^2^, LuuLy Pham^1^, Emmanuel Barreau^2^, Cherif Titah^3^, Pascal Dureau^3^, Marc Labetoulle^2^, Bahram Bodaghi^4^, Severine Guillaume Czitrom^5^

##### ^1^Paediatrics, CHU de Bicetre, Le Kremlin Bicetre Cedex, France; ^2^Ophthalmology, CHU de Bicetre, Le Kremlin Bicetre Cedex, France; ^3^Ophthalmology, Fondation Rothschild, Paris, France; ^4^Ophthalmology, CHU Pitie Salpetriere, Paris, France; ^5^Service de Medecine des Adolescents, CHU de Bicetre, Le Kremlin Bicetre Cedex, France

###### **Presenting author:** Severine Guillaume Czitrom

**Introduction:** Uveitis in children is rare; fortunately, knowledge about the different types of paediatric uveitis is growing. Intensive interactions between ophthalmologists and paediatric rheumatologists are needed in order to choose the best therapeutic strategies for, sometimes, deep uveitis attacks.

**Objectives:** To describe a cohort of 61 patients with paediatric uveitis.

**Methods:** Retrospective analysis of the cohort of patients followed in a tertiary care center by a paediatric rheumatologist (SGC) and members of 3 well known ophthalmologic departments (AR, EB, CT, PD, ML and BB) in Paris, during the 2006-2016 period.

**Results:** 61 children were diagnosed with uveitis before the age of 18 and collectively followed in 3 ophthalmologic departments specialized in uveitis care in children along with 1 paediatric rheumatologist, each time systemic treatment was to be decided. Among the 61 patients, 33 had anterior (54%, group 1), 13 had intermediate (21%, group 2), and 15 had posterior or pan-uveitis (25%, group 3). Sex ratio was equally distributed among girls and boys for groups 1 and 2, but F/M ratio was 2 in group 3. Most of the children originated from Europe but importantly, as much as 1/3 came from non-European Mediterranean countries in groups 1 and 3. JIA was the leading cause of group 1 uveitis (42%); groups 2 and 3 uveitis were idiopathic despite complete etiological work-up in 77% and 47%, respectively. In group 3, etiologies were JIA (3 with 2 SpA), Behçet (3), sarcoidosis (1) and TINU syndrome (1). At presentation, mean ages were 8.1 ± 3.6 [2-17], 9.5 ± 4.6 [4-20] and 11 ± 3.9 [4-15] years old; eyes remained white in 51%, 77%, 47%, painless in 70%, 100%, 67%, and non photophobic in 70%, 100%, 87% of group 1,2, 3, respectively. There was no correlation between the color, the pain, and the photophobia of eyes in this cohort. Bilateral eye involvement was present in 70%, 77%, 73% at onset, with a secondary bilateralisation in 9%, 8%, 13% and uveitis presentation was granulomatous in 33%, 0%, 47% in group 1, 2, 3, respectively. The mean follow-up was 4.85 ± 3.1 [0.5-16] years with no difference between groups. Complications including band keratopathy, synechiae, papillary edema, macular edema, ocular hypertension, neovascularization, chorioretinitis, neuroretinitis, retinal detachment, vitreous hemorrhage, amblyopia were observed in 58% (19/33 patients, of which 5 had more than 1 complication), 61% (8/13 patients but 4/8 had more than 1 complication), 87% (13/15 patients, of which 9 had more than 1 complication) in group 1, 2, 3, respectively. In group 1, recourse to a 3^rd^ line therapy combining at least synthetic DMARDs (MTX, AZA) and biologic DMARDs (monoclonal anti-TNFs, TCZ) was necessary in 11/33 children (33%, group 1), in 1/13 (8%, group 2), in 8/15 (53%, group 3). Surgery mainly for cataracts but also for recurrent band keratopathy and refractory glaucoma was done in 7/33 group 1 (21%), 1/13 group 2 (8%), 3/15 group 3 (20%).

**Conclusion:** Paediatric posterior and pan-uveitis induce a very high-level burden in children, but anterior uveitis as well, sometimes despite optimal therapeutic management in tertiary care centers.Tight control of uveitic children is absolutely required in order to decrease the level of definitive complications.

**Disclosure of Interest**: None Declared

### P457 Safety and efficacy of infliximab and adalimumab for refractory uveitis in juvenile idiopathic arthritis: two year follow up data from the orchidea registry

#### Vanessa Cecchin^1,2^, Maria Elisabetta Zannin^1,2^, Daniele Ferrari^2^, Francesco Comacchio^2^, Irene Pontikaki^2^, Claudia Bracaglia^2^, Rolando Cimaz^2^, Fernanda Falcini^2^, Antonella Petaccia^2^, Stefania Viola^2^, Luciana Breda^2^, Francesco La Torre^2^, Fabio Vittadello^2^, Giorgia Martini^1,2^, Francesco Zulian^1,2^

##### ^1^Department of Pediatrics, University of Padua, Padua, Italy; ^2^ORCHIDEA Network (OculaR involvement in CHIldhood rheumatic DisEAses, Padua, Italy

###### **Presenting author:** Vanessa Cecchin

**Introduction:**

The anti-tumour necrosis factor α (anti-TNFα) agents has significantly changed the management of many rheumatic conditions, included Juvenile Idiopathic Arthritis-related Chronic Anterior Uveitis (JIA-CAU) but the experience, so far, is still limited. Since 2007 a National Registry (*ORCHIDEA*), formed by Paediatric Rheumatologists and Ophthalmologists, prospectively collected online, through a protected website, data on safety and efficacy of anti-TNF-α agents used for the treatment of CAU**.**

**Objectives:** To evaluate safety and efficacy of adalimumab (ADA) and infliximab (IFX) for the treatment of JIA-CAU) in a cohort of patients followed for at least 2 years of follow-up.

**Methods:**

Patients with JIA-CAU refractory to standard immunosuppressive treatment and/or corticosteroid-dependent and treated with infliximab (IFX) or adalimumab (ADA) were managed by a standard protocol and entered the Orchidea Registry. Data, recorded every 3 months, included uveitis course, number/type of ocular complications, drug-related adverse events (AE), treatment change*.* The diagnosis and course of JIA was based upon the ILAR criteria and the diagnosis of uveitis was made according to the SUN Working Group criteria. Uveitis flare was defined as an increase of the cells in the anterior chamber of 2+ or more as compared to the baseline. Clinical remission was defined as the absence of flares for more than 6 months on treatment, without or with minimal topical treatment (corticosteroid and/or mydriatic-cycloplegic eye drops ≤ 1/day). Data of patients treated for at least 2 years were retrieved from the Orchidea Registry and analyzed using descriptive statistics.

**Results:** From January 2007 to December 2014, among 236 patients included in the Orchidea database with JIA-CAU on anti-TNF agents (IFX or ADA), 154 (125 female, 29 male) reached 2 years of follow-up and entered the study. Fifty-nine patients were treated with IFX, 95 with ADA. No major AEs were recorded; 30 patients (19.5%) experienced minor AE and 11 (7.1%) multiple AEs. The total number of minor AEs amounts to 54: 22 were related to ADA, 32 to IFX. Infections were the most frequent AE for both anti-TNF agents (37.6% for IFX and 40.9% for ADA), followed by headache for ADA (25%) and local skin reactions for IFX (27.4%). During the 2-years follow-up, 84 new-onset complications (42 with ADA and 42 with IFX) were reported. Cataract was the most common complication (18.1%) followed by band keratopathy 12.1% and synechiae 12.7%. Vitreitis was significantly less frequent in the ADA group (3.2%) than in IFX group (15.3%, p 0.006). Drug switch was reported in 35 patients (22.7%): 26 IFX to ADA, 2 ADA to IFX, 6 ADA and 1 IFX to other biological agents. In general, 129 patients (83.8%) achieved clinical remission, 89.5% with ADA and 74.6% with IFX, respectively (p = 0.015).

**Conclusion:** IFX and ADA confirm to be effective and safe for treatment of refractory JIA-related CAU after 2 years of follow-up, a higher rate of clinical remission was reported in the ADA group that presented less frequency of adverse events and lower evidence of drug switch.

**Disclosure of Interest**: None Declared

### P458 Rare NOD2 variants in patients with granulomatous uveitis

#### Caroline Galeotti^1^, Guillaume Sarrabay^2^, Olivier Fogel^3^, Isabelle Touitou^2^, Bahram Bodaghi^4^, Corinne Miceli-Richard^3^, Isabelle Koné-Paut^1^

##### ^1^Pediatric Rheumatology, Bicêtre Hospital, Le Kremlin Bicêtre, France; ^2^Genetics, CHU Montpellier, Montpellier, France; ^3^Rheumatology, Cochin Hospital, Paris, France; ^4^Ophtalmology, La Pitié Salpêtrière, Paris, France

###### **Presenting author:** Caroline Galeotti

**Introduction:** Blau Syndrome is a rare autosomal dominant inflammatory disease characterized by early-onset granulomatous arthritis, dermatitis and recurrent uveitis (1). Mutations in the nucleotide-binding domain (NBD) of *CARD15/NOD2* gene (mainly R334W, R334Q and L469F) have been identified in Blau syndrome (2). Polymorphisms in the leucine-rich repeats (LRR) of *CARD15* are associated with Crohn’s Disease, and mutations in the NBD domain are observed in Blau syndrome (3).

**Objectives:** We describe two patients with granulomatous uveitis carrying rare genetic variants of NOD2.

**Methods:** The analysis of the coding exons of the *NOD2/CARD15* gene was performed in our two patients because they had a granulomatous uveitis.

**Results:** The first patient, a 14 years old boy, had an isolated bilateral granulomatous uveitis since he was 10 years old. He was treated by steroids and methotrexate. He carried the A725G variant of *NOD2 (rs5743278* MAF 0.018). The second patient, a 15 years old girl had an isolated bilateral granulomatous panuveitis since she was 5 years old. She was treated by azathioprine and steroids. She carried two other rare genetic variants of *NOD2*: H287Y and L682F (MAF = 0.0002).

**Conclusion:** NOD2 is composed of three domains: a C-terminal LRR domain involved in ligand recognition, a central NBD and an N-terminal CARD domain. Our two patients carried a rare genetic variant located between the LRR and the NBD domains (A725G and L682F). The A725G variant has been previously described in patients with Crohn disease’s (CD) phenotype by Hugot et al (3) but our first patient had no clinical symptom related to CD. The second patient carried another rare variant located in the NBD domain (H287Y), compatible with the diagnosis of Blau syndrome. According to these observations, genetic variations located between the LRR and the NBD domains of NOD2 might be associated with the occurrence of granulomatous uveitis. It should be thus recommended to screen NOD2 mutations in patients with isolated granulomatous uveitis.

**References:**

1. Blau EB. Familial granulomatous arthritis, iritis, and rash. The Journal of pediatrics. 1985 Nov;107(5):689-93. PubMed PMID: 4056967. Epub 1985/11/01. eng.

2. Miceli-Richard C, Lesage S, Rybojad M, Prieur AM, Manouvrier-Hanu S, Hafner R, et al. CARD15 mutations in Blau syndrome. Nature genetics. 2001 Sep;29(1):19-20. PubMed PMID: 11528384. Epub 2001/08/31. eng.

3. Hugot JP, Chamaillard M, Zouali H, Lesage S, Cezard JP, Belaiche J, et al. Association of NOD2 leucine-rich repeat variants with susceptibility to Crohn’s disease. Nature. 2001 May 31;411(6837):599-603. PubMed PMID: 11385576. Epub 2001/06/01. eng.

**Disclosure of Interest:** None Declared

### P459 Characteristics of children with juvenile idiopathic arthritis and JIA-associated uveitis

#### Hala Etayari, Hashad Soad

##### Pediatric rheumatology, Tripoli Children Hospital, Tripoli, Libya

###### **Presenting author:** Hala Etayari

**Introduction:** JIA is a group of heterogeneous diseases characterized by the presence of chronic arthritis develops before the 16^th^ birthday after the exclusion of other causes of chronic arthritis. Uveitis is the most common extra-articular manifestation in JIA which has vision threatening complications. The well known risk factors for development of uveitis in JIA are 1) younger age at the development of JIA, 2) female sex, 3) short disease duration, 4) ANA positivity 5) RF negative disease 6) oligoarticular subtype. Since the disease is most of the times asymptomatic, regular screening by ophthalmologist is necessary for early diagnosis and treatment.(1-4)

**Objectives:** The Rheumatology Clinic of Tripoli Children Hospital receives JIA cases from western and southern areas of Libya. Despite adherence to the guidelines for uveitis screening, low incidence of uveitis was noticed. We studied this population to find out the reason for this low prevalence.

**Methods:** This case series study is performed by reviewing the medical records of the patients. Data regarding demographic characters, age at presentation and if MTX was received are collected, for those with uveitis if received before development of uveitis. Data was summarized by using percentages, mean and standard deviation where applicable. Chi square test was used to compare JIA with uveitis and JIA with no uveitis cases. Mann-Whitney test was used in comparing age at presentation. 155 JIA cases were included and followed up for a period ranged from 3 weeks to 12.5 years. Patients were included even if they were followed up for short period because tow has developed uveitis during this period.

**Results:** The majority of cases are of the oligoarticular type and polyarticular seronegative type representing 28.3% and 27.7% of the cases respectively. Age at presentation ranged from 10 months to 15.5 years with mean 8 ± 4years. Females represented 67.1% of the cases and female predominance was noticed in all JIA types except ERA were males and females are nearly equal (male to female ratio 1:1.2)

ANA positive cases represented 20% and rheumatoid factor positive cases represented 8.4% of total cases.

Of the 155 cases 9 (5.8%) developed uveitis. There are also 6 cases of idiopathic uveitis still following up may develop JIA not included in the analysis. 2 of the uveitis cases were males and 7 were females. By comparing uveitis and non-uveitis cases sex was not found a significant factor (p value = 0.22).The period from JIA diagnosis to development of uveitis ranged from 0 (uveitis developed at diagnosis) to 6 years with average of 2 years. The mean age at JIA diagnosis in uveitis cases is 5.6 ± 2 years. This is significantly lower than non-uveitis cases (p value (0.042)

4 cases developed in the persistent oligoarticular subtype, 2 cases in the polyarticular seronegative subtype, 1 case in the extended oligoarticular subtype, and 1 case in Enthesitis related arthritis.

All of the cases were rheumatoid factor negative and 5 of them were ANA positive. Comparing uveitis and non-uveitis cases ANA positivity was a significant factor in the development of uveitis (p value = 0.00) while rheumatoid factor result was not a significant factor (p value = 0.523)

6 of uveitis patients did not receive MTX as a treatment before the development of uveitis while 4 of them received MTX.

By comparing uveitis and non-uveitis cases MTX did not significantly affect the development of uveitis (p value = 0.47)

**Conclusion:** The incidence of uveitis is one of the lowest amung JIA cases and the factors found to be significant in the development of uveitis are age at presntation and ANA positivity.

**References:**

1. Angeles-Han ST, Pelajo CF, Vogler LB, Rouster-Stevens K, Kennedy C, Ponder L, et al. Risk Markers of Juvenile Idiopathic Arthritis-associated Uveitis in the Childhood Arthritis and Rheumatology Research Alliance (CARRA) Registry. J Rheumatol. 1 December, 2013;40(12):2088–96.

2. Angeles-Han ST, McCracken C, Yeh S, Jenkins K, Stryker D, Rouster-Stevens K, et al. Characteristics of a cohort of children with Juvenile Idiopathic Arthritis and JIA-associated Uveitis. Pediatr Rheumato. December,;13(1). down lowded from: http://www.ped-rheum.com/content/13/1/19

3. Petty RE, Laxer RM, Lindsley CB, Wedderburn L, Cassidy JT. Textbook of pediatric rheumatology Chapter/3-s2.0-C20120003493

4. Patel H, Goldstein D. Pediatric uveitis. Pediatr Clin North Am. 2003;50(1):125–136.

**Disclosure of Interest**: None Declared

### P460 The experience in children presenting with uveitis in the city of Abu Dhabi

#### Ihab El Kadry^1^, Habibullah Eatamadi^2^, Kais AlAlgawi^3^, Mustafa Al Maini^4^, Khulood Khawaja^4^

##### ^1^Academic Affair, Al Mafraq Hospital, Abu Dhabi, United Arab Emirates; ^2^Ophthalmology Department, Sheikh Khalifa Medical City, Abu Dhabi, United Arab Emirates; ^3^Ophthalmology Department, Al Mafraq Hospital, Abu Dhabi, United Arab Emirates; ^4^Rheumatology Department, Al Mafraq Hospital, Abu Dhabi, United Arab Emirates

###### **Presenting author:** Ihab El Kadry

**Introduction:** Uveitis in children can lead to blindness or other serious complications if there is delay or inadequate treatment.

**Objectives:** We aimed to describe the cohort of children presenting with uveitis to the main 2 public hospitals (Al Mafraq and Sheikh Khalifa Medical City) in Abu Dhabi which is the capital city of the United Arab Emirates.

**Methods:** Cases with the diagnosis of uveitis identified through electronic Medical Record from 1/1/2011 to 31/12/2015 using the International Statistical Classification of Diseases and Related Health Problems, i.e. International Classification of Disease version 9 (ICD 9 code) of 363.20, 364, 364.3, 714.2. Retrospective review and descriptive analysis performed by SPSS IBM, USA.

**Results:** 35 patients identified with the diagnosis of Uveitis. Mean current age 11.8 yrs. (range 4 to 20 yrs.). Mean age at diagnosis of 8.9 years (range 2 to 16). Equal male to female ratio. Mean duration from onset to diagnosis of 71 days (range 1 to 730 days). Rheumatic condition was the primary diagnosis in 12 (9 had Juvenile Idiopathic Arthritis, 2 sarcoidosis, 1 Behcet’s disease). Non rheumatic conditions in 20 (9 traumatic, 8 idiopathic, 2 tuberculosis, 1 Seckel Syndrome), 3 patients data was not available. 23 patients out of 34 patients were seen by Pediatric Rheumatologist (PR). The average duration from diagnosis to first appointment by PR is 193 days (range 1 day to 3yrs). 13 Patients had one eye affected (37%), 22 patients had both eye affected (63%). 23 patients diagnosed as anterior uveitis (66%), 7 panuveitis (20%), 1 posterior uveitis (3%) and 4 patients unclear data. 46% of patients presented with red eye, followed by 31% of patients with eye pain and vision changes, 9% with photophobia, 6% with eye tearing and 3% presented with eye discharge, foreign body sensation, eye Itching, abnormal eye movement, white pupil and upper lid ptosis. ANA + ve in 3 patients, -ve in 11 patients, 10 patients not done and 11 patients had no documentation. 7 patients had HLA status done; all negative. For treatment: 34/35 Topical steroid (97%), 17/35 systemic steroid (49%). 16/35 methotrexate (46%), 5/35 Adalimumab (14%), 3/35 Mycophenolate mofetil (9%), 2/35 Infliximab (6%). 11 patients needed surgery (31%) and 4 antiglaucoma medications (11%). 13 patients developed cataract (37%), 8 glaucoma (23%). 14 patients had partial loss of vision (40%) and 2 patients (6%) had complete blindness. One patient had methotrexate related liver fibrosis (No Rheumatology Input). Our data showed 22/35 (63%) of patients lost to follow up and 17/35 (49%) had poor compliance. 13/35 (37%) in remission off treatment, 7 (20%) ongoing treatment for active Uveitis. 4 (11%) patients had relapse, 12 (34%) had no relapse and 12 no clear data.

**Conclusion:** Lengthy duration from onset of Uveitis to first appointment with PR (average of 193 days with range of 1 day to 3 years). Some patients on methotrexate or biologics did not get to see PR. Outcome can be improved by managing patients with Uveitis as joint care between Ophthalmology and PR

**Disclosure of Interest**: None Declared

### P461 Epidemiology of uveitis in children at a tertiary referral center

#### Sophie Van den Berghe^1^, Ilse de Schryver^2^, Ann Raes^1^, Rik Joos^1^, Joke Dehoorne^1^

##### ^1^Pediatric rheumatology, Pediatric Universitary Hospital, Gent, Belgium; ^2^Pediatric ohthalmology, Universitary Hospital, Gent, Belgium

###### **Presenting author:** Joke Dehoorne

**Introduction:** Childhood uveitis is rare but devastating and can lead to severe vision loss. The inflammation has different potential causes and can be associated with systemic diseases.

**Objectives:** To analyse demographics, anatomic data, diagnosis, systemic associations, complications and treatment in children with uveitis in a tetiary eye center

**Methods:** Medical records of all patients with uveitis, up to the age of 16, referred to the department of Ophthalmology of Ghent University Hospital from 2003 to 2013 were investigated retrospectively. Patients were classified by the location of ocular inflammation according to the Standardized Uveitis Nomenclature (SUN).

**Results:** Among 66 children with uveitis, 62.1% were girls. Mean age at diagnosis was 8.6 years and mean follow-up time was 4.9 years. Uveitis developed in 18 children between 0 and 5 years, in 24 children between 6 and 10 years, and in 24 children between 11 and 16 years.The course was acute in 12.1% of the patients, recurrent in 21.2% and chronic in 66.7%. Bilateral involvement was seen in 69.7%. 56.1% developed anterior uveitis, 27.3% intermediate uveitis, 6.1% posterior uveitis and 10.6% panuveïtis. No systemic associations were found in 36.4% of patients, 31.8% had juvenile idiopathic arthritis associated uveitis, 9.1% had other systemic associations, 16.7% had an infectious cause and 6.1% had a specific form of uveitis. 75.8% of the patients received systemic treatment. The immunosuppressive agents included methotrexate (56.1%), cyclosporine (16.7%) and biologic (TNF-alpha blocking) agents (30,3%). The most common complications were posterior synechiae (39.4%), cataract (21.2%) and band keratopathy (13.6%). 11 of the 14 patients with cataract underwent phacoemulsification including primary intraocular lens implantation.

**Conclusion:** The epidemiology of the population of children with uveitis seen at the Ghent University Hospital, is comparable to previous studies except that patients are treated more often with biologicals and other immunosuppressive agents. Furthermore, less complications are observed. Prospective studies, with attention for visual prognosis, are needed to confirm our results and to investigate the effect of early immunosuppressive therapy. Uveitis in children is a devastating disease. A close collaboration between pediatric ophthalmologists and rheumatologists is recommended for early diagnosis and therapy.

**Disclosure of Interest**: None Declared

### P462 Uveitis in a paediatric rheumatology outpatient clinic

#### Lídia L. C. Teixeira, Ana Duarte, Sandra Sousa, Filipe Vinagre, Maria J. Santos

##### Rheumatology, Hospital Garcia de Orta, Almada, Portugal

###### **Presenting author:** Lídia L. C. Teixeira

**Introduction:** Uveitis describes an heterogeneous group of inflammatory diseases of the eye representing both systemic immune-mediated and infectious processes as well as inflammatory processes localized to the eye.^1^ When associated with Juvenile idiopathic arthritis (JIA), uveitis occurs most frequently in oligoarticular JIA. A significant number of patients already have ocular complications at the time of diagnosis. The use of immunosuppressive agents significantly reduces ocular complications.^2^

**Objectives:** Characterization of patients attending the Paediatric Rheumatology outpatient clinic of Hospital Garcia de Orta with diagnosis of uveitis associated with JIA and not associated with JIA, medication use and visual outcomes.

**Methods:** Retrospective analysis of patients with the diagnosis of uveitis between 2000 and 2016.

**Results:** Fifteen patients were identified, 53.3% were female and 46.7% male, 9 (60%) with Uveitis associated with JIA (UaJIA) and the others 6 with uveitis non-associated with JIA (UnaJIA).Patients with UnaJIA were referred to the paediatric rheumatologist mainly due to the need of systemic immunosuppressant therapy, but also in order to exclude underlying rheumatic disease. The first manifestations of uveitis occurred in average at the age of 6.4 years and the diagnosis established within a month. The most frequent symptoms were photophobia (66.7%), red eye (53.3%) and decreased visual acuity (46.7%). Eighty percent of the cases had sudden onset and uveitis localized to the anterior chamber, and in the UnaJIA group 33.3% had panuveitis. The number of episodes was 2 or less in UaJIA but higher in UnaJIA (in 50% more than 2 episodes). In UaJIA group the most frequent underlying diagnosis was oligoarticular JIA and in UnaJIA group all were idiopathic. Most patients were treated with topical and systemic steroids and had a medium exposition time to immunosuppressive agents of 4.85 years. Two patients with UnaJIA non-responsive to combination of oral glucocorticoids and methotrexate received adalimumab. In the UaJIA group there are also 2 patients using biologics (one adalimumab, and one certolizumab). Posterior and anterior synechiae were the main complications. Patients with decreased visual acuity at uveitis onset fully recovered.

**Conclusion:** Despite the small number of patients our results pointed out some different features between UaJIA and UnaJIA. UnaJIA seems to be more symptomatic with decreased visual acuity and photophobia at the beginning, more recurrent and chronic course.

**Disclosure of Interest**: None Declared

**Table 11 (abstract P462).** Demographic and disease characterization of children with uveitis. ERA – Enthesitis related arthritis

	Associated with JIA (n = 9)	Non associated with JIA (n = 6)	Total (n = 15)
Sudden onset n (%)	6 (66.7)	6 (100)	12 (80)
Insidious onset n (%)	3 (33.3)	0	3 (20)
Anterior n (%)	9 (100)	3 (50)	12 (80)
Intermediate n (%)	0	1 (16.7)	1 (6.6)
Posterior n (%)	0	0	0
Bilateral n (%)	3 (33.3)	4 (66.7)	7 (46.7)
Acute course	6 (40)	1 (16.7)	7 (46.7)
Recurrent course	2 (22.2)	3 (50)	5 (33.3)
Chronic course	1 (6.7)	2 (33.3)	3 (20)

### P463 Uveitis in children at the structure of juvenile idiopathic arthritis (JIA)

#### Nataly S. Shevchenko, Ludmila F. Bogmat, Marina V. Demyanenko

##### Department of cardiorheumatology, Institute of Children and Adolescents Health Care, Kharkiv, Ukraine

###### **Presenting author:** Nataly S. Shevchenko

**Introduction:** The damage of the eyes with development of uveitis is one of the most severe symptoms of juvenile arthritis. The frequency of pathological changes of the organ of vision in children with JIA ranges from 6 to 18%, and according to some authors up to 78% (40-70%). The childhood blindness and visual impairment occurs in 25% of children with endogenous uveitis (Guseva M.R., 2001). About 35% of patients with uveitis become blind (9.2%) or visually impaired. More than a third has indications for surgical intervention. The disability was depended (27%) with infringement of vision (Salugina S.O., Katargina L.A., Starikova A.V., 2004)

**Objectives:** Features of clinical manifestations of JIA with uveitis were analyzed to clarify the laws of development of eye damage in children at the present stage.

**Methods:** This was a prospective cohort study enrolling patients with JIA and uveitis. Descriptive statistics data are presented as mean ± standard error.

**Results:** 132 patients with IA (65.9% females) were enrolled.

Uveitis was diagnosed in 25 patients (18.9 ± 3,4%). 41.7% were males and 58.3% were females. Mean age was 9.8 ± 0.9 years (from one to seventeen years), mean disease duration was 79.5 ± 12.0 months (from 3 to 188 months). All patients were treated with SAIDs. 95.7% patients received MT, 15.3% MTSS. 28.0% of patients were treated with GC.

The patients with uveitis had the following options JIA: monoarthritis (20,0 ± 8,2%), oligoarthritis (36,9 ± 9,8%), polyarthritis (40,0 ± 10,1%).The term of uveitis appearance on the background of arthritis was 16.8 ± 5.7 months from the onset (from 1 to 108 months). The beginning of uveitis and arthritis at the same time was 48.0 ± 10.4%. Uveitis as first symptoms of JIA was 8.0 ± 5.4%. Only 23,1 ± 8.2% patients had ANA positive test. There were no a single RF positive patients.

Irreversible changes in the eye as the effects of uveitis developed following initial treatment of patients. There were cataract - 57.9%; degeneration of the cornea - 36.8%; synecious -42.1%; precipitates in the clear eye layers - 36.8%; fibrosis of the vitreous-10.1%.

**Conclusion:** Our results show that damage of the eyes with development of uveitis in children with JIA might exist already on the initial stages of disease course and at any stage, persist in future despite treatment. The above dictates the need to improve the diagnostic and therapeutic procedures in these patients. Timely diagnosis of uveitis at all stages of the disease should dictate the using of immune complex inflammation inhibitors (biologic treatment agents) with continuous monitoring of this category of patients.

**Disclosure of Interest**: None Declared

### P464 Chronic anterior uveitis in JIA patients taking Etanercept – a retrospective survey

#### Navdha R. Ramchurn^1^, Mark Friswell^2^

##### ^1^Paediatric Medicine, Great North Children’s Hospital, Newcastle upon Tyne, United Kingdom; ^2^Paediatric Rheumatology, Great North Children’s Hospital, Newcastle upon Tyne, United Kingdom

###### **Presenting author:** Navdha R. Ramchurn

**Introduction:** Chronic Anterior Uveitis (CAU) is the commonest extra-articular complication of Juvenile Idiopathic Arthritis (JIA)^1^. Treatment for CAU may involve topical steroid drops but some patients are also treated with systemic immunosuppression for their CAU and/or their JIA. The TNF-α inhibitor Etanercept is licenced in the UK for use as monotherapy, or in combination with Methotrexate (MTX), in patients with JIA where MTX has been ineffective or not tolerated^2^. However there is evidence to suggest it is not as effective as other TNF-inhibitors in treating CAU. It is also unclear what the risk of developing CAU is in children taking Etanercept as monotherapy compared to combination with MTX.

**Objectives:** To determine the rate of development of CAU in children taking Etanercept as monotherapy and in combination with MTX

**Methods:** We conducted a retrospective survey of our database of JIA patients at the Great North Children’s Hospital, a tertiary paediatric rheumatology centre in Newcastle, UK. All current patients were reviewed and those currently or previously taking Etanercept were identified. Data was collected on demographics, diagnosis, date Etanercept started/stopped etc. In our centre no child with previous uveitis is treated with Etanercept and any that develop it during treatment have it immediately switched to either Infliximab or Adalimumab. We therefore examined the reasons for stopping Etanercept, and in particular if CAU developed.

**Results:** 137 patients received Etanercept during the study period, with 155 distinct episodes of Etanercept therapy. 75% were female & median age was 9.7 years at the start of therapy. 56% had poly-articular JIA, 12% extended oligo-articular, 13% oligo-articular, 9% psoriatic, 7% ERA, & 4% systemic onset. There was no significant difference in demographics between those on monotherapy and those also on MTX.

72 patients were or had been taking Etanercept as monotherapy, with 77 distinct episodes of monotherapy. 5 episodes were excluded as therapy had been started within 3 months with no CAU screening yet done, leaving 72 episodes in 68 patients. Of these 72 episodes, median duration of Etanercept monotherapy was 18 months (range 3- 85).

65 patients were, or had been taking Etanercept in combination with Methotrexate with 78 distinct episodes of Etanercept treatment of median duration of 13 months (range 0-99).

34 of the 72 patients on monotherapy had Etanercept discontinued during the study period. 9 had been in remission for >2yrs, 7 were switched to an alternative Biologic for inefficacy, 5 had MTX added in, 4 stopped for other reasons including parental choice.

Nine (13%) patients taking Etanercept as monotherapy developed new-onset CAU and needed their treatment switched to an alternative Biologic. None had a history of pre-existing CAU and CAU developed a median of 7 months after Etanercept was started (range 3-65).

Seven of the 65 (11%) patients treated with MTX and Etanercept also developed CAU during the study period after a median of 3 months (range 1-18).

**Conclusion:** · Over 10% of all patients taking Etanercept in our study developed CAU and needed to switch to an alternative Biologic.

· Taking Methotrexate in combination with Etanercept did not appear to offer any protective effect on the risk of developing CAU, although the numbers are too small to be statistically significant.

**References**

1. British Society for Paediatric and Adolescent Rheumatology and Royal College of Ophthalmologists. Guidelines for Screening for Uveitis in Juvenile Idiopathic Arthritis. 2006

2. National Institute for Health and Care Excellence. Abatacept, adalimumab, etanercept and tocilizumab for treating juvenile idiopathic arthritis (TA373). 2015

**Disclosure of Interest**: None Declared

### P465 JIA-uveitis in the shared-care context: is poor documentation putting patients at risk?

#### Rebecca A. James^1^, Lucy R. Wedderburn^1,2,3^, Clive Edelsten^1^, Reshma Pattani^1^, Clarissa A. Pilkington^1,2^, Sandrine Compeyrot-Lacassagne^1^

##### ^1^Department of Rheumatology, Great Ormond Street Hospital for Children NHS Foundation Trust, London, United Kingdom; ^2^UCL Institute of Child Health, London, United Kingdom; ^3^ARUK Centre for Adolescent Rheumatology, University College London, London, United Kingdom

###### **Presenting author:** Rebecca A. James

**Introduction:** Children with Juvenile Idiopathic Arthritis (JIA) are at risk of developing uveitis and associated complications. When not diagnosed and treated in a timely manner, uveitis can result in blindness. Uveitis screening guidelines advise on time to first eye check, and the frequency and duration of ophthalmology follow up. The purpose of screening is to reduce the level of structural damage to the eye at the onset of treatment and therefore reduce the lifelong risk of blindness. The effectiveness of, and compliance with screening programs is unknown in the UK. Currently, many patients whose arthritis is managed at tertiary paediatric rheumatology centres have their eye screening partially, or entirely, conducted elsewhere.

**Objectives:** To review the time to first uveitis screening in new patients to the Great Ormond Street Hospital (GOSH) Rheumatology service who have been diagnosed with JIA, and the rate of uveitis and its complications at the time of diagnosis. To compare our institutional practices with national standards for eye screening in JIA. To review available documentation of the need for uveitis screening, and outcomes thereof, in standard clinic correspondence to local services.

**Methods:** We conducted a retrospective review of all patients newly diagnosed with JIA through our outpatient service in a 6 month period. This study was registered with the institutional Clinical Audit department. New patients were identified using the computer booking system, and the diagnosis of JIA was determined based on the first clinic letter. A chart review was then undertaken to examine time to first uveitis screen, presence or absence of uveitis and its complications, and the effectiveness of written communication between our centre and local services.

**Results:** 50 patients were newly diagnosed with JIA through our service in the six months period. Of these, 18 (36%) had completed their first uveitis screen, with known outcome, before their first rheumatology appointment at GOSH, and 28 (56%) of patients were referred for ophthalmology review by their GOSH rheumatologist. In 8% of patients, there was no record of any eye referral ever having been made. Of the 28 patients referred by our team for eye examinations, 19 were seen at our centre and 9 were referred to local services. Of the patients whose eye checks were conducted at our centre, 95.2% had their first eye screen within the national target. Median delay from rheumatology appointment to uveitis screening was 0 days (range -21 – 56 days, as some were seen by the ophthalmologist first). Of the 7 patients referred back to local ophthalmologist, outcome of uveitis screening remained unknown in 71.4%. Five patients (10%) already had uveitis by the time of their first eyecheck; over half of these children had complications present by the time their uveitis was diagnosed. Documentation of uveitis risk and the need for eye screening was inconsistent: 20% of patients had no mention of uveitis screening in their first clinic letter. Overall, 22% of newly diagnosed JIA patients at our centre did not meet national uveitis screening guidelines, primarily due to inadequate documentation.

**Conclusion:** Documentation of the need for uveitis screening in children newly diagnosed with JIA was inconsistent. The majority of children referred to local services for ophthalmology screening had no documented outcomes, including whether they had ever completed an eye check. Improved communication between tertiary and local services is important to reduce avoidable blindness arising from screening delays in children with newly diagnosed JIA.

**Disclosure of Interest**: None Declared

### P466 JIA-uveitis flare in the 6 months following methotrexate withdrawal: are patients being missed by current screening guidelines?

#### Rebecca A. James^1^, Sandrine Compeyrot-Lacassagne^1^, Clive Edelsten^1^, Reshma Pattani^1^, Clarissa A. Pilkington^1,2^, Lucy R. Wedderburn^1,2,3^

##### ^1^Department of Rheumatology, Great Ormond Street Hospital for Children NHS Foundation Trust, London, United Kingdom; ^2^UCL Institute of Child Health, London, United Kingdom; ^3^ARUK Centre for Adolescent Rheumatology, University College London, London, United Kingdom

###### **Presenting author:** Rebecca A. James

**Introduction:** Uveitis is a major source of morbidity in patients with Juvenile Idiopathic Arthritis (JIA). If not identified and treated early, it can lead to irreversible loss of vision. As uveitis is often asymptomatic, guidelines exist for routine screening in patients with JIA. Screening frequency and duration are based on age, gender, disease duration, JIA subtype and ANA status.

**Objectives:** To describe a series of 8 patients with JIA who experienced a flare of uveitis within 6 months of methotrexate withdrawal, including new onset uveitis and flares of pre-existing disease. To analyse protein MRP8/14 levels in serum taken before methotrexate cessation in a subset of these patients.

**Methods:** All cases are children with Juvenile Idiopathic Arthritis managed by a paediatric rheumatologist at our centre. This study was registered with our institutional Clinical Audit department. Cases were identified by their treating paediatric rheumatologist, ophthalmologist or specialist optometrist, and by case recognition in the clinic setting. A retrospective chart review was undertaken to identify onset and duration of methotrexate treatment, onset of uveitis and time to uveitis relapse. JIA-uveitis screening occurred at both our centre and at the patients’ local hospitals. Serum levels of MRP8/14 protein were measured by the hospital Immunology service using commercially available ELISA (Buhlman).

**Results:** Eight patients were identified with either new onset uveitis or a uveitis relapse within 6 months of methotrexate cessation; one patient had experienced this on two separate occasions. 5 patients were female. Median age at uveitis onset or relapse was 7.76 years (5.16-10.52 years) and median duration of arthritis was 5.08 years (1.60-9.25). All patients were ANA positive; no patients were on DMARDs other than methotrexate. 3 patients developed uveitis for the first time after coming off methotrexate, all within 3 months of methotrexate withdrawal (range 24-70 days). One of these patients developed uveitis after the current national screening guideline period. 75% of patients also experienced a concomitant flare of their arthritis. 2 patients experienced complications of their uveitis, including synechiae, subconjunctival haemorrhage, disc oedema and corneal folds; one of these patients required a periocular steroid injection to control the uveitis. MRP8/14 concentration in serum taken before methotrexate cessation was available for 4 patients; 3 of these patients were considered to be at ‘medium’ or ‘high risk’ of arthritis flare based on their MRP result.

**Conclusion:** The six months following methotrexate withdrawal may represent a risk period for relapse or new onset JIA-uveitis. In some, the level of uveitis activity may be sufficiently severe to cause irreversible structural damage. This suggests that the frequency of eye screening should be escalated in the months following methotrexate withdrawal, including in those who have completed current uveitis screening requirements. Methotrexate used for the treatment of JIA arthritis may delay the development or relapse of uveitis in some patients, leading to a period of increased eye inflammation upon methotrexate withdrawal. Uveitis screening guidelines may require revision to take into account systemic immunosuppression used in the treatment of arthritis.

**Disclosure of Interest**: None Declared

## Publication only

### B1 Blau syndrome, to cases positives for NOD 2 mutation in Mexico

#### Ana V. Villarreal^1^, Nydia Acevedo^2^, Enrique Faugier^1^, Rocio Maldonado^1^

##### ^1^Pediatrics Rheumatology, Hospital Infantil de Mexico Federico Gómez, Ciudad de Mexico, Mexico; ^2^Paediatric Rheumatology, Hospital Infantil de Mexico, Federico Gomez, Ciudad de Mexico, Mexico

###### **Presenting author:** Ana V. Villarreal

**Introduction:** Blau syndrome (MIM no. 186580) or Pediatric Granulomatous Arthritis it is a granulomatous inflammatory disease with an autosomal dominant inheritance pattern. The mutation in the NOD 2 gene, corresponding to chromosome 16q12.1-13, is responsible for this disease, as well as sporadic early-onset cases not associated with a inheritance pattern. Classically present in early childhood as a triad of granulomatous dermatitis, arthritis and uveitis. The following clinical cases are genetically related (siblings) and their father, who were diagnosed with Blau; until today they are the first cases reported in our Hospital and the second and youngest cases in Mexico.

**Objectives:** We report the second and youngest cases in Mexico diagnosed with Blau Syndrome confirmed finding the NOD2 mutation.

**Methods:** The first case it’s an 8 years’ male his father was 43 years old with camptodactyly since childhood without another condition, healthy mother off 46 years. Sister 4 years diagnosed with atopic dermatitis from birth and now with arthritis. The mother reports that since his 2 months of age with dermatitis described as confluent papules initially in proximal portions of side extremities, treated as atopic dermatitis, start topical steroids and moisturizers, intermittent course with periods of exacerbation. 1 year Later develop wrist arthritis. Physical examination: rash described as micropapular dermatitis arms and forearms, right wrist with painful boggy synovitis, camptodactyly, and arthritis of the left ankle.

PCR FOR GENE SEQUENCING NOD 2 / CARD 15 heterozygosity for pH603R exon 4 (c 1808 A > G).

The Father showed a positive mutation of NOD GEN 2 / CARD 15 heterozygosity for pH603R exon 4 (c 1808 A > G)

The second case its the 4 years case 1 sister, who starts her condition from birth diagnosed with Atopic Dermatitis has received multiple antihistaminic and topical steroid developing exacerbations two to three times per year, accompanied by camptodactyly.

Positive physical examination with papules and nodules smaller than 4mm that tend to coalesce in lower extremities. Limitation to the extension of both shoulders and elbows and camptodactyly in both hands.Skyn Biopsy shows the presence of granulomas. PCR sequencing for NOD GEN 2 / CARD 15 heterozygosity for pH603R exon 4 (c 1808 A > G)

**Results:** It is two patients, siblings, with a history of skin condition chronic course, and father camptodactyly and without other manifestations; who have periods recurrence and exacerbations of skin disease and then develop polyarthritis, camptodactyly and synovitis features Blau syndrome, is suspected this diagnosis and confirmed by genetic mutation of NOD 2 gene, being the second family in Mexico; the first case reported in 2010 by Villanueva et al. In Ophthalmic Genetics.

Patients presented in this case initiated manifestations from early childhood and diagnosis at 8 and 4 years, respectively, currently in treatment with Methotrexate 15mg/m2sc/day and steroid without finding ocular or renal condition when presenting clear improvement of dermatitis and synovitis

**Conclusion: Blau** is a rare granulomatous auto inflammatory syndrome which most devastating manifestation is the eye, so pediatrics should consider a differential diagnosis of atopic dermatitis and arthritis in early childhood.

**References:**

· Wouters et al. **Blau Syndrome, the prototypic auto-inflammatory granulomatous disease** Pediatric Rheumatology 2014,12:33.

· Villanueva et al. Familial case of Bleau syndrome associated with a CARD15/NOD2 mutation. Ophthalmic Genetics 2010, 31(3),155-158.

**Disclosure of Interest**: None Declared

### B2 Bibliometric analysis of the 100 most cited manuscripts in Familial Mediterranean Fever

#### Dilek Yılmaz^1^, Hilal Bektaş Uysal^2^

##### ^1^Pediatric Nephrology, Adnan Menderes University Faculty of Medicine, Aydın, Turkey; ^2^Internal Medicine, Adnan Menderes University Faculty of Medicine, Aydın, Turkey

###### **Presenting author:** Dilek Yılmaz

**Introduction:** The studies which investigate the cumulative scientific data about a specific subject and assess the scientific publication performance are named as bibliometric analysis. Bibliometric studies help researchers to monitor the literatur data closely and guide them to design new studies. Familial Mediterranean Fever (FMF) is an autosomal recessive disease which is more prevalent among Turks, Jews, Armenians and Arabs. The most serious complication of FMF is the development of renal failure due to amyloidosis.

**Objectives:** In this study, the 100 most cited manuscripts in the field of FMF are analysed.

**Methods:** This study was performed retrospectively by using “Thomson Reuters Web of Science” database in April 2016. With the “Web of science ^TM^ Core Collection” selection and using the search term “familial mediterranean fever” the dataset was composed. The dataset was filtered to include the manuscripts only in the scope of “Science Citation Index Expanded (SCI-E)”. Furthermore, the first most cited 100 manuscripts were analysed in terms of topic, journal, author, year and instution.

**Results:** The database search returned 2027 manuscripts and the most cited first100 papers were included. The most cited paper (by Aksentijevich) was focused on “Ancient missense mutations in a new member of the RoRet gene” (776 citations). Among these most cited 100 manuscripts; Livneh had 7 manuscripts, Gershoni-Baruch had 4 manuscripts and Ben-Cherit, Tunca, Chae, Toutiou, Booth, Ozen had 3 manuscripts, as the first author. All the remaining authors had one publication. Livneh had the highest volume of citations with 1198. This was follwed by Ben-Chetrit with 534 citations, Tunca with 464 citations, Toutiou with 413 citations, Chae with 406 citations, Gershoni-Baruch with 317 citations, Booth with 255 citations and Ozen with 209 citations. These most cited manuscripts were published in 44 journals. When these journals were evaluated according to the number of citations; “Arthritis and Rheumatism” had the most citations (1337) and “European Journal of Human Genetics” (1039) and “Cell” (776) were the following journals. “New England Journal of Medicine” had the highest 5 year impact factor (45.941), “Cell” (28.779) and “Nature Genetics” (24.416) were the following high impact journals. “Tel Aviv University” was the first institute with 16 publications, “Hacettepe University” was the second institute with 10 publications and “Hop Arnaud De Villeneuve, Biochim Genet Lab” was the third institute with 8 publications. Also, “Tel Aviv University” was again the first in terms of institute citation numbers with 1937 citations. “Arthrit and Rheumatism Branch, Bethesda” (n = 1859) and “Hop Arnaud De Villeneuve, Biochim Genet Lab” (n = 1560) were the following institutes. When these publications were evaluated in terms of countries; Israel had the most cited manuscripts with 37%, Turkey and USA with 18%, France with 12%.Besides, the genetics of FMF was the most widely studied topic.

**Conclusion:** This work provides the most influential references related to FMF and serves as a guide to what makes a paper citable. ‘The genetics of FMF’ was the most widely studied topic and Israel, Turkey, USA and France were revealed as the arbiter countries of FMF topic.

**Disclosure of Interest**: None Declared

### B3 The first case of Blau syndrome (early-onset sarcoidosis) in a patient from Russia

#### Evgeny Fedorov^1^, Svetlana Salugina^1^, Elena Kamenets^2^, Ekaterina Zaharova^2^, Stefka Radenska-Lopovok^3^

##### ^1^Nasonova Research Institute of Rheumatology, Moscow, Russian Federation; ^2^FSBI “Research Centre for Medical Genetics”, Moscow, Russian Federation; ^3^Pathological Anatomy, I.M.Sechenov First T Moscow State. Medical University, Moscow, Russian Federation

###### **Presenting author:** Evgeny Fedorov

**Introduction:**

Blau syndrome(BS) is a hereditary autoinflammatory granulomatous disease associated with the NOD2/CARD15 gene mutations – a variant of sarcoidosis with juvenile onset, characterized by skin lesions in joints and eyes.

**Objectives:**

to introduce the first genetically and morphologically confirmed case of BS in a Russian patient.

**Methods:**

a standard clinical examination, biopsy of testis and wrist synovial membrane with histological examination (hematoxylin and eosin stain), molecular analysis of 4 exon of the *NOD2* gene by direct automatically sequencing.

**Results:**

Case report: onset from the first year of life - the occurrence of swelling of the testicles, aggravated by the age of 1 year and 4 months. From the age of 1 year and 7 months – polyarticular boggy arthritis with lesions in both wrist, proximal interphalangeal joint of the 3^d^ finger of the left hand, left knee and both ankle. Arthritis is characterized by the involvement of periarticular tissues, a significant amount of effusion, absence of pain at movement and normal range of motion. Monitoring by an ophthalmologist in dynamics is without pathology. From the age of 1 year and 8 months there was recurrent fever to 40.0°C lasting for 2 days with an interval of 6 months. Increase in the level of acute-phase markers (ESR, CRP) was never recorded. Autoantibodies (ANA, RF) are negative. In the biopsy samples of synovial membrane and a testicle there are typical sarcoidosis granulomas. When sequencing of the NOD2/CARD15 gene a missense mutation of NM_022162.2: c.1000C > T (p.R334W) in the heterozygous state was identified.

**Conclusion:**

BS should be included in the range of differential diagnostic conditions in children with bizarre course of polyarthritis and the presence of extra-articular symptoms. BS is confirmed by morphological examination of the affected tissues and detection of mutations in the NOD2/CARD15 gene.

**Disclosure of Interest**: None Declared

### B4 CINCA syndrome and interleukin - 1 blockade treatment

#### Joao Nascimento^1^, Helena Sofia^2^, Carla Zilhão^1,3^, Rui Almeida^4^, Margarida Guedes^1,3^

##### ^1^Pediatrics, Centro Hospitalar Porto, Porto, Portugal; ^2^Pediatrics, Hospital Vila Franca Xira, Vila Franca de Xira, Portugal; ^3^Pediatric Rheumatology Unit, Centro Hospitalar Porto, Porto, Portugal; ^4^Pediatrics, Hospital Pedro Hispano, Matosinhos, Portugal

###### **Presenting author:** Joao Nascimento

**Introduction:** CAPS (FCU, MWS and CINCA) are associated with mutations or misspellings in the nucleotide binding domain, leucine rich family (NLR), pyrin containing 3 (NLRP3) gene, also known as the CIAS1 gene. A mutation of the NLRP3 gene causes the cryopyrin inflammasome to constantly overproduce IL-1β.

CINCA represents the most severe clinical picture with patients becaming symptomatic in the neonatal period. Although rare, early diagnosis and proper treatment can help CAPS patients to live healthier lives.

**Objectives:** Describe switch from anakinra to canakinumab experience in a CINCA patient with eight years of follow up.

**Methods:** Case Report

**Results:** The patient, a caucasian portuguese male with eight years old, first child of a non-consanguineous couple, developed a persistent urticarial rash during the first week of life. Family history was unremarkable.

At 3 months old the rash was still present and swollen cervical and axilar lymph nodes were noticed (ESR 79mm/h). At 8 months old the patient still had the urticarial rash and developed irritability and fever that persisted for two months.

He was admitted to our hospital (hemoglobin 8,4 g/dl, CRP 81 mg/L, ESR 55 mm/h, 0 cells in cerebrospinal fluid) and a complete investigation was performed to exclude infectious, autoimmune and neoplastic etiologies. Serum amyloid A (SAA) was 156 mg/L at this time.

While on prednisolone 2mg/kg, the patient with 12 months old continue to have an intermitent rash (SAA – 548 mg /L, ESR 29 mm/h) and develop a left knee tumefaction. The MRI revealed a nonvascular but granular and fibrotic tissue near the medial femoral condyle.

At almost the same time, the PCR sequencing of exon 3 confirmed the existence of a heterozygous mutation C.1881A > T (p.Glu627Asp) on NLRP3 gene. This was a *de novo* mutation, since the parents did not show any NRLP mutation in the genetic testing.

The auditory evoked potentials test was normal but the patient had bilateral papilloedema and delayed fine motor skills. The patient started anti-IL1 therapy with anakinra at 15 months old. The clinical and analytical response was good, since after 6 months of therapy he was asymptomatic without edema of the optic disc and with a SAA value of 21,7 mg/L.

However, at 3 years old the home daily administrations of anakinra started to be a problem with some missing days of therapy related with anxiety and refusal patient behavior, despite the support of a primary care nurse.

The rheumatology unit demand to switch for canakinumab several times but it was only possible to start the therapy in February 2016. The starting dose was 2mg/kg. However because of an increase in SAA value to 75mg/L and tension headaches, the Pediatric Rheumatology Unit decided to rise the dose until 4mg/kg/d (8/8w). The clinical and analytical (SAA 4,5mg /L) response so far is good.

**Conclusion:** Prompt interleukin - 1 blockade treatment is the key to treat CAPS patients. The treatment scheme with canakinumab can be an advantage in pediatric patients in terms of commodity. The ideal dose to treat CAPS is still on debate.

**Disclosure of Interest**: None Declared

### B5 Idiopathic Recurrent Pericarditis in an adolescent: management with interleukin-1 receptor antagonist

#### Kubra Ozturk^1^, Murat Deveci^2^, Zelal Ekinci^1^

##### ^1^Department of Pediatrics Rheumatology, Kocaeli University Faculty of Medicine, Kocaeli, Turkey; ^2^Department of Pediatrics Cardiology, Kocaeli University Faculty of Medicine, Kocaeli, Turkey

###### **Presenting author:** Kubra Ozturk

This abstract is not included here as it has already been published.

### B6 The successful use of Adalimumab in 11-year old patient with Behcet disease, common disorder of gastrointestinal tract, demanding the exclusion of Crohn disease

#### Svetlana Rodionovskaya^1,2^, Vera Vinnikova^1^, Svetlana Salugina^2^, Evgeny Fedorov^2^, Irina Tsymbal^1^

##### ^1^Central Children’s Clinical Hospital of the Federal Medical-Biological Agency of Russia, Moscow, Russian Federation; ^2^V.A.Nasonova Research Institute of Rheumatology, Moscow, Russian Federation

###### **Presenting author:** Svetlana Rodionovskaya

**Introduction:** Behcet disease is an inflammatory disease characterized by recurrent ulcers of the mouth and genitalia, frequent damage of eyes, joints, nervous system and blood vessels; gastrointestinal symptoms were observed in 1-2% of patients.

**Objectives:** Description of the clinical observation of the patient with Behcet disease, severe disorder of the gastrointestinal tract (GIT).

**Methods:** Description of the clinical observation of the patient with Behcet disease, severe disorder of the gastrointestinal tract (GIT).

**Results:** 11-year old patient has been sick since 9 months: recurrent febrile fever, stomatitis, episodes of vomiting, abdominal pains, conjunctivitis. Deterioration of the condition has been observed since 10 years: fever attacks 39^0^ every 3 weeks, multiple painful mouth ulcers, abdominal pains, isolated perianal ulcers, meatostenoz, arthritic joints of the hands, weight loss of 5 kg; WBC 14 × 10^9^, PLT 437 × 10^12^, ESR 59 mm/h, CRP 19.8 mg/l, ferritin 397 mkg/l, procalcitonin > 10 N, HLA B5(52). Primary immunodeficiencies autoimmune polyglandular syndrome, herpes viral infections are excluded. Endoscopy (esophagogastroduodenoscopy (EGD), colonoscopy, VCE) revealed erosive and ulcerative stomatitis, esophagitis, erosive pyloritis, erosive and ulcerative bulbit, ulcerative proctitis, terminal ileitis and perianal fistula. Differential diagnosis with Crohn disease was presented, no morphological characters were found. The Behcet disease was diagnosed. Prednisolone 40 mg / day (1.5 mg/kg), azathioprine 2.2 mg/kg were prescribed. At the dose of prednisolone 20 mg / day, a relapse of the disease was observed: fever 39°C, stomatitis; according to endoscopy there were no erosive and ulcerative changes in the mucous of the upper gastrointestinal tract. Adalimumab (40 mg every 2 weeks) was prescribed subcutaneously. The therapy resulted in rapid relief of fever, stomatitis. The patient has been receiving Adalimumab therapy for 11 months, disease remission has been observed (no fever, stomatitis, disorders of the gastrointestinal tract, conjunctivitis, normalization of CRP, ESR), the dose of prednisolone was reduced to 5 mg / day, and administration of azathioprine was continued.

**Conclusion:** Behcet disease has a wide range of clinical implications, including severe disorder of gastrointestinal tract, requiring elimination of inflammatory intestinal diseases. Adalimumab therapy demonstrates effective control of inflammatory activity and a good safety profile with Behcet disease.

**Reference**

Skef W, Hamilton MJ, et al. Gastrointestinal Behçet’s disease: a review. World J Gastroenterol. 2015 Apr 7;21(13):3801-12.

**Disclosure of Interest**: None Declared

### B7 The clinical value of 18F-FDG PET-CT for early diagnosis of Takayasu arteritis in a patient having chest pain, anemia and elevation of acute phase reactants - case report

#### Edyta Olesińska^1^, Jacek Postępski^2^, Agnieszka Mroczkowska-Juchkiewicz^3^, Agnieszka Pawłowska-Kamieniak^3^, Beata Chrapko^4^

##### ^1^Department of Paediatric Pulmonology and Rheumatology, Children’s University Hospital, Lublin, Poland; ^2^Department of Paediatric Pulmonology and Rheumatology, Medical University of Lublin, Lublin, Poland; ^3^Department of Paediatrics, Medical University of Lublin, Lublin, Poland; ^4^Departament of Nuclear Medicine, Medical University of Lublin, Lublin, Poland

###### **Presenting author:** Edyta Olesińska

**Introduction:** Takayasu arteritis (TA) is large vessels vasculitis primarily affecting the aorta ana its mian branches. Symptoms in the prepulseless phase are nonspecific (fever, fatigue, weigt loss, myalgia), so that the diagnosis in children is delayed, even for years. Early TA diagnosis is essential to start treatment before irreversible structural changes occur (occlusive phase).

**Objectives:** To demostrate the role of 18-fluorodeoxyglucose (18F-FDG) positron-emission tomography-computed tomography (PET-CT) in early diagnosis of TA and monitoring disease activity.

**Methods:** A 14-years old boy presented with low-grade fever, chest pain and weaknes, of one month’s duration. On admission physical examination did not reveal any abnormalities. Laboratory tests revealed elevation of acute phase reactants (AFR) and anemia. All extensive examinantions (microbiological, serological, bone marrow, radiological /x-rays, CT scans, ECHO/, panedoscopy) did not explain the source of inflammation. 18F-FDG PET-CT images schowed high FDG uptake along the walls of the ascending aorta, aortic arch, its major branches, and abdominal aorta proximally to renal arteries. Takayasu arteritis was diagnosed. Patient started treatment with systemic steroids. We systematically assessed TA activity upon the presence of systemic features, elevation of erythrocyte sedimentation rate (ESR) and C-reactive protein (CRP), vascular ischemia. In 6th and 15th month of disease 18F-FDG PET-CT was performed that showed increased FDG uptkate along the carotid common arterie and persistent level of FDG uptake ina aorticarch and ascending aorta. We analyzed SUV max (maximum standardized value) and AFR in patient having mildly expressed clinical symptoms. [Table 12] The results were helpful in treatment decisions. Our patient was treated with methotreksat, followed by cyclophosphamid, and finally with adalimumab.

**Results:**

In our patient the early TA diagnosis, in the prepulseless phase, was performed with 18F-RDG PET-CT. Estimation of SUV max was useful tool to determine TA activity and to make treatment decisions.

**Conclusion:** 18F-RDG PET-CT is valuable diagnostic tool for the evaluatiom of children with FUO and unexplained signs of inflammation. 18F-RDG PET-CT is useful for early diagnosis of TA and for monitoring disease activity during and after treatment. Further prospective studies are needed in Poladn, especially for real and fast diagnosis and treatment.

**Disclosure of Interest**: None Declared

**Table 12 (abstract B7).** SUV max and acute phase reactants in 0, 6, and 15 months of disease

	0-Month	6-Month	15-Month
Carotid common right arteria (SUV max^a^)	4.2	4.6	3.1
Carotid common left arteria (SUV max)	3.4	4.9	4.3
Ascending aorta (SUV max)	4.1	3.7	2.7
Aortic arch (SUV max)	4.5	3.7	2.7
Abdominal aorta (SUV max)	3.6	3.6	3.1
CRP mg/dl (0-0,5)	15.2	8.1	4.8
OB. mm/h (<10)	119	34	28

^a^SUV maximum standardized uptake value

### B8 Clinical and MRI outcome in juvenile idiopathic arthritis patients with cervical spine involvement

#### Damjana Ključevšek^1^, Nina Emeršič^2^, Nataša Toplak^3^, Tadej Avčin^3^

##### ^1^Radiology Unit, University Children’s Hospital Ljubljana, Ljubljana, Slovenia; ^2^Department of Allergology, Rheumatology and Clinical Immunology, University Children’s Hospital Ljubljana, Ljubljana, Slovenia; ^3^Department of Allergology, Rheumatology and Clincal Immunology, University Children’s Hospital, Ljubljana, Slovenia

###### **Presenting author:** Damjana Ključevšek

**Introduction:** Cervical spine arthritis is a well-recognized complication of juvenile idiopathic arthritis (JIA). If untreated, it could be life-threatening with a poor prognosis. Therefore, early recognition and treatment is of main importance.

**Objectives:** The purpose of the study was to evaluate clinical and magnetic resonance imaging (MR) outcome of JIA patients with cervical spine involvement and importance of early treatment with biologics.

**Methods:** We conducted a retrospective study in JIA patients with cervical spine involvement. Medical charts and imaging were reviewed. Data, including age at disease onset, JIA type, disease activity, treatment and outcome were collected. Initial and follow-up MR examinations of cervical spine were performed according to the hospital protocol in 3 planes with paramagnetic contrast application to evaluate the presence of inflammation and potential chronical changes.

**Results:** 14 JIA patients with cervical spine involvement (10 girls, 4 boys, median age 6y, range between 2.5 and 15.1 y) were included in the study. Nine children were diagnosed with polyarticulare subtype, 4 with extended oligo-arthritis, and 1 with psoriatic arthritis. All children were initially treated by metotrexat (MTX) and pulse methylprednisolone therapy. In addition, 10/14 were treated with anti/TNF alpha drugs early in diseases course within 3 months of clinical signs of cervical spine involvement confirmed by MR. There was delay in introducing biological treatment in 3/14: first 2 patients in cohort received biologics after 1.2 year (hospital protocol at that time didn’t include early treatment with biological drug), and in 1 patient the reason for delay was the parent concerns regarding biologics. One boy didn’t received biological treatment due to parent refusal. The disease activity was evaluated at the time of initial and follow-up MR examinations. In addition, we were searching for possible chronical changes on MR: in 2 children thinned cortical layer of dens was found and in 1 morphological change of dens with atlanto- axial subluxation.

**Conclusion:** Based on our experiences, early treatment of cervical spine arthritis in children with biologics showed good response with rare and minimal chronical changes seen on MR.

**Disclosure of Interest**: None Declared

### B9 Experience of definition comethotrencentration of methotrexate in children’s with juvenile idiopathic arthritis serum

#### Faina Rokhlina

##### Pediatric, SPbGPMU, St.Petersburg, Russian Federation

**Introduction:** Juvenile idiopathic arthritis is a chronic rheumatic disease, the most common in rheumatic diseases in childhood. In many cases, children with JIA resistant to therapy with nonsteroidal anti-inflammatory drugs (NSAIDs), intra-articular injections and physiotherapy.

**Objectives:** According to various authors, about 70% of patients receiving MTX therapy are included in disease remission.

**Methods:** In our study, all patients receiving MTX, MTX was conducted determining the concentration in the blood serum. The results can be divided into three main groups: low concentration (≤1 mmol/l), the average concentration (1-2mmol/l) and high concentration (greater than 2 mmol/l). The percentage of a group of children with MTX concentration from 0 to 1 mmol/l - is 10 children (9.71%), 1 to 2 mmol l - 57 children (55.34%) and higher in group 2 mmol/l It included 36 patients (34.95%).

**Results:** We have conducted a study that included 103 children - 63.11% girls and 36.89% boys, with various forms of juvenile idiopathic arthritis: polyarthritis - 46 children, oligoarthritis - 25 children, systemic arthritis variant - 14 children, arthritis entezitassotsiirovanny - 18 patients. All the children included in the study received basic therapy with parenteral MTX at a dose of 15 mg/m2 for 3 months or more. Carol A. Wallace and colleagues have developed criteria for inactive disease.In the group of children with methotrexate concentration from 0 to 1 mmol/l of 1(10%) the child is in the inactive disease. In patients with methotrexate concentration of 1 to 2 mmol/l - 14 children (24.56%) with inactive disease, and in the group with high concentration (higher than 2 mmol/l), 12 patients (33.33%) correspond to the criteria inactive disease.

**Conclusion:** Among the surveyed our patients with JIA, we observe the relationship between increasing concentrations of methotrexate in blood and inactive disease.

**Disclosure of Interest**: None Declared

### B10 Case report: the effectiveness of adalimumab in a child with psoriasis developed during treatment of polyarticular juvenile idiopathic arthritis with abatacept

#### Galina Glazyrina^1^, Natalia Kolyadina ^2^

##### ^1^South Ural Governmental Medical University, Chelyabinsk, Russian Federation; ^2^Chelyabinsk Regional Clinical Paediatric Hospital, Chelyabinsk, Russian Federation

###### **Presenting author:** Galina Glazyrina

**Introduction:** Juvenile psoriatic arthritis is a rare variant of juvenile arthritis. According to the ILAR criteria, its rate is approximately 7%. Onset with a polyarticular arthritis is reported in 20% of patients; rarely arthritis is preceded by cutaneous psoriasis.

**Objectives:** Clinical case

**Methods:** A girl aged 10 years was admitted to the Chelyabinsk Regional Children’s Clinical Hospital (CRCCH) at the first time in September 2015 complaining of morning stiffness up to 2 hours, swelling, pain, and limitation of movements in the elbows, wrists, metacarpophalangeal joints, hip joints, knees, ankles; pain and limitation of movements in the cervical spine.

**Results:** Onset of the disease was reported 08/12/2014 with fever and swelling of the left wrist. Within one week, swelling of the elbows, knees, ankle joints, and cervical spine developed along with morning stiffness up to 2 hours. Subsequently arthritis of the proximal interphalangeal joints of the hands and feet developed. ESR was 40 mm/hr. CRP was 60 g/l. Juvenile seronegative polyarthritis was diagnosed. Methotrexate 15 mg/ m^2^ / week, folic acid, and NSAIDs were administered. Short-term improvement with reduced swelling and pain in the joints was reported. In August 2015 re-exacerbation of articular syndrome developed.

The patient was admitted to the CRCCH in September 2015. State of moderate severity. Pale skin without any lesions and/or nail disorders. The number of active joints 21, the number of joints with impaired function was 21. VAS score by physician was 80 mm. VAS score by parents was 85 mm. CHAQ Index was 2.0. ESR was 57 mm/hr. CRP was 100 g/l. RF was negative, HLA B27 antigen was not found. Uveitis was not revealed. No systemic arthritis symptoms were detected. Tuberculosis was excluded. Diagnosis was as follows: Juvenile arthritis, seronegative, 2 degree of activity, radiologic degree 2, FC 2. The patient was treated with: Metoject 15 mg/m^2^/ week SC, folic acid, and sodium diclofenac. Due to the methotrexate ineffectiveness during 9 months, 15 November 2015 abatacept 10 mg/kg was initiated via IV drips at week 0, 2, 4 (3 infusion). A slight positive changes were reported. In December 2015, a psoriatic rash over the elbow and knee joints developed. Dermatologist confirmed the diagnosis “psoriasis vulgaris.” No psoriasis have been identified in the first-degree relatives. The high laboratory activity persisted: ESR was 50 mm/hr. CRP was 88 g/l.

Given the change in diagnosis to the “Juvenile psoriatic arthritis,” the decision to switch from abatacept to the TNF alpha inhibitor (adalimumab) was made. Since January 3, treatment with adalimumab 40 mg SC every other week was initiated. After 1 month of treatment psoriatic rash significantly decreased, joint pain decreased as well, but the limitation of movements and laboratory activity persisted: ESR was up to50 mm/hr. CRP was over 100 g/l.

After 3 months the psoriatic rash resolved, range of motion in the joints increased, morning stiffness decreased, and laboratory activity decreased. The number of active joints was 12, the number of joints with dysfunction was 10. VAS score by physician was 40 mm. VAS score by parents was 45 mm. CHAQ Index was 1.0. ESR was 37 mm/hr. CRP was 22.2 g/l. The effectiveness according to the ACRpedi criteria at 3 months was 50%.

**Conclusion:** The special aspects of this case include the onset of seronegative arthritis without symptoms of nail disorders, dactylitis and/or psoriasis in other relatives; the development of psoriasis during treatment with abatacept; the effectiveness of adalimumab in the treatment of cutaneous manifestations of psoriasis and arthritis (according to the ACRpedi criteria at 3 months – 50%).

**Disclosure of Interest**: None Declared

### B11 Association of HLA-B27 with juvenile idiopathic arthritis

#### Kwangnam Kim^1^, Sinae Eom^1^, Daeyoung Kim^2^, Jungwoo Rhim^3^

##### ^1^Pediatrics, Hallym University Medical Center, AnYang City, Korea, Republic Of; ^2^Pediatrics, Inje University, Goyang City, Korea, Republic Of; ^3^Pediatrics, The Catholic University, Daejeon City, Korea, Republic Of

###### **Presenting author:** Kwangnam Kim

**Introduction:** It is well known that HLA-B27 is related to the manifestations of ankylosing spondylitis (AS) in adults. However, the influence of HLA-B27 in children is not clear. According to epidemiologic studies, it is suspected that HLA-B27 is associated with juvenile-onset spondyloarthropathy (SpA), enthesitis, and psoriasis in pediatric patients.

However, there are currently no satisfactory diagnostic criteria for juvenile-onset SpA. We can predict, to some degree, that patients with HLA-B27 have the possibility of being diagnosed with juvenile-onset SpA and developing adult-type AS later. Patients with HLA-B27 can have sacroiliitis and axial diseases 5–10 years after disease onset.

**Objectives:** To assess the clinical relationship between human leukocyte antigen B27 (HLA-B27) and manifestations of juvenile idiopathic arthritis (JIA) joint involvement site in in the single center of Hallym University Medical Center, Korea.

**Methods:** Medical records of 230 patients with JIA from a pediatric rheumatology clinic were reviewed during 6-year period between March 2006 and December 2014. We analyzed the children with regard to features of the joint involvement site and identified the HLA-B27 type by using the PCR sequence-specific primer methods.

**Results:** Of 230 children with JIA (boy : girl = 114 : 116), HLA-B27 was found to be positive in 69 (boy : girl = 47 : 22). The positive rate of HLA-B27 was 30%. The proportion of HLA-B27-positive patients was higher in boys than girls (p = 0.001). Among the positive HLA-B27, systemic JIA (7/69, 10%), oligo persistant (31/69, 45%), oligo extended (7/69, 10%) and poly JIA (19/69, 27.5%). In boys, the age at disease onset was older in the HLA-B27-positive group (9.3 ± 2.9 years) than it was in the HLA-B27-negative group (6.4 ± 3.5 years, p = 0.001). Back pain was associated with HLA-B27 in boys (p = 0.002) but not in girls (p = 0.616). In both sexes, involvement of the small joints in the lower extremities was highly associated with HLA-B27 (p = 0.001 for boys, p = 0.021 for girls). In addition, HLA-B27 was associated with enthesitis in boys(p = 0.004). The radiologic change of the sacroiliac joints among the 230 JIA, HLA-B27 positive group was 12/69 (17.4%), and HLA-B27 negative group was 2/161 (1.2%).

**Conclusion:** We found characteristic patterns of joint involvement sites in HLA-B27-positive JIA patients. Lower-extremity small joints and entesitis were distinctively involved in HLA-B27 positive JIA. Conversely, the proportion of upper extremity joint involvement was higher in HLA-B27 negative JIA boys. These characteristic patterns are important factors for predicting future disease progression in patients with HLA-B27-positive JIA. We also suggest that the ILAR classification have some weakness for diagnosing HLA-B27 positive JIA patents in the early stages.

**Disclosure of Interest**: None Declared

### B12 A case of severe PPRD (Progressive Pseudorheumatoid Dysplasia) associated with inflammatory polyartrhritis, responsive to TNF-blockade

#### Francesca Ricci^1^, Paola Montesano^1^, Barbara Bonafini^1^, Veronica Medeghini^1^, Ilaria Parissenti^1^, Antonella Meini^1^, Marco Cattalini^1^, Paolo Airò^2^

##### ^1^Clinical and Experimental Sciences, Pediatric Clinic, University of Brescia, Brescia, Italy; ^2^Clinical and Experimental Sciences, Immunology and Rheumatology Unit, University of Brrescia, Brescia, Italy

###### **Presenting author:** Marco Cattalini

**Introduction:** Progressive Pseudorheumatoid Dysplasia is a genetic disorder that may be misdiagnosed as Juvenile Idiopathic Arthritis (JIA). Patients typically show negative inflammatory markers, and no response to anti-inflammatory drugs or DMARDs.

**Objectives:** We present herein a case of pseudorheumatoid arthritis who developed secondary inflammatory arhtopathy, with good response to Methotrexate and anti-TNF treatment.

**Methods:** Case report

**Results:** P.E. is an albanian boy who came to our attention at the age of 13 for suspected arthritis.He was born at terms to non consanguineous healthy parents, his perinatal course was unremarkable and his development was normal until the age of 2, when he presented with walking difficulties due to malalignment of the left foot. He was then referred to orthopedic surgeons, a tibial CT-scan revealed a disostosic deformity and for this reason a tibial osteotomy with lengthening was perfomed at the age of 5 years.When he was 8 years old he began to experience arthralgia, mornig stiffness and his difficulties in walking severely deteriorated. The orthopedic surgeons performed multiple X-Rays, showing osteochondrosis of the right knee and chondrodysplasia of the hands and elbows. They run routine labworks, with ESR, CRP and CPK, which were normal. Because of the persistence of joint pain, swelling and stiffness, he was then sent to our attention: on clinical examination he showed disharmonic dwarfism, multiple joint deformities (with periarticular bone enlargement), kyphotic posture, and severe limitation in the range of motion of almost every joint. Given the clinical picture we hypothesized Progressive Pseudorheumatoid Dysplasia and started genetic testing. Meanwhile, since the patient was rapidly progressing with morning stiffness and joint tenderness we decided to start NSAIDs, with partial improvement. MRI of the pelvis showed large effusion with cartilage hypertrophy of the coxo-femoral joints, and signs of sacroiliitis. For this reason we hypothesized the co-occurence of inflammatory arthritis and started Methotrexate. Notably, the inflammatory markers were persistently negative. The patient responded with partial improvement of stiffness. We then decided to add Anti-TNF treatment with etanercept (50 mg/wk). The introduction of etanercept was followed by dramatic improvement: joint stiffness and pain resolved. Meanwhile genetic testing revealed the presence of two mutations in the WISP3 gene, already known to be associated with PPRD. Actually the disease is under good controll and therapy with etanercept have been lowered to 25mg/2wks.

**Conclusion:** PPRD is a skeletal dysplasia caused by recessive loss of function mutations in WISP3. Indeed, PPRD is a differential diagnosis of Juvenile Idiopathic Arthritis, since the metaphyseal enlarging may be wrongly interpreted as arthritis. Still, it has been described that a minority of patients with PPRD may present in the second decade of life with joint inflammation, secondary to the cartilage loss. This is, to our knowledge, the first report of a patient with PPRD, who developed secondary joint inflammation, that responded dramatically to Methotrexate and AntiTNF treatment.

**Disclosure of Interest**: None Declared

### B13 The follow-up study of children with positive anti modified citrullinated vimentin antibodies, who are out of character of the criteria of juvenile idiopathic arthritis

#### Nataliya Panko^1^, Nataliya Shevchenko^2^, Iryna Lebec^2^, Yevgeniya Zajceva^3^

##### ^1^Department of Pediatrics, V.N. Karazin Kharkov National University, Kharkov, Ukraine; ^2^Department of Cardiorheumatology, SE “Institute of Adolescence and children health care of National Academy of Medical Sciences”, Kharkov, Ukraine; ^3^Department of Cardiorheumatology, Kharkiv children’s city hospital N 24, Kharkov, Ukraine

###### **Presenting author:** Nataliya Panko

**Introduction:** Department of pediatrics, V.N. Karazin National University, Kharkiv, Ukraine. Juvenile idiopathic arthritis (JIA) is a chronic and socially significant disease as it leads to quick disability in childhood. Due to non-specific clinical manifestation of the disease at early stage, it has similarities with other rheumatic diseases, which can lead to diagnostic mistakes.

**Objectives:** To study the development of arthritis in children who have positive titer of anti-modified citrullinated vimentin antibodies (anti-MCV) and are out of character of the criteria of juvenile idiopathic arthritis (JIA).

**Methods:** The study involved 5 children within 2-13 years with clinical and ultrasonic signs of arthritis, who were difficult for diagnosis because had positive titers of anti-MCV and were out of character of the EULAR 2012 criteria of juvenile idiopathic arthritis. They had being evaluated over the following 5 years.

**Results:** The group of children consisted of 2 boys and 3 girls. All children had negative result of HLA-B27 and ANA. 9 years old boy was presented with arthritis of left knee, high titers of IgG to ureaplasma ureliticum in serum; he was diagnosed with reactive arthritis at the initial examination. Two years after, arthritis of left sternoclavicular joint and a bilateral sacroiliitis were developed and his diagnosis was changed in favor of juvenile spondylitis. 13 years old boy, who was engaged professionally in football from 6 years, with synovitis of the knee joints associated with dysplasia of knee joints and posttraumatic arthritis of the right hip joint was presented. One year after treatment by orthopedist only transient synovitis associated with dysplasia of knee joints was found. Over the following 5 years JIA did not develop in this patient. 2 years old girl was diagnosed with chronic hemorrhagic villous synovitis of right knee joint at the initial examination, synovectomy was carried out. Over the following 5 years she was healthy. 12 years girl with arthritis of knee joints and 11 years girl with arthritis of hip joints were presented. Both of them were diagnosed with hypothyroidism associated with autoimmune thyroiditis. One year after cessation of treatment with L-thyroxin and diclofenac both girls had remission of arthritis, which was diagnosed in one of girls after next 5 years without treatment also. After 3 years without treatment other girl had disease recurrence, which involved increased titers of thyroperoxidase antibodies, arthritis of hip joints and wrist joints bursitis, osteoporosis of bones of hands by X-ray. Her diagnosis was changed in favor of JIA, oligoarthritis, ANA - negative.

**Conclusion:** Positive titer of anti-MCV is not specific for JIA only, is the marker of affecting of joints, which caused by inflammatory and noninflammatory process. If positive titer of anti-MCV is found, joints should be investigated thoroughly.

**Disclosure of Interest**: None Declared

### B14 The effectiveness of foot-orthoses in children and adolescents with juvenile idiopathic arthritis-a pilot project vith randomized controlled design

#### Sara Rostlund^1^, Marie André^2^

##### ^1^Master program in Clinical Medical Science, Dep of Neurobiology, Care Sciences and Society, Karolinska Institute, Stockholm, Sweden; ^2^Physiotherapy unit, Karolinska University Hospital, Stockholm, Sweden

###### **Presenting author:** Sara Rostlund

**Introduction:** Children and adolescents with juvenile idiopathic arthritis, JIA, is often affected by inflammation and functional disability of the feet. A common intervention is to prescribe customized foot orhoses in order to improve footpositioning, reduce pain and improve function, all to create the best conditions for physical activity.

**Objectives:** The aim was to evaluate the effect of foot orthoses, used for seven to nine weeks in children och adolescents with JIA, regarding function, activity and participation. Additionaly explore if foot orthoses can reduce pain in the lower extremity and improve balance function.

**Methods:** This was a pilot-study with an experimental, randomized, controlled design. The population consisted av ten children and adeolescents wiht JIA, six in the intervention group and four in the control group. They all were clinically assessed to be in need for foot-orthoses in order to improve foot positioning and/or to reduce pain.Evaluation instrument consisted of self-reported questionnaires and a balance test.

**Results:** A significant improvement was found in the intervention group on the parts of the self-rated questionnaire Juvenil Athritis Foot disability Index, JAFI, regarding body function/disability, this dimension is mostly about footpain. No other significant differences related to activity and participation in JAFI, pain measured by visual analouge scale 0-100 mm and the balance could be established.

**Conclusion:** The pilot study showed that customized foot orthoses, used for seven to nine weeks, reduced self-rated foot related disability in children and adeolescents with JIA.

**Disclosure of Interest**: None Declared

### B15 Usefulness of joint ultrasonography in the treatment of juvenile idiopathic arthritis

#### Takuma Hara^1^, Takayuki Kishi^2^, Yumi Tani^2^, Aki Hanaya^2^, Takako Miyamae^1^, Satoru Nagata^2^, Hisashi Yamanaka^1^

##### ^1^Institute of Rheumatology, Tokyo Women’s Medical University, Tokyo, Japan; ^2^Department of Pediatrics, Tokyo Women’s Medical University, Tokyo, Japan

###### **Presenting author:** Takuma Hara

**Introduction:** Musculoskeletal ultrasonography is a non-invasive technique suitable for children with juvenile idiopathic arthritis (JIA). Musculoskeletal ultrasonography has contributed to clinical diagnoses and application of appropriate treatments as a specific and sensitive imaging modality.

**Objectives:** We report three cases involving Japanese children with JIA. The clinical contribution of joint ultrasonography in the treatment of JIA was assessed.

**Methods:** The predominant points of joint ultrasonography compared to conventional assessment methods, such as physical examination, x-ray, and the serum MMP-3 level during the clinical course, were evaluated. Ultrasonographic and color Doppler examinations were performed with a HI VISION Avius® (Model# EZU-MT-29-S1; Hitachi Medical Systems, Tokyo, Japan) equipped with a 14 MHz linear transducer.

**Results: Case 1** (5-year-old girl with polyarticular JIA). At the first visit, she did not exhibit shoulder joint pain and no abnormalities were found on physical examination; however, a prominent power Doppler (PD) signal was noted in both shoulder joints. In shoulder joints, findings consistent with arthritis are sometimes difficult to detect by physical examination. Joint ultrasonography was useful in the initial assessment of polyarticular JIA. **Case 2** (8-year-old girl with monoarticular JIA). She visited our institute after undergoing a synovectomy of the right elbow joint at another hospital. There was limited range of motion of the right knee, swelling and local heat within the joint. But the serum MMP-3 level was within the normal range and the clinical diagnosis of JIA was uncertain. Joint ultrasonography revealed synovial proliferation and a positive PD signal in the joint; the technique was useful as a unique method in the clinical diagnosis of JIA and evaluation of arthritis. **Case 3** (16-year-old girl with polyarticular JIA). Adalimumab, an anti-TNF monoclonal antibody, was administered at 14 years of age at another hospital. She attended our hospital at 17 years of age for evaluation of wrist pain. Based on the ultrasonographic findings, there was a moderate grade PD signal in the joint, and the biological DMARD was switched to tocilizaumab even though the serum MMP-3 level and X-ray findings were normal.

**Conclusion:** Joint ultrasonography is a sensitive technique in the treatment of JIA.

**Disclosure of Interest**: None Declared

### B16 Perplexing case of juvenile idiopathic arthritis with unilateral sternoclavicular involvement during the course of the disease

#### Velma Selmanovic^1^, Aida Omercahic-Dizdarevic^1^, Adisa Cengic^1^, Almira Cosickic^2^

##### ^1^Department for Allergology, Rheumatology and Clinical Immunology, Children’s Hospital University Clinical Center Sarajevo, Sarajevo, Bosnia and Herzegovina; ^2^Children’s Hospital University Clinical Center Tuzla, Tuzla, Australia

###### **Presenting author:** Velma Selmanovic

**Introduction:** Juvenile idiopathic arthritis (JIA) is not a single disease, but a group of related, phenotypically diverse immunoinflammatory disorders affecting joints and other structures. It could be quite perplexing disease with puzzling classification, unexpected disease course, joint involvement and therapy response

**Objectives:** to report an unusuall case 7,5y boy with JIA and sternoclavicular joint (SCJ) arthritis during the late course of the disease.

**Methods:** Retrospective review of medical charts.

**Results:** Disease onset was in April 2014 (age 7,5y) with 15 days of high-spiking fevers, arthritis of knees, ankles, right wrist, multiple small joints of hands and feet; dactylitis of 4th finger.Reffered to Children’s Hospital Sarajevo in June 2014. Laboratory revealed raised inflammatory markers, anemia, marked hypergammaglobulinemia, ANA -, HLA B27+, RF-. Generalised peripheral and mesenterial lymphadenopathy. No signs of entesitis, inflammatory bowel disease noor uveitis. Family psoriasis was negative. He was partially fullfilling crieteria for several forms of juvenile idiopathic arthritis. Initially treated as usuall (NSAR, pulses of metil-prednisolon, peroral steroids, metotrexat (MTX)) with partial response. Steroids were accordingly tapered. Five months later, patient experienced disease exacerbation (spiking fevers, active arthritis, raised inflammatory markers). Cytokine level were not determined. Biologics (tocilizumab) were not approved, so sulphasalasine was added plus treatment with intraarticular steroids. Remarkable improvement. Unfortunately, sulphasalasine had to be reduced soon due to leukopenia which was followed by arthritis exacerbation. In September 2015, tocilizumab was successfullly introduced with excellent clinical, radiological and laboratory response. Three months later (November 2015), significant left SCJ swelling occured. Immediate ultrasound examination revealed ……probable abscess of SCJ…cervical lymphadenopathy..“; MRI in december showed ……bony erosions of left clavicle immediatelly to SJC… zone of probable inflammatory infiltrate 10,5x9x9 mm immediatelly to SCJ..“. In January 2016, multidisciplinary team (rheumatologist, ortopedic surgeon, oncologist, infectologist) decided to stop tocilizumab, to perform biopsy of the SCJ biopsy as well as cultures for Mycobacterium tuberculosis (received negative). Patohistology revealed inflammatory arthritis consistent with JIA. Biologics were successfully re-introduced in March 2016 and showed remarkable effect so far : child is in clinicall, radiological and laboratory remission. He was finally smiling.

**Conclusion:** We reported a patient who had episodes of spiking high fevers, peripheral and mesenterial lymphadenopathy (suggestive for systemic form of JIA), agressive, polyarticular disease onset at age 7,5y, HLA B27+ positivity, dactylitis, significant improvement with added sulphasalasine - was suggesting entesitis-related (ERA)or psoriatic arthritis. SCJ arthritis occured when other joint were in remission. In adults, SCJ arthritis is usually seen in ERA patient. Treatment with anti-IL-6 showed remarkable improvement. JIA could be perplexing disease with unexpected disease course and therapy response

**Disclosure of Interest**: None Declared

### B17 Juvenile dermatomyositis; a case series report

#### Aida Omerčahić Dizdarević

##### Allergology,rheumatology and Clinical immunology, Childrens hospital, Sarajevo, Bosnia and Herzegovina

**Introduction:** Juvenile dermatomyositis (JDM) is rare, systemic autoimmune inflammatory muscle disease and vasculopathy primarily affecting skeletal muscle and skin.

**Objectives:** To describe the clinical features, laboratory data and therapy of patients with JDM hospitalized in our tertiary pediatric centre.

**Methods:** Medical records of 5 patients, diagnosed and treated in our department between 2008-2015 years, were retrospectively rewieved.

**Results:** Three of five children were boys. Mean age at disease onset was 5,2 years (3-11). Duration of symptoms, before hospitalization was 3,9 months (1,5-6). One child had previously infection with trichinella. Clinical manifestations were: weakness/myalgia (n = 4) (estimated by Childhood Myositis Assessment Scale- CMAS), malar rash (n = 5), Gottron’s papules (n = 5), heliotrope rash (n = 5), livedo reticularis (n = 1), vasculitis (n = 5), ulcerations (n = 1), arthritis (n = 2), limb edema (n = 1) and fever (n = 3). One child developed calcinosis during therapy. Internal organs were not affected. Laboratory data were: elevated serum muscle enzymes in four children (creatine kinase, lactate dehydrogenase, aspartate aminotransferase, alanine aminotransferase, aldolase). Periungual nailfold capillary changes were found in four children. Electromyography was done in 4 children, the results showed changes for myositis in all. Whole body MRI was done in 4 children and positive finding was found in all. Muscle/skin biopsy was done in 3 children and all results were suggestive for mysitis. Initial treatment was oral steroids or pulsed methylprednisolone, hydroxychloroquin and metotrexat. For three children, resistent to initial treatmen, additional pulsed methylprednisolon and intravenous immunoglobulin (IVIG) were given. One child was resistent on additional therapy and IV cyclophosphamide-IVCy/Rituximab were given. Two children are in remission without therapy (therapy lasted 3,5 years), two children are in remission on therapy (therapy is lasting 2,3 years) and one child is resistant on therapy (all specific myositis antibodies done-negative).

**Conclusion:** The most frequent initial manifestations were cutaneous and muscular. Muscle enzymes were elevated in mostly children. One child developed muscle calcinosis during therapy. Although diagnosed early and treated aggressive, responses to therapy were different. One child had refractory disease nevertheless multiple therapies and biological for refractory disease were applied.

**Disclosure of Interest**: None Declared

### B18 Juvenile dermatomyositis anti-p155/140 and anti-p140 antibody positive and severe persistent skin manifestations: two patients with the typical phenotype

#### Gemma Lepri^1^, Paolo Picco^2^, Clara Malattia^3^, Eleonora Bellucci^1^, Marco Matucci-Cerinic^1^, Fernanda Falcini^1^

##### ^1^Department of Rheumatology, Transition Care, University of Florence, Florence, Italy; ^2^Istituto Giannina Gaslini, Genova, Italy; ^3^Istituto Giannina Gaslini, Genoa, Italy, University of Genoa, Italy, GENOVA, Italy

###### **Presenting author:** Gemma Lepri

**Introduction:** Juvenile dermatomyositis (JDM) is characterized by symmetric and proximal muscle weakness, elevated serum muscle enzymes and typical rashes (Gottron’s papules, heliotrope discoloration of eyelids, malar rash, periungueal changes). Among the novel JDM-related antibodies (Abs),anti-p155/140 and anti-p140 have been reported to play a pathogenetic role in the dermatological lesions identifying a peculiar disease phenotype.

**Objectives:** To report two JDM pts with anti-p155/140 and anti-p140 Abs, characterized by a typical clinical presentation, severe and relapsing skin lesions with chronic course and muscle impairment.

**Methods: PT1**.4-year old girl presented with papule-erythematous cutaneous skin lesions on elbows and ears and a malar rash initially diagnosed as atopic dermatitis. 1month later she developed muscle weakness with increased creatine kinase (CK)(900U/L),lactate dehydrogenase (LDH)(1005U/L), aminotransferase(AST)(120UI/L), aldolase and ANA positivity(1:1280,granular pattern). The capillaroscopy showed megacapillaries with microhemorrhages. JDM was diagnosed: therapy with 3 pulses of methylprednisolone 30mg/Kg in 3consecutive days followed by prednisone 2mg/Kg p.o. daily, MTX 12,5mg/mq weekly and hydroxychloroquine 6mg/Kg daily was stareted with initial benefit on muscle weakness, but not on skin lesions. 6months later the disease relapsed: weekly steroids pulses were performed and 2months later muscle strength significantly improved but skin manifestations were exacerbated. Cyclosporine (2mg/Kg) was added, MTX was increased up to 15mg/mq/week, and steroid tapered. As the pt did not present benefit, cyclosporine was stopped and intravenous immunoglobulin (IVIg) 2gr/Kg were started with mild amelioration. Mofetil mycophenolate (1000mg/die) was added with only partial control of skin lesions and normal levels of muscle enzymes despite persistent muscle weakness. Following a flu, the pt had a severe relapse of skin symptoms, and a new cycle of steroid pulses with transient reduction of skin lesions was introduced. The last evaluation revealed a good control of muscular symptoms (RM STIR negative) despite persistent, remitting/relapsing skin lesions. **Pt2.**7-year old girl, had the onset of JDM with malar and heliotrope rash. Within few weeks, she developed rash on elbows and hands, weakness enabling to perform the activities of daily living. CK,LDH, aldolase and AST were increased and ANAs positive. JDM was diagnosed and 3steroid pulses (methylprednisolone 30mg/Kg/bolus) followed by prednisone 2mg/Kg in 2divided doses, MTX 15mg/mq/week and hydroxychloroquine were given. In both pts, the clinical phenotype was characterized by relapsing-remitting course with initial acute exacerbations of skin involvement. However, in this patient a good control of muscle involvement was not obtained and initially every time she presented skin lesions exacerbation, an increase of muscle serum levels was detected. For this reason, a new cycle of 3steroid pulses was performed and IVIg and Mofetil mycophenolate (1000mg/die) were started with only partial control of skin lesions and normal levels of muscle enzymes despite she still complains of muscle weakness.

**Results:** Our pts appear to confirm the possible relationship between some Abs and the phenotype of JDM. Namely, pts anti-p155/140 and anti-p140 Abs positive seem to develop a more severe disease course with a severe skin disease.

**Conclusion:** Anti-p155/140 and antip-140 Abs have to be checked in children with JDM as their presence could suggest a more aggressive therapy to reduce skin damage.Our limited experience seems to confirm recent results indicating a particular JDM phenotype associated to specific Abs.

**Disclosure of Interest**: None Declared

### B19 Membranous nephropathy in case of juvenile dermatomyositis

#### Margarita Dubko, Anton Solovyev, Elena Fedotova

##### Saint-Petersburg Pediatric Medical University, Saint-Petersburg, Russian Federation

###### **Presenting author:** Margarita Dubko

**Introduction:** Juvenile dermatomyositis is one of the most frequent inflammatory myopathy at children. It is characterized by typical cutaneous eruption and muscle weakness. Systemic features include fever, body weight loss, lymphadenopathy, Raynaud’s phenomenon etc. The most well-described organ involvement include interstitial lung disease, involvement of gastrointestinal tract. For a long time kidneys were considered to be the organ-exception at JDM. We suggest the case of JDM with membranous nephropathy.

**Methods:** A girl V.M., birthday *18.12.2006*

Diagnosis of juvenile dermatomyositis was made in 2010, at the age of 4. There were noted progressing muscle weakness, typical cutaneous eruption (heliotrope rash, Gottron’s erythema, capillaritis of palms and nail-beds), low-grade fever, arthralgia. Lungs involvement manifested by dyspnoea, zones of hypoventilation of both lungs at CT. Creatine phosphokinase increase to 1126, no registered immunologic activity (antinuclear antibody 1:160, microgranular glow type, antibodies to Mi- 2, Ku, Pm – Scl, Io-1, PL- 7, PL- 12 were not founded)

In the course of hormone-cytostatic therapy and IVIG, stable control over the disease has not been attained. In 2 years after the onset massive sites of calcification appeared in pelvic areas. Therapy was intensified with Remikade and Pomegara, and during 1 year the calcifications fully resolved.

Since 2013 microhematuria started to appear in urinary tests. The child was examined: normal concentration and excretory functions, normal filtration at Rehberg’s test. There were completed urography, cystography, cystoscopy, there are normal levels of blood coagulation and thrombocytes. The urogenital infections were excluded. Nephrobiopsy was taken in 12.2015.

**Results:** In two histological sections there were 26 increased glomerules. In course of Johns’ stain, glomerular basal membrane (GBM) was diffusively thickened, “dotted with spikes” kind, with tiny hollows. While IHC investigation along basal membrane of capillaries of the glomerulus (Pic. 1) and in the endothelium of the interstitium (Pic. 2) there is designated diffusive microgranular expression of IgG (+++). In normal cellular glomerules mesangial matrix is not extended; limbus penicillatus of nephrotelial of proximal canals is preserved.

**Conclusion:** Membranous nephropathy I-II stage.

**Disclosure of Interest**: None Declared

### B20 Etanercept treatment for a patient with refractory macrophage activation syndrome in juvenile systemic lupus erythematosus: 16-year old female. Case report and brief review of literature

#### Rocio Maldonado, Enrique Faugier, Ana Victoria Villarreal, Nydia Acevedo, Talia Diaz, Yuridiana Ramirez

##### Paediatric Rheumatology, Hospital Infantil de México Federico Gómez, Mexico City, Mexico

###### **Presenting author:** Yuridiana Ramirez

**Introduction:** Macrophage activation syndrome (MAS) is a serious, potentially fatal complication of childhood systemic inflammatory disorders, and it is most frequent in Systemic Juvenile Idiopthic Arthritis, for instance, it is increasingly reported in other pediatric rheumatic diseases as lupus erythematosus and Kawasaki disease. MAS may occur spontaneously, with a excessive activation and proliferation of T lymphocytes and macrophages with massive hypersecretion of proinflammatory cytokines, including interleukin- 1b, IL-6, interferon-g, and tumor necrosis factor a. A therapeutic protocol for MAS is not available: first line treatment is usually represented by parenteral administration of high dose corticosteroids. Mild forms are reported to respond to steroids alone in association with supportive medicaments. Steroid-resistant cases or the most severe forms of MAS require the addition of cyclosporine A, other therapeutic regimens have been studied such as high-dose intravenous immunoglobulins, antithymocyte globulins, etanercept, etoposide and plasmapheresis. We discuss a case of a 16-year old female with recent Juvenile Systemic Lupus Erythematosus and MAS refractory to systemic corticosteroids, cyclosporine A, etoposide. Etanercept is described as a treatment for MAS, so we start with Etanercept twice a day (Dose: 0.4mg/kg/dose) each week during 5 weeks, having an excellent response.

**Objectives:** The aim of this descriptive study, is the show the efficacy of Etanercept in refractary Juvenile Systemic Lupus Erythematosus.

**Methods:** Descriptive study, review of medical records.

**Results:** A 16-year-old adolescent with recent diagnosis of Juvenile Systemic Lupus Erythematosus who developed Macrophage Activation Syndrome at the same time. At admission with continuous fever, daily, predominantly during night, 102-104°F, lasting 2 months, with initial laboratory studies with Triglycerides 395 mg / dl, Ferritin 3.300 ug / l, soluble receptor IL-2 2.838 U / ml, fibrinogen 166 mg / dl, Hemophagocytos in bone marrow, presence of persistent cytopenias. Initial management with methylprednisolone 30 mg / kg / day (3 days) without clinical response. Cyclosporin A (10 mg / kg / day), was added to management, reporting subtherapeutic cyclosporine A serum levels despite high doses without clinical response and improvement of laboratory controls. She was admitted in the hospital durint three months of treatment with Etoposide (180 mg/dose), with ferritin levels in 2,350 ug / l, triglycerides 438, Fibrinogen 455 mg / dl, WBC 5,000 ul^-1^ (3,400 neutrophils, lymphocytes 1,050), platelet count 78 x 103 ml^-1^, hemoglobin 7.2 g/dl. After three months of treatment, Etanercept was initiated 0.4mg/kg/dose, twice a day. Currently in week 3 of treatment with WBC 6,500 ml^-1^, hemoglobin 9.7 g/dl, platelet count 140 x 103ml^-1^ and ferritin 4,270 ng/ml. The patient remains disease with costicosteroid, cyclosporine, and etanercept, without adverse events.

**Conclusion:** The diagnosis of MAS secondary to SLE is difficult due to common characteristics such as fever, pancytopenia, lymphadenopathy, neurological symptoms and skin manifestations. Dysregulation of macrophage-lymphocyte interactions leading to uncontrolled proliferation of highly activated macrophages and massive release of proinflammatory cytokines including tumor necrosis factor-alpha appears to be central to the pathogenesis of this syndrome. Until now the mainstay of therapy has been corticosteroids and cyclosporin A. We describe a patient with MAS and Juvenile SLE successfully treated with the anti-TNF agent etanercept. The outcome in this patient suggests etanercept might be an effective therapeutic agent in MAS.

**Disclosure of Interest**: None Declared

### B21 Relapsing polychondritis: interesting similarities between father and son

#### Teresa Giani, Achille Marino, Gabriele Simonini, Rolando Cimaz

##### Pediatric, A. Meyer Children Hospital, Florence, Italy

###### **Presenting author:** Achille Marino

**Introduction:** Relapsing polychondritis is an uncommon, multisystem, chronic, and life-threatening disorder mainly due to the possible laryngeal, tracheal and cardiovascular involvement. The pathogenesis is autoimmune. There is no specific diagnostic test or pathognomonic clinical, nor histological features.

The diagnosis can be considered conclusive in presence of at least three of the six features of the disease: 1) bilateral auricular chondritis; 2) nasal chondritis; 3) non-erosive seronegative inflammatory arthritis; 4) ocular inflammation; 5) respiratory tract chondritis; and 6) audiovestibular damage with or without histological confirmation.

**Objectives:** A 10-years-old Caucasian boy with a one-year history of recurrent swellings of both ears presented to our hospital’s pediatric rheumatology department. The patient complained unilateral or bilateral auricular chondritis, accompanied by swelling, discomfort and warmth of the skin, with the earlobe spared of any inflammation.

The episodes were intermittent and usually resolved spontaneously in one day. No precipitating factors could be identified. Two months before arriving to our attention, the boy started to present conjunctival hyperemia in association with ear maifestations.

The physical exam was unremarkable.

**Methods:** Full blood count, erythrocyte sedimentation rate, urea, and electrolyte were all normal. Autoantibody screen was negative.

Echocardiography resulted in normal limits.

**Results:** *Primary care physician referred the child* to our attention because his father showed an analogous ear involvement associated with episcleritis, sensorineural hearing loss, and pericarditis for which he received the diagnosis of polycondritis two years before.

**Conclusion:** Actually our patient presents some evocative aspects for polycondritis, however clinical picture is still incomplete, and a longer period of observation is required to confirm the diagnosis. The similarity of the manifestations in our patient and in his father is intriguing and suggested us to consider some genetic investigations, although familial clustering has not been described before.

**Disclosure of Interest**: None Declared

### B22 Deep venous trombosis and pulmonary embolism: a rare presentation of recurrent pancreatitis

#### Daniel Hunt, Muthana Al Obaidi^2^, Veli Veli^1^, Charalampia Papadopoulou^2^, Jochen Kammermeier^3^

##### ^1^Imperial College London, London, United Kingdom; ^2^Paediatric Rheumatology, Great Ormond Street Hospital and UCL Institute of Child Health, London, United Kingdom; ^3^Paediatric Gastroenterolog, Great Ormond Street Hospital and UCL Institute of Child Health, London, United Kingdom

###### **Presenting author:** Daniel Hunt

**Introduction:** Pancreatitis is an inflammatory disease caused by auto-digestion of the pancreas and the release of inflammatory mediators. Vascular complications of pancreatitis are well documented and understood; common complications include venous thrombosis (VT) and arterial haemorrhage which are present in 25% of pancreatic patients and can contribute significantly to the disease mortality and morbidity. Pulmonary Emboli (PE) as a result of pancreatitis has only been reported in a small number adult cases. To our knowledge, this is the first report of PE following pancreatitis in a paediatric patient.

**Objectives:**

Case presentation of a 14 year old girl with chronic pancreatitis, VT and PE. Review of the literature. Raise awareness of this possibly underreported consequence of the disease.

**Methods:** Retrospective review of medical notes, pathology and radiology databases from previous and current hospital admissions. A literature review was carried out.

**Results:** A 14 year old Caucasian female presented with acute abdominal pain, diagnosed as pancreatitis. She was treated with IV fluids and antibiotics. Over the next 6 months she had three episodes of pancreatitis, initially managed in the outpatient clinic with analgesia and oral hydration. Due to the recurrence of the symptoms, she was admitted and had further investigations where she was found to have left pleural effusion and ascites, an amylase of 1200 Units/ L (Lab reference 30-100Unit/L), C- Reactive Protein 40 mg/ L (Lab reference 0-20 mg/L) and splenomegaly. Normal IgG level, liver autoantibodies: negative, Lactate Dehydrogenase (LDH) 198 Unit/L (Lab reference 390-580 Unit/ L) normal, while she was also found to have positive lupus anti-coagulant (LAC).Magnetic Resonance Cholangiopancreotography (MRCP) was performed which revealed the presence of a pancreas divisum as well as a pancreatic pseudocyst.

Two weeks later, the patient presented with pain in her left arm, along with being warm, red and swollen. An ultrasound Doppler confirmed thrombi in the left axillary vein and subclavian and brachiocephalic veins for which she was started on a low molecular weight heparin, 10.000units OD. A month later she presented in A&E complaining of chest pain and difficulty breathing. A chest x-ray (CXR) revealed possible right lower lobe consolidation for which she was treated with IV antibiotics with mild improvement of her symptoms while furosemide was also added.

Due to the recurrent episodes of pancreatitis and positive LAC, she was referred to the rheumatology team for further investigations. During her admission, she had a CXR which found bilateral pleural effusion and due to her previous history a Computed Tomography (CT) Pulmonary angiogram was performed. It found several pleural PE distributed throughout the right lung, the largest being in the right upper lobe pulmonary artery

Extensive investigations were carried out which showed negative Anti-Nuclear Antibodies (ANA), MPO, PR3 and beta-2 glycoprotein IgG while LAC was equivocal. ECG was normal and an echocardiogram found there were no signs of infective endocarditis with structurally and functionally healthy heart. The LMWH dose was optimised.

**Conclusion:** Pulmonary thrombosis is a rare but potentially lethal complication of pancreatitis. It is unclear if the mechanism of action is the same than in the reported adult cases. Familiarity with this complication is important as early diagnosis and treatment can significantly reduce mortality and improve long term outcome.

**Disclosure of Interest**: None Declared

### B23 Joint pain and deformity of fingers and toes in 10 years old boy as first symptoms suggesting tricho-rhino-phalangeal syndrome

#### Edyta Olesińska^1^, Anna Poluha^2^, Jacek Postępski^3^

##### ^1^Department of Paediatric Pulmonology and Rheumatology, Children’s University Hospital, Lublin, Poland; ^2^Department of Medical Genetics, Medical University of Lublin, Lublin, Poland; ^3^Department of Paediatric Pulmonology and Rheumatology, Medical University of Lublin, Lublin, Poland

###### **Presenting author:** Edyta Olesińska

**Introduction:** Tricho-rhino-phalangeal syndrome (TRPS) is a very rare genetic disorder characterized by craniofacial and skeletal abnormalities. The syndrome is divided in TRPS I, caused by mutations in the zinc finger transcription factor (*TRPS1*), and TRPS II, caused by a contiguous gene deletion affecting TRPS1 and the gene for multiple hereditary exostoses (*EXT1*). TRPS III is a severe form of TRPS I, so may represent a spectrum of severity for specific mutations in TRPS1.

**Objectives:** To present the phenotype and genotype of patient with TRPS.

**Methods:** A 10-year-old boy was referred to Outpatients Paediatric Rheumatology Clinic with history of joint pain and progressive fingers deformity and brachydactyly of toes, of two years’ duration.

**Physical examination revealed**: a very short stature, abnormal body proportions (long trunk), sparse, fine hair, a pear-shaped nose, long and featureless philtrum, a thin upper lip, large protruding ears, erythema on chicks and chin, increased mobility of joints, ulnar deviation and clinodactyly of fingers without oedema, effusion or limitation of interphalangeal/metacarpophalangeal joints, “rocket” thumbs, brachydactyly of the third fingers and distal curvature of little fingers in both the hands, brachydactyly of the toes.

**His past medical history** was unremarkable.

**His parents** were not from related families. **His father and grandfather** presented with alopecia of the scalp, pear-shaped nose with bulbous tip, long philtrum, lateral sparse eyebrow, protruding ears, cartilaginous exostoses, brachy- and clinodactyly of fingers and toes; father was diagnosed with psoriasis.

**Laboratory findings** showed normal total blood count, markers of inflammation, kidney and liver function tests, but deficiency of 25(OH)D3. He was consulted by endocrinologist because of decreased linear growth concerning no hormone disorders.

**The X-ray findings demonstrated as follows:** scoliosis; AP view of the hand demonstrated the shortening of the middle phalanges of third fingers with metaphysis widening, partial connection of metaphysis with epiphysis; skeletal age indicated 13 years of age; AP view of the foot was demonstrating brachydactyly of toes with metaphysis widening ; AP view of the hips demonstrated shortening of both femoral neck and greater angle of both femoral neck

The joints USG revealed neither synovial hypertrophy nor destruction.

Densitometry (DEXA) revealed osteopenia.

Chromosomal analysis of the patient revealed normal karyotyping (46, XY). Genetic molecular analysis revealed mutation in *TRPS1* c.2179_2180delGT.

**Results:** The diagnosis of tricho-rhino-phalengeal syndrome type 1 was confirmed.

**Conclusion:** Tricho-rhino-phalangeal syndrome must be considered in differential diagnosis of child with skeletal abnormalities, hypermobility and growth impairment.

**Disclosure of Interest**: None Declared

### B24 Case report of primary erythromelalgia in a child-challenges in management

#### Gangadhara C. Bharmappanavara^1^, Alison Kelly^1^, Lindsay Shaw^2^

##### ^1^Paediatric Rheumatology Department, Bristol Royal Hospital for Children, Bristol, United Kingdom; ^2^Department of Dermatology, Bristol Royal Hospital for Children, Bristol, United Kingdom

###### **Presenting author:** Gangadhara C. Bharmappanavara

**Introduction:** Erythromelalgia is a rare disorder, characterized by triad of episodic erythema, intense burning pain and warmth, typically affecting the extremities and when chronic, associated with significant disability. Episodes lasting for minutes to hours are triggered by warm environment and/or physical exertion and often relieved by cooling.

**Objectives:** Erythromelalgia is challenging to manage as pain is often refractory to treatment.

**Methods:** A case of 6 year old girl with episodes of pain with warmth, redness of her feet, precipitated by heat, activity, and classically relieved by ice packs. She had an episode of seizure. There was no family history. Borderline persistent tachycardia and hypertension were recorded even when pain free. She appeared well, with normal systemic examination. Investigations were done for exclusion of secondary causes. Genetic tests revealed sodium channel heterozygous sequence change in the SCN9A gene. She constantly used ice packs and fan for symptomatic relief. There was evidence of chronic contact injury to the skin of her feet which was red, thickened and ulcerated. She could not participate in school or social activities and family environment was stressful with poor quality of life. Psychologist developed strategies to help her cope with the pain. Trials of various combinations of medications failed to relieve her pain and improve sleep pattern. Medications used were ibuprofen, naproxen, cetirizine, chlorpheniramine, alimemazine, melatonin, chloral hydrate, tramadol, gabapentin, mexilitine, carbamazepine, amitriptyline, Magnesium aspartate and clonidine. Oral Mexiletine dose was optimised over 2weeks to 100mg eight hourly.ECG was done regularly to monitor QTC intervals. Mexiletine levels remained stable. Mexiletine was only partially effective in reducing the frequency and severity of the painful episodes. This may be due to the heterozygous gene mutation being associated with a large change in neurophysiology compared with other mutations.

**Results:** SCN9A-related primary erythromelalgia is associated with mutation of SCN9A gene that encodes Nav1.7, a voltage-gated sodium channel alpha subunit, expressed primarily in sensory and sympathetic ganglia. Nav1.7 shares significant similarity with other isoforms of sodium channel alpha subunit in the brain, peripheral nerve,muscle, and myocardium. Mutations in these ion channels have been related to epilepsy, periodic paralysis, and long Q-T syndrome.^1^ Inheritance is autosomal dominant and the penetrance in families reported to date is 100%. No accurate data on the worldwide prevalence of SCN9A-related IEM is available. Onset is usually in childhood or adolescence with bilateral and symmetric involvement of the extremities.^2^ Though diagnosis is made clinically, treatable secondary causes should be excluded. Management is challenging as pain is often refractory to treatment. In a review of data for 168 patients with erythromelalgia at the Mayo clinic, patients had used 84 different types of medications.^3^ Various treatment modalities, including tricyclic antidepressants, anticonvulsants, clonidine, opioids, lidocaine patches, epidural infusions, sodium nitroprusside infusions, and gabapentin have been tried with varied success.

**Conclusion:** Most patients’ symptoms will progress overtime and develop self-mutilating behaviours or tissue damage such as ulceration, necrosis, and gangrene of affected extremities. Patients’ quality of life is greatly compromised with disability.^4^ Treatment is individualized depending on the response as no controlled trial has compared the medications and effectiveness. Further pharmacological studies are required.

Parents consented for publication.

**References:**

1) Yang Y, Wang Y, Li S, Xu Z, Li H, Ma L, et al. Mutations in SCN9A, encoding a sodium channel alpha subunit, in patients with primary erythermalgia. J Med Genet. 2004;41:171–4. doi:10.1136/jmg.2003.012153.

2) Hisama FM, Dib-Hajj SD, Waxman SG.SCN9A-Related Inherited Erythromelalgia, GeneReviews® [Internet]. http://www.ncbi.nlm.nih.gov/books/NBK1163 (Accessed-19 May 2016)

3) Davis MDP, O’Fallon MW, Rogers RS, et al. Natural history of erythromelalgia: presentation and outcome in 169 patients. *Arch Dermatol.*2000;136:330–336

4) Zhaoli Tang, Zhao Chen, Beisha Tang and Hong Jiang, Primary erythromelalgia: a review Tang et al. Orphanet Journal of Rare Diseases (2015) 10:127. doi:10.1186/s13023-015-0347-1

**Disclosure of Interest**: None Declared

### B25 Unusual presentation of sarcoidosis in a girl: asthenia, weight loss and hepatomegaly, in absence of respiratory symptoms

#### Teresa Giani^1^, Giovanna Ferrara^2^, Michele Luzzati^1^, Achille Marino^1^, Mattia Giovannini^1^, Gabriele Simonini^1^, Rolando Cimaz^1^

##### ^1^Pediatric, A. MEYER CHILDREN HOSPITAL, Florence, Italy; ^2^Pediatric, Burlo Garofalo, Trieste, Italy

###### **Presenting author:** Giovanna Ferrara

**Introduction:** Sarcoidosis is a systemic granulomatous disorder of unknown etiology that involves multiple organs, with pulmonary findings in more than 90% of patients, that is characterized by non-caseating granuloma. The paediatric disease onset is rare and often underdiagnosed due to the lack of specific signs and symptoms, and the real incidence from birth to adolescence is unknown. *Two distinct* forms of *sarcoidosis* exist in children: the early-onset that is a unique form of the disease characterized by the triad of rash, uveitis, and arthritis, and the classical form characterized by involvement of lungs, lymphnodes and eyes as seen in adult.

**Objectives:** Here we present the case of a 12-year old girl presenting to her p*rimary care physician* complaining asthenia and weight loss. Physical examination revealed a painless hepatomegaly. Laboratory tests were normal. Abdominal ultrasound revealed an inhomogeneous liver aspect, with multiple solid neoformations. MRI of the superior and inferior abdomen with contrast confirmed nodular zones of altered signal in liver, inter-aorto-caval lymphadenopathies, and splenomegaly in association with multiple nodular formations. A ultrasound-guided biopsy highlighted chronic granulomatous lesions typical for sarcoidosis at liver and lymph nodes. High-resolution chest CT with contrast shows widespread fibrous strands and multiple enlarged lymph nodes and analysis of the broncho-alveolar lavage fluid revealed a CD4/CD8 ratio of 9.2.

**Conclusion:** The final diagnosis required a process of exclusion first of all of tuberculosis, other infections, and lymphoma. Our patient presented only few, and unspecific manifestations, in absence of typical although not diagnostic laboratory findings of sarcoidosis such as ACE, lysozyme, calcium. Once ruled out other diagnoses and in presence of suggestive histological findings in two different tissues (liver and lymph nodes), the diagnosis of paediatric-onset adult sarcoidosis was finally postulated and a steroid and mycophenolate mofetil treatment was started.

**Disclosure of Interest**: None Declared

### B26 Hypercalcemia and renal injury in pediatric sarcoidosis: a case report

#### Liliana Jurado^1^, Sebastian Giraldo^2,3^, Juliana Chamorro^4^, Lorena Sarmiento^5^, Adriana S. Diaz^6,7^

##### ^1^School of Medicine, Universidad Nacional de Colombia, Bogota, Colombia; ^2^Rheumatology and Inmunology, Hospital Militar Central, Bogota, Colombia; ^3^School of Medicine, Universidad Militar Nueva Granada, Bogota, Colombia; ^4^Pediatric Nephrology, Hospital de la Misericoria, Bogota, Colombia; ^5^School of Medicine, Fundación Universitaria Sanitas, Bogota, Colombia; ^6^Rheumatology, Hospital de La Misericordia, Bogota, Colombia; ^7^Rheumatology, Care for Kids, Bogota, Colombia

###### **Presenting author:** Liliana Jurado

**Introduction:** Sarcoidosis is a multisystemic chronic granulomatous disease of unknown etiology, relatively rare in the pediatric population. Two classical clinical forms are distinguished: early and juvenile onset, however the clinical spectrum is very wide. Diagnosis requires high index of suspicion plus clinical findings as hypercalcemia and hypercalciuria, elevated angiotensin converting enzyme, and compatible histological findings.

**Objectives:** a case of a male patient diagnosed with sarcoidosis who makes predominance renal clinic given by hypercalcemia and hypercalciuria with disease activity and demand early intervention for management, uncommon clinical manifestation in this disease occurs.

**Methods:** To present the case of a male patient of 17 years with documentation of focal segmental glomerulosclerosis and pulmonary nodules with histological diagnosis of Sarcoidosis, who was admitted with severe chronic hypercalcemia (serum calcium 16.2 mg / dl), significant proteinuria and hyperuricemia (uric acid 11.7 mg / dl) in the context of the underlying disease and vitamin D replacement. Was admitted with renal replacement therapy criteria, however received hyperhydration, allopurinol, furosemide, systemic corticosteroid and anti-hypertensive, showing significant improvement so dialysis was avoided. Nuclear magnetic resonance documented myositis of extraocular muscles due to sarcoidosis so methotrexate treatment was started, showing good evolution.

**Results:** The disease has a wide spectrum of presentation. At renal level described 23 - 48% of patients, with granulomatous noncaseating interstitial nephritis classical injury, but there are other variety of less common lesions such as focal segmental glomerulosclerosis presented in our case. In a metabolic aspect, alpha 1 hydroxylase increases the serum and urinary calcium (10% to 17% of patients) a phenomenon not susceptible to normal regulatory mechanism. Renal damage entity is more related to hypercalcemia and hypercalciuria tan with direct renal granulomatous infiltration, promoting decreased glomerular filtration rate, tubular cell necrosis, loss of concentration and urinary calcium deposit progressive. The most common ocular involvement in sarcoidosis is uveitis, however, it has been reported commitment damage of the orbit and its annexes, but is rare. The presentation of this case illustrate these phenomena.

**Conclusion:** Sarcoidosis is a systemic disease with a heterogeneous behavior, which requires a high level of suspicion for diagnosis and treatment, chronic judicious maintenance, and close monitoring of possible complications. The decision of vitamin D supplementation in these patients should be individualized and have close monitoring practice. It requires an interdisciplinary management, including pediatrics, rheumatology, nephrology and others as the clinical commitment, to give possibility of dynamic patient management.

**Disclosure of Interest**: None Declared

### B27 Scurvy as a cause of musculoskeletal pain: a case report

#### Veronica Medeghini, Francesca Ricci, Paola Montesano, Barbara Bonafini, Ilaria Parissenti, Antonella Meini, Ester Conversano, Marco Cattalini

##### Clinical and Experimental Sciences, Pediatric Clinic, University of Brescia, Brescia, Italy

###### **Presenting author:** Marco Cattalini

**Introduction:** Musculoskeletal pain is a very common complaint in children, and a frequent reason for pediatric referral. The differential diagnosis is wide, encompassing metabolic, traumatic, inflammatory, hemato-oncologic and genetic causes. Scurvy is a deficit of vitamin C that may manifest with ostheoarticular symptoms. The disease is very rare in developed countries, where malnutrition in the pediatric age is not a common problem, still the pediatrician should consider it in the differential of a child with musculoskeletal complaints.

**Objectives:** We present herein the case of a child diagnosed with scurvy, secondary to a very limited diet.

**Methods:** Case report.

**Results:** S. is a three and half years boy who presented to our emergency room for the onset in the last 5 days of an abnormal gait and daily fever. On physical examination we confirmed the limping and a bended position of the trunk. In the prone position the child tended to maintain knee flexed and hips flexed and extrarotated, with a “frog-like” position. There was a slight swelling of the right knee, without joint effusion or other signs of inflammation. S. looked pale and had petechiae on the trunk with swollen and bleeding gums. The child’s weight was under the 3rd percentile and the height between the 3rd and 10th percentile. On medical history the parents didn’t report recent injuries but backpain was present during the last month, exacerbated by the standing position and exercise. Blood tests showed only mild normochromic and normocytic anemia and modest elevation of ESR (35 mm/h). A lower limbs X-Ray was done and reported as negative, we obtained also a lumbar spine MRI to rule/out spondylodiscitis or tumors. MRI showed minimal hyperintensity signal in the femoral metaphysis and in the sacral synchondrosis. After further questioning we found out that Simone’s diet included only milk, cookies and sometimes raw tomatoes. Given this information and the clinical picture we dosed vitamin C serum levels that was compatible with a deficiency state (11 umol/L). We immediately introduced a C vitamin supplementation; the day after the fever was gone and joint pain and back pain resolved within 15 days. Given the diagnosis we looked again at the X-Ray, finding hyperlucent areas in right femur distal metaphysis that were previously overlooked, compatible with the diagnosis of scurvy.

**Conclusion:** Although scurvy is very rare in developed countries, children with very restricted diet may develop it. The pediatrician should maintain a high awareness on this disease and consider it in the differential of a child with musculoskeletal complaints, especially if associated with skin or mucosal lesions.

**Disclosure of Interest**: None Declared

### B28 Case report of a child with persistent lameness

#### Maria Francesca Gicchino, Giulia Macchini, Carmela Granato, Assunta Tirelli, Alma N. Olivieri

##### Department of Women, Chid and General and Specialistic Surgery, Second University of Naples, Naples, Italy

###### **Presenting author:** Maria Francesca Gicchino

**Introduction:** Lameness is a symptom very common in childhood, it could depends on infective, orthopedic, neoplastic diseases.

**Objectives:** to describe a case of persistent lameness in a 36 old month child.

**Methods:** R. 36 months, was admitted to our hospital because of persistent lameness. The symptomatology began four months previously following a trauma. The child underwent X-ray and right hip ultrasound, both negative. For persisting lameness and pain the child was checked by an orthopedist who diagnosed “hip transitory synovitis” and prescribed therapy with NSAIDs with improvement of symptoms. After some time the child’s symptomatology reappeared. When he came to our observation the articular examination revealed pain, functional limitation of the right hip and lameness. Erythrocyte sedimentation rate (ESR) and C-reactive protein (CRP) were normal. Suspecting a Perthes disease another right leg radiography was performed which documented a remodeling area with an osteolytic lesion near the big trochanter. The CT scan confirmed the osteolytic lesion with sclerotic regular margins eroding the cortical bone. Scintigraphy performed with tc99 showed a solitary bone lesion with a slightly increased metabolism. We decided to perform a needle biopsy to define the diagnosis. Histologic examination revealed different type of immune cells (lymphocytes, histiocytes, plasma cells, granulocytes). The bone lesion swab was positive for “Peptostreptococcus Anaerobius”.

**Results:** According to symptomatology, laboratory, instrumental tests and bone biopsy Chronic Osteomyelitis of the big trochanter of the right femur was diagnosed. Treatment with Imipenem (200mg ev, 4 times a day) for 15 days, rapidly improved the symptoms; therapy was continued with amoxicillin-ac.clavulanico (50mg/kg/die divided in three doses) for 15 days. During the follow up the child no longer experienced lameness and pain at the right leg. An X-ray of the right hip performed a year later documented the resolution of the area of osteolysis.

**Conclusion:** Lameness is very frequent in childhood, it could be secondary to inflammatory, neoplastic, infective, orthopedic conditions, so an accurate differential diagnosis is very important. Osteomyelitis is an infection of the bone, it could be acute (<2 weeks), subacute (>2 weeks <3 months), chronic (>3 months), mainly secondary to bacterial infection. It is most often hematogenous in origin but may result from trauma.

**Disclosure of Interest**: None Declared

### B29 DRESS syndrome with the suspected underlying autoimmune disorder

#### Marija Perica, Lana Tambić Bukovac

##### Department of Pediatric and Adolescent Rheumatology, Children’s Hospital Srebrnjak, Zagreb, Croatia

###### **Presenting author:** Marija Perica

**Introduction:** Drug Reaction with Eosinophilia and Systemic Symptoms (DRESS) syndrome is a rare, life-threatening hypersensitivity drug reaction. Patients present with rash, fever, lymphadenopathy, hematologic abnormalities and visceral organ involvement. Annual incidence is estimated to 0.9/100,000 with 10% mortality rate. The most common causative agent underlying DRESS syndromes are anticonvulsant drugs.

**Objectives:** Our aim is to present a case of a 13 year-old-girl who developed DRESS syndrome to lamotrigine, with possible underlying autoimmune disorder.

**Methods:** Patient started lamotrigine therapy due to persistent headache with paroxysmal EEG abnormalities. Seven weeks after therapy introduction, patient presented with fever, generalized rash resistant to antihistamine therapy, facial oedema, lymphadenopathy, hepatomegaly. Laboratory findings revealed leucocytosis, atypical lymphocytosis, eosinophilia, hypoproteinemia, elevated serum transaminases and creatinine kinase, low C3 level and weak ANA positivity. Skin biopsy analysis was consistent with eosionphilic inflammatory infiltrate. Serologic viral studies were negative. Due to DRESS diagnosis (score 8 on scoring system by *Kardaun et al*) lamotrigine was ceased and patient received 0,5 g/kg of IVIG, …pulse“ steroid therapy and dual antibiotic therapy due to *Staphylococcus aureus* sepsis.

**Results:** Due to rash, muscle weakness, elevated CK levels, low levels of C3 and weak ANA positivity, patient was suspected to have an underlying autoimmune disorder; such as dermatomyositis or an overlap syndrome. However, muscle biopsy and EMNG were postponed while the patient developed acute respiratory failure due to hemothorax, requiring mechanical ventilation and drainage. With continuous IVIG and corticosteroid therapy, patient’s general condition gradually improved. As the patient developed unilateral anisocoria, a MR was performed, showing no abnormalities.

With the improvement of general condition, corticosteroid therapy was gradually lowered and, at discharge completely discontinued. Few days after discharge, the patient had the first relapse of discrete skin rash with febrile episodes and headache which diminished while on 1,5 mg/kg of prednisone. Two months after the relapse, while on low dose of corticosteroid therapy, the patient had a similar relapse. During both relapses, the elevation of creatinine kinase was not detected. Repeated full immuno-rheumatological screening panel was negative including ANA, but EMNG revealed pattern consistent with myositis or chronic neuropathy. Currently, eight months after the first symptom manifestation, patient is on continuous low dose corticosteroid therapy.

**Conclusion:** We present a patient with DRESS syndrome after lamotrigine therapy and possible underlying autoimmune disorder. Considering short follow up period, we believe that repeated diagnostic tests at discontinuation of corticosteroid therapy will resolve our diagnostic dilemma.

**Disclosure of Interest**: None Declared

### B30 Deficiency of vitamin D in patients with onset of rheumatic diseases

#### Ludmila F. Bogmat, Nataly S. Shevchenko, Marina V. Demyanenko

##### Department of Cardiorheumatology, Institute of Children and Adolescents Health Care, Kharkiv, Ukraine

###### **Presenting author:** Nataly S. Shevchenko

**Introduction:** Vitamin D deficiency in rheumatic diseases (RD) is one of the factors, causing the development of osteopenia and its complications (spontaneous fractures or fractures at a light load) due to its regulation of calcium metabolism. It is widely accepted that calcium is involved in the significant physiological processes in most of the cells of the organism; it also regulates secretion of a number of key hormones, enzymes and proteins. All the above serves as a reason for the prescription of preventive doses of vitamin D metabolites to ensure calcium effective absorption, when Ca-containing medicines are included in the treatment programs of patients with RD.

**Objectives:** To determine the content of the active metabolite of vitamin D in the blood of children with RD, namely: systemic lupus erythematosus (SLE), dermatomyositis (DM), juvenile rheumatoid arthritis (JRA)) as well as its relationship with the severity of the pathological process.

**Methods:** The level of 25-OH- vitamin D3 was determined by electro chemiluminescent immunoassay (ECLIA) in 17 patients, aged 9-18 years, (mean age – 12.9 ± 4 years), predominantly girls (82.4%) with JRA (58.8%), DM (29.4%), and SLE (11.8%). Values, exceeding 50 ng / ml, were regarded as normal, in the range of 30-50 ng / ml (due to nutritional deficiency) as reduced, and below 30 ng / ml as low.

**Results:** The age of the disease onset in children was 9.3 ± 2.5 years, and duration of the disease averaged 2,7 ± 1,3 months. Glucocorticoid therapy received 58.8% of the patients, cytostatic (cyclophosphamide and methotrexate) were given to 70.6% of patients, all of our patients were treated with calcium medicines containing vitamin D (daily dose 400 IU). The level of 25-OH -vitamin D3 in all the patients was reduced, while in 17.6% their findings were above 30 ng / mL, and in 82.4% its absolute deficiency was registered. Blood values of 25-OH -vitamin D3 came to 22.9 ± 2.4 ng / mL (16.1 - 32.5 ng / mL), they correlated with age of patients(r = 0,89, p <0.01) and were not dependent on the RD nosology. There is not significant correlation between the degree of vitamin D deficiency and the immunological and biochemical indices of the disease activity in its early stages.

**Conclusion:** Investigations, carried out in the study, have shown a pronounced deficiency of the main metabolite of vitamin D in children with RD, despite the intake of its medications in the combined treatment. This necessitates an additional prescription of the D3 active metabolite for children with all key RD diseases, especially for the younger children.

**Disclosure of Interest**: None Declared

### B31 Sarcoidosis associated with sjogren syndrome

#### Reza Sinaei, Vadood Javadi Parvaneh, Reza Shiari, Khosro Rahmani, Fatemeh F. Mehregan, Mehrnoush Hassas Yeganeh

##### Pediatric Rheumatology, Mofid Children’s Hospital, Tehran, Islamic Republic of Iran

###### **Presenting author:** Reza Sinaei

**Introduction:** Sarcoidosis and sjogren syndrome are chronic inflammatory conditions with multisystem and extensive features and sometimes their differentiation can be challenging.

**Objectives:** We would like to report a 14-years-old girl who presented with generalized lymphadenopathy and fever, cough, weight loss, skin lesions, parotitis, red eyes and dry mouth.

**Methods:** Physical examination revealed submandibular lymphadenopathy, tiny scaling rashes, flexion contracture of both knees and parotid gland enlargement. Blood investigations showed mild anemia, leukopenia, predominantly eosinophilia, thrombocytopenia, hypergammaglobulinemia, and high erythrocyte sedimentation rate and C-reactive protein. Computed tomography of the thorax and abdomen showed bilateral submandibular, mediastinal and para-aortic lymphadenopathy, septal thickening and diffuse mosaic pattern with a ground glass appearance of lungs, and also splenomegaly. Anti Ro (SSA) and Angiotensin Converting Enzyme (ACE) were significantly elevated. Work up for mycobacterium tuberculosis was negative. CT sialography showed sialectasis. Later, histopathological examination of the mediastinal lymph node, skin and lip revealed non-caseating granuloma, consistent with sarcoidosis. Finally, after diagnosis of sarcoidosis and sjogren syndrome, she was prescribed prednisolone 12.5 mg daily with subsequent reduction in the size of cervical lymphadenopathy and parotid swelling. The patient also treated for sarcoid hepatitis and other manifestations with azathioprine, ursodeoxy cholic acid and vitamins.

**Results:** after six months follow up, the patient was in full remission and her complaints were controlled with prednisolone 5mg daily and azathioprine 2mg daily.

**Conclusion:** On the basis of our knowldege, there are a few cases of pediatric sarcoidosis and sjogren syndrome in the litratures;therefore, in cases with chronic inflammatory involvement of multisystems such as exocrine, respiratory, digestive, skin and muskuloskeletal systems this diagnosis should be considered.

**Disclosure of Interest**: None Declared

### B32 Macitentan treatment in a patient with persistent digital ulcer (DU) and mixed connective tissue disease (MCTD)

#### Inmaculada Calvo Penadés, Berta López Montesinos, Ma Isabel González Fernández, Adriana Rodríguez Vidal

##### Hospital Universitario Policlínico La Fe, Valencia, Spain

###### **Presenting author:** Inmaculada Calvo Penadés

**Introduction:** Digital ulcers may appear in different forms of connective tissue diseases, including systemic sclerosis, systemic lupus erythematosus (SLE) and MCTD. These lesions adversely affect the patient’s ability to perform work and daily activities, particularly those associated with fingertip functions.

**Objectives:** To report a clinical case of a patient with persistent digital ulcer treated with macitentan.

**Methods:** A 12-year-old male patient was referred to our Pediatric Rheumatology Unit after presenting a proximal interphalangeal polyarthritis in both hands and Raynaud phenomenon (RP), with suspected SLE. The main laboratory findings were ESR: 47 mm/h; GOT: 178 IU/L; CPK: 1497 IU/L; aldolase: 39 IU/L; ANA: 1/1280; RNP > 200; and Anti-Sm positive. Kidney function was normal. The electromyogram showed non-acute myophatic and neurogenic features, constituting a mixed pattern. The spirometry pattern did not show signs suggestive of pulmonary hypertension. Capillaroscopy results revealed a pattern of sclerodermiform characteristics (megacapillars, avascular areas and multiple haemorrhages). Based on these results, the diagnosis of MCTD was established. The initial treatment was low-dose prednisone, hydroxychloroquine and methotrexate.

During follow-up, the patient presented several fever outbreaks with articular affection treated with azathioprine as an oral corticosteroid sparing agent. After two years of disease onset he presented RP worsening, starting oral nifedipine treatment, and preischemic digital lesions evolving to DU, consequently starting bosentan (62.5 mg BID initially, titrated to 125 mg BID afterwards) resulting in DU enhancement. Afterwards, he presented an acute chest pain with echocardiographic findings of acute myopericarditis treated with acetylsalicylic acid at anti-inflammatory doses and mycophenolate mofetil subsequently.

**Results:** Despite bosentan treatment, DU reappeared after 6 months in the previous location with torpid evolution, and treatment was switched to macitentan 10 mg/day due to bosentan lack of response, with good tolerability, controlled transaminase levels and normal Hb. The patient was treated with nifedipine, metamizole, ibuprofen and local vitamin E during the whole process. He required amoxicillin/clavulanic treatment due to bacterial superinfection. The patient experienced a DU improvement after 2 months, with complete healing after 4 months.

**Conclusion:** In this MCTD/DU patient lacking bosentan response, macitentan resulted in an effective and safe therapeutic option, without hepatotoxicity or other secondary effects.

**Disclosure of Interest**: None Declared

### B33 Mycophenolate mofetil induced transfusion dependent anemia in a patient with SLE

#### Anand Prahalad^1^ Rao, Ayesha Romana^2^, Jyothi raghuram^1^

##### ^1^Pediatric Rheumatology Clinic, Bangalore, India; ^2^Indira Gandhi Institute of Child health, Bangalore, India

###### **Presenting author:** Anand Prahalad Rao

**Introduction:** SLE is a multisystem disease and it sometimes can be associated with anemia.Anemia tends to be mild and is related to the disease process usually, though rarely, drugs used in the treatment of SLE may sometimes be implicated.

**Objectives:** To demonstrate that SLE can be associated with mycophenolate mofetil induced transusion dependent anemia and that it is a reversible phenomenon on cessation of the drug.

**Methods:** We describe the case of a 17 year-old girl, who presented with SLE and was diagnosed to have renal involvement (Class 4 lupus nephritis). She was started on immunosuppression with steroids and MMF. Within a few days of starting MMF she started having transfusion dependent anemia. She was transfused thrice in a matter of 2 weeks. Her corrected reticulocyte count and reticulocyte production index were very low and her direct Coomb’s test (DCT) was negative. She was subjected to a bone marrow biopsy, which revealed features of depletion of erythroid precursors with normal leukocyte and platelet precursors. The discontinuation of MMF led to a recovery of anemia.

**Results:** MMF was implicated in this child to cause severe transfusion dependent anemia by temporal sequence of events and it’s withdrawal led to a rapid improvement of anemia.

**Conclusion:** Anemia in a child with SLE might not only be related to the disease process alone. It is important to keep in mind the drugs the patient is currently on, because that is where the cause might be hidden.

**Disclosure of Interest**: None Declared

### B34 An unusual case of pediatric lupus with isolated retinal involvement

#### Ankur Kumar^1^, Deepti Suri^1^, Vishali Gupta^2^, Amit Rawat^1^, Surjit Singh^1^

##### ^1^Pediatrics, PGIMER, Chandigarh, India; ^2^Ophthalmology, PGIMER, Chandigarh, India

###### **Presenting author:** Ankur Kumar

**Introduction:** Systemic lupus erythematosus (SLE) is a chronic, autoimmune, multisystem disease associated with variable and often unpredictable clinical course.About one third of patients with SLE have some form of eye involvement. SLE can involve almost any part of ocular system. Keratoconjunctivitis sicca is the most common manifestation of ocular involvement in lupus while the most common vision threatening manifestation is optic nerve or retinal involvement.

**Objectives:** To describe the case of a young boy who presented with an unusual manifestation of lupus with retinal involvement.

**Methods:** A 9 year old boy presented with complaints of high grade, continuous fever for 15 days associated with vomiting for 8 days. He also had multiple episodes of generalized tonic clonic seizure and alteration of sensorium in last 3 days before admission. There was no significant past history and he had normal development. At presentation, he had poor GCS (8/15) with signs of raised intracranial tension (ICT). Initial possibilities considered were viral meningoencephalitis, enteric fever with CNS complications, tubercular meningitis and scrub typhus. He was intubated and ventilated for 10 days in view of raised ICT. He was initiated empirically on Injectable ceftriaxone and acyclovir; and investigated for an underlying etiology. He had transient thrombocytopenia and leucopenia with lymphopenia which recovered spontaneously. CECT head and MRI brain were normal. All infectious disease workup and CSF examination was normal. However he had cotton wool spots in both eyes detected on a bedside fundus evaluation by direct ophtalmoscopy.

Subsequently a possibility of SLE was also considered and his antinuclear antibody (ANA) test was positive (4+ speckled pattern). His sensorium continued to improve and he was extubtaed on day 10 of hospital stay.

After extubation, he was found to have low vision and on objective assessment the visual acuity was 3/60 in both eyes. Fundus fluorescein angiography was suggestive of capillary ‘drop-out’, vessel wall staining and leakage of dye.

**Results:** He was treated as SLE with major organ involvement. He received 5 doses of daily pulse injection methylprednisolone and 6 doses of monthly pulse injection cyclophosphamide therapy. After methylprednisolone he was initiated on oral prednisolone which was gradually tapered. Warfarin and Aspirin was also initiated (considering the possibility of occlusive retinal vasculopathy), though he had no antiphospholipid antibodies in the serum.

His vision continued to improve and 9 months on follow up, his objective visual acuity is 6/36. Fundus examination was suggestive of significant reduction in cotton wool spots and FFA showed significantly reduced leakage of the dye and improved perfusion of some of the previoulsy infarcted areas.

**Conclusion:** We report an unusual case of pediatric SLE with isolated retinal involvement. In the presence of consistently positive ANA, cotton wool spots, typical FFA findings and the fact that retinopathy has improved significantly after initiating immunosuppression (which almost rules out an infective etiology); the diagnosis of retinopathy of lupus seems justified. Though retinal involvement in pediatric lupus is well known but isolated involvemement of retina and no other systemic manifestations has not been reported previously. We emphasize the role of aggressive immunosppression for retinal involvement in SLE to improve the visual outcome.

**Disclosure of Interest**: None Declared

### B35 Two cases of systemic lupus erythematosus presenting with liver involvement

#### Elif Comak^1^, Gülşah Kaya Aksoy^1^, Aygen Yılmaz^2^, Atike Atalay^2^, Mustafa Koyun^1^, Reha Artan^2^, Sema Akman^1^

##### ^1^Pediatric Nephrology - Rheumatology, Akdeniz University, Antalya, Turkey; ^2^Pediatric Gastroenterology, Hepatology & Nutrition, Akdeniz University, Antalya, Turkey

###### **Presenting author:** Elif Comak

**Introduction:** The liver is generally not a major target organ in systemic lupus erythematosus (SLE). Nevertheless, autoimmune primary liver disease or SLE-related liver diasease may be seen in some cases.

**Objectives:** The aim of this study was to evaluate the liver involvement in children with SLE.

**Methods:** The children with the diagnosis of SLE were enrolled in the study. Information including demographic features, clinical and laboratory findings were collected from the hospital’s computerized database. Children with elevated liver enzymes caused by hepatotoxic drugs, coincident viral hepatitis and fatty liver disease were excluded from the study.

**Results:** A total of 40 children, 34 (85.0%) girls with a median age of 14 (mean 13.69 ± 3.46) years (5-18) were included in the study. Two children (5.8%) had liver involvement.

**Case I:** A 12 year-old girl was admitted with malaise, rash on the legs and swelling of left ankle which started one week prior to her presentation. On physical examination, she had hepatosplenomegaly, active arthritis of left ankle and nonspecific maculopapular rash on lower extremities. Her laboratory examination revealed elevated liver enzymes (ALT: 737 U/L, AST: 1018 U/L), increased total and direct bilirubin levels (3.34 mg/dl and 3.1 mg/dl, respectively), anemia, leukocytosis and thrombocytosis (hemoglobin 9.3 g/dl, WBC: 14.870/mm3, platelet count 495.000/mm3). Also, direct Coombs test was negative, ANA 1/1000 homogeneous positive, anti-dsDNA positive, ASMA (IFA) negative, anti- LKM negative, C3: 121 mg/dl, C4: 10.1 mg/dl. Liver biopsy showed chronic hepatitis with severe inflammatory activity characterized by portal infiltration by inflammatory cells, confluent necrosis, areas of spotty necroses and bridging fibrous in the parenchyma. We suggest that these findings were compatible with hepatic manifestations of SLE. Oral prednisolone, hydroxychloroquine and azathioprine were started.

**Case II:** A 14 year-old girls who had been treated with corticosteroids for 2.5 months and azathioprine for one month because of autoimmune hepatitis was referred to our center due to a rash on face, palms and fingers with thrombocytopenia. According to the her past medical history, elevated liver enzymes were detected in another center three months ago during routine assessment and liver biopsy was performed. Her systemic examination findings were unremarkable except of cushingoid appearence. On her laboratory investigations, she had mild elevation in liver enzymes (ALT: 46 U/L, AST: 49 U/L), total and direct bilirubin (3.08 mg/d Land 1.69 mg/dL, respectively), thrombocytopenia (platelet count 101.000/mm3), Direct Coombs negative, ANA 1/320 granular positive, ENA panels negative, ASMA-IFA negative, anti- LKM negative, C3: 78 mg/dL, C4: 9.7 mg/dL. These findings suggested an overlap syndrome involving SLE and autoimmune hepatitis. At follow-up, leukopenia (3550/mm3) and thrombocytopenia (61.000/ m3) developed, which was succesfully treated with IVIG.

According to the SLE classification criteria (SLICC - 2012), the first case had 3 clinical and 2 immunological criteria, the latter one had 2 clinical and 2 immunological criteria.

**Conclusion:** We suggest that differentiating hepatic involvement in SLE and autoimmune hepatitis has been challenging, as there are similarities in clinical features and biochemical parameters in both these entities.

**Disclosure of Interest**: None Declared

### B36 Neuropsychiatric systemic lupus erythematosus : a case report

#### Maria Francesca Gicchino, Giulia Macchini, Carmela Granato, Alma N. Olivieri

##### Department of Women, Chid and General and Specialistic Surgery, Second University of Naples, Naples, Italy

###### **Presenting author:** Maria Francesca Gicchino

**Introduction:** the term chorea defines a movement disorder characterized by not repetitive involuntary movements that may involve any portion of the body. The most common etiology of chorea in pediatric patients is the autoimmune form of post-streptococcical origin. Chorea may also be a complication of Systemic Lupus Erythematosus (SLE).

**Objectives:** to describe a case of pediatric SLE complicated by neurological involvement.

**Methods:** S., a 14 year old girl, was admitted to our division due to headache, involuntary movements of the upper and lower limbs, arthralgia, emotional lability. She had a history of fever, headache, fatigue, joint pain treated with antibiotics and low dose of costicosteroids. The objective examination revealed pharyngeal hyperemia, a 1/6 murmur on cardiac examination, involuntary, jerky movements of the limbs pain and functional limitation of the right hip. Suspecting a post-streptococcical chorea we performed a Doppler echocardiography that documented mild tricuspid regurgitation. Blood tests showed erythrocyte sedimentation rate (ESR) increased, presence of antinuclear antibodies (ANA), Anti dsDNA and IgM anticardiolipin.Because of persistent involuntary movements a brain MRI was performed which revealed on FLAIR images a hyperintensity focal lesion at the level of the left corona radiate. The EEG documented bioelectric anomalies in the left parietal lobe. SLE diagnosis was confirmed by the presence of the classification criteria and supported by neurological involvement. The treatment with intravenous methylprednisolone (30mg/kg/die for three days) rapidly improved the symptoms. Single photon emission computed tomography performed after three days of therapy showed a normal brain perfusion. Treatment was continued with oral prednisone (1mg/kg/die) gradually tapered, azathioprine (1,5 mg/kg/die), cardioaspirin (100mg/die). Two months later patient underwent a control MRI that documented a gliotic evolution in correspondence of the area of abnormal signal previously described.

**Results:** thanks to clinical, laboratory and instrumental data we diagnosed SLE with neurological involvement, in fact in our patient we found at least 4 of 11 ACR criteria needed to diagnose SLE. According to the Committee on Neuropsychiatric Lupus Nomenclature a neurological involvement was diagnosed for the presence of chorea and subclinical cerebral ischemia.

**Conclusion:** neuropsychiatric manifestations in SLE include psychiatric and neurological syndromes involving central, peripheral and autonomic nervous system. Neurological involvement has been reported to occur in 20% to 95% of patients with SLE. Chorea is the most common movement disorder correlated with the presence of antiphospholipid antibodies (aPLs). In our patient neurological involments is due to both aPLs and particular antibodies able to provoke anatomical or functional injury to the neurons.

**Disclosure of Interest**: None Declared

### B37 Successful use of rituximab in a male patient with systemic lupus erythematosus, ro-associated vasculitis and recurring episodes of macrophage activation syndrome

#### Maria I. Kaleda^1^, Irina P. Nikishina^1^, Sergei K. Soloviev^1^, Victor A. Malievsky ^2^, Ekaterina V. Nikolaeva^1^

##### ^1^Pediatrics, V.A. Nasonova Research Institute of Rheumatology, Moscow, Russian Federation; ^2^Pediatrics, Bashkortostan State Medical University, Ufa, Russian Federation

###### **Presenting author:** Maria I. Kaleda

**Introduction:** Systemic lupus erythematosus (SLE) is rare disease in children, especially at an early age in boys. Severe manifestations, such as macrophage activation syndrom (MAS) and Ro-associated vasculitis make the diagnostics and management of SLE difficult and challenging. New therapeutic regimens combining immunosuppressive agents and targeted B-cell depletion often provide improved disease control.

**Objectives:** In this report a case of early onset SLE in boy with MAS and Ro-associated vasculitis is presented.

**Methods:** Case report.

**Results:** A previously healthy 4-year-old Caucasian boy got sick on march of 2010: persistent fever up to 39°C with shiver unresponsive to antipyretics, polyarthritis, laboratory abnormality – Hb 96 g/l, platelets 178.0x10^9^/l, increase the level of TA-2N, CRP 20 mg/l, RF 30 mg/l. JIA, polyarthicular subtype, RF positive was diagnosed. He was admitted to the regional hospital and treatment with NSAIDs, GC iv 125 mg №3 + methotrexati iv 20 mg (once) was started. During the next three weeks laboratory results were constantly getting worse (pancytopenia - Hв 80 г/л, platelets 145.0x10^9^/l, leucocytes 1.0x10^9^/l, increase TA – 10N) and new clinical symptoms occurred (maculopapular rash, accompanied by itching; splenomegaly; recurrence of febrile fever; weight loss). An infectious process and leukosis were ruled out. This state was regarded as an allergic reaction. He received treatment with GC iv 25 mg/kg №3 + per os 20 mg daily + locally on the skin without significant effect. On 1^st^ admission in our clinic in September of 2010, the boy was easily excitable and whiny, physical exam revealed classic malar rash, palpable purpura on the body and legs, digital capillaritis, syndrom Rheino, enantema, myopathy, lymphadenopathy, hepatosplenomegaly, polyarthritis. The blood count showed Hb 96g/l, ESR 27 mm/h, ANA 1:640, anti-SS-A(Ro) > 200, RF 230 ME/ml. The revision of bone marrow biopsy showed typical MAS evidence. So the diagnosis of SLE according to SLICC criteria was established with MAS**.** The initial SLE Disease Activity Index (SLEDAI) was 29. Severe skin lesions were typical for Ro-associated vasculitis. The patient was treated by GC iv repeating courses, oral GC max 1,0 mg/kg, intravenous immunoglobulin repeating courses, DMARDs consecutively: cyclophosphamide iv + plaquenil, azathioprine + plaquenil, mycophenolate mofetil (MM) + plaquenil with incomplete and temporary respond. Rituximab (RTX) was introduced in November of 2013 in dose 375mg/m^2^ two doses two weeks apart. Patient received also GC per os 0.5 mg/kg and MM 500 mg/day, plaquenil 200 mg/day. Excellent result was achieved and persisted during 6 month after RTX introduction. It allowed to avoid the GC dose decreasing to 0.2 mg/kg. But disease relapses required repeating of RTX therapy each 6 months (in total 6 courses) with good efficacy and safety.

**Conclusion:** This clinical case demonstrated the combination of rare complications of SLE, such as early onset in boy, MAS as initial presentation and further Ro-associated vasculitis. It illustrated, that children may have more severe presentations of SLE than adults. Aggressive treatment strategy, including of GC, MM and RTX in a very young patient proved treatment success and seemed to be justified in similar cases.

**Disclosure of Interest**: None Declared

### B38 Juvenile systemic lupus erythematosus onset accompanied by hypocalcemia

#### Teresa Giani, Achille Marino, Gabriele Simonini, Rolando Cimaz

##### Pediatric, A. Meyer Children Hospital, Florence, Italy

###### **Presenting author:** Teresa Giani

**Introduction:** Hypocalcemia is a biochemical abnormality that can range in severity from asymptomatic to life-threatening situations. Serum calcium levels are regulated by 3 main hormones, parathyroid hormone, vitamin D, and calcitonin, through their specific effects on the bowel, kidneys, and skeleton.

**Objectives:** A 16-year-old female was admitted to our Hospital for a 2-months history of fatigue, arthralgia and hair loss. In the last two days before admission she started to be febrile, with a severe worsening of asthenia, and she began to complain palpitations and finger stiffness in both hands.

When examined, she was febrile (39°C), her pulse rate was 110 beats per minute, and her blood pressure was 104/64 mmHg. Her heart and breathing sounds were clear. The abdomen was flat and soft, and liver and spleen were not palpable. She presented a rash with a malar distribution, and a nasal ulcer with septal involvement, and a diffuse bright red discoloration of the gingiva were observed. Pain at knees, wrists, and ankles was referred during muscolo-skeletal examination, without evidence of effusion, nor limitation of motion.

Neurologic exam, Trousseau and Chvostek signs were negative.

**Methods:** A complete blood count showed a white cell count of 1.60 × 10^3^/μL, hemoglobin 10.3 g/dL, hematocrit 34.8%, and platelets 140,000/μL. ESR, 110 mm/h (0-20 mm/h); C-reactive protein, 4.4 mg/dl (0.00-0.30 mg/dl). There was no renal and liver dysfunction.

High anti-nuclear antibody, anti-double-stranded DNA antibody and anti-Smith antibody titers were documented with low complement levels, and positive direct Coombs’ test. An active sediment was noted on the basis of urinalysis, and proteinuria was 0.8 g/day.

Blood chemistry revealed total serum calcium level 5,8 mg/dL (8.4-10.2 mg/dL, with free calcium level 2,8 mg/dl), phosphate level 3 mg/dL (2.9–4.8 mg/dL), magnesium level 2.2 mg/dL (1.8–2.4 mg/dL), alkaline phosphatase level 380 U/L (134–359 U/L), and albumin 3,9 g/dL (3.5-5 g/dL). Serum 25(OH)D level was decreased (10 ng/mL), and parathyroid hormone PTH resulted intact (68 pg/mL, nv 8-76 pg/mL). TSH was 50 U/ml (0.27-4.20 U/ml), T3 was 3 ng/ml (0.80-2.00 ng/ml), and T4 was 0.5 ug/dl (5.10-14.10 ug/dl), anti-thyroperoxidase antibodies were 1600 IU/mL (normal <34 IU/mL).

**Results:** Electrocardiogram revealed a QTc interval of 0.45 sec (normal level 0.37–0.44).

**Conclusion:** Based on clinical manifestation, history and laboratory test the diagnosis of SLE was therefore established in association with a severe and unclear hypocalcemia.

The patient was immediately treated with intravenous methylprednisolone pulses and oral hydroxychloroquine, associated with 1g/day for three days of calcium gluconate followed by oral calcium (500 mg/day) and 0.5 μg/day of *1*,*25*-dihydroxy *cholecalciferol*

Serum calcium gradually recovered in one week, simultaneously with the progressive improvement of general conditions and thyroid function.

In this girl hypocalemia was quite pronounced although not seriously symptomatic and transient. The underlying cause of decline in serum calcium level is unclear.

Hypocalcemia has very rarely been reported in association with lupus, and usually it was the result of hypoparathyroidism. However, in our patient parathyroid hormone maintained in normal range, as well as magnesium, phosphate, and albumin. Renal function was conserved and kidney biopsy ruled out a tubular injury.

Therefore, we suspected that the patient’s hypocalcemia was mainly caused by a pre-existing vitamin D deficiency, probably due to an insufficient dietary intake, in addition to a concomitant thyroid imbalance, probably characterized by an initial phase of hyperthyroidism followed by hypothyroidism.

**Disclosure of Interest**: None Declared

### B39 Etanercept treatment in patient with juvenile idiopathic arthritis and interstitial lung disease

#### Agnieszka Gazda, Beata Kołodziejczyk, Lidia Rutkowska-Sak

##### Paediatric Rheumatology Clinic, National Institute of Geriatrics, Rheumatology and Rehabilitation, Warsaw, Poland

###### **Presenting author:** Agnieszka Gazda

**Introduction:** Patient with JIA polyarticular onset and interstitial lung disease. The onset of the disease in 2007(10th year of life)-insidious

**Objectives:** Since 2011 (after pertussis) the course of the disease aggressive with inflammation in almost all peripheral joints and cervical spine, ESR-60mm/h, RF -4160 IU/ml, antyCCP-218 U/ml. In ultrasound persistent active inflammation, in X-ray destructive changes in joints(erosions, geodes, narrowing of the joint space). Due to atrophy of the arm muscles, discreet deterioration of muscle strength in forearms, transient changes in emg (myogenic damage in left deltoid) a history of polymyositis cannot be excluded.

**Methods:** Since 2007 HRCT has revealed interstitial changes in the lungs: micronodular and reticular changes causing mild bronchiectasis in pulling, enlarged lymph nodes paratracheal. In Bodyplethysmograhpy: restrictions type of changes, DLCO- 82%. Tuberculosis, sarcoidosis, other infections were excluded (BAL).

In Nov 2011 the patient had lung biopsy which showed : reactive hyperplasia of follicular lymphoid tissue around the bronchioles, small inflammatory infiltrates in alveolar septums and fibrosis in underpleural areas.

**Results:** The treatment was modified: prednison, azathiopryne + chlorochine, methotrexate + Cyclosporin A, cyclophosphamid iv -6 months + IVIG 3 months with no improvement.Since 2012 the patient has been treated with etanercept,

**Conclusion:** On etanercept treatment the activity of the inflammatory process in the joints decreased (CHAQ reduction from 3 to 0.250), reduction of inflammatory markers was obtained. In HRCT after 2 years of treatment (2014) partial regression of interstitial changes was noticed.

There were no side effects of etanercept treatment.

**Disclosure of Interest**: None Declared

### B40 Kawasaki disease in an adolescent presenting with adenitis and rash

#### Angela Mauro^1^, Teresa Giani^2^, Gabriele Simonini^2^, Rolando Cimaz^2^

##### ^1^Rheumatology Unit, Second University of Naples, Naples, Italy; ^2^Rheumatology Unit, Anna Meyer Children’s Hospital, Florence, Italy

###### **Presenting author:** Angela Mauro

**Introduction:** Kawasaki disease is a systemic vasculitis with an acute and self-limited course that affecting medium and small size arteries.

**Objectives:** A 14 year old girl was admitted to our hospital with fever for approximately 7 days, right cervical adenopathy and an erythematous rash. She reported a febrile, flu-like episode one month ago. One week before, she developed right cervical adenopathy. After 3 days, a cutaneous rash appeared on the lower limbs and she became febrile. She was seen by a general practitioner, who suggested a neck ultrasound and anti-inflammatory therapy. The girl then presented at an Emergency Department. At physical examination, the patient appeared in good general conditions but was febrile, had right cervical lymphadenopathy and an erythematous rash. Blood tests were performed and notable results included increased values of CRP (6 mg/dl, nv < 0.5) and neutrophilic leukocytosis. Liver function tests, blood clotting tests, LDH and bilirubin were within normal range. Infectious investigations were negative (serologies for EBV, CMV, Borrelia, Rickettsia, Parvovirus B19, Measles, Rubella, HHV6-HHV7, Coxackie, Hepatitis B, ASO, blood cultures, throat swab). Chest X-ray was normal and neck ultrasound showed multiple vascularized lymph nodes of about 2 cm in diameter with an unrecognizable hilum.

**Methods:** She was admitted to our Hospital for further investigations. On physical examination, she presented in fairly good general condition, with a right cervical adenopathy non purulent conjunctivitis and a macular erythematous rash at upper and lower limbs, involving the palms and soles with edema of the feet. Cardiac, pulmonary, and abdominal examinations were negative. Blood tests revealed increased C-reactive protein (3.75 mg/dl), normal erythrocyte sedimentation rate, mild normocytic normochromic anemia (Hb 10.9 g/dl), increased platelet count (717,000/mm^3^), hypereosinophilia (eosinophils 21.1%); Liver and renal function were normal. Blood cultures and infection serologies were again negative. She underwent an echocardiography that showed enlarged coronary diameter, (descending anterior 3.1 mm, right 2.8 mm, left 3 mm; worse Z score +2). Three days later echocardiography also showed hyperechoic aspect and increased wall thickness of posterior basal left ventricle, where there was also a small epi-pericardial separation. Left coronary artery showed circumscribed dilatation of proximal descending anterior coronary (5.5 mm), and right coronary diameter was increased (4 mm).

**Results:** For the presence of fever >5 days, lymphadenopathy, conjunctivitis, cutaneous rash, edema of the feet with cardiac involvement we diagnosed complete Kawasaki disease and started IVIg (2 g/Kg) and Aspirin (80 mg/Kg). After the first IVIg dose, she became afebrile, but one day later fever reappeared (T Max 39°C) and a second dose of IVIg was administered. After the second dose the patient quickly became afebrile, and rash and conjunctivitis gradually disappeared. Serological markers of inflammation returned in the normal range. The epicardial separation disappeared while the coronary dilatation was still present. At last follow-up, the patient was in good general conditions, afebrile, asymptomatic, but echocardiography showed a right coronary dilatation (4 mm diameter). She is on antiplatelet therapy, that she’ll need to continue until the cardiologist determines it will be necessary.

**Conclusion:** The patient suffered from complete Kawasaki Disease with cardiac involvement. Predominantly it affects children < 5 years of age and the outcome depends on to the cardiac involvement. Several studies have demonstrated that if the onset of disease is <1 year or > 5 years of age the outcome is worse because these patients have a greater risk of coronary aneurysm involvement.

**Disclosure of Interest**: None Declared

### B41 Granulomatosis with polyangiiitis: case report

#### Maria Francesca Gicchino, Pierluigi Marzuillo, Stefano Guarino, Alma N. Olivieri, Angela La Manna

##### Department of Women, Chid and General and Specialistic Surgery, Second University of Naples, Naples, Italy

###### **Presenting author:** Maria Francesca Gicchino

**Introduction:** Granulomatosis with Polyangiitis (GPA), previously known as Wegener’s granulomatosis (WG), is a chronic vasculitis affecting predominantly small vessels. It is characterized by granulomatous inflammation of the upper and lower respiratory tracts, pauci-immune necrotizing glomerulonephritis, vasculitis that involves other organs and the presence of anti-neutrophil cytoplasmic antibodies (ANCA). In children, the estimated incidence is approximately 0.1:100,000

**Objectives:** to describe a case of Granulomatosis with Polyangiitis in an 8 year old child.

**Methods:** an 8 year old child, was admitted to our hospital due to fever and fatigue. The patient was in treatment with L-tiroxina because of autoimmune tiroiditis from the age of six years. She had a history of fever, weight loss, fatigue, rash, treated with antipiretic drugs. Blood tests previously done documented increased erythrocyte sedimentation rate (ESR). When she was admitted to our structure she presented pharyngeal hyperemia, a 1/6 murmur on cardiac examination. Laboratory examinations documented increased serum creatinine (2,06 mg/dl), ESR (125mm/h), anemia(Hb 9,5 g/dL). Serum complement, antinuclear antibodies, anti ds-DNA were negative, while pANCA were elevated. Urinanalysis revealed microscopic hematuria and proteinuria. A protein to creatinine ratio measurement in the patient’s 24 hours urine was 1mg/mg. An ultrasound of the abdomen showed normal sized kidneys with normal cortico-medullary differentiation. A renal biopsy was performed and a kidney fragment with 33 glomeruli was evaluated. In about 19 glomeruli sclerotic involution was documented, in 10 glomeruli segmental sclerosis, segmental fibrinoid nescrosis and crescent formation, 4 glomeruli were normal; the tubules with mild atrophy contained hematic casts; the interstitial showed excess of mixed inflammatory cells. Kidney biopsy results suggest a pauci immune glomerulonephritis with crescent formation. A lung CT scan revealed diffuse interstitial infiltrate in both lungs. ENT evaluation was normal.

**Results:** according to EULAR/PReS criteria for the classification of childhood vasculitis was diagnosed Granulomatosis with Polyangiitis, previously known as Wegener’s Granulomatosis. Immunosuppressive therapy was started with intravenous methylprednisolone (30mg/kg/die for three days) following by intravenous cyclophosphamide. Blood tests performed after two weeks of treatment showed a normalization of ESR, hemoglobin and an improvement of serum creatinine (0,85mg/dL). A protein to creatinine ratio measurement in the patient’s 24 hours urine was 0,94mg/mg.

**Conclusion:** Granulomatosis with Polyangiitis although rare in childhood should be considered in the above clinical scenario to avoid complication due to a diagnosis delay.

**Disclosure of Interest**: None Declared

